# Genome-Wide Characterization and Expression Profiling of Sugar Transporter Family in the Whitefly, *Bemisia tabaci* (Gennadius) (Hemiptera: Aleyrodidae)

**DOI:** 10.3389/fphys.2017.00322

**Published:** 2017-05-23

**Authors:** Zezhong Yang, Jixing Xia, Huipeng Pan, Cheng Gong, Wen Xie, Zhaojiang Guo, Huixin Zheng, Xin Yang, Fengshan Yang, Qingjun Wu, Shaoli Wang, Youjun Zhang

**Affiliations:** ^1^College of Plant Protection, Hunan Agricultural UniversityChangsha, China; ^2^Department of Plant Protection, Institute of Vegetables and Flowers, Chinese Academy of Agricultural SciencesBeijing, China; ^3^Department of Biocontrol, Institute of Plant Protection, Heilongjiang Academy of Agricultural SciencesHarbin, China; ^4^Key Laboratory of Bio-Pesticide Innovation and Application of Guangdong Province, Department of Entomology, South China Agricultural UniversityGuangzhou, China; ^5^Key Laboratory of Molecular Biology of Heilongjiang Province, College of Life Sciences, Heilongjiang UniversityHarbin, China

**Keywords:** *B. tabaci*, sternorrhynchan insect, sugar transporter, phylogenetic analysis, expression pattern

## Abstract

Sugar transporters (*STs*) play pivotal roles in the growth, development, and stress responses of phloem-sucking insects, such as the whitefly, *Bemisia tabaci*. In this study, 137 sugar transporters (*STs*) were identified based on analysis of the genome and transcriptome of *B. tabaci* MEAM1. *B. tabaci* MEAM1 encodes a larger number of *STs* than other selected insects. Phylogenetic and molecular evolution analysis showed that the 137 *STs* formed three expanded clades and that the genes in Sternorrhyncha expanded clades had accelerated rates of evolution. *B. tabaci* sugar transporters (*BTSTs*) were divided into three groups based on their expression profiles across developmental stages; however, no host-specific *BTST* was found in *B. tabaci* fed on different host plants. Feeding of *B. tabaci* adults with feeding diet containing dsRNA significantly reduced the transcript level of the target genes in *B. tabaci* and mortality was significantly improved in *B. tabaci* fed on dsRNA compared to the control, which indicates the sugar transporters may be used as potential RNAi targets for *B. tabaci* bio-control. These results provide a foundation for further studies of *STs* in *B. tabaci*.

## Introduction

In multicellular organisms, sugars provide energy and carbon skeletons but are also involved in signaling and osmotic processes. Sugar, however, cannot directly pass through the cell membrane and the exchange of sugars between cells or between subcellular structures is mediated by sugar transporters (*STs*). Sugar transporters belong to the major facilitator superfamily (MFS). The MFS contains at least 24 distinct families with more than 249,360 sequenced members and shares a conserved 12 transmembrane domains, which is organized into two pseudosymmetrical halves (Yan, 2015). Because they are involved in sugar transport, *STs* are often the focus of MFS studies. In humans, *STs* are related to diabetes and have therapeutic importance because they mediate sugar movement across cancer cells (Wright et al., 2007). In plants, *STs* are critical for crop yield and are involved in resistance against pathogens (Doidy et al., 2012; Schroeder et al., 2013).

The whitefly *Bemisia tabaci* (Gennadius) is a globally distributed polyphagous pest that includes a number of genetically divergent but morphologically indistinguishable species (De Barro et al., 2011; Liu et al., 2012; Boykin and De Barro, 2014). Two of these species, MEAM1 (formerly cryptic species “B”) and MED (formerly cryptic species “Q”), are major agricultural pests in more than 60 countries (Chu et al., 2006; De Barro et al., 2011; Pan et al., 2011, 2015). *B. tabaci* damages plants both directly by sucking phloem sap and indirectly by transmitting plant viruses (Jones, 2003; De Barro et al., 2011; Pan et al., 2012a). As a diet, phloem sap contains low levels of nitrogen and essential amino acids. In the pea aphid, *Acyrthosiphon pisum*, for example, two *STs* were reported to be up-regulated in the bacterocyte (Hansen and Moran, 2011). The bacterial symbionts in phloem-feeding insects provide the essential amino acids and vitamins that compensate for the lack of essential amino acids in phloem sap (Baumann, 2005; Pan et al., 2012b). Sugar transporters are very important for *B. tabaci*. First, *B. tabaci* ingests only plant sap, which contains sucrose (Byrne and Miller, 1990; Hayashi and Chino, 1990), the ingested sucrose, however, cannot directly pass across the gut epithelium and is hydrolyzed into glucose and fructose in the gut. Transport of glucose and fructose across the gut epithelium is mediated by *STs* (Ashford et al., 2000; Cristofoletti et al., 2003; Price et al., 2007a). In addition, trehalose, the principal sugar circulating in the blood or haemolymph of most insects (Thompson, 2003), is also transported by *STs* (Kikawada et al., 2007). Thus, *STs* are essential for sugar exchange in *B. tabaci*. Second, although the concentration and osmotic pressure of phloem sap varies among plants, the osmotic pressure is always higher in phloem sap than in the phloem-feeding insect (Douglas, 2006). To prevent loss of water in response to this difference in osmotic pressure, phloem-feeding insects have developed an osmoregulation mechanism. Sugar transporters contribute to osmoregulation by importing sugars, mainly glucose, from the gut lumen into cells (Price et al., 2007b; Kikuta et al., 2015; Tzin et al., 2015). Third, the interactions between *B. tabaci* and virus may require *STs*. Sugar transporters are receptors for human T cell leukemia virus and white spot syndrome virus (Manel et al., 2003; Huang et al., 2012). A recent study found that an *ST* was up-regulated in *Bombyx mori* infected with *B. mori* nucleopolyhedrovirus and that the expression levels of three *STs* differed in virus-susceptible vs. -resistant strains (Sagisaka et al., 2010; Govindaraj et al., 2016). Although five genera of plant viruses are transmitted by *B. tabaci* (Jones, 2003), the interactions between *B. tabaci* and viruses are unclear at the molecular level but might involve *STs*.

Several recent studies have reported on *STs* from insects (Kikawada et al., 2007; Liu et al., 2013; Govindaraj et al., 2016), including phloem-feeding insects (Price et al., 2007b, 2010; Kikuta et al., 2010; Price and Gatehouse, 2014; Ge et al., 2015). For example, genomic studies identified *STs* in the pea aphid, *A. pisum* (Price et al., 2010; Price and Gatehouse, 2014). Among those *STs, APST3*, and *APST4* were determined to be highly expressed in the gut (Price et al., 2010; Price and Gatehouse, 2014). Functional analysis showed that *APST3* and *APST4* were a low affinity transporter and a high affinity transporter of glucose and fructose, respectively (Price et al., 2010; Price and Gatehouse, 2014). In another study, 18 putative *STs* were identified in the brown planthopper, *Nilaparvata lugens*, and seven of them were highly expressed in the gut (Kikuta et al., 2010). Further analysis revealed that *NlST1* and *NlST16* were glucose transporters and that *NIST6* was a fructose transporter (Price et al., 2007b; Kikuta et al., 2010, 2015).

In the current study, a genome-wide approach was used to investigate the *STs* in *B. tabaci*. Our overall goal was to increase our understanding of these *STs* and to identify *ST*s that might serve as targets for controlling *B. tabaci*. We first identified *STs* in *B. tabaci* MEAM1 (hereafter *BTSTs*) based on its genome and transcriptomic datasets. In addition, *STs* from the following 11 other insect species were identified based on their genomes: *A*. *pisum, Diaphorina citri, Nasonia vitripennis, Apis mellifera, Musca domestica, Drosophila melanogaster, Anopheles gambiae, Aedes aegypti, Manduca sexta, Plutella xylostella*, and *B. mori*. Next, the phylogenetic relationships were analyzed among *BTSTs* and the *STs* from seven other insect species, and evolutional pattern of expanded *BTSTs* were investigated. The gene expression patterns of *BTSTs* were then measured based on the transcriptome datasets, which included *B. tabaci* samples from different developmental stages and different host plants. Reverse transcriptase-quantitative polymerase chain reaction (RT-qPCR) was used to verify the *BTSTs* expression patterns across different stages. Finally, we determined the effect of orally delivered dsRNAs of nine *BTSTs* on the motality of *B. tabaci* MEAM1.

## Materials and methods

### *De novo* identification of *STs* in *B. tabaci* and other insects

The version of the genome of China *B. tabaci* MEAM1 (http://111.203.21.119/download/) were annotated by homolog search and *de novo* predicting and transcriptome/EST correspondence. To search for all *STs* in the reference protein dataset, predicted proteins that matched the Pfam HMM (Hidden Markov model) profile of sugar transporter (PF00083) were identified by hmmsearch (HMMER software, version 3.1b1) with an *E*-value <0.001 (Eddy, 2011; Finn et al., 2016), and the identified *STs* were used as query to search the *B. tabaci* MEAM1 genome again to avoid missing annotation. Next, the identified 228 putative proteins were annotated by blasting against the NR database (Version 2015.6) with the blastp program (Altschul et al., 1997). Proteins that did not match known *STs* with an *E*-value <0.001 were excluded. After that, the identified *STs* were searched against *B. tabaci* transcripts database (Xie et al., 2014; SRP064690) [*de novo* assembled using the transcriptome sequencing data by Trinity software (Haas et al., 2013)] using the tblastn program (Altschul et al., 1997). Then, manual corrections were preformed. Finally, whether the corrected *STs* contained TIGR00879 with a score above 237.80 and an *E*-value <0.001 was determined using hmmsearch (HMMER software version 3.1b1) (Haft et al., 2001; Eddy, 2011). Trans-membrane regions were predicted using the TOPCONS Server (http://topcons.cbr.su.se/) (Bernsel et al., 2009).

To compare the composition and diversity of *BTSTs* with *STs* from other insects, *STs* were identified in the reference protein datasets of 11 other insect species using the pipelines described above. The information of the selected datasets is provided in Table S1.

### Insect strain and RNAseq samples preparation, RNA isolation, cDNA library construction, and illumina sequencing

The strain of *B. tabaci* MEAM1 BtB1-China population was originally collected in 2004 from cabbage, *Brassica oleracea* cv. Jingfeng1, in Beijing and was subsequently reared on cotton in separate screen cages in a glasshouse. The purity of the culture was monitored every 2–3 generations based on haplotypes of the DNA sequence obtained using *mtCOI* primers (Chu et al., 2010).

For The different developmental stage samples (eggs, L1–L2, L3, L4 nymph, females, and males) were collected from BtB1-China population. Since distinguishing first and second nymph is laborious, a mixture of first and second nymphs was collected as one sample. Only 2-h-old adults were collected using a glass tube (5 × 0.5 cm), the sex was determined under a stereo microscope and same sex female or males pooled into a plastic tube using an aspirator. Total RNA was extracted using Trizol reagent (Invitrogen) according to the manufacturer's instructions, and purity and degradation were checked on 1% agarose gels. RNA-seq libraries were constructed as previously described (Xie et al., 2014) and sequenced on a HiSeq 2500 system according to the manufacturer's instructions with sequenced at 125 bp (PE125, library size is 280–320 bp).

### Genome-wide expression analysis of *BTSTs*

The expression patterns of *BTSTs* were investigated by using a series of different developmental stage transcriptome sequencing data (SRP064690) as described above and *B. tabaci* from different host plants (Xie et al., 2014). Initially, all of the transcriptome datasets were filtered with Trimmomatic with default parameters (Bolger et al., 2014). The clean data were then mapped to the *B. tabaci* MEAM1 genome by TopHat v2.1.0 (Trapnell et al., 2009). Transcript expression, represented as FPKM, was calculated using Cufflinks v2.2.1 without taking splicing isoforms into account (Trapnell et al., 2012). Finally, The FPKM values were log transformed, and genes were clustered in term of expression patterm by Cluster 3.0 and visualized by Treeview software (Saldanha, 2004).

### Phylogenetic analysis of *STs*

To understand the phylogenetic relationship of *BTSTs* with *STs* from other insects, amino acid sequences of *BTSTs* and *STs* of seven other insect species were used to conduct phylogenetic analysis. To perform a high quality phylogenetic analysis, full-length sequences that contained > 250 amino acid residues were aligned by MAFFT software v7.221 with the L-ins-I method (Katoh et al., 2002). The alignment was trimmed in Trimal using a gap throughout of 25% (Capella-Gutierrez et al., 2009). The phylogenetic tree was estimated using a maximum-likelihood (ML) method. Before the phylogenetic analysis was performed, the best-fit model LG+I+G+F was determined by Prottest 3.4 (Abascal et al., 2005). ML analysis was then conducted by Raxml V8.2.4 with 1,000 replicates using the fast bootstrap option (Stamatakis, 2014). Finally, the result was visualized by iTOL (Letunic and Bork, 2007).

### Positive selection and *ST* gene conservation analysis

To further understand the *BTSTs* expanded clades, molecular evolutional rates of those *STs* were estimated. Before the molecular evolutionary analysis, the amino acid sequences of the expanded *STs* were aligned by MAFFT, and their phylogeny tree was constructed by Mrbayse V3.2 (Huelsenbeck and Ronquist, 2001). Because the LG protein substitution matrix is not available in Mrbayse, the phylogeny was constructed using WAG+F+I+G. Finally, the tree was used as the input file for further molecular evolution analysis.

In our study, the branch-based models and site-based models of molecular evolution were selected and analyzed by Paml 4.9a software (Yang, 2007). Non-synonymous/synonymous substitution rate ratios (dN/dS) of all selected *STs* were estimated based on their coding sequence (CDS). A dN/dS ratio >1 indicates positive selection, a dN/dS ratio <1 indicates purifying selection, and a dN/dS ratio = 1 indicates neutral evolution. Both branch-based models and site-based models were tested as described previously (Price et al., 2011). In brief, for branch models of molecular evolution, a series of ω categories were calculated (model = 0 or 2, NS sites = 0). The other parameters, ω and κ, started from 0.2 to 2, respectively, and the coding frequency was using 3 ^*^ 4 codon table. The bayesian trees of *BTST* expanded clades with assigned ω were shown in Figure S1 and the models of molecular evolutional test were shown in Table S2. For sie-based models of molecular evolution, three pairs of models were used: M0/M3, M1a/M2a, and M7/M8.

### RNA extraction and cDNA synthesis

Total RNA was extracted from different stages of *B. tabaci* using TRIzol reagent following the manufacturer's recommendation (Invitrogen, Carlsbad, CA, USA). Gel electrophoresis and a NanoDrop 2000 c spectrophotometer (Thermo Fisher Scientific Inc., Waltham, MA, USA) was used to determine the quantity of RNA. PrimeScript™ II 1st strand cDNA Synthesis Kit (TaKaRa, Dalian, China) and PrimeScript RT kit (containing gDNA Eraser, Perfect Real Time, TaKaRa, Dalian, China) were used for preparing first-strand cDNA for cloning and gene expression analysis, respectively. The first-strand cDNA was stored at −20°C until used.

### Cloning of *BTSTs*

Primers of selected *BTSTs* (Table S3) were designed based on their complete coding sequences. PCR reactions were performed in an S1000 TM Thermal Cycler PCR system with the following parameters: initial denaturation at 94°C for 10 min; followed by 35 cycles of denaturation at 94°C for 60 s, annealing at 55–60°C for 45 s, and extension at 72°C for 120 s; and a final extension at 72°C for 10 min using La-Taq polymerase (TaKaRa, Dalian, China). For *BTST40* and *BTST107*, the PCR reaction system (25 μl volume in total) contained 2 μl of cDNA template, 2.5 μl of 10 × PCR buffer (Mg^2+^), 4 μl of dNTP mixture (2.5 mM), 0.25 μl of La-Taq, 1 μl of each primer (10 mM), and 14.25 μl of ddH_2_O. For the seven other *BTSTs*, the PCR reaction system (25 μl total volume) contained 2 μl of cDNA template, 12.5 μl of 2 × GC I buffer (Mg^2+^), 2 μl of dNTP mixture (2.5 mM), 0.25 μl of La-Taq, 1 μl of each primer (10 mM), and 6.25 μl of ddH_2_O. Amplicons of the expected bands were excised from 1.5% agarose gel, purified using the TIANgel Midi Purification Kit (TIANGEN, Beijing, China), and subcloned into the pEASY-T1 vector (Transgen, Beijing, China) before transformation into *Escherichia coli* TOP10 competent cells (Transgen, Beijing, China) for sequencing.

### RT-qPCR analysis

The RT-qPCR (ABI 7500 Real-Time PCR system, Applied Biosystems, USA) was used and specific primers (Table S4) were designed to investigate the expression levels of 15 *BTSTs*. The reaction mixture (25 μl) contained 9.5 μl of ddH_2_O, 12.5 μl of 2 × SuperReal PreMix Plus (TIANGEN, Beijing, China), 7.5 μM of both upstream and downstream primers, 1 μl of first-strand cDNA template, and 0.5 μl of 50 × ROX Reference Dye (TIANGEN, Beijing, China) based on the manufacturer's recommendations. The RT-qPCR program had the following parameters: initial denaturation for 15 min at 95°C followed by 40 cycles of denaturation at 95°C for 15 s, annealing for 30 s at 59–60°C, and extension for 32 s at 72°C. Finally, an automatic dissociation step for melting curve analysis was used. The standard curve for each candidate gene was generated from cDNA 2-fold serial dilutions (1/1, 1/2, 1/4, 1/8, and 1/16). The corresponding RT-qPCR efficiencies (E) are expressed as percentages according to the equation: E = (10^[−1/slope]^ –1) × 100. Primer sets that showed a single peak in melting curve analyses, 90–110% primer amplification efficiencies, and > 0.95 coefficients (*R*^2^ values) were used for subsequent experiments. Conventional PCRs were performed before RT-qPCRs. The amplified fragments were sequenced to confirm. Four technical replicates and three biological replicates were used for each treatment. For negative control reactions, ddH_2_O was used in place of the cDNA template. Data were normalized to the *elongation factor 1 alpha* gene (Li et al., 2013), and relative gene expression was calculated using the 2^−ΔΔCt^ method (Livak and Schmittgen, 2001).

### RNA interference

Based on the genomic and transcriptome data, unique regions of selected *STs* were used as templates for dsRNA synthesis. The dsRNA for enhanced green fluorescent protein (EGFP) was used as the negative control. Primers are listed in Table S5. DsRNAs were prepared according to the T7 RiboMAX Express RNAi system protocols (Promega, Madison, USA). The RNAi bioassay was performed by directly feeding dsRNA to *B. tabaci* adults in a feeding chamber for 4 days. A 0.20-ml drop of diet solution that combined 5% yeast extract and 30% sucrose (wt/vol) with 100 ng of dsRNA was placed in the chamber. The details of feeding were performed as described in previous studies (Yang et al., 2016). Approximately 50 adults (2 days old, mixed sexes) were used for each *ST* gene treatment, and each treatment was represented by three biological replicates. The experiment was conducted in an environmental chamber at 25°C, a photoperiod of L14: D10, and 80% RH. After 2 and 4 days, mortality was recorded and *B. tabaci* specimens were collected for analysis of the expression levels of targeted *ST* genes.

### Statistical analysis

One-way ANOVA was used to compare the gene expression levels and mortality rates among different treatments. Means were compared with LSD tests at *P* < 0.05. SPSS version 20.0 (SPSS Inc., Chicago, IL, USA) was used for statistical analyses.

## Results

### Identification of *BTSTs*

**Figure 1:**
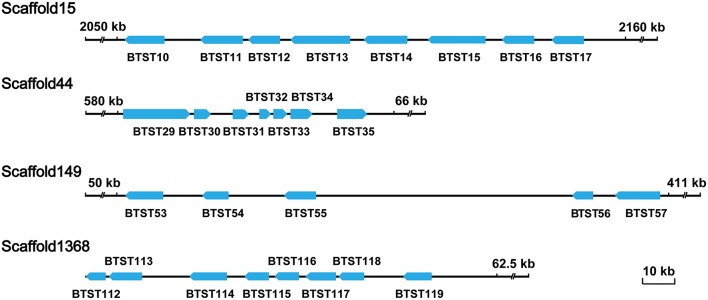
**Location of tandem repeat *BTSTs* on scaffolds**. The blue arrows indicate the transcriptional orientations of the *BTSTs* on the scaffolds. One *BTST* cluster was identified on each of four scaffolds (15, 44, 149, and 1,368).

To obtain a genome-wide identification of *BTSTs*, we searched the predicting protein sequences of *B. tabaci* for the PF00083 (Sugar and other transporter). This resulted in the identification of coding sequences. Blasting against non-redundant protein 176 sequence (Nr) database subsequently showed that these coding sequences could be divided into three sub-families: *STs*, organic cation transporters, and synaptic vesicle proteins. Coding sequences that matched *STs* were then selected and manually corrected. Finally, 137 *BTSTs* were identified (Tables S7–S9). Among them, nine proteins matched the sugar transporter family motif TIGR000879 with scores above the cutoff. Sequence analyses revealed that most of the *BTSTs* contained the 12 transmembrane and that all of the *BTSTs* genes contained multiple exons except the gene coding *BTST65*, which was intron-less. In addition, some *BTSTs* formed different gene clusters that were located on different scaffold; for example, *BTST10* to *BTST17, BTST29* to *BTST35, BTST53* to *BTST57*, and *BTST112* to *BTST119* were located on scaffold 15, 44, 149, and 1368, respectively (Figure 1). The tandem repeated pattern can be accounted for the expansion of *BTSTs*. Based on *B. tabaci* transcriptome datasets that were derived from different developmental stages and different host plants, 134 coding sequences had transcriptome coverage, suggesting that they were expressed in *B. tabaci*.

### Expression profiles of *BTSTs*

**Figure 2:**
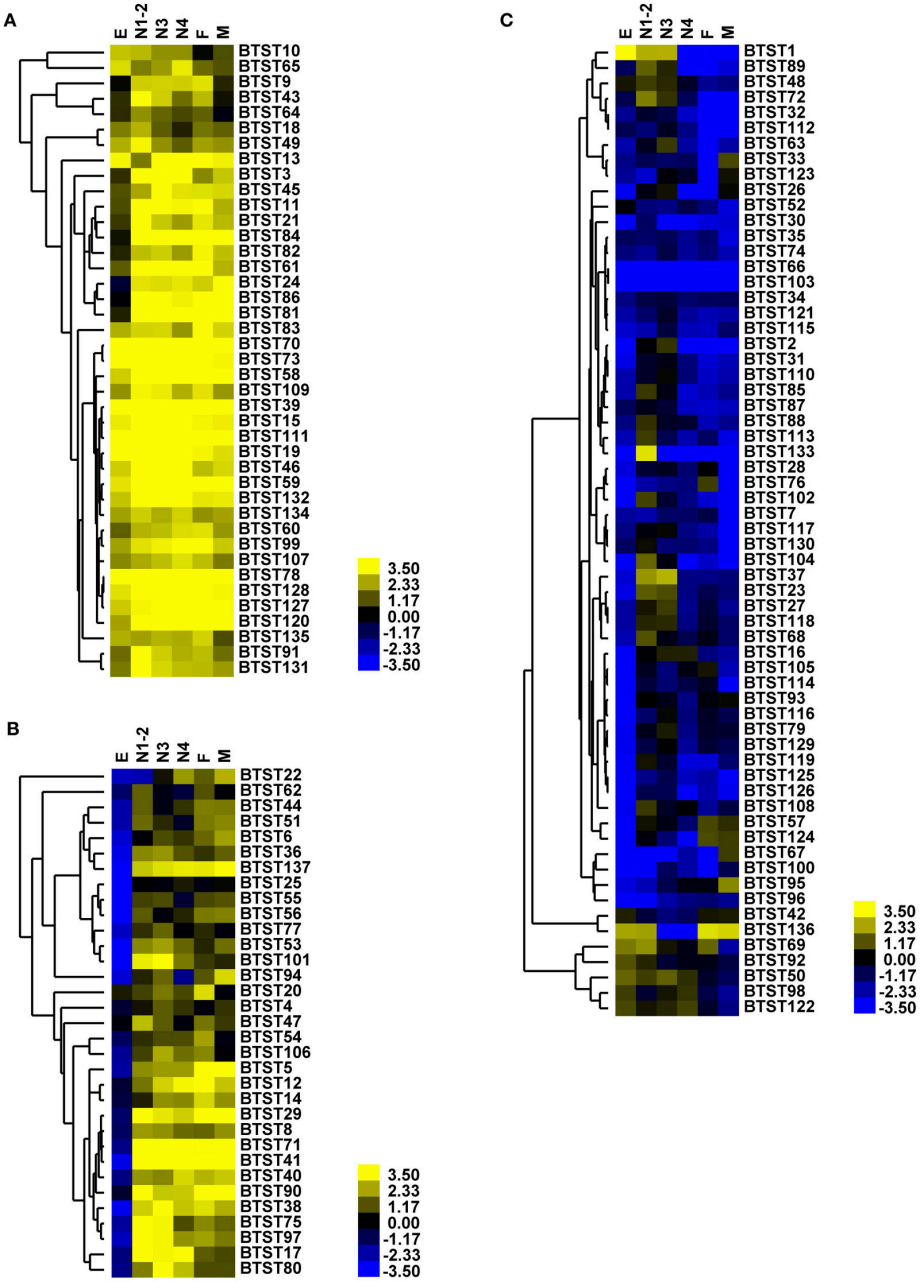
**Gene expression of *BTSTs* across different developmental stages**. The log_2_(FPKM) values are presented as a heatmap. Bright yellow indicates higher expression values, dark blue indicates lower expression values, and gray indicates missing values. E, eggs; N1-2, 1st and 2nd instar nymphs; N3, 3rd instar nymphs; N4, 4th instar nymphs; M, males; F, females. **(A)** Group 1; **(B)** Group 2; **(C)** Group 3.

**Figure 3:**
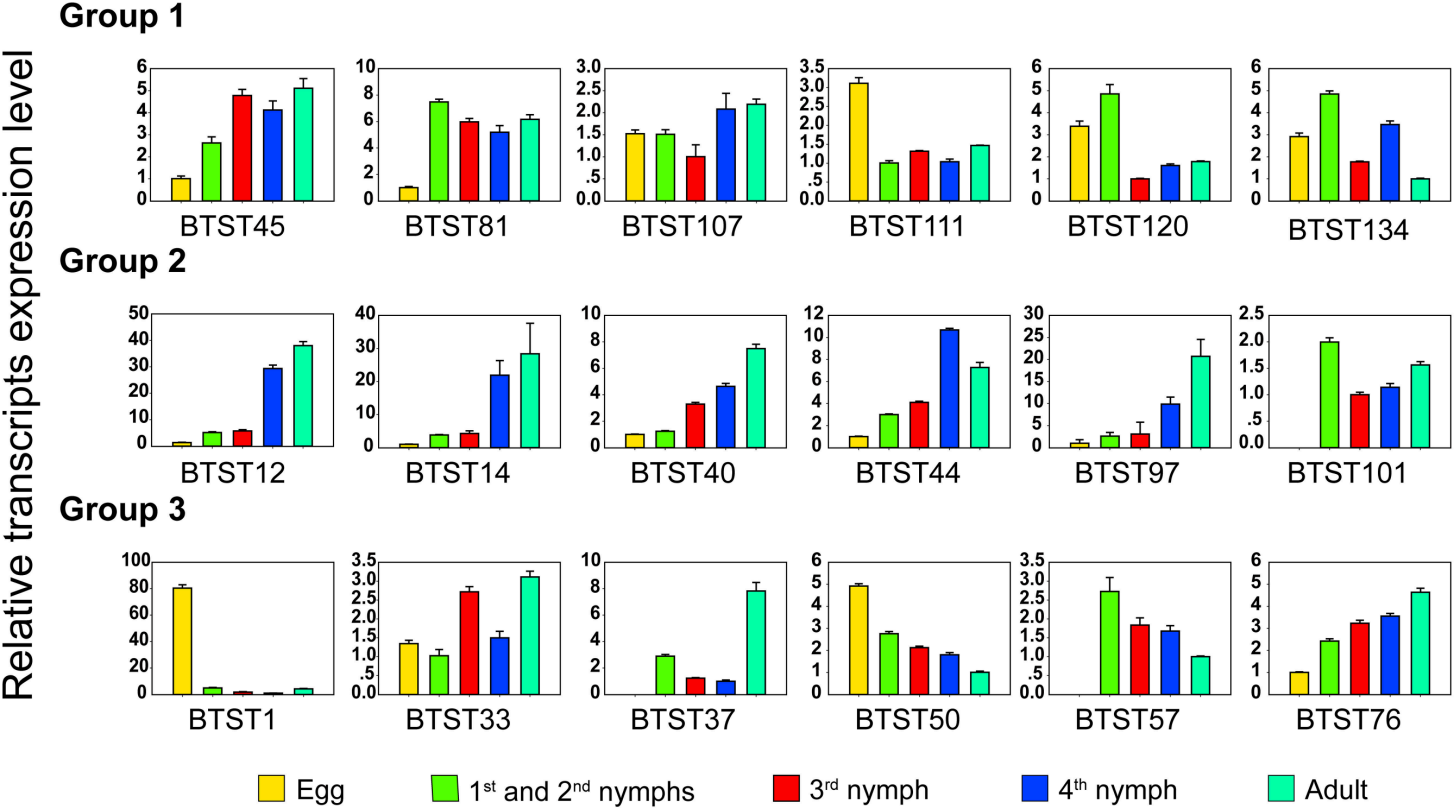
**Expression profiling of *BTSTs* across different developmental stages based on RT-qPCR**. E, eggs; N1-2, 1st and 2nd instar nymphs; N3, 3rd instar nymphs; N4, 4th instar nymphs; A, female and male adults.

The expression patterns of *BTSTs* were investigated using RNA-seq datasets obtained from different developmental stages of *B. tabaci*. The egg stage clustered separately from the other stages, whereas the 3rd and the 4th instars clustered together with female and male adults (Figure 2, Table S10). Transcript expression of most *BTSTs* was the lowest in eggs and the highest in adults, and only a few *BTSTs* exhibited a stage-specific expression pattern. Based on the expression values, the *BTSTs* could be divided into three groups. Group 1 contained 41 *BTSTs*, which were expressed in all developmental stages; among these, 15 *BTSTs* were highly expressed. Group 2 contained 33 *BTSTs*, which had low levels of expression and in some cases were not expressed in one or two stages. Most *BTSTs* in group 2 were not expressed in eggs, while the expression of *BTST40, BTST44, BTST80*, and *BTST5* was low in eggs but was high in other stages. Group 3 contained 63 *BTSTs*, which were expressed in fewer than three stages, i.e., group 3 contained stage-specific *BTSTs*. For example, a male-specific gene *BTST33* and a female-specific gene *BTST76* were identified, although they were weakly expressed; the expression level of *BTST124* was higher in adults than in eggs (fold-change: female vs. egg = 41, male vs. egg = 36); and expression levels of *BTST1* and *BTST68* were the highest in eggs and nymphs (1st and 2nd), respectively. To test and verify the RNA-seq-based expression results, expression patterns of 15 *BTSTs* across different stages were measured by RT-qPCR, and the results were consistent with the RNA-seq results (Figure 3).

**Figure 4:**
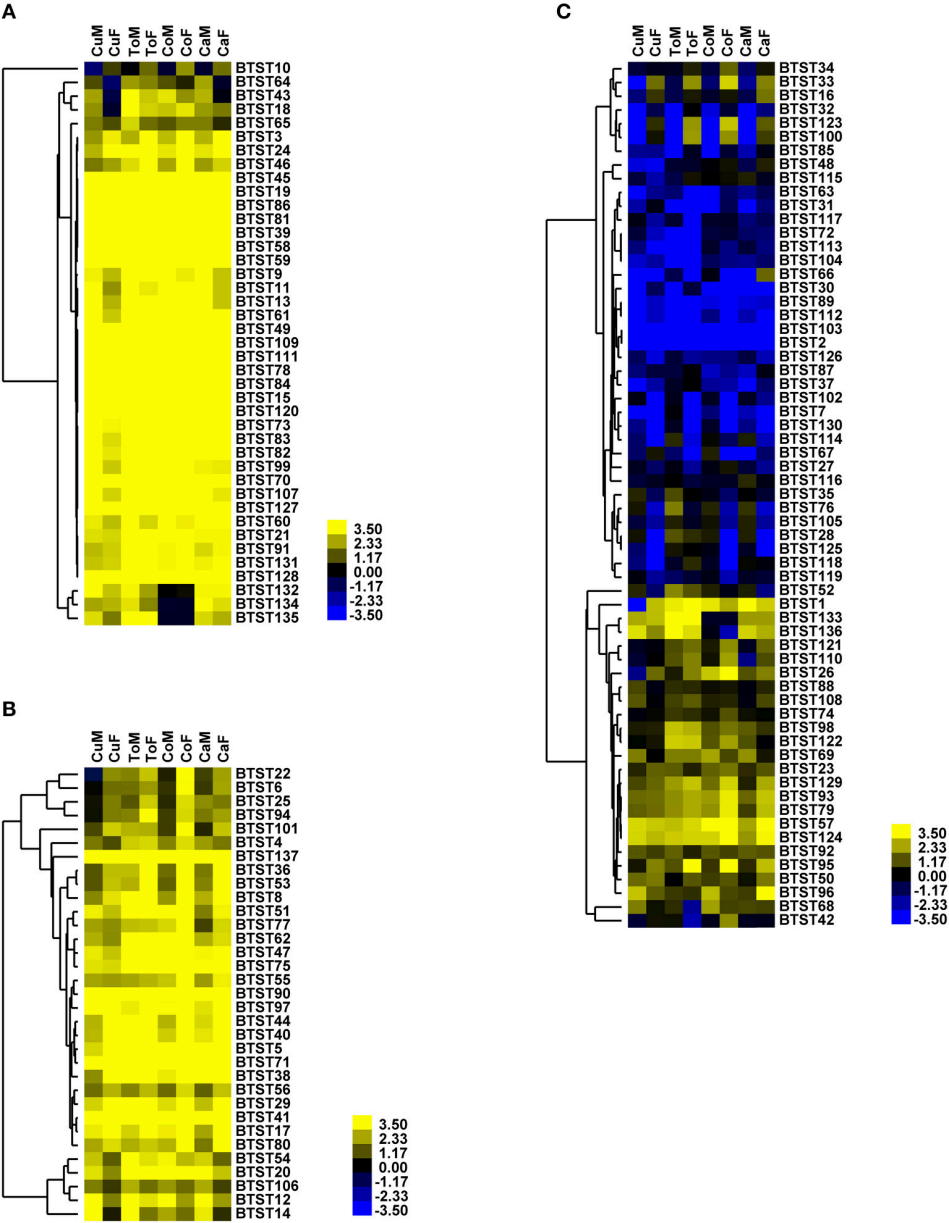
**Gene expression of *BTSTs* in male and female adults that fed on different host plants**. CaF, females reared on cabbage; CaM, males reared on cabbage; CuF, females reared on cucumber; CuM, males reared on cucumber; CoF, females reared on cotton; CoM, males reared on cotton. ToF, females reared on tomato; ToM, males reared on tomato. **(A)** Group 1; **(B)** Group 2; **(C)** Group 3.

RNA-seq data were also used to investigate how the expression profiles of *BTSTs* were affected by host plants. The results showed that expression was more affected by *B. tabaci* sex rather than by host plants (Figure 4, Table S11). Although the expression patterns of all *BTSTs* were not the same on all hosts, no host-specific *BTST* was identified.

### Phylogenetic analysis of *STs*

**Figure 5:**
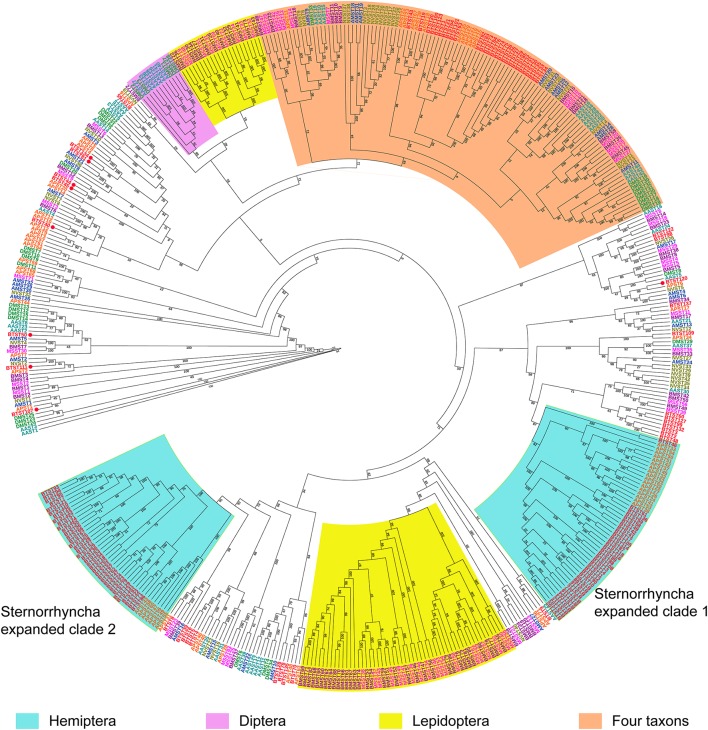
**A maximum-likelihood tree of *STs* in *B. tabaci* and seven other insects**. The analysis was constructed with Raxml V8.2.4 and the LG+F+I+G model. Bootstrap values expressed as percentages of 1000 replications are shown at branch points. Sternorrhyncha (AP, *A. pisum*, orange; BT, *B. tabaci*, red), Hymenoptera (AM, *A. mellifera*, blue; NV, *N. vitripennis*, brown), Diptera (AA, *A. aegypti*, cyan; DM, *D. melanogaster*, green), Lepidoptera (BM, *B. mori*, dark purple; MS, *M. sexta*, bright purple).

Sugar transporters from the other 11 insect species were also identified. The number of *STs* varied among these insects (Table 1). Insects in the Hymenoptera and Diptera encoded fewer *STs* than insects in the Sternorrhyncha and Lepidoptera. The number of *STs* was greater in *B. tabaci* than in the other 11 insects (Table 1). Phylogenetic analysis showed that *STs* from the 12 insects formed several conspicuous, expanded clades (Figure 5). In addition to shared expanded clades that included insects from multiple orders, there were two Sternorrhyncha, two Lepidoptera, and one Diptera expanded clades. Number of *STs* in each of the expanded clades in the Lepidoptera and Sternorrhyncha expanded clades was more than it in the Diptera expanded clade. Interestingly, *B. tabaci* contributed more *STs* to the Sternorrhyncha expanded clades than *A. pisum*.

**Table 1:** **Number of *STs* in *B. tabaci* and other insect species**.

**Order**	**Species**	**Number of *STs***
Sternorrhyncha (Hemiptera)	*Bemisia tabaci*	137
	*Acyrthosiphon pisum*	71
	*Diaphorina citri*	94
Hymenoptera	*Nasonia vitripennis*	43
	*Apis mellifera*	34
Diptera	*Musca domestica*	28
	*Drosophila melanogaster*	34
	*Anopheles gambiae*	35
	*Aedes aegypti*	44
Lepidoptera	*Manduca sexta*	83
	*Plutella xylostella*	99
	*Bombyx mori*	90

*The genome and predicted protein sequences of other insect species were obtained from NCBI databases and insect genome databases. Detailed information is provided in Table S1*.

### Molecular evolution analysis of *STs*

As indicated in the previous section, phylogenetic analysis showed that *STs* from the selected insect species formed several expanded clades. Several tandem repeat genes were also identified in the expanded clades. To test whether the *BTSTs* in Sternorrhyncha expanded clades have accelerated rates of evolution, a series of molecular evolution tests were performed. For all *BTSTs* in the two Sternorrhyncha expanded clades, the significantly improved likelihood estimated for the two ratios model compared with one ratio model suggested the *BTSTs* of the expanded clades evolved, on the average, at a higher rate than the *STs* outside the expanded clades (Table 2). We then tested the hypothesis that the evolutionary rates of *BTSTs* in expanded clades were consistent with their expression levels. Differences in lnL values were not significant when two ratios model was compared with three-ratio model. However, lnL values were significantly different when the more ratios model was compared with the two ratios model. The results indicated that *BTSTs* had different evolution rates within expanded clades.

**Table 2:** **Results of the *B. tabaci* Sternorrhyncha expanded *ST* branch-based molecular evolution analyses**.

	**Clade 1**		**Clade 2**	
	**Category**	**Parameters for Branches**	**Category**	**Parameters for branches**
One ratio	0	ω_0_ = ω_1_…ω_14_ = 0.17141	0	ω_0_ = ω_1_…ω_16_ = 0.14396
	InL	–57634.628771	InL	–47518.943747
Two ratios	0	ω_0_ = 0.084107	0	ω_0_ = 0.0337427
	1	ω_1_ = ω_2_…ω_14_ = 0.18921	1	ω_1_ = ω_2_…ω_16_ = 0.157192
	InL	–57603.803961^*^	InL	–47474.088928^*^
Three ratios	0	ω_0_ = 0.0837559	2	ω_0_ = 0.033742
	1	ω_1_ = 0.234393	1	ω_1_ = 0.156906
	2	ω_2_ = ω_3_…ω_14_ = 0.17808	2	ω_2_ = ω_3_…ω_16_ = 0.156906
	InL	–57598.536150	InL	–47474.086207
More ratios	0	ω_0_ = 0.0836167	0	ω_0_ = 0.0336747
	1	ω_1_ = 0.246863	1	ω_1_ = 0.188756
	2	ω_2_ = 0.0525813	2	ω_2_ = 0.105008
	3	ω_3_ = 0.243982	3	ω_3_ = 0.164453
	4	ω_4_ = 0.287662	4	ω_4_ = 0.125235
	5	ω_5_ = 0.106381	5	ω_5_ = 0.108414
	6	ω_6_ = 0.107114	6	ω_6_ = 0.165197
	7	ω_7_ = 0.180552	7	ω_7_ = 0.116217
	8	ω_8_ = 0.244492	8	ω_8_ = 0.175559
	9	ω_9_ = 0.180377	9	ω_9_ = 0.197858
	10	ω_10_ = 0.0963282	10	ω_10_ = 0.11441
	11	ω_11_ = 0.106107	11	ω_11_ = 0.138239
	12	ω_12_ = 0.183247	12	ω_12_ = 0.286897
	13	ω_13_ = 0.215783	13	ω_13_ = 0.158052
	14	ω_14_ = 0.157359	14	ω_14_ = 0.243745
	InL	−57560.187655^*^	15	ω_15_ = 0.155815
			InL	−47459.189793^*^

*Branches assigned to different ω categories are shown in Figure S1. An asterisk indicates a statistically significant improvement in the model (P < 0.05). The following model pairs were compared with a likelihood ratio test: one ratio and two ratios, two ratios and three ratios, and two ratios and more ratios*.

To determine why accelerated rates of evolution associated with expression in the Sternorrhyncha expanded clades (Table 2), site based analyses were performed to identify the positive selected coden sites. For both the two clades, the significantly differences in the first model pairs (M0 vs. M3) indicated ω varied among all sites (Table 3). To search for the positive selected coden sites in the expanded sequences, two other model pairs were used. However, for both of the expanded clades, the two model pairs (M1a vs. M2a and M7 vs. M8) did not show significantly difference and no positive selected sites were identified (Table 3). The results indicated that the elevated ω we identified in the expanded clades (Table 2) resulted from relaxed selection constrained, but not the positive selected.

**Table 3:** **Results of the *B. tabaci* Sternorrhyncha expanded *ST* site-based molecular evolution analyses**.

**Clade**	**Nested model pair**	**Model name**	**Parameters estimated**	**lnL**
Clade 1	1	M0: One ω among sites	ω = 0.08565	−22212.672057
		M3: Variable ω among sites	*p0* = 0.11391, *p1* = 0.47220 (*p2* = 0.41388) ω0 = 0.00881, ω1 = 0.05765, ω2 = 0.15312	−21809.015449^*^
	2	M1a: Nearly neutral	*p0* = 0.95290 (*p1* = 0.04710) ω0 = 0.10976 (ω1 = 1.00000)	−22206.064429
		M2a: Postitive selection	*p0* = 0.95290, *p1* = 0.03354 (*p2* = 0.01355) ω0 = 0.10976 (ω1 = 1.00000), ω2 = 1.00000	−22206.064429
	3	M7: Beta	*p* = 1.41800, *q* = 13.68970	−21797.997485
		M8: Beta, ω > 1	*p0* = 0.99999 (*p1* = 0.00001) *p* = 1.41800, *q* = 13.68970, ω = 9.73852	−21797.999860
Clade 2	1	M0: One ω among sites	ω = 0.14018	−30918.813043
		M3: Variable ω among sites	*p0* = 0.18944, *p1* = 0.47675 (*p2* = 0.33382) ω0 = 0.02313, ω1 = 0.11269, ω2 = 0.28290	−30277.286454^*^
	2	M1a: Nearly neutral	*p0* = 0.89488 (*p1* = 0.10512) ω0 = 0.16426 (ω1 = 1.00000)	−30824.131640
		M2a: Postitive selection	*p0* = 0.89488, *p1* = 0.05342 (*p2* = 0.05171) ω0 = 0.16426 (ω1 = 1.00000), ω2 = 1.00000	−30824.131640
	3	M7: Beta	*p* = 1.23340, *q* = 6.76221	−30253.861908
		M8: Beta, ω > 1	*p0* = 0.99999 (*p1* = 0.00001) *p* = 1.23339, *q* = 6.76220, ω = 33.38611	−30253.864820

*Model pairs are grouped and numbered. Pair 1 tests for variable ω among sites. Pair 2 and 3 test for positive selection. An asterisk indicates a statistically significant improvement in the model pair (P < 0.05)*.

### Effects of oral delivery of *STs* dsRNA

**Figure 6:**
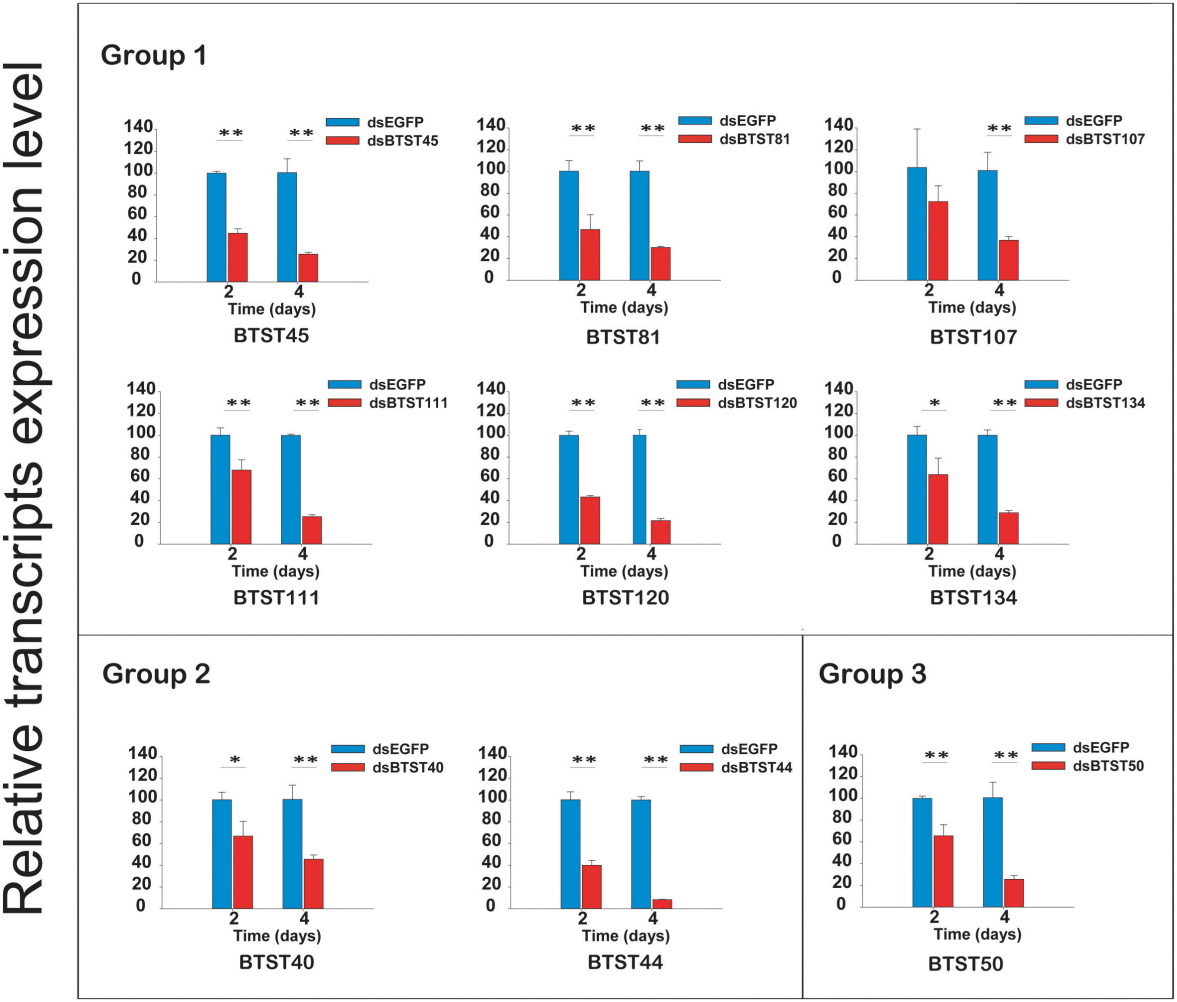
**Temporal profiles of RNAi effects**. A total of nine *BTSTs* were investigated. The gene expression levels in *B. tabaci* adults were monitored by RT-qPCR after treating by 2 and 4 days. Statistical analysis of mRNA levels was performed with student *t*-test (^*^P < 0.05; ^**^P < 0.01).

Based on our annotation, nine *BTSTs* that matched the TIGRT00879 domain with scores above 237.8 were cloned (Figure S2). To investigate the function of *BTSTs* in *B. tabaci*, dsRNAs of the nine *BTSTs* were synthesized and separately fed to *B. tabaci* adults. Mortality was recorded after the adults had fed on the dsRNAs for 2 and 4 days (Table 4). Feeding on the dsRNAs significantly suppressed the expression levels of the corresponding *BTSTs* (Figure 6). Silencing of seven of the *BTSTs* (all except *BTST120* and *BTST134*) resulted in significantly higher mortality compared to the control at day 2. However, only dsBTST40 and dsBTST44 caused significant mortality at day 4. DsBTST40 caused higher mortality than the other dsRNAs on both day 2 and 4. Mortality caused by the dsRNAs was the lowest for dsBTST134.

**Table 4:** **Effects of silencing nine *BTSTs* by oral delivery of dsRNA on the mortality of adult *B. tabaci***.

**Days**	**Mortality of adults (%) that were fed the indicated dsRNA**									
	**dsEGFP**	**dsBTST40**	**dsBTST44**	**dsBST45**	**dsBTST50**	**dsBTST81**	**dsBTST107**	**dsBTST111**	**dsBTST120**	**dsBTST134**
2	0.0 ± 0.0	15.3 ± 3.1^*^	10.7 ± 1.2^*^	10.7 ± 3.6^*^	6.7 ± 1.2^*^	5.3 ± 1.2^*^	8.7 ± 3.1^*^	6.0 ± 2.0^*^	2.0 ± 2.0	1.3 ± 2.3
4	16.7 ± 5.3	35.3 ± 6.1^*^	34.7 ± 9.4^*^	24.7 ± 7.6	18.7 ± 3.1	10.7 ± 6.1	21.3 ± 8.1	18.7 ± 4.2	15.0 ± 3.0	9.3 ± 8.08

*Values are means ± standard deviation. An asterisk indicates a statistically significant increase in mortality relative to the control (dsEGFP) (P < 0.05)*.

## Discussion

In our study, a total of 137 *BTSTs* were identified from *B. tabaci* MEAM1. Because of genome complexity, some redundant *BTSTs* exist. However, whether those duplicated *BTSTs* exist on different scaffolds is still uncertain. Among these identified *BTSTs*, for all but three of *BTSTs* had transcriptome coverage. Complete coding sequences of nine *BTSTs* that contained the TIGR00879 motif and partial coding sequences of 24 randomly selected *BTSTs* were also cloned successfully (Figures S2, S3 and Table S6). These results suggested that the quality of the *BTSTs* annotation was good. The 137 *BTSTs* were located on 86 scafflolds and a total of 26 gene clusters were also identified, which indicated duplication could be accounted for the expansion of *BTSTs*. We also anticipate that the several remaining *BTST* expansion members are likely located on neighboring scaffolds, which will be resolved by the improvement of *B. tabaci* genome in the future.

Previous studies have shown that *A. pisum* and *B. mori* contained a large *ST* family (Price et al., 2010; Govindaraj et al., 2016). Our study showed that *B. tabaci* encodes much more *STs* than *A. pisum, B. mori*, and nine other insects. Our phylogenetic analysis showed that the evolutionary pattern of the *ST* family differed among selected lepidopterans and sternorrhynchans, which was consistent with the phylogenetic relationships of the insects. This phenomenon also consisted with the previous finding that the pattern of insect's digestion correlated well with its phylogenetic position (Terra, 1990). As noted earlier, *STs* mediate the transport of sugar into or out of cells. Furthermore, the sugar content of food, feeding behavior, and digestion differ among sternorrhynchans (like *B. tabaci* and *A. pisum*) and lepidopterans (like *B. mori* and *M. sexta*) (Terra, 1990; Ashford et al., 2000; Douglas, 2006). A recent study also revealed that the divergent evolutionary pattern of *STs* is associated with the difference in sugar accumulation between grasses and eudicots (Wang et al., 2016). Thus, the different evolutionary patterns of *STs* in different insects may result from differences in food quantity, suck-feeding behavior, and digestion. The Diptera expanded clade grouped with one of the Lepidoptera expanded clades, perhaps because lepidopterans are phylogenetically close to dipterans (Misof et al., 2014). In addition, phylogenetic analysis showed that *B. tabaci* had 68 *STs* more than *A. pisum*, while it had 63 *STs* more than *A. pisum* in the expanded clades, which suggesting that most of the difference in number of *STs* in *B. tabaci* and *A. pisum* was accounted for by *STs* in expanded clades.

Although predicting the function of *STs* based on the similarity of amino acid sequences is difficult (Kikuta et al., 2012), the expression level of *STs* provides clue about their functional role. Using gene expression data, previous studies have revealed the roles of *STs* in sugar accumulation in plants (Reuscher et al., 2014; Li et al., 2015). In aphids and planthoppers, several gut-specific *STs* were identified based on expression data (Kikuta et al., 2010; Price and Gatehouse, 2014). For *B. tabaci, BTSTs* were divided into three groups based on their expression levels. The expressional pattern of group 1 indicates that those *BTSTs* play vital roles in *B. tabaci* development, because *B. tabaci* does not feed in the egg stage. Some of these *BTSTs* in group 2 may be involved in feeding or post-egg development. *BTSTs* in group 3 were expressed weakly across all stages or were only expressed in one or two stages. Interestingly, among the 17 *BTSTs* that contained fewer than 12 transmembrane domains, 12 of them were assigned to group 3. The 12-transmembrane structure has been demonstrated to be important for *ST* function (Pazdernik et al., 1997; Yan, 2015). Loss of transmembrane domains may lead to reduced or no expression of *STs*. The lower expression pattern and fewer transmembrane domains of 12 *BTSTs* in group 3 indicate that they have probably lost their functions. Expression profiles showed that nearly all of the *BTSTs* were expressed at a higher level in nymphs than adults. This is probably because *B. tabaci* nymphs develop rapidly and store nutrition for molting. Finally, the transcriptome data from our recent study (Xie et al., 2014) in which female and male adults of *B. tabaci* which were collected from different host plants showed that the expression patterns of *BTSTs* were clustered based on sex rather than on host plant. The differences in the expression of *BTSTs* between females and males, may be caused by differences in the requirements for sugars by females and males. Because *B. tabaci* has a wide range of host plants that likely contain different concentrations of sugar in their phloem, these results indicate that *B. tabaci* has probably evolved a highly effective sugar transport system.

Previous study also showed the expression profiles between or among duplicated genes were different (Zhang, 2003) and amino acid transporters and the slimfast amino acid transporters exhibited male-biased expression in aphid (Duncan et al., 2011; Price et al., 2011). In our study, the expression between or among several duplicated genes, such as *BTST41* and *BTST42, BTST10* to *BTST14*, and *BTST67* to *BTST69* in expanded clade 1 and *BTST23* and *BTST24, BTST93* to *BTST95* in clade 2 were different. Those expressional differences of the duplicated genes were perhaps resulted from the evolution of their cis-regulatory regions and could lead to the functional diversity of the duplicated genes (Nowak et al., 1997; Wagner, 2000). To investigate whether *BTSTs* in Sternorrhyncha expanded clades had accelerated evolution rates associated with its expression profiles and to determine why, we performed a series of molecular evolution studies. Results of branch-based tests showed that the accelerated evolution of *BTSTs* in the expanded clades related with their expression profiles. Subsequent site-based tests suggested that the accelerated rate of evolution probably resulted from relaxed selection constaint. Sequence number, sequence length, and sequence divergence will influence the finding of positive selection sites (Anisimova et al., 2001). Because the sequences varied and each analysis involved massive numbers of sequences, perhaps these factors may explain the failure to identify positive selection sites. However, previous studies also detected relaxed selection constaint in amino acid transporters from slimfast-expanded clades of *A. pisum* (Price et al., 2011). Relaxed selection constraint could maintain duplicated genes by complementary degenerative mutations. To fulfill the complete function of the parent gene, each of the duplicated genes could not be removed (Force et al., 1999). Relaxed selection constraint could also cause the functional diversity of duplicate genes by fixed mutation in one daughter gene. The fixed mutation could lead to subfunctionalization of the daughter gene when the environment or the genetic group changed (Zhang, 2003). In addition, relaxed selection constraint could lead to new form of biased expression (Hunt et al., 2011). So, the accelerated rate of evolution probably resulted from relaxed selection constaint the relaxed selection constraint. For expanded genes, the different expression profiles, and the relaxed selection constraint indicated that those duplicated *BTSTs* may have different functions. Apart from transporting sugars as part of normal cell metabolism, *STs* also participate in other biological processes in insects. Under desiccation or elevated temperatures, the silencing of a trehalose transporter reduced the survival of *A. gambiae* (Liu et al., 2013). Sugar transporters were also reported to play a role in interactions between insects and pathogens (Dussaubat et al., 2012). As an invasive and polyphagous pest, *B. tabaci* is confronted with various biotic and abiotic stresses; therefore, the expanded *BTSTs* may help *B. tabaci* endure these stresses.

Pervious study reported that candidate gut Ap*STs* which contained TIGR00879 motif transported glucose in *A. pisum* (Price et al., 2010). Later, another studies showed silencing candidate gut Mp*ST* which contained TIGR00879 motif increased hemolymph osmotic pressure in *M. persicae* (Tzin et al., 2015). As osmoregulation mechanism is vital important for *B. tabaci* survive, silencing the genes in osmoregulation mechanism could be used for controlling *B. tabaci*. In this study, a total of nine *BTSTs* which contained TIGR00879 motif were chosen to investigate the possibility of using *STs* as RNAi target for whitefly control. All the genes were expressed across the whole developmental stages; with *BTST40* and *BTST44* in group 2 were low expressed at the egg stage. The results showed that silencing seven of these *BTSTs* could significantly reduce the survival of *B. tabaci*. Compared with silencing *BTST40* and *BTST44*, silencing five of these *BTSTs* including *BTST45, BTST50, BTST81, BTST107*, and *BTST111* can only significantly increases the mortality rate of *B. tabaci* at day 2. One possible explanation for the differences in mortality rate was perhaps these five *BTSTs* were not involved in regulating osmotic pressure. Another possibility may result from influencing the normal metabolism upon silencing at 2 days and then the functions of the five *BTSTs* were accomplished by other *BTSTs*. Silencing the *BTST40* and *BTST44* significantly increasing the mortality rate of *B. tabaci* at day 2 and 4. Interestingly, based on their expression, *BTST45, BTST81, BTST107*, and *BTST111* were divided into group 1, *BTST50* was divided into group 3, while *BTST40* and *BTST44* were listed in group 2. It seems that the silencing effect were associated with the expression group (gene expression values) (Figure 2). As *B. tabaci* did not feed at egg stage and almost all genes in group 2 did not expressed at egg stages, genes in group 2 were assumed to involve in feeding. The expression profiles and silencing effects of *BTST40* and *BTST44* in group 2 suggested that these two genes may be used as potential targets for controlling *B. tabaci*. Other studies showed that silencing *N. lugens* or *A. pisum* sugar transporters were harmful to insects, such as reduced the survival of *N. lugens* nymphs (Zha et al., 2011; Ge et al., 2015) and reduced the fecundity of *A. pisum* (Tzin et al., 2015). The current and previous results indicate that the silencing of *STs* could be used to control of phloem-feeding insects. To find the suitable *STs* for whitefly biocontrol, much more work, such as biochemistry analysis, function analysis and other detailed expressional analysis, still needs to be performed.

In this study, a genomic-wide identification and expression analysis of *BTSTs* was performed based on genome and transcriptome datasets. Comparative analysis of *BTSTs* and *STs* in other insects showed that *B. tabaci* encodes an expanded *ST* family. Furthermore, molecular analysis suggested that the expanded clades of *BTSTs* have an accelerate rate of evolution, and the accelerate rate of evolution is consistent with the *BTSTs* expression profiles. Additionally, *BTST40* and *BTST44* were identified as potential target genes for *B. tabaci* control. In short, our study presents a global view of *BTSTs* and provides a starting point for further research on the functions of *STs* in *B. tabaci*.

## Author contributions

YZ and ZY conceived and designed the experiment; ZY and JX performed the experiment; ZY, CG, and HZ analyzed the data; WX, ZG, SW, XY, FY, and QW contributed reagents/materials/analysis tools; ZY drafted the paper; YZ and HP edited the manuscript; YZ supervised the entire project.

## Funding

This work was funded by the National Natural Science Foundation of China (31420103919, 31572014), China Agriculture Research System (CARS-26-10), Beijing Training Project for the Leading Talents in S & T (LJRC201412), and the Beijing Key Laboratory for Pest Control and Sustainable Cultivation of Vegetables.

### Conflict of interest statement

The authors declare that the research was conducted in the absence of any commercial or financial relationships that could be construed as a potential conflict of interest.

## Abbreviations

ST: sugar transporter
BTST: *B. tabaci* sugar transporter
*mtCOI*: mitochondria cytochrome oxidase one
FPKM: fragments per kilobase of exon model per million fragments mapped
RNAi: RNA interference
dsRNA: double-stranded RNA
MFS: major facilitator superfamily.
