# Supplementary material for: Genome-Wide Characterization and Expression Profiling of Sugar Transporter Family in the Whitefly, Bemisia tabaci (Gennadius) (Hemiptera: Aleyrodidae)
Source: Front Physiol. 2017 May 23;8:322. doi: 10.3389/fphys.2017.00322 (PMC5440588; doi:10.3389/fphys.2017.00322)
Supplement: Supplementary file 1 [file Table1.DOCX]

**Table S1. Sources of other selected insects’ genome**

| **Organism** | **version** | **Database** |
| --- | --- | --- |
| *Acyrthosiphon pisum* | aphidbase_2.1b | Aphidbase |
| *Diaphorina_citri* | version 1.1 | NCBI |
| *Apis mellifera* | Amel_4.5 | NCBI |
| *Nasonia vitripennis* | Nvit_2.1 | NCBI |
| *Musca domestica* | MdomA1.1 | Vectorbase |
| *Drosophila melanogaster* | dmel6.05 | Flybase |
| *Anopheles gambiae* | AgamP4.3 | Vectorbase |
| *Aedes aegypti* | AaegL3.3 | Vectorbase |
| *Manduca sexta* | OGS2 | Agripestbase^a^ |
| *Plutella xylostella* | DBM_FJ_V1.1 | NCBI |
| *Bombyx mori* | ASM15162v1 | NCBI |

^a^The data was stored in i5K database now.
