# Supplementary material for: Genome-Wide Characterization and Expression Profiling of Sugar Transporter Family in the Whitefly, Bemisia tabaci (Gennadius) (Hemiptera: Aleyrodidae)
Source: Front Physiol. 2017 May 23;8:322. doi: 10.3389/fphys.2017.00322 (PMC5440588; doi:10.3389/fphys.2017.00322)
Supplement: Supplementary file 2 [file Table2.DOCX]

**Table S2. Tested models for branch-based molecular evolution of *B. tabaci* Sternorrhyncha *STs*.** The model names indicate the number of ω categories assumed (e.g., one ratio assumes one ω for all branches).

|  | ω Category | |
| --- | --- | --- |
|  | Clade 1 | Clade 2 |
| One ratio | All ω were equal. | All ω were equal. |
| Two ratios | ω_0_≠ω_1_…ω_14_; ω_1_=ω_2_…ω_14_ | ω_0_≠ω_1_…ω_16_; ω_1_=ω_2_…ω_16_ |
| Three ratios | ω_0_≠ω_1_≠ω_2_…ω_14_; ω_2_=ω_3_…ω_14_ | ω_0_≠ω_1_≠ω_2_…ω_16_; ω_2_=ω_3_…ω_16_ |
| More ratios | All ω were not equal. | All ω were not equal. |
