# Supplementary material for: Genome-Wide Characterization and Expression Profiling of Sugar Transporter Family in the Whitefly, Bemisia tabaci (Gennadius) (Hemiptera: Aleyrodidae)
Source: Front Physiol. 2017 May 23;8:322. doi: 10.3389/fphys.2017.00322 (PMC5440588; doi:10.3389/fphys.2017.00322)
Supplement: Supplementary file 3 [file Table3.DOCX]

**Table S3. Primers for cloning of the nine *BTSTs* full-length coding sequences**

| **Genes** | **NCBI accession** | **Primer** | **Primer sequences** |
| --- | --- | --- | --- |
| *BTST40* | KY350166 | fwd | GGCGTATAGACGGACTTCCTGC |
|  |  | rev | TCACTCGTGTCGTTCGGGTC |
| *BTST44* | KY350167 | fwd | TGCCTGCTGGATTACGTTTC |
|  |  | rev | ATTTCGAGGTGCCAGTTGTG |
| *BTST45* | KY350168 | fwd | CGAGTGTTATTTTGGCAGTGG |
|  |  | rev | GACCGGGGGATAGTGTAAGC |
| *BTST50* | KY350169 | fwd | CAAGAACTCAATCTGGAAGAATAAG |
|  |  | rev | GCACCGACACCATAACTGAAG |
| *BTST81* | KY350170 | fwd | CAGGTCGAAAATATCAGGCACG |
|  |  | rev | TCTTCACAGGCGATGGCAAC |
| *BTST107* | KY350171 | fwd | ATCAACCTCAGGCTCATTCTCG |
|  |  | rev | CAGCCAGAGGGATGGAGTTG |
| *BTST111* | KY350172 | fwd | GTTACCCAGTGGCAGCATC |
|  |  | rev | GCAGTCCTGAGTACGGTTTAT |
| *BTST120* | KY350173 | fwd | GTTCCGTATCGCCGTGTCG |
|  |  | rev | TAACACCCCCGTTCGCAGC |
| *BTST134* | KY350174 | fwd | TCTTCGCCTACTTACCCAGC |
|  |  | rev | GCTACTCAGCCCGCAATAC |
