# Supplementary material for: Genome-Wide Characterization and Expression Profiling of Sugar Transporter Family in the Whitefly, Bemisia tabaci (Gennadius) (Hemiptera: Aleyrodidae)
Source: Front Physiol. 2017 May 23;8:322. doi: 10.3389/fphys.2017.00322 (PMC5440588; doi:10.3389/fphys.2017.00322)
Supplement: Supplementary file 4 [file Table4.DOCX]

**Table S4. RT-qPCR primers for selected *B. tabaci* *STs* and reference gene**

| **Genes** | **Primer** | **Primer sequences** | | **Amplication (bp)** | **PCR Efficiency (%)** |
| --- | --- | --- | --- | --- | --- |
| *BTST1* | fwd | CGTCGAGTCGCCGTACTACCA | | 156 | 103.19 |
|  | rev | GCTCTTCTGACCGCTCATATCCTC | |  |  |
| *BTST12* | fwd | GGCTCAGAGGTTGGGTTGGC | | 96 | 90.31 |
|  | rev | CCGACGCTTTGGCTTCTTTGG | |  |  |
| *BTST14* | fwd | GTGGCTCTTCTGGCGAACATTC | | 116 | 97.87 |
|  | rev | GCGAGTCCCTGGCTAAGTCC | |  |  |
| *BTST33* | fwd | GTTTGCGGGGTTCCTGGTTTTC | 149 | | 92.66 |
|  | rev | GAGAGTAGCGGAGATAGACGACAC |  |  |  |
| *BTST37* | fwd | CCAGATTCGCCAGTTCACTTGAC | 161 | | 97.27 |
|  | rev | CAGCCCACGGTCGGAAGC |  |  |  |
| *BTST40* | fwd | TGCGAGAGGAAGGGAAGAAACTAC | 106 | | 103 |
|  | rev | GGCGAGGCTGACGAAGACC |  |  |  |
| *BTST44* | fwd | TGGACGAAAGCAAAGCAAAGACC | 96 | | 93.41 |
|  | rev | ATGACCACGAGAGCATCTGACC |  |  |  |
| *BTST45* | fwd | CACGACAGAGGACTCCACGAC | 112 | | 92.37 |
|  | rev | TCCTCCTCGCCCTCTACTTCTG |  |  |  |
| *BTST50* | fwd | GATGTAATCCGAGCCTGGTGTAATG | 148 | | 95.72 |
|  | rev | GCCAACCGCCAATGAAACTTCC |  |  |  |
| *BTST57* | fwd | TGTGTGCTTCTCGGGGTGTTC | 114 | | 90.1 |
|  | rev | AGTCCTTCGCTCGCCATTCC |  |  |  |
| *BTST76* | fwd | CAGGAGGCGTGGGTCATCAAC | 121 | | 108.26 |
|  | rev | ATGGCGAAGACGGTGAAGAGG |  |  |  |
| *BTST81* | fwd | GTTCGGCTCCTACTCCACCTTC | 146 | | 97.4 |
|  | rev | CGCTCTGGCTCTCGTTCTCG |  |  |  |
| *BTST97* | fwd | GGCACCCGCTCATCTTTATCTTG | 145 | | 93.01 |
|  | rev | CGCTGGACGCATTAGGCTTAGG |  |  |  |
| *BTST101* | fwd | TAGACCGCAGGCAGGAGAGG | 136 | | 98.22 |
|  | rev | GGAGAGGGTGATGACGATGATGG |  |  |  |
| *QBTST107* | fwd | ACTCTCCGACCTCCCTTAATCATC | 119 | | 99.21 |
|  | rev | TCCTCCGAAAGACCAGAACTTACG |  |  |  |
| *QBTST111* | fwd | TGTTGACACTGTTCTCTTGACCATC | 169 | | 94.57 |
|  | rev | CGGGCGGGCAGACTGATTG |  |  |  |
| *QBTST120* | fwd | ACCTCACCAGCGTTGCCTTC | 133 | | 109.3 |
|  | rev | GCGGTCCTCCTGCCATTCC |  |  |  |
| *QBTST134* | fwd | TGATTGACCGCTATGGACGAAGG | 91 | | 104.08 |
|  | rev | AGGTGAGGCTTTGAGGCTATGAG |  |  |  |
| *EF-1α* | fwd | TAGCCTTGTGCCAATTTCCG | 110 | | 103.92 |
|  | rev | CCTTCAGCATTACCGTCC |  |  |  |
