# Supplementary material for: Genome-Wide Characterization and Expression Profiling of Sugar Transporter Family in the Whitefly, Bemisia tabaci (Gennadius) (Hemiptera: Aleyrodidae)
Source: Front Physiol. 2017 May 23;8:322. doi: 10.3389/fphys.2017.00322 (PMC5440588; doi:10.3389/fphys.2017.00322)
Supplement: Supplementary file 5 [file Table5.DOCX]

**Table S5. PCR primers for nine *BTST*s dsRNA synthesis**

| **Genes** | **Primer** | **Primer sequences** | **Amplication (bp)** |
| --- | --- | --- | --- |
| *BTST40* | fwd | TAATACGACTCACTATAGGGAGAGAAAGTTGCGTGGCTTCTGTAC | 360 |
|  | rev | TAATACGACTCACTATAGGGAGAATCGATCTCCTGCTTGATGTTGT |  |
| *BTST44* | fwd | TAATACGACTCACTATAGGGAGATCGAGGCACAACCCGTGAG | 437 |
|  | rev | TAATACGACTCACTATAGGGAGACAACAACCAGGACGCCATTT |  |
| *BTST45* | fwd | TAATACGACTCACTATAGGGAGACTGGCGACGGGAATAGTGT | 413 |
|  | rev | TAATACGACTCACTATAGGGAGAGATACCGATGACGATGAAGAGTT |  |
| *BTST50* | fwd | TAATACGACTCACTATAGGGAGACCGTTGCTCAAGTTCTCCG | 445 |
|  | rev | TAATACGACTCACTATAGGGAGACATTGCGATAGGACGAGGC |  |
| *BTST81* | fwd | TAATACGACTCACTATAGGGAGAAAATGTCCCTACAGTGGTTCCG | 469 |
|  | rev | TAATACGACTCACTATAGGGAGAGATGTTGAAGCCGCAGACG |  |
| *BTST107* | fwd | TAATACGACTCACTATAGGGAGATACCATCCTTGCGTCTATGC | 382 |
|  | rev | TAATACGACTCACTATAGGGAGATAGGGACTAATGATGTATTCAAACC |  |
| *BTST111* | fwd | TAATACGACTCACTATAGGGAGATGAGGCAGCCCAATACAGT | 433 |
|  | rev | TAATACGACTCACTATAGGGAGACAAAGAGGACAAACAAACCC |  |
| *BTST120* | fwd | TAATACGACTCACTATAGGGAGAACTATCATTTCTACTGCTGTGCCTTTC | 435 |
|  | rev | TAATACGACTCACTATAGGGAGACGTTGGTCTTCTCGATTTCGTC |  |
| *BTST134* | fwd | TAATACGACTCACTATAGGGAGATATGTCTTGGACGCTTTATT | 487 |
|  | rev | TAATACGACTCACTATAGGGAGAAACCGCATTGACACCTGAA |  |
| *EGFP* | fwd | TAATACGACTCACTATAGGGAGACCACAAGTTCAGCGTGTCCG | 469 |
|  | rev | TAATACGACTCACTATAGGGAGAAAGTTCSCCTTGATGCCGTTC |  |
