# Supplementary material for: Genome-Wide Characterization and Expression Profiling of Sugar Transporter Family in the Whitefly, Bemisia tabaci (Gennadius) (Hemiptera: Aleyrodidae)
Source: Front Physiol. 2017 May 23;8:322. doi: 10.3389/fphys.2017.00322 (PMC5440588; doi:10.3389/fphys.2017.00322)
Supplement: Supplementary file 6 [file Table6.DOCX]

**Table S6. Primers for sub-cloning of 24 *BTSTs***

| **Genes** | **Primer** | **Primer sequences** | **Amplication (bp)** |
| --- | --- | --- | --- |
| *BTST9* | fwd | ACTCAGACCAGGCACAGGG | 388 |
|  | rev | ACTCAGACCAGGCACAGGG |  |
| *BTST11* | fwd | ACACCAGGCATCACGTTGAA | 388 |
|  | rev | GAACAGTAAGCACCGATACCAAT |  |
| *BTST21* | fwd | CACGGAGTCGCCCTTCTACC | 395 |
|  | rev | GAGATGACGAGCAGCGGTTT |  |
| *BTST60* | fwd | ACTCGACCTCCAATCGTTC | 354 |
|  | rev | AATCGCTAGACCTGCCACT |  |
| *BTST64* | fwd | CCGACAAAGATATTAGAGGTGC | 375 |
|  | rev | GCGATGACTATGATCGAGGC |  |
| *BTST86* | fwd | GGACCATCTTCCGACACTTC | 371 |
|  | rev | TAAACGCCAACAAACCCAC |  |
| *BTST127* | fwd | TTAGGAGCTGGATTTATGGA | 368 |
|  | rev | ATCATCGCTCTTGTATTTGG |  |
| *BTST132* | fwd | ACCCTCGGAAACTTCGTCG | 361 |
|  | rev | GCATTGCCATTGGTCTTCAT |  |
| *BTST20* | fwd | GAACTCGTGGCTGCCTTCTACT | 365 |
|  | rev | CTGGATCTCGCTCAGGGTCTT |  |
| *BTST29* | fwd | CTCATAGACAAGGCAGGTCGTAA | 362 |
|  | rev | AAACCCAACAGCCGAGAAAA |  |
| *BTST51* | fwd | GGCTCACCAACGGAATAAA | 361 |
|  | rev | TGAGGAGGATGAGGAAAGG |  |
| *BTST62* | fwd | AAATGAAGATGAACCTCCTACTGG | 384 |
|  | rev | CACCGAGGCTGGGAAACTA |  |
| *BTST71* | fwd | GACAGTCTTCCACCATGTTACCG | 356 |
|  | rev | AGGAGGAGACCGTCGCAGT |  |
| *BTST77* | fwd | CGCTGTTGGTTTGGAAGTG | 397 |
|  | rev | TGAGGAACGGTTTATAGATGGTC |  |
| *BTST80* | fwd | TTTTACAATGCTTGCTCTGGGTC | 388 |
|  | rev | AATCGCACTTGGGCTGGTT |  |
| *BTST106* | fwd | AGAAACCCACCGTGCTTAA | 370 |
|  | rev | CAAGGCAAGGAGGATAAGG |  |
| *BTST31* | fwd | CTCGCCACTTCACCTAACCC | 367 |
|  | rev | CAATGGACGCTCCTCCTCTT |  |
| *BTST52* | fwd | CTTCCTCCAGACAGGCACCG | 361 |
|  | rev | GCTCCCTGGTCATCAGCCAC |  |
| *BTST63* | fwd | TACTTCTTCCTCAGCCTCGTCT | 368 |
|  | rev | AAACATCACATCCATTCCCAAC |  |
| *BTST72* | fwd | TGGAGAACTGTTGCCTTGA | 375 |
|  | rev | ACGGTCTAATCGCACTGAA |  |
| *BTST74* | fwd | GAATTGGTCGGAAGAGGCT | 396 |
|  | rev | TTCCCGCTCGTTTCTATGA |  |
| *BTST85* | fwd | CCCTTCACGTCAGACAACTGC | 374 |
|  | rev | CGCGAACACGGAAACACTC |  |
| *BTST102* | fwd | CAGTCTGTTATCTGGCTATGTC | 396 |
|  | rev | CAACCGATTTCTTACCACTAA |  |
| *BTST121* | fwd | TTGCTGATCGGGCCAATAAC | 346 |
|  | rev | AGGAGCTGGACGACGAAGTAGA |  |
