# Supplementary material for: Genome-Wide Characterization and Expression Profiling of Sugar Transporter Family in the Whitefly, Bemisia tabaci (Gennadius) (Hemiptera: Aleyrodidae)
Source: Front Physiol. 2017 May 23;8:322. doi: 10.3389/fphys.2017.00322 (PMC5440588; doi:10.3389/fphys.2017.00322)
Supplement: Supplementary file 7 [file Table7.DOCX]

**Table S7. Summary of annotated *BTSTs***

| **Genes** | **Scaffold**  **(Orientation)** | **Start** | **End** | **Protein Length** | **Exon** | **TM^a^** | **Transcripts** | **Pfam^b^** | | **TIGRFAMS^c^** | | **Nr database^d^** | |
| --- | --- | --- | --- | --- | --- | --- | --- | --- | --- | --- | --- | --- | --- |
|  |  |  |  |  |  |  |  | **E-value** | **Sscore** | **E-value** | **Score** | **E-value** | **Blast hit** |
| *BTST1* | scaffold1 (-) | 24799 | 20477 | 458 | 4 | 12 | √ | 1.10e-61 | 202.2 | 7.70e-57 | 185.9 | 4.00e-114 | BAQ02356.1 |
| *BTST2* | scaffold2 (+) | 4693465 | 4700026 | 459 | 6 | 11 |  | 9.70e-51 | 166.1 | 6.20e-48 | 156.6 | 2.00e-77 | ETN59145.1 |
| *BTST3* | scaffold2 (+) | 4724650 | 4736129 | 540 | 7 | 12 | √ | 5.70e-52 | 170.1 | 4.00e-49 | 160.5 | e-109 | XP_321919.5 |
| *BTST4* | scaffold10 (-) | 668826 | 654079 | 590 | 8 | 12 | √ | 6.50e-53 | 173.2 | 4.80e-45 | 147 | 7.00e-32 | XP_969266.1 |
| *BTST5* | scaffold10 (-) | 692569 | 674850 | 505 | 7 | 12 | √ | 3.10e-53 | 174.3 | 3.50e-46 | 150.8 | 2.00e-66 | XP_969266.1 |
| *BTST6* | scaffold10 (-) | 750072 | 710450 | 534 | 7 | 12 | √ | 2.50e-57 | 187.8 | 6.10e-51 | 166.5 | 2.00e-74 | KDR07564.1 |
| *BTST7* | scaffold12 (-) | 818970 | 814262 | 348 | 2 | 8 | √ | 6.30e-32 | 104 | 5.90e-24 | 77.4 | 7.00e-34 | XP_014241896.1 |
| *BTST8* | scaffold13 (-) | 3562750 | 3512862 | 497 | 5 | 12 | √ | 1.10e-62 | 205.5 | 1.10e-54 | 178.8 | 0.00E+00 | XP_001944504.2 |
| *BTST9* | scaffold13 (+) | 3663266 | 3675011 | 514 | 5 | 12 | √ | 1.40e-76 | 251.2 | 2.60e-69 | 227.1 | e-141 | XP_001652873.1 |
| *BTST10* | scaffold15 (-) | 2064396 | 2052568 | 488 | 7 | 12 | √ | 8.70e-50 | 162.9 | 7.40e-41 | 133.2 | 2.00e-96 | XP_001943575.1 |
| *BTST11* | scaffold15 (-) | 2087477 | 2074877 | 526 | 8 | 12 | √ | 2.00e-48 | 158.4 | 7.00e-42 | 136.6 | e-105 | BAQ02359.1 |
| *BTST12* | scaffold15 (-) | 2099997 | 2090621 | 532 | 8 | 12 | √ | 1.80e-44 | 145.3 | 2.20e-38 | 125 | 5.00e-98 | BAQ02359.1 |
| *BTST13* | scaffold15 (-) | 2120966 | 2103048 | 517 | 8 | 12 | √ | 4.90e-47 | 153.8 | 2.00e-38 | 125.2 | e-110 | BAQ02359.1 |
| *BTST14* | scaffold15 (-) | 2139158 | 2126200 | 525 | 8 | 12 | √ | 3.80e-44 | 144.3 | 7.00e-37 | 120.1 | e-106 | BAQ02359.1 |
| *BTST15* | scaffold15 (-) | 2162002 | 2144477 | 522 | 8 | 12 | √ | 1.40e-43 | 142.4 | 1.30e-36 | 119.2 | 2.00e-96 | BAQ02359.1 |
| *BTST16* | scaffold15 (-) | 2178344 | 2168793 | 518 | 8 | 12 | √ | 1.60e-39 | 129 | 4.20e-33 | 107.6 | 2.00e-96 | XP_001943575.1 |
| *BTST17* | scaffold15 (-) | 2195734 | 2184013 | 530 | 7 | 12 | √ | 1.40e-54 | 178.7 | 2.10e-50 | 164.7 | e-110 | XP_001943575.1 |
| *BTST18* | scaffold17 (-) | 1519231 | 1503256 | 503 | 7 | 10 | √ | 5.60e-36 | 117.4 | 1.90e-28 | 92.2 | 7.00e-46 | XP_971034.1 |
| *BTST19* | scaffold19 (-) | 1085903 | 1082835 | 493 | 2 | 12 | √ | 1.40e-69 | 228.2 | 1.90e-55 | 181.3 | 3.00e-57 | KDR15737.1 |
| *BTST20* | scaffold19 (+) | 1094394 | 1101975 | 485 | 2 | 12 | √ | 6.30e-65 | 212.8 | 1.30e-63 | 208.2 | e-154 | XP_008483238.1 |
| *BTST21* | scaffold20 (+) | 64456 | 95538 | 456 | 5 | 12 | √ | 1.70e-50 | 165.3 | 4.50e-40 | 130.6 | 3.00e-59 | XP_001952640.1 |
| *BTST22* | scaffold20 (+) | 175979 | 188644 | 500 | 6 | 12 | √ | 3.10e-58 | 190.7 | 4.50e-47 | 153.7 | 1.00e-67 | KDR15737.1 |
| *BTST23* | scaffold21 (-) | 1177347 | 1166515 | 461 | 6 | 12 | √ | 5.70e-54 | 176.7 | 8.10e-41 | 133.1 | 2.00e-43 | XP_308203.4 |
| *BTST24* | scaffold21 (-) | 1194139 | 1179661 | 468 | 6 | 12 | √ | 5.90e-55 | 179.9 | 9.90e-41 | 132.8 | 1.00e-56 | EFA05603.1 |
| *BTST25* | scaffold21 (-) | 1439566 | 1421419 | 516 | 5 | 12 | √ | 8.00e-50 | 163 | 1.60e-40 | 132.1 | 6.00e-77 | KDR15737.1 |
| *BTST26* | scaffold23 (-) | 513449 | 485427 | 474 | 3 | 12 | √ | 4.70e-71 | 233 | 2.30e-62 | 204.2 | e-102 | BAQ02358.1 |
| *BTST27* | scaffold36 (+) | 140125 | 148937 | 482 | 6 | 12 | √ | 3.90e-44 | 144.3 | 1.90e-30 | 98.8 | 5.00e-52 | KDR15737.1 |
| *BTST28* | scaffold36 (+) | 151459 | 159764 | 475 | 6 | 12 | √ | 2.20e-45 | 148.4 | 5.30e-31 | 100.7 | 2.00e-61 | KDR15737.1 |
| *BTST29* | scaffold44 (+) | 581833 | 602053 | 473 | 4 | 12 | √ | 1.00e-69 | 228.6 | 2.50e-63 | 207.4 | e-119 | BAQ02358.1 |
| *BTST30* | scaffold44 (+) | 604597 | 609448 | 464 | 3 | 12 | √ | 1.50e-64 | 211.6 | 1.10e-57 | 188.7 | 5.00e-102 | XP014259104.1 |
| *BTST31* | scaffold44 (+) | 616251 | 620804 | 124 | 5 | 10 | √ | 1.30e-50 | 165.7 | 3.50e-48 | 157.4 | 6.00e-87 | BAQ02358.1 |
| *BTST32* | scaffold44 (+) | 623777 | 626927 | 456 | 3 | 11 | √ | 2.40e-57 | 187.8 | 1.30e-45 | 148.9 | 2.00e-103 | XP014259104.1 |
| *BTST33* | scaffold44 (+) | 628095 | 631997 | 475 | 4 | 12 | √ | 5.00e-65 | 213.1 | 1.60e-59 | 194.8 | e-111 | BAQ02358.1 |
| *BTST34* | scaffold44 (+) | 633363 | 639746 | 474 | 4 | 12 | √ | 1.20e-60 | 198.7 | 3.80e-53 | 173.7 | 2.00e-95 | BAQ02358.1 |
| *BTST35* | scaffold44 (+) | 647727 | 656534 | 511 | 6 | 12 | √ | 2.60e-45 | 148.2 | 4.40e-43 | 140.5 | 1.00e-91 | XP014259104.1 |
| *BTST36* | scaffold47 (+) | 231115 | 248694 | 485 | 5 | 12 | √ | 1.90e-46 | 151.9 | 1.60e-34 | 112.3 | 1.00e-78 | KDR15737.1 |
| *BTST37* | scaffold51 (+) | 1109660 | 1120950 | 466 | 2 | 12 | √ | 1.40e-71 | 234.7 | 1.10e-66 | 218.4 | 3.00e-113 | XP014259104.1 |
| *BTST38* | scaffold56 (-) | 782626 | 768383 | 472 | 4 | 12 | √ | 2.30e-64 | 210.9 | 3.10e-59 | 193.8 | e-109 | BAQ02358.1 |
| *BTST39* | scaffold65 (-) | 649843 | 628547 | 489 | 6 | 12 | √ | 1.00e-55 | 182.5 | 2.00e-48 | 158.2 | 5.00e-111 | KDR15737.1 |
| *BTST40* | scaffold65 (-) | 687913 | 671757 | 499 | 5 | 12 | √ | 6.50e-88 | 288.6 | 1.30e-84 | 277.6^e^ | e-111 | KDR15737.1 |
| *BTST41* | scaffold74 (+) | 139712 | 151067 | 527 | 7 | 12 | √ | 4.00e-54 | 177.2 | 1.70e-54 | 178.2 | e-105 | BAQ02359.1 |
| *BTST42* | scaffold74 (+) | 154640 | 160653 | 518 | 7 | 12 | √ | 9.20e-53 | 172.7 | 3.70e-52 | 170.5 | 6.00e-103 | XP001943832.1 |
| *BTST43* | scaffold108 (+) | 634572 | 652949 | 507 | 6 | 12 | √ | 5.00e-69 | 226.3 | 5.80e-64 | 209.4 | 2.00e-87 | BAQ02352.1 |
| *BTST44* | scaffold113 (+) | 585113 | 597097 | 516 | 6 | 12 | √ | 3.80e-83 | 272.9 | 5.80e-80 | 262.2^e^ | 2.00e-98 | BAI83425.1 |
| *BTST45* | scaffold113 (-) | 606980 | 600331 | 502 | 4 | 12 | √ | 1.90e-86 | 283.8 | 2.30e-81 | 266.8^e^ | e-112 | XP_005183299.1 |
| *BTST46* | scaffold117 (+) | 234136 | 287654 | 548 | 8 | 12 | √ | 1.50e-60 | 198.3 | 7.50e-58 | 189.3 | e-152 | BAQ02359.1 |
| *BTST47* | scaffold129 (+) | 777028 | 793487 | 466 | 6 | 12 | √ | 1.00e-59 | 195.7 | 2.50e-47 | 154.6 | 2.00e-63 | XP_308203.4 |
| *BTST48* | scaffold134 (-) | 630792 | 619938 | 514 | 8 | 12 | √ | 1.50e-51 | 168.7 | 1.80e-46 | 151.7 | 2.00e-62 | XP_321919.5 |
| *BTST49* | scaffold134 (-) | 649139 | 638593 | 525 | 7 | 12 | √ | 5.20e-50 | 163.7 | 1.10e-45 | 149.1 | e-100 | XP_008473869.1 |
| *BTST50* | scaffold135 (+) | 302501 | 313686 | 459 | 11 | 12 | √ | 1.50e-95 | 313.8 | 2.60e-82 | 270^e^ | 8.00e-104 | XP014254974.1 |
| *BTST51* | scaffold136 (-) | 619487 | 571221 | 502 | 6 | 12 | √ | 6.70e-52 | 169.9 | 2.20e-45 | 148.2 | 3.00e-73 | KDR15737.1 |
| *BTST52* | scaffold136 (-) | 651684 | 622602 | 488 | 6 | 12 | √ | 6.30e-44 | 143.6 | 3.00e-34 | 111.4 | 1.00e-60 | KDR15737.1 |
| *BTST53* | scaffold149 (-) | 64014 | 52739 | 429 | 6 | 9 | √ | 3.30e-37 | 121.5 | 2.90e-29 | 94.9 | 2.00e-51 | KDR15737.1 |
| *BTST54* | scaffold149 (-) | 83651 | 74196 | 476 | 4 | 12 | √ | 2.60e-47 | 154.7 | 3.40e-35 | 114.5 | 2.00e-65 | KDR15737.1 |
| *BTST55* | scaffold149 (-) | 111093 | 101632 | 488 | 6 | 12 | √ | 1.30e-49 | 162.3 | 2.00e-32 | 105.4 | 1.00e-60 | KDR15737.1 |
| *BTST56* | scaffold149 (-) | 184600 | 178560 | 483 | 4 | 12 | √ | 1.40e-45 | 149 | 3.60e-33 | 107.8 | 2.00e-65 | XP_001944504.2 |
| *`BTST57* | scaffold149 (-) | 212717 | 199205 | 514 | 6 | 12 | √ | 6.40e-54 | 176.5 | 3.90e-43 | 140.7 | 2.00e-67 | KDR15737.1 |
| *BTST58* | scaffold160 (+) | 609927 | 622397 | 540 | 7 | 12 | √ | 3.20e-48 | 157.8 | 1.10e-44 | 145.8 | 3.00e-96 | XP_008473869.1 |
| *BTST59* | scaffold160 (-) | 669010 | 654093 | 525 | 7 | 12 | √ | 1.80e-57 | 188.3 | 6.50e-53 | 173 | e-114 | XP_008473869.1 |
| *BTST60* | scaffold173 (+) | 76317 | 93802 | 556 | 10 | 12 | √ | 3.80e-51 | 167.4 | 1.70e-52 | 171.6 | e-121 | XP_001849608.1 |
| *BTST61* | scaffold200 (-) | 287393 | 242839 | 528 | 7 | 12 | √ | 2.50e-57 | 187.8 | 3.60e-46 | 150.7 | e-101 | XP_008472428.1 |
| *BTST62* | scaffold207 (-) | 594243 | 585893 | 467 | 4 | 12 | √ | 1.60e-56 | 185.1 | 2.10e-42 | 138.3 | 9.00e-61 | XP_971034.1 |
| *BTST63* | scaffold209 (+) | 606769 | 613891 | 460 | 4 | 11 | √ | 2.30e-49 | 161.5 | 2.50e-46 | 151.3 | 7.00e-59 | XP_014254567.1 |
| *BTST64* | scaffold209 (+) | 633185 | 637752 | 415 | 4 | 12 | √ | 3.40e-50 | 164.3 | 1.50e-46 | 152 | 1.00e-62 | XP_001952640.1 |
| *BTST65* | scaffold256 (+) | 71653 | 72989 | 453 | 1 | 12 | √ | 3.80e-71 | 233.3 | 3.10e-64 | 210.3 | e-121 | XP_008471383.1 |
| *BTST66* | scaffold273 (-) | 175430 | 169890 | 481 | 5 | 11 | √ | 2.00e-49 | 161.7 | 2.90e-46 | 151 | 2.00e-63 | XP008476300.1 |
| *BTST67* | scaffold273 (+) | 183608 | 206189 | 486 | 5 | 12 | √ | 3.80e-46 | 150.9 | 3.20e-33 | 108 | 1.00e-63 | XP_008476300.1 |
| *BTST68* | scaffold280 (-) | 422657 | 412047 | 535 | 8 | 12 | √ | 3.40e-50 | 164.2 | 6.50e-40 | 130.1 | e-106 | BAQ02372.1 |
| *BTST69* | scaffold280 (-) | 442989 | 426286 | 512 | 8 | 12 | √ | 9.70e-38 | 123.2 | 1.80e-32 | 105.6 | 9.00e-91 | XP_001943575.1 |
| *BTST70* | scaffold289 (+) | 453017 | 458159 | 484 | 3 | 12 | √ | 2.90e-57 | 187.6 | 3.70e-54 | 177.1 | e-152 | XP_008482330.1 |
| *BTST71* | scaffold290 (+) | 453553 | 471173 | 468 | 6 | 12 | √ | 1.10e-55 | 182.3 | 6.90e-46 | 149.8 | 7.00e-77 | KDR15737.1 |
| *BTST72* | scaffold292 (-) | 531983 | 520465 | 511 | 6 | 12 | √ | 6.10e-40 | 130.4 | 8.10e-34 | 110 | e-102 | BAQ02359.1 |
| *BTST73* | scaffold298 (-) | 138278 | 130416 | 483 | 2 | 12 | √ | 4.30e-67 | 219.9 | 4.50e-58 | 190 | 3.00e-79 | BAQ02358.1 |
| *BTST74* | scaffold326 (-) | 79887 | 76320 | 478 | 5 | 11 | √ | 3.30e-64 | 210.5 | 4.50e-56 | 183.4 | 3.00e-99 | BAQ02358.1 |
| *BTST75* | scaffold326 (-) | 85834 | 82655 | 478 | 3 | 12 | √ | 2.90e-63 | 207.4 | 1.70e-55 | 181.5 | 3.00e-99 | BAQ02358.1 |
| *BTST76* | scaffold326 (-) | 94981 | 91499 | 508 | 3 | 12 | √ | 7.50e-57 | 186.2 | 2.70e-48 | 157.7 | 6.00e-80 | BAQ02358.1 |
| *BTST77* | scaffold384 (+) | 139277 | 148759 | 475 | 5 | 12 | √ | 4.70e-49 | 160.5 | 1.30e-38 | 125.8 | 9. 00e-66 | KDR15737.1 |
| *BTST78* | scaffold384 (+) | 268949 | 301644 | 547 | 6 | 12 | √ | 2.00e-68 | 224.4 | 4.50e-60 | 196.6 | e-120 | XP_008485629.1 |
| *BTST79* | scaffold384 (+) | 307837 | 311682 | 461 | 4 | 12 | √ | 7.90e-56 | 182.8 | 5.50e-47 | 153.4 | 8.00e-54 | KMQ97694.1 |
| *BTST80* | scaffold384 (+) | 316990 | 322519 | 441 | 3 | 12 | √ | 1.80e-50 | 165.2 | 2.30e-40 | 131.6 | 2.00e-54 | XP_012220615.1 |
| *BTST81* | scaffold400 (-) | 342041 | 327538 | 488 | 5 | 12 | √ | 2.50e-96 | 316.3 | 6.60e-85 | 278.5^e^ | e-167 | AIU99813.1 |
| *BTST82* | scaffold414 (-) | 310980 | 301791 | 483 | 2 | 12 | √ | 9.90e-70 | 228.6 | 2.70e-60 | 197.3 | 3.00e-66 | BAQ02358.1 |
| *BTST83* | scaffold414 (-) | 326555 | 320074 | 473 | 4 | 12 | √ | 3.20e-63 | 207.2 | 1.30e-52 | 172 | 2.00e-62 | BAQ02365.1 |
| *BTST84* | scaffold438 (-) | 85934 | 77663 | 501 | 2 | 12 | √ | 3.80e-61 | 200.4 | 1.20e-51 | 168.8 | 6.00e-98 | BAQ02358.1 |
| *BTST85* | scaffold512 (+) | 103691 | 108920 | 463 | 4 | 12 | √ | 2.10e-64 | 211.1 | 2.70e-57 | 187.4 | 3.00e-108 | XP014259104.1 |
| *BTST86* | scaffold519 (+) | 192112 | 199420 | 467 | 4 | 12 | √ | 5.00e-57 | 186.8 | 1.80e-48 | 158.3 | 5.00e-64 | XP_001944504.2 |
| *BTST87* | scaffold527 (+) | 94843 | 108644 | 320 | 7 | 12 | √ | 7.60e-55 | 179.6 | 1.10e-49 | 162.3 | 1.00e-101 | BAQ02359.1 |
| *BTST88* | scaffold530 (+) | 25042 | 32260 | 515 | 7 | 12 | √ | 1.40e-52 | 172.1 | 1.00e-45 | 149.2 | e-103 | XP_008473869.1 |
| *BTST89* | scaffold530 (+) | 34154 | 41953 | 475 | 7 | 10 |  | 3.30e-39 | 128 | 4.70e-35 | 114 | 2.00e-74 | XP_001943575.1 |
| *BTST90* | scaffold530 (+) | 45347 | 57433 | 481 | 4 | 12 | √ | 2.90e-48 | 157.9 | 4.70e-36 | 117.4 | 1.00e-92 | XP_001943575.1 |
| *BTST91* | scaffold538 (-) | 297780 | 281393 | 490 | 6 | 12 | √ | 2.00e-39 | 128.8 | 1.10e-29 | 96.4 | 6.00e-26 | XP_003702933.1 |
| *BTST92* | scaffold539 (+) | 311616 | 327893 | 562 | 10 | 12 | √ | 1.50e-50 | 165.4 | 3.30e-52 | 170.6 | e-147 | BAQ02372.1 |
| *BTST93* | scaffold605 (-) | 126996 | 111956 | 490 | 6 | 12 | √ | 6.60e-52 | 169.9 | 3.70e-39 | 127.6 | 3.00e-69 | KDR15737.1 |
| *BTST94* | scaffold605 (+) | 130195 | 139622 | 507 | 5 | 12 | √ | 8.30e-55 | 179.5 | 3.40e-40 | 131 | 1.00e-71 | XP_001944504.2 |
| *BTST95* | scaffold605 (-) | 171383 | 160370 | 482 | 5 | 12 | √ | 1.30e-53 | 175.5 | 1.00e-38 | 126.2 | 1.00e-72 | KDR15737.1 |
| *BTST96* | scaffold606 (-) | 190811 | 179536 | 394 | 5 | 10 | √ | 1.70e-35 | 115.8 | 3.40e-28 | 91.4 | 6.00e-61 | KDR15737.1 |
| *BTST97* | scaffold622 (-) | 285156 | 280282 | 469 | 5 | 12 | √ | 4.20e-25 | 81.6 | 3.70e-22 | 71.5 | 4.00e-39 | XP_001944504.2 |
| *BTST98* | scaffold628 (+) | 208278 | 219991 | 525 | 5 | 12 | √ | 2.00e-68 | 224.4 | 8.70e-64 | 208.9 | 0.00E+00 | BAQ02366.1 |
| *BTST99* | scaffold654 (-) | 17913 | 2760 | 556 | 8 | 12 | √ | 3.80e-51 | 167.4 | 1.70e-52 | 171.6 | e-121 | XP_001849608.1 |
| *BTST100* | scaffold658 (+) | 132589 | 136894 | 467 | 4 | 12 | √ | 6.40e-47 | 153.5 | 8.40e-45 | 146.2 | 5.00e-81 | BAQ02356.1 |
| *BTST101* | scaffold854 (+) | 14610 | 31427 | 447 | 4 | 10 |  | 1.50e-50 | 165.4 | 1.60e-45 | 148.6 | 4.00e-46 | BAQ02356.1 |
| *BTST102* | scaffold859 (-) | 99909 | 93496 | 462 | 7 | 11 | √ | 7.60e-50 | 163.1 | 1.60e-40 | 132.1 | 8.00e-96 | BAQ02359.1 |
| *BTST103* | scaffold859 (-) | 101260 | 93496 | 515 | 8 | 12 | √ | 9.30e-50 | 162.8 | 1.80e-40 | 131.9 | e-106 | XP_008470356.1 |
| *BTST104* | scaffold859 (-) | 114863 | 103828 | 500 | 8 | 12 | √ | 3.70e-47 | 154.2 | 1.10e-38 | 126 | e-104 | XP_001943575.1 |
| *BTST105* | scaffold859 (-) | 137114 | 128809 | 531 | 7 | 12 | √ | 7.10e-53 | 173.1 | 2.60e-48 | 157.8 | e-119 | XP_001943832.1 |
| *BTST106* | scaffold882 (+) | 145989 | 154716 | 522 | 6 | 12 | √ | 1.50e-54 | 178.6 | 2.80e-52 | 170.9 | e-100 | XP_001849608.1 |
| *BTST107* | scaffold866 (-) | 35466 | 8134 | 604 | 11 | 12 | √ | 4.20e-134 | 440.8 | 2.50e-116 | 382.2^e^ | 0.00E+00 | XP014484168.1 |
| *BTST108* | scaffold1018 (+) | 136005 | 142580 | 515 | 6 | 12 | √ | 1.40e-52 | 172.1 | 1.00e-45 | 149.2 | e-103 | XP_008473869.1 |
| *BTST109* | scaffold1045 (+) | 40255 | 55275 | 545 | 9 | 12 | √ | 1.90e-58 | 191.5 | 3.50e-59 | 193.6 | 0.00E+00 | KDR18984.1 |
| *BTST110* | scaffold1305 (-) | 82962 | 81907 | 324 | 2 | 12 | √ | 3.40e-45 | 147.8 | 7.50e-33 | 106.8 | 6.00e-54 | KDR15737.1 |
| *BTST111* | scaffold1314 (+) | 568 | 16791 | 488 | 8 | 12 | √ | 8.20e-127 | 416.8 | 1.10e-104 | 343.8^e^ | 0.00E+00 | XP_012350801.1 |
| *BTST112* | scaffold1368 (-) | 5846 | 205 | 515 | 5 | 12 | √ | 8.80e-50 | 162.9 | 2.70e-40 | 131.3 | e-105 | XP_008470356.1 |
| *BTST113* | scaffold1368 (-) | 18481 | 8697 | 500 | 8 | 12 | √ | 8.80e-48 | 156.3 | 1.10e-39 | 129.3 | e-104 | XP_001943575.1 |
| *BTST114* | scaffold1368 (-) | 46013 | 38203 | 531 | 8 | 12 | √ | 7.10e-53 | 173.1 | 2.60e-48 | 157.8 | e-119 | XP_001943832.1 |
| *BTST115* | scaffold1368 (-) | 54957 | 49337 | 511 | 8 | 8 | √ | 2.50e-26 | 85.6 | 1.90e-17 | 56 | e-101 | BAQ02359.1 |
| *BTST116* | scaffold1368 (-) | 65684 | 58640 | 522 | 7 | 12 | √ | 2.00e-49 | 161.7 | 5.50e-43 | 140.2 | e-108 | BAQ02359.1 |
| *BTST117* | scaffold1368 (-) | 77020 | 68132 | 520 | 8 | 12 | √ | 3.30e-49 | 161 | 3.10e-38 | 124.5 | 3.00e-112 | BAQ02359.1 |
| *BTST118* | scaffold1368 (-) | 85763 | 78295 | 530 | 7 | 12 | √ | 9.80e-51 | 166 | 2.40e-45 | 148 | e-105 | XP_001943575.1 |
| *BTST119* | scaffold1368 (-) | 106781 | 98416 | 510 | 8 | 12 | √ | 3.30e-49 | 161 | 3.10e-38 | 124.5 | e-108 | XP_008470356.1 |
| *BTST120* | scaffold1379 (-) | 55645 | 7464 | 637 | 7 | 12 | √ | 2.50e-94 | 309.7 | 6.20e-96 | 314.9^e^ | 0.00E+00 | XP_001950990.1 |
| *BTST121* | scaffold1469 (+) | 8425 | 17748 | 450 | 5 | 12 | √ | 2.80e-45 | 148 | 6.70e-33 | 106.9 | 5.00e-58 | XP_001944504.2 |
| *BTST122* | scaffold1476 (+) | 11372 | 23826 | 525 | 3 | 12 | √ | 9.80e-66 | 215.5 | 8.70e-64 | 208.9 | 0.00E+00 | BAQ02366.1 |
| *BTST123* | scaffold1497 (-) | 6793 | 1043 | 467 | 4 | 12 | √ | 6.40e-47 | 153.5 | 8.40e-45 | 146.2 | 5.00e-81 | BAQ02356.1 |
| *BTST124* | scaffold1531 (-) | 30919 | 17338 | 514 | 6 | 12 | √ | 6.40e-54 | 176.5 | 3.90e-43 | 140.7 | 2.00e-67 | KDR15737.1 |
| *BTST125* | scaffold1750 (-) | 10167 | 1297 | 475 | 6 | 12 | √ | 2.20e-45 | 148.4 | 5.30e-31 | 100.7 | 2.00e-61 | KDR15737.1 |
| *BTST126* | scaffold1750 (-) | 22180 | 12767 | 482 | 6 | 12 | √ | 3.90e-44 | 144.3 | 1.90e-30 | 98.8 | 5.00e-52 | KDR15737.1 |
| *BTST127* | scaffold1882 (+) | 11840 | 29435 | 556 | 8 | 12 | √ | 3.80e-51 | 167.4 | 1.70e-52 | 171.6 | e-121 | XP_001849608.1 |
| *BTST128* | scaffold2134 (+) | 5653 | 16606 | 547 | 5 | 12 | √ | 2.00e-68 | 224.4 | 4.50e-60 | 196.6 | e-120 | XP_008485629.1 |
| *BTST129* | scaffold2134 (+) | 22394 | 27969 | 461 | 4 | 12 | √ | 7.90e-56 | 182.8 | 5.50e-47 | 153.4 | 8.00e-54 | KMQ97694.1 |
| *BTST130* | scaffold2784 (+) | 5318 | 9259 | 417 | 4 | 10 | √ | 1.70e-35 | 115.8 | 3.40e-28 | 91.4 | 6.00e-61 | KDR15737.1 |
| *BTST131* | scaffold2900 (-) | 10912 | 3435 | 490 | 5 | 12 | √ | 2.00e-39 | 128.8 | 1.10e-29 | 96.4 | 6.00e-26 | XP_003702933.1 |
| *BTST132* | scaffold6879 (+) | 134 | 20150 | 578 | 9 | 12 | √ | 8.00e-58 | 189.4 | 7.30e-58 | 189.3 | 3.00e-172 | BAQ02372.1 |
| *BTST133* | scaffold6880 (-) | 71918 | 66186 | 459 | 5 | 11 | √ | 1.10e-55 | 182.4 | 2.60e-41 | 134.7 | 5.00e-77 | XP015363132.1 |
| *BTST134* | scaffold6881 (-) | 18037 | 10835 | 449 | 7 | 12 | √ | 2.00e-86 | 283.7 | 2.10e-81 | 267^e^ | 1.00e-157 | XP015363132.1 |
| *BTST135* | scaffold6882 (-) | 9669 | 6471 | 473 | 3 | 12 | √ | 3.70e-60 | 197.1 | 1.50e-52 | 171.8 | 1.00e-128 | XP008482330.1 |
| *BTST136* | scaffold6883 (+) | 58779 | 70964 | 463 | 7 | 11 | √ | 6.80e-60 | 196.2 | 3.20e-52 | 170.7 | 6.00e-109 | [BAQ02359.1](https://www.ncbi.nlm.nih.gov/protein/733604584?report=genbank&log$=prottop&blast_rank=1&RID=NCG1JY0R01R) |
| *BTST137* | scaffold6884 (-) | 53635 | 37615 | 529 | 7 | 12 | √ | 7.50e-75 | 245.5 | 8.00e-71 | 232.1 | 0.00E+00 | XP014272907.1 |

^a^TM (Transmembrane) predicted by TOPCONS Server (<http://topcons.cbr.su.se/>);

^b^Pfam e-value and score for sugar transporter signature PF00083;

^c^TIGRFAMS e-value and score for sugar transporter motif TIGR00879;

^d^Information of top BLAST hit e-value and accession number, whitefly sugar transporters versus nr database (Non-Redundant Protein Database, NCBI);

^e^indicated score above 237.8.
