# Supplementary material for: Genome-Wide Characterization and Expression Profiling of Sugar Transporter Family in the Whitefly, Bemisia tabaci (Gennadius) (Hemiptera: Aleyrodidae)
Source: Front Physiol. 2017 May 23;8:322. doi: 10.3389/fphys.2017.00322 (PMC5440588; doi:10.3389/fphys.2017.00322)
Supplement: Supplementary file 8 [file Table8.DOCX]

**Table S8. Amino acid sequences of the *BTSTs***

| Genes | Amino acids sequences |
| --- | --- |
| *BTST1* | MPRCIPRHIFNQIVSSITAFLTLLLSGIWLGWMSAVLPKFRGGEIPIPMTTDDLTWTVALMDFGNLLSPIPTGYLMDRYGRLLTLRLAMLAFVAASALVLAASAPYHLFLARLLAGVGKGVGFTAATLYVAEIAGAKIRGALSGVFIVMLMGGTVVSMTVGPYVSFTTLNVITAVCPVVGLFLTLFIVESPYYHLIRDDPAAAGEAFARVRDQSKGAANEAEFALVKRKVAEDMSGQKSILNLFTEKGNRRGLIIILVQGFLQRSGGISCILAYASTTLPSDSFWQGKISVMVFSWIMVVFGLVALSLVDRFGRKPLHLISCVGLTLVTGVSAVYYYFYQKTEVDVSQFMFVPHVGVVLFGVFYPVGVGQIPHTLQSELFPTSVKGQASALMTMALAISSFIVNKVYFAVDRGLGVYFMYLIFALSNFLSMVFTAFYVFETKGKTLEEIQHFLKK |
| *BTST2* | MVIGFPAILIPAVTNDDNADNLHLTMAQASWCASLSFIFQPVGGIMTGLCLQSLGCKAVMILLNIPHIVCWLMTYYASSIYTLCFAQAFFGCVLGLIEVPGLRYVSEISEPSVRGIIISSTSFFVSVGYLIMIFIGSLTDWRNAAAISASLPLLCIILLILIPESPMWLMSKGRSEDALRSLQWLRGWTSAQMVHEEFQRIQFYSKNKTQKFLKTDHSGDSRSGSGGLSYFTTSTFLKPLIKCCIIFAIFDFGGMISFRSYLVKILQDLHSPVSSKWSSLWVALFGILGNVGCMLFIKKVKKKPMLRISLLCCILCLIFLTMFLFGYMQVIADSAFHHWCPLIMIVALFFFYNLGIHPIAWAFLGEILPYRGRGPATSFVVCSHNLFTFVSVKTFPNLTQWLGLEGALLVYAGVCLCGILFTYFLPETEGKHLSDIEIEVACGGKTNEDGLKATPCGS |
| *BTST3* | MELHLDTCPDGRSQQCQSSSLRQTLAQVLAATGKSLIMLSIGMLIGFPTVLIPVLTSKDYKGDLHFNRDQASWYGALTYIFQPVGSIASGALLQSFGCKKLMILINVPQIGCWLMIYFATSNFVLYVSSALVGLVIGLMEAPTIRYISEISHPSLRGILTTYSVLFTSLGFLAVYSLGSLTDWQHVALISAAVPVICIIILFQIPETPMWLMSKGRAAEALEALQWLRGWTTADMVEDEFAKLEYYAKKKSCKTLHGYGKSVDTKDEVKETPAENGIDGVSGGGKVSVKPSVSFQVEVGFEKRGFADKLGDLTCKEMTVPLGKCIVLITLSCFSGLPLIRPYLVAIFAQLNLPIQPNWTSVLVTFLGIAGNIGCMALVNRVGKKPLVICSSAASAVCLILLSIFLMQSTPEAMQDRANPNWWPLILFFVLFFSFNMGLQPLPWVYLSEILPYKGRGIATGIAASIFYIVIFFGVKMFSTMERELGLQGTFLLYAAVCIAGIFFTYFILPETEGRFLSDIETDAEREITVISNKGNTVNA |
| *BTST4* | MSCSSGNAVYAALPKHGDPVEIENFDVERPSTKTEFIHSKRDILYQGFTSLVAFSHVIHAGINLSFSTVLHPQLDITKEQASWIASLGAVGTPIGSISIGFIMDRIGRQKTLLATAAINILAWSTLCLSPAQVDIKMIYLARLLEGIASGMTSIAIVYVAETTDKHWRPLFLSMNSIALSGGILLTTTVGVVLAWRAFAVFCLGVSTLSALLSLTLPESPSWLAQHHPERAKQALARLVTDPRAFEIEWTSLEPKAPKLLKTRKRPSLDKKIIRPLAVVLILITLQQLVGIYPTIFYSLELFQKINKRAQQSTEFNSSEETTMIIPTTPHIVTTPSSAQLFNSSSFEKSTGYPLNLDVFNTSALIDHQHNVSAFIRGTGNPHDTRAFIQRTSLPQDAKVVINGTKHELGDELGEIKSKFDLRSGAVQALMGLGIIRFITSVSMTALSKHLGVRTLLMSSSLGVGLTAILFSAYQAGWCSRSSDAVSFAIVLAFITFGSLGLLVIPWTLVGELLPFEFRGVGQGIVVAYAYVIMSVVVKSYPSVEDELGTSAVFGFFGAVAFLTIAFIYYFVPETKGRTMDEIQVYFQKR |
| *BTST5* | MENKRSDEYTALKLKNFDNNVQIVKHEFPDSESSDTDHRFRDGLYQSFVSLGVLSLLVHPGICVTLSTILYPQLNITKYQNAWIASLLSIGMPIGSLFVGPMLDKFGRKRTCIFTAILTSITWAMIISLPEDFDLNLLYIIRFISGIALGLATSVIVYVSEVTNKHWRPVYLATVSVFLASGVLVATTVGIMMPWRSFALFSFGVSLVNITLILLVPESPHWLVRFSPEQAKRALVKLNKNKQDFNEEWEALEARRSARASSTFRKARLLSREVRRPMLRLAMVFTFQQLGGGYLVVVYALQMFQTVVQSATTTSNHAPTPAPTNVTLPLVQKHEFRLGDLDTLCFICVGVIRVGMSGLAVLLTKHVNSRPILISSALSSAFSAFAFAAVMSGVFGPVRDLLPLFFVLLFLLLQSYGMLVIPWAQIGELIPLSYRAKGGGFMTAYAYILMFVAVKIYPYAVDWVGLASVFVFFGLVAALASVYVYLYIPETHGKTFKEIEDFFK |
| *BTST6* | MEYDNECTSLRHEENSARIVKGAAEKNGAVTSDHTWRDGFYQGFVSLVALSMLIHPGVGMGFSTILHPQIKHLVSDEQNSWIASLVAFGTPVGALSSGPLMDKFGRRSTCILTCGVAIASWSALVFMPPEFSLLLLYIARILSGVAGGLTSAGVVYVSEVTNKYWRSVFLGLASVLLSTGVLLVTSVGYWMHWKSFSVFCLAIAVLNLLLLLTIPESPHWLIRTRPEKAKRALMRLNKNIESFEEEWQSLDEQFRKKQKAQMEGTPRPSLSSRQVYVPFLIMGVTFTMQQLCGVYPIIFYALEVFQAITGDPGEISPSANSTNPSTETTTLASLIGNSTLMATDGSRQDLKVKSLIGVGLIRFVMSILAVSLSRTIGRRPLLLSSCAGSAISGFAFSLYFFGCFGQEVNDLVSMSLVLVFLLFSSYGLLVIPWAQIGELIPSSHRAKGGSYLISYAYFLMFLVVKVFPFTMETFGIGGLFMFFSIILTLEGFFVYFYMPETLGKTFLEIEKYFATGVDSNGKGARSKWPIDKV |
| *BTST7* | MFPGLESVALVYLVELSVKEYRSLLLGATAAIYTVGILISNVVGGYLPWHLASGIFSLTAFGFGVVHFFAPESPAWLYKSGRPDAAVRSLQALGRSPASVRAELQLLELSARTVSENVSVAVLLEPTVWKPLVIVSLLLVFQAFTGGYQINSYSEDIVQRLGTKYDPLHVSNIMSVATAVTNCTLGVYCISYMRRRPATITLSILVTLASLGAGIYELLLRGAPGPFDWLPIALLAANLSLGSVVTNISWILSGEVFPLRVRGSTTGAIFFVGWGSQSLAIKLYYASLAALQVSGLCFAYAAGSLCTVPLAVFALPETHNKTLYEVEQSFKRRESKDAETPVQDESNL |
| *BTST8* | MKSALKELHRIWESGIGRTVAATVAAHLNSISVGMCQGYSAVLLPQLTSHASPLQVSNDEASWIASLGVISNPVGALLSGVCMEIFGRRTAVQLTSLPFLIGWTIIALSQTLTTLCIGRFISGMAIGMASACYVYVAEISQPEHRGILSSTGPVFVSLGVLIVYSLGSLCSWQFVSAVCAAAAMLSFSAMQLVPESPYWLASKGMTKESHAALSWLRSSAHVEKDISELVNNSRDISPRVSTLKLISDRFNDPCVWKPFFILVGFFLFQEGSGIYIILYYAVDFFRRAGSTVDHNVASIIVASLRFAMSIFGSLCIQNFGRRTLAVTSGILMALSIGAAGVYEHFFEDFAPADRPYPWVPLACILTNVCASMLGLLQLPWLMIGELFPLKVRGIMGGVVSSLAYLFIFATVKIYPNLMANLQMSGSMFGFAIASLMVVVYALMFLPETRGKTLLEIEQRFCDIPKTNSTENLEKGFYINPAISVSTVCAIVENIKK |
| *BTST9* | MYRRFKDITMSAKKELDLAVAADGDQRPKAAPNKEEKPLISSKAKFSPFFRQFLAASGPIIATLSSGMTAGFSAVLLPQLKSPNSTLKIDHDQASWIASMAALPMALGCIFSGVLMERYGRRMTQLLLCVPFLLGWVLLSLATTVWHLYVGRFLTGFSVGLLGPPSIVYIAETAEPRHRGALLATVTLAISVGILLSHVLGTFLYWKVAAAVSVFFPCLSFGLFWICPETPSWLAIKGYTSEAEEAFHWLRGYSDQAQGELKVILSKKPASRSDSEEGSFKRLAHSLRSIFSLSFLKPFFIMNVFFFVQQFSGVNAVAFYSVDIMKTVSGNVDEYLATIVIDVIRVVMSLATCILLRQFGRRPLGLISLVGTTVSLLSLAAVLKTPFYKEYPSLSWLPTGLLASYICFISIGLVPLPWVMTGEVFPAAHRELGSGATSFFGFFVFFVVVKSSPFFFSTLGMVGTFQLFGGITLLGTVFIFCFLPETKNKTLEEIEDLFSKSKPKPSPESAEVV |
| *BTST10* | MHNQAEPSTSLDVPEQNVKDHFQYANATKSAWAQILASIMQNWLFIEIGLELVMPTVILGSLHNNPAEPLNMNDDQASWFGSIPAFCHPIGSLMSGLLQDKFGRKGAMMLVNIPIFMGWMILYFAESIHALYIVSVIMGLCTGLAEAPLHAYIGEIGEPRMRGTISTISTSCCSIGVWLMFLFGYLFDWRTVALVSSSCSIITFTFMTQLPESPTWLTLRGRLDEAKKSLCWLRGWVSSAEVEPEFLSLVKYTVKSARLSQENSAYSSLPIKEGEPVAKRGGFLKEQLKELTNKRTFRPLRLMFIVFIITDIAWVHGIKPYFVKELRMLESPIDPNLALMIFSGLFIMGAMVNVAFLRRFGKRRIALFSHILAGICILSIGMYASFLQSLTQYPLRVWLPLMLWFIIKFLHGFSIITLPWQLVCEVFPLSGRGTATGLAAAWTHIVMSVLTKTYLYMEAWLGFSGVMYLYGVCTMAGVVHHYFYLPETEGKTLEQIETYFTKNHDKREKFSVGKLQRQRAQSGSPF |
| *BTST11* | MGDSPKSSEREKLIKRYGYSSRSDFAQISATLIQGFILINHGLFMAAPTLILGALYEHPEDELYMDDDEASWFGSIPYICTPLANLASGLLQEMLGRRGSGLLSTMPMFATWILLYSANSVTTLYAVAAMMGLSVGLSEAPLNSYLGEISEPHIRGTLVTIASSAISVGIIVLYALGSFYDWRTTASIISVVPVITFILMTQIPESPAWLIGRNRLEDAKKSLSWLRGWTSPNEVEEEFADLVSYTRHHVEDDEGALAKVAKMNGLLMTQVNAVFSKKVLRPLRLVLISFAITFLAAISGMRPYSINELNAMNCPIDPKLILMVAQVLFIGGAVVNVSFLQRLGKRKIALFSYGVAAVSIIGIGAYCSFYRDLSSYSSAVWLPPSLLLVINFLGGLSILVLPWQLMSEVFPPAGRGLATGISAAWTHLVVSALIKSYLSIKSRVGMDGVMYLYGGATALGCVYFYFCLPETEGKTLEQIEACFVDQPDTEEMLSVGASPVRHPENTRLRGNRKERGYGSTARSEL |
| *BTST12* | MSDPSPASGGPSDEKSSLTTKRQYGYSSRSTYAQALATIIQGWILIDHGLMMGASTLILGALHGNPNEELNMDDAQASWFGSLPYFCTPLANFASGFVQEALGRRGSGMLVNIPIFAAWILLSFADSIATLYVVAIIMGVCIGLSEAPLNSYLGEIGEPHIRGTLITMTSTAISTGMTIMYGLGVFFDWRTTALISSIFPVLTFALMSQIPESPAWLISHNRLDEAKKALCWLRGWVGPDEVEEEFQNLLMYAKKSAKEAKASEKTDDIDSKTDGLLMTQLKALTNKNVLRPLRLVLISFTITFLAVVSGMRPYSINELNALNSPVDPKIILILFQILFILGAVAYGSFLRDFGKRKIALFSNGIAAASIIGIGVFCSFFLDASEYPYLVWFPSILLLVINFLSGFGLYALPWQLMSEVFPQAGRGLATGISAAWTHLVVSALIKSYLYIKAWVGLSGVMYLYGATTFLGFLYYYLYLPETEGKSLEQIESYFTDDPDLEEFFSVRRSPATDAEKIGLRSNKEDHSYGSTA |
| *BTST13* | MRDPPGPSVSLIDDAVDVDEDVPVPTNTRYEYSNRSTFAQVLATIVQSWLLIDNGLMKAVPTLILGSLHDNPNEPLDMNDDQASWFGAIPYICTPITSFASGIFQEKFGRKGSMILVNIPIFVAWILLYVAESIAAFYTVAVIMGLSIGLSEAPLSCYIGETSEPHLRGTLATIMSTAMIIGYFIMYTLGYFFDWRTAALISSAFPIVTFVLMTPIPESSTWLIGRSRFKDAKKSLRWLRGWVTAEAVEEEYQSLLSNNRDPIRKKPSESTLQERGPEQEDYGFFRTQYQTLMNDNIMLPLRLVLITSFIGYVAVLRGMTPYLIGELNALSTPIDAKLVLIITQILFLLGAAADMMFLQGLGKRKIALFSHAIAAVCLLGIGFYAFHLQASAVYYPHLAWLPVIFLMVINFLGGFSLQVLPWQLMCEVFPRVGRGLATGISAAWTHLVISLLIKSYLYIKAWIGLGGVMYLYGTITAFGVVYFYLHLPETEGKSLKQIETYFTSNHDRKEKFEVEK |
| *BTST14* | MSDPSEHSKSLCTGKNPEKTSSRYGYSSRSAFAQVIATLIQSWLILENGLLFGAPTLILGALHGNSSAEGLRMNDDEASWFGAIPSICTPLASFASGYLQDRFGRKGVALLANIPILATWLLLYTANSIPALYIGAGMMGLSQGLAEAPIISYTGEISEPHLRGILSTITSTAVMIGMIIMFVLGYYFDWRTATLISAAFPMITIAVMTQIPESPTWLLGKNRLDDAKRSLCWLRGWISNEEIEEEFQNLVNYTRNSAKEDCSATSNGHVQDSCNVKSDGFLKTHYNILTRKNVLRPLRLVMLTAFFTFVAVLVGMRPYYINELKALNSPLDPKLLLIIGQFLFIGGAVTNMAVLWMTGKRKLVLFSYLTAAISILGLAAYSSFLKDSATWVTWIPIVLLCVISFLSGLSILILPWQLSGEVYPPVGRGLATGISAAWTHLVISALIKSYLYMKAWVGFSGVMYLHCGCVVIGFVYLYCNLPETEGKSLEQIETYFTKNVSRREKFSISKATNKSKSLRGDQRL |
| *BTST15* | MSDSQETISAHDEVPLVPRNTRYGYSSKSTYAQVSAVLIQSWLFIDLGLQMTMPTLVLGALHRNPDAAPLDLDDDQASWFGSIPDLSLPLASLSSGLFQDTFGRKGSMMLVTIPLFSGWLLLYSARSITTLYAVAVIWGLVGGLCEAPLMCYMGEIGEPHLRGTLSSISTLATLTGSFMMYTLGYFFDWRTAALVCSAFPAITFVIVTQMPESPSWLIARNRLDDAKKALCWLRGWVEPHEVEEEFQDIVNYTRMSTGVETILDDSDQSIEGNSLGKKDGYLKTQYKQMTDKKILRPLRLIFALFSICGVASVAGNRPYLIGELTELGVPINPKLVLIGALVCFTLGAMGNVIFLRRFGKRNIALVSHFLGALCIFGIGAYCSYAKVFTPEPQLRWLPVILWFTLQFLAGLSIILLPWQLVSEVFPLTGRGLASGIAGAWAHLSSSILVKSYLYTESLISLSGIMYLYGCGTVVGLIYLYLYLPETEGKSLEQIETYFTDQHDRKEKFSIGRPSSFSHASP |
| *BTST16* | MTDTVVPSRGSDVQHGKSTSNHQTKSSYRSGFAQILVALIQSCLIFDHGLEMGIPILVIGALHRNSSEALNMNDDQASWFGSILNIVHPVASLTSGFFQEKLGRKGSMISTTIPLFGAWMTLYFAQSVYALYAVVLIFGLCRGLTEAPLHAYTGEIGEPLLRGTMSTISVSAAIIGASVVFALNYFFNWRSVALICSAFPIVTFALLTQIPESPTWLISKNRLDDAMKSLCWLRGWVEPNKVETEFKNLVNYVRNSTEQNESSSNGSDQTKKDGFFAKGTRYLATNYKIMTSRKVLRPLRLVFIVVVVSLTAFLAGIRPYFIREIQELKSPIDARLILVSSTGSLFIGAIMNVAFLRRFGKRKIALFSHAVAACCIFGMGAYSSFLRDAGSYVQLRWIPIIFWLLLNVVCGLSISVLPWQLICEVFPVAGRGLAVGISAAWAHVVMGLMVKFYIYTEAWLGFSWMMYLHGAGTLIGLTYYFFYFPETEGKSLEQIEQYFAGNYNREENNSIDRRMKY |
| *BTST17* | MSSPEKPVKIQPIYQGCETNKFRYKNASRSTLSQVVATLIQNWLLIDLGMQLVMPTIVLGAIHNNPAEDLSMNDEQASWFGSILFFAHPIGSMVSGFLQEQFGRKGSLILVNIPIFAAWSTLYLAGSIYMLYFVSLAMGLSVGFCEAPLHSYIGEVGEPHLRGTLSTVTSAACILGMLIMYIIGYLVHWRTAALISSAVPVITIIFMTQIPESPTWLIMKDRLKDAQKSLSWLRGWVEPEEVQEEFQELLSYTKISPPPYQANDIEIEKYELVRTDENGREVAKEKESYLQAKFRELTDKKLLRPLRMVFIVFVFCYASSLIAMRPYMVGVFNEFGFPMDSKLILILTSAFFFVGSILNVVLLRRLGKRRLTLLCQGMASVSIVLLGVYCSFFDRTNRIPSLVWVPISLLVSISFFSGLSVALLPWQLLSEVFPLKGRGAAGGISAAWAYYVGAVMSKTYLYLERWIKLNGVLFFYGAISLIGFWYFLRYLPETEGKSLEKIESYFTKNHDKKEKFSKPKRSKKPVSPL |
| *BTST18* | MMPAADGSAPRTSSKWFRTFLAVTGAIGIEFIAGTIEAQSAVLLPQLEGSKELPITKDQASWIASMGTLLCPLTSILCGPLMDILGRRLVFKIYYSVSTIGYLIIAFAKEVWHLYIGRLCLAFSLGFTVANVIYLPEITTTSQRSLVLATINPLFSLGLLFSYVVGGYLRWDVASLIHTLICGLGLFSVLFLPESPAWLVKEQRPEEARNVFRWLGRNAAKIDGDISRLQTTGNGPVKRSIPLKQLRHATVWKPFLILITFHFLQTMTGIYNIMFYTVEFFRDLGTAFDPVLVTIGFAFSRFVVCVTVGYYFTTKCPRRVATAVSGFGSGAAYLVAVAYEVWWKGDRRFQWVPVAAVLVSPGSPVDYDRRGVPALRARVHGRRDILRRERVPVPVHQVPLRAGRGPGDARRDDLLRGGVPRVGRLRAPRPPGDPGQDHAPDRARLHGQEAEAATGEEVPIRQGGGALVRAAQRGSSTARGSARDGGGVRCQTTINGMEMLHV |
| *BTST19* | MGVGTYRQLVAGITASISVMCLGIAMGWSSPILQKFATERPSPILPVPTEDQLSWMIAFMEFGNLFTPVITGILVDVIGRQKTLVLIGPLFALSWLIIYLSQTIYFLYVARVIQGLGCGVVYTAVPIYLGEISDPKVRGALSNLFQGFMYIGLLYAYVLGPFYSYSNFTLFCMAIPLVYSISVLFVPETPYFLLMQNKDTLARKVLRELRDSTDDINEEMRVMKESVEKEMEGAKNPDKNRKITNIKLFLISQFFGSCQIMTNMYAILTYSSMVFDKGGSHWLTPDQYTIFLGVVTLVSTVPSSFLVDKLGRKPLLVTSAVTCGVLELLAGVYFLLRERNAIQGDDYGWCVFVFVSGLSFCYSFGLGPIIPTIQCELFPTNARGLAFGLTILITSVTAFLNILQFQWFASSPKLGMSANFFFGCVMCLLVAEFTVAVMPETKGRTFAEIQLLFHKDEGVERVSVLEAGEGDGPGSRLNPEEREQDEGAGVGS |
| *BTST20* | MLIPRCHLRQYYAAAIASLSAMMTGAAMGWPSPVLEHFCEGAHCEVRMTAGEASWVLSLIEIGNLFSPIPCGYLVDMYGRQPCLFATGPLFLGSWTLIICSRSVGYLYLARLVQGLGMGIVYTVTPMYISEIAGADVRGRLSILFVGLLNLGILLEYIVGPFVSYRTLGYISISVPILFIATSIWLPESPYFLLMNDKSKEAINALMWLRSDYSKDRIFEELTLIKDEVEQEKGTKTAAKNTFGDIFSSAANRKAFLIVQIAACADVLSGMTAILAYASVIFAAPHNTTMEAEDYPIMLGLIMLLAIFPAAYLVDMAGRRPLLIFSCLFSGLFELVAAFYFYAAMKLDCDVARLKWIPLLAICAFSVAYSMGLGSLVPTLMGECFPSQIRGPASSMTSITLCAISFLVIKFFQVVNEEIGLYFNFFIYGVSSIACSAVLWLVLPETKGKTLSEIQQDLKASCKPSKPSLPLYVNASVPKGVEAS |
| *BTST21* | MFVKLQRGVVNQIAAAFTAELAGYNFGVWRVWPSLSITELRAGSAGFAVSDDQLAWITSLLYLGFLLTPFFCSYLVVRLGRRTILYLTSALHTVSWLLVVLAQNPYHLYIANFFGGLAGGVGMTMVPVYVSEISSVNIRGALIGSFLVFINLGQVMMVNMGIWLSYQEVNLFGLGGASVAFVLQFVVLTESPFYLLASKREEKATEAYKRFHASSGEDKIETEVSALKAAVEKDMEHKSSYMELLACKRAFIILVVESFFQNLGGANSMLAFGVISLPKTELFLTPHQTILICVISGTFFNCISSSIVDSVGRKPLLVISVGMCSVFTGGLCVYFFLVEELQVDSLEYEYIPHLLLIGFIASYTSGFAISRSLIIGEFFPTNTRTHAGLTSTFCFATAGFVVTLSFLHVVRLIGLYFMFFLFFLVNFTNFWFSLFFFVETKGKNLFEIQEYLKSL |
| *BTST22* | MTPRKPPVPSPNQFFISCENVQLMQTEWSDETTNNTSTNTDDIDRPPASIFKPVLATLASTACQFFLGAMLGQSSTMLPQLKAEGSSIRITDEQATWIASMGVIGTPMSSILCGPLTDKLGRKRIILVFLFLSAVGHALLGYSSNLTEILIGRFLLGTAAGFGFPSLVYISEISTPKHRSLLLSSATISASLGLTYVYSVGGSMPWDRASLITASLSLLALVYACSIPESPAWLFQHQRRQETIESLKWLKGQHCNIEVELKLLEASCNHQKKRALALSQLTKPTVFKPFLVLSCLAFMQNGTGFYILLYYSVDFLREFKTTMDPQKVSIALALTRLISCTVASLFIKRLKRRTVGIFSGLAMAGILGVIYLSLTAFREAVGSGPVPVLSLLAYIFACSLGVHPLPWLMIFELYPLSVRGLMCGLSNSVCYLFTFLFLKLYYVMITNLQIHGTILLFLCSSAAFGLFSAFLLPETQGKTLLEIEQGFMSKKDRAARRSG |
| *BTST23* | MEADKLPVSEGFIRPFLLSACIYPLHIVGGAIMGQSAGMLPQLLNKDSSIPIDMEQATWIASSTAIGTCISSAISGPLSDMFGRMRVVQMSYFLMALGHALMMAASSFTGLVVGRLTVGLGLGCDFASFIYVSETVPAALRGVLMALYTVMCSLGFIYIYVVGGYYHWTIATGINAITATSGLALSFFLYETPVWLVRQGRLKAARKSLRQSGIAASNLEAKLKELQDAAENKSTETFSLGDLLGPTVWKPFSMVCIMAVLQNMAGFYIVISYSIQFMAEFHSGYSPVQVTVGIAVVRLIAMTLTSFWMRHARRRVIGSVSGFGSSACLLAVFAFLHFGHLAPVLVENQWILIALFFAYIFTMTLGIYALPWTMPFEIFPMKVRGLMSGMTYVSQFIAMFVSVKLYNVLMDNLHLQGMILMFAVGSALFGSFCVTWLVETHRRTLDEIEAEFAGKSKVYT |
| *BTST24* | MEAEPPASAPTGCLRPFFVAACLFPLHICVGSIYGQSAGMLPQLLEKGSSIPIDRDQATWIASAPTLGACLASAISGTLSDVFGRMRVVRLSFFCMALGYAVMVAAESFMLIVLGRFLAGAGIGCNFPAFVYVSETAPPAYRGLFLSLNALMSSLGLVYIYSLGGYFPWVYAAGATCLMATAGLVLTFFLHDSPAWLVRNGKLEAAQKSLRRIEENAANVEIKLKELQETAKNEPTSSFNLRIFIEPTVWKPFMQVLGMSVLQNIAGFYIVIYYTVQFMSEFHSTIGPLEVTVLIAVVRLVGISVASAWMRHAGRKFIGAFSGFSTAVVLLAIYAILKLGDRVKFLAENNWILIALFLLYVLTMTLGIFPMPWTMPYEMFPIKIRGMMCGVCFCAMHVVMFVSVKLYNVLLDNLELDGMILLFAAGAALFGVFSASMLVETHRRTLDEIEAVFAGRPIVTPPQKQKT |
| *BTST25* | MAESGDRKESLSWRCWSRTLIACSGAMMAFVFSGVTEGQSAVLLPQLKDEASPIHLTPEEETWIASLGIVLSPVSASLTGPITDAFGRKLGLVVYHIIMGIGFAVIAVAKEVWHFYVGRCICSFAIGLEVAAIVYLTETCSKEQRGLLLSTISPAFTIGVVVAYVIGGFLPWNVASAIFAAGSFLCSLGQLFGVESPAWLYKRGHTEASTRALRRLGRTQAGIRQELELFKLTVKEQSQKFHLRELLHPTVWKPFIIMTIFHLIHCATGVHHIVYYTIDFINRLGTTYDPLTVSIAISVARTIATCTIGVYFTSYVKRRFATILSGTLMTILSVGAGVYVYVWRDTAVDRRPFQWLPVACVIAYIVIGRVGVTPLPWLMSSEVFPLRVRGSMSGATFVIGTGSIFISIKMYEDLIAAFHIWGLLFIFGTACFSAVLLAVFVLPETLNKTLYEIEQYFMPKKGKKSGEQVDSTSRGEVIDSSPFKAVVKNRFLRHSLRTPCLSIIFQRFIWQINRY |
| *BTST26* | MFSATPGVRRQFAAAITCSIGCLIAGLMVGWPAPTLKKLRQPDSPVHLTPHEEAWVVNAMYYGTILSPFPSGFLINLIGRKTTLLVLAVFPTLSWILVYFSTSATMLMVARLFSGFWLGGIQTVVPLYTGEISEPHVRGIFGSFFQVSSFVGNNFSFIVAPYVTIQTMATICGFFPVLFIILFVFCPESPYYYAMKKNSKAAGKSLSWLRGDEPIMKELQTIQTSVDKELKDDETFTQKLTSIATDPANRKGFIIVETLDVMQRLCGISCMKAFSSIILPPKLGPLTTDHCTIIIGFVWMFSSLICTGLIDKAGRKPLLYASSLGIFVSMLWTGIWYYLNDNTDIDVSSFKWLPLAGFLVFGVTFSFGLGPIVKIYQGEMFPNNLKGMASALTAIIAAFASSVSTGMFPILTETIGMYANFLIFSTVGLVNFLFTYFYVIETKGKSLQEIQAAELNGERPVVTQSNGDSEKAV |
| *BTST27* | MATLPGKGEAPKGQELEKVAPEKWCRVLWACGGAMMIFFFSGVTEAHTAVLLPRLEEVDSPILIDADEKTWIASLGIVATPLSSVLCGPCVDYFGRKIMVQCYYLVCALGFALIASANSVYQIYAGRLICSLGIGFEVAAIVYIAEVSTVRMRSVLLSLTYSVLYGGGTLFAYAVGLSLPWNLGSAVFALVCLILFGYESFVPESPSYYYKKGDTKKAIVAFTQLGRTEDQIAQEIKILEERKTKTEQKVDWRTFIHPTVWKPFLIIAFFHCLQAFMGLWDELYYTVDLVTELDSAYDPFEVSFILTLSRFLVASTAGVYFTTRVSRKLAAAASSFSMAVALLVVAVYEKRYELTAKWERPYPLVPIVGLVGAVMASGAGMFFLPMLMSGEVFPLRVRGTMSGAVFFVGTGSMFLFLKLHVFLVTTLGVPGIYTMWTTACFVAGFFAVFVLTETHGKELHEIEDSYRSKKHRSTDIERTKF |
| *BTST28* | MEFATEQEPKQTPSEKWGRMFWACGGAMMIFFFNGVAESHTAVLLPRLQEPDSPIHINPDQMTWIASLGIVGAPVSGVLCGPCVDYFGRKIVVQCYFIVCALGYALIGAASSVYEIYVGRLILSLGIGFEVAGIVYIAEVSTARMRSVLLSLTYSVLYGGGTLFAYVVGLSLPWNLGSAVFALACVLLFGYESFTPESPPYLVKNGHTDEAIAAFKRLGRSDDQIAQEIRILERKGEPRQQVEWRTFLEPTVWKPFLIISCFHFLQAVTGVWDTLYYTVDLVTNLGTQYDPYEVSLFLTVGRSLMASTAGVYFTTRVSRKMAAAVSTFSMAVSLFILAVYEKMYEFTSELERPYPLLPICALIGAVMASGAGFFFLPMLMSGEVFPLRVRGTMSGAVFFVGTGSMFLFLKLHVFLVTTLGVWGFYAMWTAASFITGFYSIFVLTETHGRELHEIENSYRSKKQKGADIERTSQF |
| *BTST29* | MLTLTLGIRRQLAAAFACSLASLIAGCVLGWPSPTLKKLREPDSPLHLSTYQEAWVVNALYYGTVLSPFPSGYLMNKLGRKMSLLVLCVFPTLSWILIYFSSSAYMLMLARLFAGFWTGGTQTVMPIYIAEISEPQVRGVFGTFIQLNIYLGTNFAFLVGPYVSIQLMAILCGILPVIFFVLFGLCPESPYFYTMEGRHAAAADALTWLRGDAPVDAELRTVRHSVEKESANQSGVFRRIADLVTVPANRKAFIIVETMNALQRFSGISCMMAFSSVVLPETGALNSDHCTIIMGIVWMVSCLGTSGLIDKAGRKPLLYVSSIGIGVSMLWTGVWYYLDENTTYDVTGWNWLPLAGFLAYGCTFSLGLGPLSSTYQGEMFPSNLKGQASAITTITTALASAISTGLFAVLSKNVGVYMNFYIFSAVGFINFFFTYFYVIETKGKSLQMIQAELNGEEIIKPEMKRLGKSVKK |
| *BTST30* | MYTFACSGIQRQLAVAFTAAFSQFLIGFLMGWPAPTLKILRHPSSEVHLTPSEEAWVVNAMYVTSFLSPLPSGVLMDTIGRKTTMVVLCLFPIISWILIFYQQTGLMLLIARAFAGVFVGGVQMLSPVYAGEIAEPRVRGIAGALIMVHGFAGAISVYIIGPYVSIRTMAVIGGAFPIIFLLLFTLCPESPYYYIMRGRQKSAEEALTWLRGGAPVKQELDIIQTAIEKETQSGKGYFTKMLSLVTVPGNRKAFFIVEVMNFMQRFSGLSCLTVFSTIVLPERVGPVTSDHGTLLMGVCCLLASLGCIALIDKAGRKPLLYFSSIGIFFSMLPTAFWYYLDRETSTNVKEVNWIPYAGFLSFAATLSLGLGTIAPAYKGEMFPSDLKGQACALTSIIVGIASALGTALFPVLTSHVGLYANFLLFAAMGLVNLIFTYFCVIETKGKTLQMIQAELNGETLEKI |
| *BTST31* | MYLFKLNLGVERQLAAAFVASISIFTVGMLLGWPAPTLKLLRQPDSPLHLTPSEEALIVNALFFGTFLAPFPCGALMDHVGRKTSMLTLALFPILSWAVNGFAGMIFVYIVGPFVSLPTMAMIGGIFPVTFLVLFLHCPESPYYYIKRGLHADAGKALSWLRGGASIETELASIRTSIEKEAKADRGLVKMLRLITNPANRRAFIIVQGIACLHRSTGIPCIVAFSTVLLPSHIGALTNDNCTIIMGVALLSASLCCSAIVDTVGRKPLLYLSSIGMFTSMLPTAVWYYLDRETSTDVSGVNWIPLAGLLCFAVTFSVGLGPIMQIYAGEMFPTDLKGHACALATMNQAVSAVIVTQIFVALTVYVGLYANFVLFAAMALVNLGFTYFCVIETKGKTLQMIQAELNDFVQPLLLTRSTVRRSQ |
| *BTST32* | MTSFIYGTLVGWSAPTLKKLREPDSPIHLTPGEEVQMINAIYAGTLLGTFPCGALMNRVGRKGSLLLLSAFPITSWSAIYFARTASTLLIARFFAGVWGAAAMTIRPIYVAEIAEPRVRGAAGAFTMVAMFAGTIFVFVVGPCVSIQTMAVINGVAPPVFFLLFSLCPESPYYYIMRGRHADAAKTLAWLRGGAPIESELTSIQTSIEREAKAGQGYFKKMLSLVTVPANRKAFFIVEVMNFLQRVCGLSCMAAYSTVVLPQRVGPFTADHCTLIIGIVWFLSSLGCSTLVDKLGRKPLLYVSSIGILASMLPTSAWYYLDKETATDVTWINWVPLAGFLLFGVTVNVGLGSIAPTYMGEMFPSNLKAEASALTIMAVSVSSGVSIAVFALLTVHVGLYANFLVFAAVGVVNWVFTYFCVIETKGKSLQLIQDELHGGTRWKPPKNNHSDGKLTV |
| *BTST33* | MLNFIVVSGIERQLAAALISGFSCFIAGALTGWPAPTLKKLREPDSAIRLTPSEEAWVVNALHITTILSTLPLGSLMNTLGRKTTMLVLCVSPIVSWVLVYFARTSFVFIVARSIGGLWLGGCQTLLPIYIAEIAEPRVRGIAGSFIMVNAFAGIIFVFTIGPYVSVPLMAVINAACPCVFFLLFLFCPESPYFYVMRGRYEAAGRALTWLRGGAPIDGELNIIQTSIENEAKEGQGFYKRMLLLITNPANRKAFIIVEVMTFLQRFSGLSVLNSFSTVILPERTGLLTADHCTLIMGIVWLLASMCCSALIDKLGRKPLLHISSIGIFASMIPTAVWYYLDRETSTDVTRVNWVPFAGFLVFGFTVSIGIGPIAAMYPGEMFPSHLKAQASALSNMVSSISATLSTVMFVTINAHIGLYANFLVFAAVGIVNLVFTCFCVIETKGKSLQMIQEKLKHGTWKESGVNRETPETH |
| *BTST34* | MLNFITGGIQRQLAASLIAGFSCFIGGSLMGWPAPTLKKFREPNSAVRMSPSEEAWMVNALYIMSMLCTLPIGAVMNRIGRRTTMLVLCASPTISWIMIYFARTSFVLIAARAIAGFWLGGCLTVLPIYIAEIAEPRVRGIAGCFMMLNAMVGMLSAFAIGPLLSVLTVAVINLVYPILFFALFLFCPESPYFYAMRGRHAAAGRALAWLRGGAPIEGELTIIQTSIEDEAKAGQGYVTRMLLFITNAANRKAFFIVEVMNFLQRFSGLSALAAFSTVILPNRIGPLTADHCTFFMGVNWLVASVCCTCLIDKVGRKPLLYLSGIGIFASMFPTAVWYYLDRETSTDVTRVNWVPFAGFLLFGFTMDIGLGCIAPIYTGEMFPSNLKAQAAALSNMVASISSTLSTALFVVISEKVGLYANFLVFASVGIVVFLFTYFCVIETKGKSLQMIQEELHCKSRKKSETDREKSVTS |
| *BTST35* | MLTLTFSGTQRQLLAAFISTISLFMLGSMMGWPAPTLKLLREPDSSLHLTPSEEAWVVNALYFTTILSPLPSGALMNAIGRKATMLALCVFPTASWALIYFGRTASVLLAARVLAGFWVGGCQTIMPIYIGEIAEPRVRGIAGTSIMVNAFLGTIFVFIVGPYVSVPTMAVMNGVIPPVFFLLFSFCPESPYYYVMRGRHADAARTLAWLRGGAPIESELTSIQTSIQTCIEREARAGQGYFNKMLSIVTVPANRKAFFIVEFMNFLQSAFSTVILPAHAGPLTADQCTLLLGAAWLISSLCCSALIDRLGRKPLLYFSSLGILVSMLPTAVWYYLDRETSTDVREVEWVPFAGFLLFGLTFSAGLGSIGPAIAGEMFPSHLKGQASALTTITAAASSTLSIALFSALDARVGMYANFLIFAAVGPVSGVFTATSGDYREAVEFQRASVLRACLNGDFSGKFEVMRRSRNNRKLPYSASGAAGSSPPGKSPAEKRSRNMTEILHYLSEHR |
| *BTST36* | MSFRRVHGVRKLQPAISRDDAAESEHYGKMVFACGGAIMIFFFNGVVEAHSAVLLPCLQEPDSPIQITKDQETWIASLGIFAAPLSAILCGPFVDYFGRKVVIQCYFLTSALGYGIIAAATSVIHLYIGRILCSLGVGFEVAGIVYIAEVCTKRQRSLCMSLSYSTFTAGILFTYVVGAALPWNLGSALYALLCLLLFLYEWFTPESPPWLVKKGRSDRAVAELQRLGRTETAIAEEIKVLRLTCQEESNQRVEWHTFLQPTVWKPFLIIALFHFLQAATGMYDLLYYTVDFIDQLRTDYDSFKVSMGLAIGRFLMTSTVGSFFTTKVPRKLATAISGFSMGGTLLVAAYYEYLFDGVAPGQRPYTWVPILAVFASVMVSCAGVLHLPWMMSGEVFPLNVRGAMGGAVFFVGSWAMFVFLKYYIFFMETFKVTGTLLLCAAASIVTGLFGVFVLTETQNKTLQEVEDSYRRKPRKEIDVEKTGL |
| *BTST37* | MFLTPGVRRQLAAALTCSLCCLITELMVGWAAPSLKKLREPDSPVHLTRHQEAWVVNAMYYGNIVSPLPSGFLINLIGRKTTLLIVAVLPTVGWILIYFSTSATMLMVARFLYGLWTGVIMTTLPIYTGEISEPHVRGVFGSFFQICNSVGSNLSFLVAPYVSIQTMAVLCGSVPIMFIVLFSQCPESPYYYVMKKRPDAAARSLFWLRGGKPVSEELQVIQTSVANEEKHGSRCINLSVPATRKAFVIVVTMNILQRLSGISFMKAFSSVVMPRVGILSPDHCTIIMGVVWTVSSFICTALIDRAGRKPLLYASSLGIFVSMLWTSVWFYLNDKTDVDVSHLSWLPLAGVLVYGCTFSFGLGPITKLYPGEMFPSDVKGQAAALTVMIAAFSSSVSTGLAPILNEHFGVYSNFLIFSLIGLINLLFTYFCVVETKGKSLQLIQAQLRGERLDEIRDSDASEKHA |
| *BTST38* | MCLALGIKRQLIAAVACALATLVGGMINGWPAPTLKKLRQPGSPIHLTTEQETWLVTALHVGTLLSPFPAGFMMNKLGRKASLLALGVLPVISFGLIYLSTTPEMLILARLFAGLWIGGSHTVVPIYIAEISEPEVRGILSALNQVLSFLGNILIYAVGPYVSIRSTAVFCGAVAVIFLVLFASCPESPYFHIMRGHPERAVTSLIWLRGGPPTPAELGAIRGYLAARGGHGWRRITDLLTTPENRKAFVIVEVLSGLDRLSGISCMKAFSSVVLPAHLGPLTSDHCTLIVSLVWMASSMVCTALIDKTGRKPLLYISSLGVAVSMFWTGMWYFLSSQTHIDVSSFSWLPLAGFLVYGCAFSVGWAPITHTFQGELFPSHLKEQASAMTTIVTALTASFSTFVFGMVTKRVGVYANFLWFSVVGVVNLIFVYVYVIETKGRTLEEIQAELKGKKREEPVVRASTDSIFYFN |
| *BTST39* | MIATLKLIKEKIFNKTLLAVIIVNSINIATGMGQGFSAILLPQLENSKEFFISQEEKSWLASLGVILNPVGAMAAGVIMQFAGRKYTLIGACIPFFFGWLIIAMSTSLAMLYVGRLLSGLGMGMASAAYVYISEISTTHERGLYSSFGPTGTSFGVLTVYFLGYVADWKTVAWICAATCALNALSICFMPETPSWLVSRQRLPDALRSLVWLRRNELVAKKELNDIVSHAVLESQVTRKQRTILSILRKATVWKPTVILVAFFILQQGSGIYIMLFYSVTVFQEIQSVLNPFVDSIIVSVVRLLTCIIGSAFIQVISRKRLVILSSFGMFFSMLSLFAYGNIASDDATARLVPWFPEMCLLINISFSMFGTLQLPWIMIGELYPLAYRGIMGGLISSVGYALIFLHVKIFPAISTVMNIYSIFLVYGLFSLVAIVFGKVYLPETKDKELHEIEEIFKKKKGEVQKVDEKPNQTPIFICNPKLPFVPMQEQVVLSAPQV |
| *BTST40* | MSQGDKMERGQESITMIPRGDVREEGKKLPQYIAAITATLGAVAIGTVLGWSSSASPFLKGEITNVTSTIDPPLSVDESARVESFVAIGAIMGALPAGYFADLLGRKTLIAALTLPFLLSWIMILLAKVAWLLYVARILAGIATGATCTVVPMYISEIAELSIRGTLGAYFQLMITLGIFYAYVYGYLVRFAVLNILCALIPIAGFFMFMFVPESPKYLLMRQKKQSAEKSLRWLRGNKYNIKQEIETLQNEIAKSSRTKVSFKDLVATKVAFKSVNIALGLMVFQQLSGVNAVIFNMNAIFMASGSTIEPAICSIIIGAIQVIVTFFSSILIDKAGRKILLLISLGVSTLSLGVLGYYFHLKNSGEDVSGIGFIPLICLILFIVVFSLGLGPIPWMMSGEILAAEIKGLASSLATALNWTLTFVVTRSYAPMEKTLGTDVTFWLFACICAIGFVFVVLIVPETKGKTVDQVQQLLAGKKPARKNGLV |
| *BTST41* | MSTPEKQPPPSYQSSEKGRFHYSKKSTYAQVLVALLQNWLLLDFGMMLAMPAIVLGSLHNHPSEELCLNDAQASWFGSILFFSHPLGSLCSGFFQEQFGRKGSMMLVNIPIFIAWITLYFAESVYALYFVALTMGLGIGFDEAPLHSYIGEMGEPHLRGTLCALLCAASFFGTLVMYFIGYLVPWRTAALISSAVPIITVVCMSQIPESPTWLVMQGRLKDAQKSLCWLRGWTEPEVIRAEFEQLCSYAKISPNPDQEKNPEKEKYELVPGNDNEKVSPEEEEESYLKTKFKELTDKKLLLPLRMVFIVFIFCHATQLTAMRPYMVGILDEFGFPVDSKLVLIVTGASVFAGTTMNMLFLRKFGKRKMTLTSQGMAALCILLLGVYCSFYDRSNRILSLAWMPVALMLLASFFGGLSLALLPWQLLSEVFPLKGRGAAGGISAAWAYYVSFAMSKTYLYLEHWIKLNGVFFFYGGITLCGFLYFLRNLPETEGKSLEQIESYFTKNYDRREMFSKPKRSKKVPFSA |
| *BTST42* | MCIPEEPSRQSREMGPFNYDRRSTYAQVLVALLQTPMIFNFGMMLSMPTIVLGALHNRKSEALWLDDDQASWFGSILFATHPLGSVCSGFFQTYLGRKGSMLLVNILFFIAWITLYSAESPLVLCIAALTMGLGIGFDEAPLHSYIGEMSEPHLRATLCALLVSCSWAGALLMYLIAYLAPWRTVALISSAVPIFTIICISQIPESPTWYVMKGRLNDAQKSLCWLRGWTEPDVTRTEFEQLCGHIKKSSNSDQEKPQAEEKYQAVPGNDNEKESVGKAESLFKTKFRELTDKKLLLPLRMVFIVFIFSRATQLTPMGPYMIGILDDFGFPADSKLILFVAGLSGFAGTLMNILLVRKLGKRKISLTSQGTVAFCMLLLGVYCSFFDRSNRILSLTWIPISLLVIAGFFAGLSMALLPWQLLSEVFPLKGRGVGGGVSAAYAYYVSFAMTKTYLYLEHWIQLNGVFFFYGGITLYGVWYLLRYLPETEGKSLEQIETYFTKNYDKKEMFSKPRGSAA |
| *BTST43* | MAIDKGKLRQYLAGFIASIGSACFGVAMGWPAPVMWALRDPRGRIRMTAEESSWMVSIMELGNLLSPIPGGVLADRYGRKFVLHLAAPLFAASWIIVLLSKAKLMLYAMRVLQGLATGLVFTLTPMYLGEISKKEHRGTIGSMFSVMMYVGSLYAYVFGPPFSYDVFAMICLAMPTVMFFGFMFIPETPYFYLMVGDLKAARKSLAFFRSKDDPIEEELLLMQESVETDMANKSTFMDLMTEVGNRKALIILQVLSMFKIMTGICALLTYATMTFEETGTRCDANLVSISFAVVIVVSTIASAGFVELTYATMTFEETGTRCDANLISISFAVIIVVSTIASAGFVDRCGRRPMLLISSAGLTLTNVLIALYFYELRLLPPLSDYAFIVFAAVGALACFHTIGYGAVHSTIQCEYFPSNTRGLANGITAVTLTVFSFFTLKIFQSIDTYFGMYVNFIVFAAFCFSASVFVNEVVFETKGKSFSEIHAEFRNRVSEPYVDEHHVVTV |
| *BTST44* | MDTPTIEAQPVSVFTKLHFQKSESSVSTSKADGMKEIEAAKPEDSPREVFTLKDEEVDESKAKTGTQYLAAAIASLTGVMMGQMLSWSSPVTPLLIKEGKIDKIEESWLVSILNFGAVLGCTAAGSVNSYVGRKSVLLTACLPQMASWLLLALCSDIRLLCLGRFLGGLCVGFFCVTSPLYISEIAQVSVRGALGALFQLSVTIGILTTYTLGLLPTATSITLASSSTVVLFFALFFWMPETPVFLLRTSQSNRAATSLRWFRGPAYNLIPEMRLLERMVQKADSAAAYSDFVTDPASRRALVVALGLFFFQQFSGINAVVFYMNTVFRTAGGDVSPTVATIVIAALQVVGTALSVFLMERAGRRFLFLASFSACTLCVFALGLFFFLKERGHPVAGPWQWVPLGSVGLFLVVYALGAGPVPWAVVGELFSKRMAALAMSLVTGVGHWASAFVVTKAFAMLEAWLGIGGTFWVFVGFCLVGIVFAWALLPETKGRPLQEILDELGGKKKGVKSET |
| *BTST45* | MMAETNDNMDLPATNKVQANTTEDSTTNSRPPPCPQEVCDTEKGDGSRTGPQFLATGIVSLTGFLMGEMVSWSSPVTPLLIKSHRITKDEESWIVSTVNFGAIIGCLLAGYVNKYVGRKTVLLSLCVPEMISWLMLAFCEGAILLCLARFLSGLCLGFICVTTPLYIAEIAQPCVRGALATFFQLFIVIGILFTFILGILQDALWITLGCSIVVLVFFALFLWMPESPVYLTMVSRPKAAAASLQWLRGRDYDIYAEIRVIEAVVQEGRDVEVTYGDFISDAASFRGIIIAMGLFFFQQMCGINVVIFYMNTVFETAGSTISPTLATVIIGIVQVLATALSVYMMDKAGRRFLFIFSQAACSLCLISLGTYFFLKSRGDDVTPIGWLPVASVAVFLVMFAFGSGPVPWAITSEIFSKNIASLALSLVTAVHWLLAFFVTKVYTSLEAFMGTGPTFWMFAAWCWVGVTFCCLLMPETKGRPQEDIVDELRGCKKKQTSGNCP |
| *BTST46* | MAPQDTLFGSGEKKKEYDDSVDEVKSLDLETSDSEQSVNMDNMRTALPQVLATLAQSLLLLSLGMIIAVPTIVIGAIYKAKEGLSLDDDQSSWFCSILLIVQPIGSLLSGYVQEVVGRKISLVVVNIPQLVGWYLMYAATTVDMLYWSCVTLGFSIGFMEAPTLAYVGEISQPRLRGMLSCITNSHVPLGHLVEFFIGGYVAKDWRMAMAISAVFPIISILAISQVPESPVWLLTKGRKADAMKALCWLRGWTTPECVRDEFEGLVRYVEASRLQNENQQKAAGKNYVQVPTAGYVNADGKGTTPAKVPAENSYKFKVTIGEKIKDLLRPAMLRPLVLVVSYFFFYNCASLNAIRPYMVPVFQKLRLPKDPHFVAILSAALQVLGGLVCIATVHKLGKRCLSLISMTLCAVACILIGIYAVLIERTDFDCPWFPFIVLLALYFCCNVGISPIPWMLISEVFPSRGRGAGGGVSAALFYIILSIISKTYLDLESIVTFPGVFFVYGLVACAGVIFIFLCLPETEGKTLQEIEDYFTRSRKKGINNLSV |
| *BTST47* | MTEPDSTPPSSGCCRPFLVISSLFPLYVCVGALFGESAGMLPQLMEEDSLIPTSREEATWIASVPTIGTCIAATTSGSLSDVFGRIRMVQMAYFLLGMGYGIIGAANDFTLLVVGRFLAGVGVGCSFPANVYVSEMAPPAYRGLFLVLNPLLASTGLVYMYVVGVYLPWNIAALFSCLIAFLGLLLTFFCRDSPVWLLRKNRPDAARRSLAQIEGPANVDARLKQLQEIADAQRDAETQSGHKTFSLRVLASPTVWKPYLTVLILSALQNISGFYIVISYTVNFMREFHSTFDPLQATVAIGVVRLAAICVTSALLRHVGRRTIGAVSGFGAAASLLLVYWCLVKPELAPNAWTPMALFLAYIFTMTLGIFPLPWTMPYEMFPIKVRGTMCGVSFCSMYGLMFVSVKLYNTLLDNLRLEGMILLFAAGSLVFGLYSATLLVETHRKTLDEIEAVFAGKRTKKVDG |
| *BTST48* | MEDPYVPPRSDVKKISRFRQILPQIIATSATIVLYLTMGMIIGFPTILIPALTAKNSQDVLHLTMEQASWCGSVGCIFQPLGSIIAGLALQPLGCKKSMMLLNIPLIACWLIVHFATSNYALYFANGLFGCVMGLTTAPGLRYVIEISEPSLRGILVASTSLFISLGFSFIIFLNSLTDWRQTAAISASIPLLCIIILFQVPETPMWLVSKGRSDAALKSLRWLRGWTDAETVREEYEKIVSFTKNQNQKLLKRQWSGKVAEYKNCPTVEEAELAEPASASQGLSERVRKMFKDMTRKEMLIPLTKCCIIFAINCFSGVPILRTYMVKIFDDLNLPVDPKKASVWVALMGMLGNIGCMFVIKKLKKKPLFLASLAGSALCLFSMAADLMGYLEGTVIASFHRWCHLTFAMALYFFWNLGIQPIAWSYLGEILPYKGRGPATSVASSFYFILTFVGIRTFPAMTEFLRLEGVLLFYAVVCVAGLFFTHFLPETEGRHLSDIQAQDKEGAETEKL |
| *BTST49* | MSGNGKRQPVEKSFLGYPGDEVDRCKSTLSQFYATCVQCIFLISLGMQFVMPTIVLGALHNKAVINDAMYLDDADASWIGSTLYICHPIGSLISGFLSERFGRKGGMMLVNIPFIGGWVLLYCATSVRGLYVATLTMGLGMGFCEAPIAAYLGETSEPRLRSIFTTMTTAACNLGVLIELAIGSSLDWRTSTLVSSFVPVFSFVLFFTIPESPVWLITKGRMKDAQKSLAWLRGFAKPHQVQNEFDELVRYTKMSYALGNSQGNPEEKAPLDNKNNTSKHVEEDSDGWLKTRYKEITNPKLYMPLKFVMFSFFWAQCACLIPFRSYMIGILENFWFPVDRKWILIMTGVVAFIGSVVPMFIIQYTGKRKLGLSCMFVATASILCLGVFASFYTHTENLVVAWLLIVDLAVVHFVGFLGIINVSWMLVCEIFPVRARGIATGISTGWSCFIAFLLTKGFLWMESMVGLSGLFYMYGVLSFLGCIYYYFNLPETEGKTLERIETYFTSNHDKKEKYSMPSRSASKA |
| *BTST50* | MIQDSTQLVSAPNSPFFTPTRIKQGWTSLLMFCAFSTIMGLAIPMGYGIGVINTPADVIRAWCNETLQANYDVVLTDKKDLDLIWSVIVSVFLLSGVVGSFIGGWLANLIGRKGAMLVSCLLSTVAGFCFLSPLIVNRIELLFAGRVIVGLSAGLGTAVVPMYLLEIAPTKLQGSIATFFSLGITIGVLLGQILGLNWLLGGETRWPYLLSAYILCVLFCLLTFPCLPESPKYLFSVKNERQSALQALSRLRGLPADLLQSELDSKDTAENNFNNEEIQTWSVAQVLRTRSLLLPLALVIALQAGQQFAGINAVFFYSSDIFKSAGLDETSREYAVIGTGCVNLGVNVIAVFTLKYFTRRFLVLLSCYGTVLSLLLLTLCSHYMQTVSWLPNASIAVVMLYVFMYGVGLGPIPYFIGSELFAVGPRPIAMAFGSFANWGGNFLVSLTFTTFFNYLAGYSFLIFAGSTMLLSIFIHAYLPETKNSVASL |
| *BTST51* | MVVATGDFVSTSRDSFIRPLLASVPVFWLQLLTGSIEGHSAVLLPQLEESEKFYISLEEESWIASLGIMATPLVAVLSGPMVERYGRKFIFYIFYILCTLGFSFIGLAQRVEHIYIGRILGAGAHGLTLCSILYIYEICVAHQRNKLLPLLCPMCSAGILYAYVIGGYLPWNVASLVLAGSGVVGLICIFFIPESPAWLVMQGDINAAIQSLEWLKRDKETIAQEVDELRKSSTSKDLPQSISLQHFLHPTVWKPFLILLIFSALQNGSGFYMLLYYTVNFFQNLGTGGEIDPLTITVGLALMRLVSGSFGALFIARFSRKKLTATTAFGMFIVASAAVAYLVTFGEDPTNRPHQWFLVLCSLSYVLLCTLAIQPLPWLMTNELYPLQLRGLMSGITFFCLFTMVFVGIKAYPFFMFYIHITGILCIFAGACLLAVIFAVFFLPETYNKTPYEIEEYFMRRKKKSVHCVNFYEKTCVQIPLKFKEFASYYGEHSLFARKSA |
| *BTST52* | MMAIKKELNLDNPRESYVRPIAAGAILGMQLINGIMEAQSAVMLPQLAAESSAISITQSQSSWIASLGIVASPISSVLCGPLMDFFGRKMILEGYYVIAMLGFLIIACAKKVVHLYIGRFALSIANGFGVGTIVYLPEMCSRDQRSRLAALLMPLFSSGILTAFLVGGYLPWNVASASYTGVCTIGLICSVVWTPESPCWLVNEGRYEEAKRSLRYLRGSDSTNIEAHVEVLKMSRKSVLVERNAVFSDLLEPTVWKPFVILVLFHFLQTGTGFYGLMYYTVDFFDDLRTSFDPLTVTIFLSVARLVMSCVFGTYCATRLNRKVVTALSSGLSGVSLLGAAAYEHVFTSIDADERLHTWIPIGCILTNVLVCTITVQPLPWLMTRELFPLPVRGIMCGLTYFIGTVLVFLSVKYFMSVMGLFGIPGALSFFSASSFLVCLFGIFVLPDTNNKNRSEIERNFTKAKPRVEEQPLIGKGVGNVMPTLLV |
| *BTST53* | MVFACGGAIMIFFFNGVVEAHSAVLLPCLQEPDSPIQITKDQETWIASLGIFAAPLSAILCGPFVDYFGRKVVIQCYFLTSALGYGIIAAATSVIHLYIGRILCSLGVGFEVAGIVYIAEVCTKRQRSLCMSLSYSTFTAGILFTYVVGAALPWNLGSALYALLCLLLFLYEWFTPESPPWLVKKGRSDRAVAELQRLGRTETAIAEEIKVLRLTCQEESNQRVEWHTFLQPTVWKPFLIIALFHFLQAATGMYDLLYYTVDFIDQLRTDYDSFKVSMGLAIGRFLMTSTVGSFFTTKVPRKLATAISGFSMGGTLLVAAYYEYLFDGVAPGQRPYTWVPILAGRAPPPLDDERRGLPAQRPRGHGRRRLLRRLLGHVRLPQVLHLLHGDVQGHGNAPLVRRRVHRHRSLRGLRPHGDAEQDAPGSGG |
| *BTST54* | METFKEQKSPPPENWIKTFWACGGAITILVFNGVVEAHSAVLLPQLQEAYSPIHVDKDEETWIASLGISASPLSAVLCGPCIDRYGRKIVIQGYFLISAIGYGIIAAASSVVHLYIGRIICSLGVGFEVAGVIYIAEVCTKYQRSLFLSLTLPMFTGGILFTYVVGATLPWTMGSSLYALLCLLLFVYESFTPESPPWLVKQGKISRAKAEFKRLGRSDEWIEEELKLLQASCDTLERNHHLDCKTWLEPTVWKPFLIIALFHFLQAATGVYDLLYYTVDFVGELGTQYDPFQVSLYLAIARFLMTSTVGLYFTSKVRRKTATALSGFAMGASLLVAAIYEQRFDGVAPAERAHTWIPILAVSVSVLVSCAGVLHLPWLMSGEVFPLRVRGLMSGYVFFVGSCSMFVFLKSYVFFVEVFKVTGVLLLCAAASVLIALFGLFVLTETQDKSLYEIERGYEKKSRGDADKTNLREVE |
| *BTST55* | MKLMIGDEEVCVKIGNDGKEELSWRCWLRTMFAASGAMAVFVFTGVTEAQSAVMLPQLKQKDSPIQISADEETWIASLGILLTPVSAILAGPLVDAFGRKKGLQGFYIIIGLGFGVIASAKEVYQIYIGRCICAFAVGMEPIAVIYLAEISTKRQRSLFFSLMAAMYSGGVTITYVIGGFLPWNVASAIFSLGCFAIFVVQCVTPETPAWLYKTNQVEASTESYLRLGRSHTNILQELESLGLSSQQRTEKFHIRAFLEPTVWKPFLILSLFHIIHCGAGIYDVLFYTVEFVETLGTSYDPLAVSIFTSVARFITNMTVGLYFTASLSRRFATIFSSFFMCLSLFVMGVYEYLYRDVSVKPFDWVPVLFTVVSVVSCSTGLLSLPWLMPGEMFPLHVRGVMNGAAFLVGSACMFVTLKLYAFCMETLQIWGMLLMFAGFAFTGIFFGMFVLPETQGKTLYEIEQGFLPTQKKRENEISPSGKVETIT |
| *BTST56* | MAFVVDTEVACAKIQSSDEDESEKLSWRCWFRTLFAASGPLMVFLYTGVAEAHSAVLLHQLKKEDSQIPVTTDEATWIASLGILLAPISALLAGPVLDAFGRKKGLLSFFLSMGLGFCVVAFAEEVYHIYIGRCICAIAIGLEVTSVVYLAEICTKRQRSCFLSLTAPFFSLGVALVYLVGGYLPWQMAATIFSLSSFGFFVIQCFAPESPAWLFKTGQIEASTKSLRRLGRSHENILHELDLLTLSTRTRSGKFHLRAFLEPTVWKPFLILSIFHFITNAAGVFDVLYYTVDFVKAFGLTVDPLIFTVLLAVARFTTNCTLGAYFLVSVPRKFTTAFSGFIMAASLLGSTVYEYAYRGLAEKPLQWIPVTLTVIAIVASAMGMNFLPWIMPGEMFPLQVRGAMTGASFLVGTFCTFVSLKIYGFYVETFCIWGLLLRFALFAFAGALFGILVLPETQNKTLYEIEQGFVAGNKKIPEAPEQ |
| *BTST57* | MTSSVSDEETRGKISSNEETKDKTVSDTEIGAKPVNDEECGEEVSWRCWIRTLFAASGAMMVFVFTGVTEAQSAVMLPQLKKPDSYIRVGPDEETWIASLGILLAPPSGILVGPVIDAFGRKKGLLFFFLCMGLGFAVIACATEVYHIYIGRCICAFAVGLEVVAVVYLAEISTKRQRSGFFSMMSVVFSGGVTLTYLIGGYLPWYIASAIFSAGCFAYFAVVCFAPESPAWLFKTGQIDASTKSFLRLGRSHVGIVAELENLKLSSKEDDEKLEFKAFLEPTVWKPFVILSMYHIFQCGTGVYDILYYTVDFVESLGTSYDPLPVSILLSVARFVTTATLGIYFTASVSRRFATAFSAFWMAVTLAGTGVYTYVYRDTTQKPYDWFPIVCMLINIVASALGVTSLPLLMSGEVFPLRVRGAMTGASFLIGLGALFVVVKIYAFCLQILQIWGLLFVYAVFSVLCVLLGVFLLPETQGKTLWEIEQGFLPKKERRRNGERRTEDTLGSGVIRK |
| *BTST58* | MGKVEMHSSEKVYRPCEMTTSNVNNVKSTVAQFYATFVECCFLILVGMIYMMPTIVVGALHKTESSNSTLTSPEDEAMRMDDHTASWIGSIVLMSHPVGALTSGFVSERFGRRGAMMLGNVPFLGCWVLYYLATSVKGLFIASMLMGFTIGLCEAPMGAYLSESCEPRFRGISNSMVVAFCTMGNSLELFLGSAFHWRTSALVGVSVPVICFLGFLTVPESPVWLITKGRLEEAHKALAWFRGFAEPQHVREEFDDMVRYSMASSRLSRHDLSAISEEKAPLDGKHNIQDPEKSQSIKRGNWLVERWRELSNPRLYLPLRMVLITFFFTQSAGLVPFKAFIIEILNEFWFPFDNKWAVVATGVASFLGSVGATILVKLAGKRLMCIICMIISTISIFVLGFSASFFRHQEDLLLSWLILSVFAVVHFVGNVGVINIPWMLSYEVYPVRARGMANGISAASGCFMAFLQTKTYLDTERAIGLDGVFYAYGVVALAGCIYVILYIPETEGKSMEQIETYFTPHHDRKEKYRMPSKKNRSKA |
| *BTST59* | MSEKEKLHSTEKVYKPCEYSTENTGNFKSNLAQFYVTCVECIFLISLGMQYVMPTIVVGALHNKVGDSMALDDTTASWIGSILYFCQPLGSVTSGFLSERFGRKGAMMLVNVPFVAGWILLYYATSVQGLCIATLTMGLGIGFCEAPIAAYIGEVSQPHLRGIFTAMTTAACQLGNLIELFVGSVFDWRTSALISTVIPLISLISFTTIPESPVWLITKGKMEEAQKALGWLRGFLEPHHVQKEFDEMVRYARMSNTLSSEPGENFSEKIPLDSEKIPIKEVSDGFFKQRYRELTNPKLFLPLRMIFITFFFTQTASLAPFRAYFVRILDQFWFPIQSRWVLVMTGATAFIGSVTAIFILQKTGKRRVMLFSMAVNLLATFILAIYATFFTHTVDMTVSWILIVTFGISYFVGSLGINNIPWMMLCEVFPVRARGIASGLSAAWSYFVQFVMTKTFLQTESLIGLSGMFYMYALISVGACIYTYLCVPETEGKSLELIETYFTKNCDRKQKFRMLKRGRNPSKA |
| *BTST60* | MISSGKTQSPNERPYQYEYTALGTQDVEKSGEHGTGVSGNRRKVNRFRSAAPQILAVTAKNLVLLDLGMTMAFSTIVVPVLLDPNNKDPNGLSFTEDQATWFASIPMVFQPLGSALSGLISAPLGRKRSLMLVNIPQIIGWLMLYSSSSVNIMYLAAAIQGLGAGFMDAPIFTYVGEICEPSLRGVLISYSLQFCSVGFFLQCLLGSLTTWRHVAFISMLFPTLAFLAISQIPETPMWLLSKNRMKEAEKALCWLRGWVSKEEVAEEFAQLVQYSKNSKYKSDDDKKKLQMDLISTAKQPCGGCTRPPIVPCDSNTGDDDYAKLKLHEKVKDLLRPEILKPMSIIIIVNFLYFTSGFPGFKTYMVLLFQRVHSPIDPNWASVFVSTSIILIHIAQMVAVKTIGKRWMTLISSFGAAVAGLAIGVHMSFQGFFDETFGDLSNWLLFTYFEILTLATVIGLGPVPWMLMSEIFPFRGRSFASGFCAAIYYAASFFAAKTYLSTLNLFGVAGTYYIFGTISALGLVYVYLYLPETEGLTLEEVEDIYRPKKRSEVKNL |
| *BTST61* | MSDTTPSREAPAPTMTTESPANSLDVYYIPNINNEEKNYKSNCKSTLSQVIATLVESLLMVVLGTQSVMPTIVLGALRNNPHETLSLNDYDAAWLGSILFLCQPFGSVASGFLSEKFGRRGSMTLINVPFIVGWILLYYASSVTGLFAAVLVMGIGIGFCEAPIAAYLGEIGEPHLRGSLLCIMCSAVSLGYLSTFFLGSIMPWRTFALVNVIYPVTTMILFTQIPESPIWLIHKGRLKEAQKALGWLRGFVEPRRVQQEFDRMVKHIEASKSSDRPKGNVESQDDGSCESKFTVIKRICELRNKKLYLPLRLVFITFIFTQCMCLQAFKPYLVNILDTFKFPVDSKWVLVMIGLMNFVGSAMPLFIFRFTGKRQLILCNQFICVVGVFALGLYCSFLNDTLDTNSDWRWLPIVLFAIVFFSASTGIMNIPWMLMGEVFPIQYRSFANGLCGAWAYCVTFVTARLYLPMEHVLSLSGMFYLYGIVGILGFFYFLFFLPETEGKTLEKIESYFTPHHDKKEKFTRPKR |
| *BTST62* | MAKNEDEPPTGGFLRPFLIMTALAPIQLVVGSVLGQSAGMIPQLMQEDSVIKIDIDVATWIASMSTVGTFVAASSSGFFADKFGRIRMVQVAYFFLAIGYGIIGAANTFFLLIFGRRLIGFGVGCSFPASVYISEIAPPAYRGLLLTTNPAIASLGLVYMYVLGGYYPWNIASLATGLMSILGLIIAFFFYDSPVWLLRKNRIEDARKSLSRIEGPTNVDVKLKQLQEIVDSHPVSKFSPHVFIEPTVWKPYVITIILSILQNTAGFYIVVSYTVNFMREFHSTYDPLQIMVAIGLVRLVAICLSSAILRHVGRKTIGAFSGFAAAACLLPIYGCLVAPHAAVLRTYPWIPIALFLGYIFTMTLGIFALPWTMPYEMFPIKVRGFMCGVSFCSMYALIFVAVKLYNFLLENLQLPGMILMFAVGSLLFGVFSATVLVETHKKTLDEIEEVFLGRRSKKIQKTTSDS |
| *BTST63* | MFSVSILPFGFGQFATWPSLAIEQLLEGDAGFSVDQSEISIIASTWSLGLCLLPILFGFMLVRQGRRRNLLITAVVYIVAWALILFARSPLWLMAGNFIGGLGSSIQLIIGPIFIAEIADKYIRGALISFYIAAIPLGQAFMCSVGIYVTYFQLNLIALVISTVAFFCILATAVESPSWKLMKQKESEAESCFNYYWNTRNADRTESTVALAELRETVELEMRSKCSYSELVRTPSNVRGTIIVAAISLFQSASGILVILDYGSTTLPKYEGFWAPHPTMAAVSILYFFLSLVSAGLVDRLGRKPLTILSNAGDALGTAIVAVFFALERRTEWDTTNLQWLPYVGMLLFIASYGSAMSAMPHVLVGELFPANVRYHASVLSVIAIAGSLAFFNYTYLGGCRLLGMDVMFFIYTLCSIAATIFSWLFMFETKNLSLAEIQAIMTGRKMASNDPPQELNDLR |
| *BTST64* | MVQLAWLSRNRGRVRQISVCCSASILPFGHGLLVAWPSLAIERLRRGDAGFEVSSGEISIIVSMMSLGMCLMPIPFGYVLVRLGRKTNLLINAVVYAVAWGLIAFAPSPLWIMVGNFFAGLGSSIQLIIGPLYIAEVADKDIRGALISIYIMAIAIGQVFMTSIGIFVSYFELNLMSLIIAIVAFFCILTTAVESTSWYLMVDNEYEAERSFHYYWNTGAGTDRTQSLATLKETVALEMKSACSYVELFRTPSNIRASIIVIAQSVFQSAGGIVAILTYGSTTLPAYDGFWKPNPTMALVSVLNLVFNIVSAGFVDRIGRKPLTIISNAGNALGTAMVAAYFAVERRTNWNVTDLEWLPYVGFLTYVAFYGSGMFTVPHILVGELFPVNVRYHAAVLSTISIAGSCAFFNYIYLGVSQVAGVDVMFLIFTLCSISATVFSWIFMFETKNLSLADIQAKMTRNSTRASDPTPELENRR |
| *BTST65* | MVKDEIEIELSKEYDDSVRCPVNRLGKFRQCLATFIANIITICLGTVNGWAAPVQPQLQSETPPVGRRLSDDEISWLGAITFMGGVAGVLVWARAADLLGRKGAGYLIAAPFLLSWTLLLFCDHYYLLLAARFIAGFGGTGVLVNTPLYVGEIACAQLRGPLGSSLILFINFGYLLAYFFGSVLTYARFNLFCLLLPVVYLALFAYLPETPNYLYMNRREDEAKRSLLYFCGDNARAMNHEFNLIASVTNGGPRVELSDFLRKKSTRRALVIGMVLITGQQVVGINILLTYTVAIFSAAGSAISPNLCSVIVGVAMLIASIPSCYLINRLGRKYLLIFTSTGMSASLLLLAVCFLFDKSNAFVQSTYLPLVSFSTAIVCYALGVGPVPFVLSSEIFPSSVRNMATSLIIAWGIFGSFATVKLYPSMLSLLGYFGTFSLFSVSALCLSLFIHFCVPETKNLSLNAVIELLENHSTLKVGRFS |
| *BTST66* | MASLGIVTAPIGAILIGPFVDAFGRKVGILIFYLTIGSGFGVIALSMDVTQIYIGRIICAFCEGFKACAVVYIAEICTPTQRSLFLSAISTMFSGGVLICTVMSAFVSWNSACLAYSLAAFAFAGVQWFVPESPGWLYRHGKEDEALRSLERLGRSKADILREMDDLKERKSNQEKLELKSFFEPIVWKPFVILSTFHVLQFSTGIYDIIYYQVDFIQSLGTTYDPMTVSVAMSTIRFLSNATIGVYAKSVSRKGSTALCGLGMALTLLATGAYELAYRDTEIPARPYQWLPISLILSCIVASNLSVTCLPWAMSGEMYPLRVRGIMSGATLVVAYFAFFFYIKMYYVFLEALKIYGVLFVFAACSVVVLLFGIFVLPETQGKSLLEVELGFEKKAKRSENVENRNGRVEKGEKSDFVTRF |
| *BTST67* | MLIEDLHVECIIAADVTFSRVKLIVRLSSNYNIECLVEKEYLAGATRQFLILSSISPQNRKLRVQAAPLKTIDDMAGEPEIITWSCWLRTVVAGITALFLLAFAGMNNGASNLLLSQLTKKDSLIPISQDQESWVASLGLLAAPIAPILIGPFIDFFGRKKGVLVFYLIMGIGWAVIGSAKNVTQLYIGRMICSFGEGFEACAVVYLAEICATEQRSIVLAWLRALFSAGVLFVDVINTCVSWPVACLGFSLAAFAFAIAELFVPESPAWLFRQGEEEAAVKNLQRLGRSQAGVHLEIETLRQRESSTESLSWRTFLKPTVWKPFVILAVFHVLQLSTGCDVIIFYQVDFLASLGTTYDPVSVSVALSTVRFLSNITVGVYTNSISRKISTAISGFCMAVPLAGAVIYEYHYRSVPVLDRPVQWLLLTFIFAYLVAAELAVNCLPGTMVGELFPLSVRGTMSGATHFAAHCSYFAYVKFYFACLRVLKIHGILFVFAASSFLAGLFGIYILPETHGKSLVEVEQGFEGKTQEIDQNIVVPLSSINS |
| *BTST68* | MITVASAVMENDTNSCRKASVENGLKTDISNNRRGKRRFRSASAQILACVIQAWLLVDLGMEMATPTLIIGALHKISAEAEPLHMNDEEASWFGSISNMVFLFASLSSGFLQELIGRKGSMIVVNVPRFAGWMTLYFASSLSTMYLAAVVMGICEGLCEASVHSYIGEIGDPRLRGTLASISSHGYFFGTLTTLILGCYFEWRTVVLISSAVPVLAFICLTQIPESPTWLIVRNRLDEAKKSLCWVRGWVSPDEVEEEFQEMVNYVKNSSEESLKAFNSNECAESNAKDLTVFKGVLSTMKAVASKKVLRPLCMVCTAFLTSLAGNVIGITPYMIRELRELGAIVEPKLILVMFQIIFVVGSLTNVAFVRRFGKRRLALLSQGLAVLCILGIGTFCSLAFSSADRSPQLSWIPVALFFFLNFINGVGVRLLPWQLLSEVFPPVGRGFASAISVAFAKLILFTLIKTFLMTEDWLHLSGVMYLYAGVSFFGLCYYYLYLPETEGKTLEQIESYFTKNHDRTEKFRIGNQDRNTLY |
| *BTST69* | MGASMNACTRTPTEDASKTTAKRHPCRSTCAQLLATLIQGWLFLDLGLEAAVPTLIIGALHRNPSATESLRMNNDQASWYGSLQSFCFPIASLSSAFLQELIGRRGCMMAVNVPSFAAWMTLYFAESVPALYVASAIMGLSSGISEASLHSYIGEIGEPRLRGTLSSLSSSGFCAGSLGGFILGYYFDWSDWRTVVLISSACPVIAFICMTQIPESPTWLIVRNRMDEAKKSLCWLRGWVSPNEIEEEFQTLVQYVKNSSAETRRGLRQETSIASTEENSSILKEFILIIKVLASKKVFRPLRMVFITFVISSVACVGGIRPFLIGELKDLGTTIDPKLVLIMFEVIFFVGSMFNVTFVHRFGKRRLAIYSHSFAAIMITGMGVYCSYSSFYEDNSNSQLPWIPVALFAILNLVEGVGISLLPWQLTCEVFPPVGRGLAAGMSAAWSKLVFSALIKSFLYLEVWLNLSGVMYLYAGLTVFVSLLLSTRNGGEKSGADRIILHESSHPEGEN |
| *BTST70* | MTETEKNETSGQEEAPSEPVISNTTYRQIVLALILAIPSIAPGMTFGYSAVSLDSIPANLSQESWFASLAWIATPVGCLASGPIMDNWGRRPALLLINIVGFCGWILLAYASTTLSLYTGRILTGASIGFASAPSSVYVAECIASNSLQLRGILLTWPTVALSTGILLVYIMGSLLRFTVVAGLGAIISVASFFCILFFIPESPAWLLLKGRREDAEVAQRRLGLGKPLSESRVESGEASTSTKLLPSQSELTWSTAWEELKKPEAYKPLTIVIFFFLFQQFSGVLVVINYLVEIVRISGFVLLNPYFVTVVAGFIILICACSVSFLLPKFGVKGLSTISGVGIAISWLIIGLYIFIRRTWLVELQYSLFNLIPLCGIILNVVSSSIGFYPLPFAILGEIFPPKIKGVASGIATCVAYLFSFIAVKTFIYLQLHFYSAVIFFYAVMAAFGVIHVNLFLPETTGKSLQEIVKHFSATKSGYEKI |
| *BTST71* | MGAEDAASAESFLKPFLAALASFMCQFQLGAILGQSSTMLPQLQAEDSPIRITKEYASWIASAGVIGTPIASVLAGPLTDKMGRKSVIRMHFLLSAIGHTIVGVSSDGTEILIGRVILSCATGFGVPSLVYIPEICNPRHRSPLLFTATVSSSLGLVYVYTLGGILSWDITAMLTSSLAIIGLVYTFIVPESPAWLFRSHRLNEAIDSIKWLKGQNVNMELELRSLKDACHEQPKERVSLLKQFASPTVIKPFLVLTIISFLQNASGFYILLYYSIDFFLEFKSSYDPRFVSVGLAVTRLVSCTVASVIINRFCRKTMGTFSGLSMGVILLGILGYLHAFGDDVEVLSRYSWVPAAGLTLYVFACSLGVHPLPWLMIFELYPLEVRGRMCGISNGMCYVFTFVFTKLYYTFIANFKIQGTILLFMVASVLFGLFSAFVLPETQGKTLVEIEDRFRPKKKPDKESTLP |
| *BTST72* | MSLEAEKLEGLNTQNEAVTIIKSRYNYSRRSAFAQVLATLIQNWLLIEIGLDTAMTTMVIGALHLNSAEALSMNDEQASWFGSLPFICHPLASLLLSGYFQDRFGRRTTMILVTIPTFIAWVSLYFAQSMYVLYMVSAVTGMCTGLTEAXLHSYIGXIGEPHLXGTLSSISTSAVXVGIFMMYVFCYXFTWRTVALICSACPVITFTCMTQIXESPTWLIVKNRYEDARKSLXWLRGWVDPSEVEEEFQALVXHARNSXQKNKXAQSXGXGLIKKDSYLKTXFKEMTSKRVLLPXRLILIVFVFREITTFSAIRPYLIGELNKLHTPINAKLILILSEVLVFVGAMMNVVFLRRLGKRKIAIFANGIXXICILGTGIYCSFLQDSTRXPQAAWLPXXXXLMLSLFCGFSATLLPWQLVCEIFPIVGRGLATGITAGTKYLIQSAMVKSYLFIETYIGLSGMMYLYGTGAVLGVIHLYFCLPETEGKTLQQIESYFTKNHDRKEKYSIGKAA |
| *BTST73* | MTQASDETEKLVESPSTTAVYRQSLAALTCCLSCFTIGLSIGWSSPAFHKIQASETSFTLDGFQQSLVVSALNIGIMFGAIPTSFLMDQLGRKKTLLYTATLSLLHWVLIAGAMNAKFLYFGRFLGGIYSGIATAIAPVYLAENLEPQIRGSIGTLFSILLYGGILCTYIIGPIASYMNLSLFCGAFTVLFMVTFAPMPETPYFCIIKNRREDARKSLEWLRGHSNVDAELKQIEAYVTSEQEHVTGWSDIFTDPNLRRPFLVCVALCFIQKSTGFFTIISYQSVILPGMVGPLTSEGATLVIGVVLLMAGTASAFLIDKVGRIILLNLSYVGVVLSMIPTALWFYFNKTDEPADVEYVNHYNWVPFFGFIAFIVCHAMGLGPVGNIYPGEVLPLSIKADAMALVVSLAALFTAINTEIFAFFNAYIGMYANYFSYAVVAVVGALFTRLYIVETKGKSLQAIQEEFIEQAKMPRMKGDYFVL |
| *BTST74* | MTVGAIAGWSASAFPKIRNDELKFRLTLFQEAWVINTYYVGIMMGPLLAGIAMDAIGRKTTLLLFSIFSVANWTLVILASNEYMLYIARVFSGLWAGSVFTVCPAFLAEVLQPHVRGSLGSFLMSMYFLGNLYEYIIGPYVSYSTFGIASCIPCLIFAVAFLFIPESPYYYIMKNQRGKAEASLSWLRGDVDVNQELDAIETYAVAFMRNRGSFKDVFLNENYRAALINVQAIYFLQKLCGMFTVLAYLTVIIPPYVGPFTSENCTLIVGVVLWISTTLAASLMDRIGRKRLLVISNAGIIVTMTITGAWYYLDSTDLDLSETTYVPFLGLVIYGIFFCLGLGPIPTLYQGEILPSNIKARACTVTTMCSAWASILNTTLFAICIRYIGLYINFFLFAATSVFGLYFAKYHFIETSGKTLQEIQEELMKRRHRGKFTDDKKSPVNAKPTIYTVPMPNATEKVETKKHFEKDAKWTDE |
| *BTST75* | MFKISRSIFRQTLAAFCCSIGPMTVGAIAGWSASAFPKIRNDELNFRLTLFQEAWVINTYYVGIMMGPLLAGIAMDAIGRKTTLLLFSIFSVANWTLVILASNEYMLYIARVFSGLWAGSVFTVCPAFLAEVLQPHVRGSLGSFLMSMYFLGNLYEYIIGPYVSYSTFGIASCIPCLIFAVAFLFIPESPYYYIMKNQRGKAEASLSWLRGDVDVNQELDAIETYAVAFMRNRGSFKDVFLNENYRAALINVQAIYFLQKLCGMFTVLAYLTVIIPPYVGPFTSENCTLIVGVVLWISTTLAASLMDRIGRKRLLVISNVGIIVTMTITGAWYYLDSTDLDLSETTYVPFLGLVIYGIFFCLGLGPIPTLYQGEILPSNIKARACTVTTMCSAWASILNTTLFAICIRYIGLYINFFLFAATSVFGLYFAKYHFTETSGKTLQEIQEELMKRRHRGKFTDDKKSPVNAKPTIYTVPMPNATEKVETKKHFEKDAKWTDE |
| *BTST76* | MFIIPKRVRRQIFAALSCCIGPLMVGSIAEWSASAFPKIRSNELGFRLSVFQEAWVINLIYAGIMVGPLLAGIAMDAIGRKSTLLLFTVFAIINWTLVTFAPTKHITLLLFTVFAVTNWTLVTFAPTKHILYLGRFCGGIWNGCVITIVPAFLAEILEPDVRGSLGSLFVMMYFAGNLYENLIGPYVTYRSFCLISSAPVFVFAATFVFIPETPYYYMMKGQRKKAEASLMWLRGDGDVTVELDKIEKYAETFMKQRGSFKDLIFNEKYRKAFLNVQGVYFIQKLCGTFTVLAYLTVIIPKRVGPLAPSNCTQITGIVLLLSTFSSTFLLDAVGRKPLFIISNIGIIVTTSITGAWYFLDGHTDFNMAGTTYVPFLGILLYGGFFCVGVGPIASIYQGEVLPSNIKARASTVTTMISAFASIVNTTLFAVCNRYIGIYVNFFLFALTSVFGLYFAKYHFIETKGKTLQEIQEELMMSYQKRKASALSGKLGVCQVPIPHSVKTATKR |
| *BTST77* | MTEEETKEKLSWSCWLRTMFACSGAMMLFVFTGVVQAQSAVLLPQLKGNDSIIHVTPEEETWIASLGIFMSPVSALFVGPFIDVLGRKKGLLFFYINMGLGFSIIACASKVWHIYLGRCICSFAVGLEVAAVVYMSETCPKELRSIILSISSATLTIGISITYVIGGYLHWALASAIFAVGCFVYVIIQALAPETPPWLFKQGFKDDATRSLQQLGRSPSGILREIKLLEISAPEHTERLSIGTFLDPTIYKPFLIIFAFMFLQVLTGVYHIMYYTLNFVERLGTTYDSLQVSIIIALARMLANLTLGGYSTAFVSRKWATALSAGLGAIVLALAGAYEFLYRSVPVGQKPYEWVPIALVVVNIAASMIAVTPLPWLMGGEVFPLRVRGSMSGAVFVVGSAMMFVFIKIYEELMELLQIWGMLFFYAVASVVMVLFAVYLLPETQGKSLFEIEQGFLPKNKRLSREPEPAGGATS |
| *BTST78* | MTTDMNFGAKPESRKAILIQIISSVIASSTLLSSGMSLGFSGVALPHMEAPDSLVKVGPQEASWIASLANLATPVGCLLVGPLLDRLGRKNTMIFVGVPAVCGWLLIAVEPSLPRVYLGRLLTGLATGLSSIPSTVYTSEITSNAMRGILVTCSSISIAVGILTEYCLGWWFQRHWHCVALVSGVISILVSGLVLIGIPESPVWLVSRGQNQEASKALCTLRGTKSKNKIEKELNQIIENCRAYRGRSTSIARSISGLALPQAYKPLIIMNTYFLFQQVSGLFVIVFYAVDVIKIAGVTADAYLIAVLIAFLRLVTIIVSVWVNKAFGRRFASIISGVGITLSMFALVGYCYFVPGAAAPTPVLVNSTTTTTAIPQALVGSTDAPIPMANFSLVENVTVVMSESFQGVHGLSWIPIAALFVHIVFGTIGFLTVPWCMIGEVFPAQVRGVACSITSCFAYLSSFVVIKLYKSMLMSMGTVGIFTFYGIMSLLGTLFVMIYLPETKGKSFEAIEKHFANGSGVPASPEEVSLQTKNSKQPIIRPSRPN |
| *BTST79* | MAEEWTXTAPPKASFLRSFLVAASMFPLYICLGALIGQSAGMLPQLLEEDSTIHINKNQATWIASLPTIGTCMSSAASGYLSDLFGRIRVVQAAYSFFAIGFATMMAADSFMLLALGRFLAGIGMGCYFSGNVYLSEVTPPKYRGALLTLNSVLCSCGLVYVYIVGGYYPWYIAAAATCLISIIGLTLTFSLYDSPVWLVRQNRLKTAAKSLRLVEISSNVETKLRKLQETAENHPKTDFTLKILTEPSVWKPFVMILVLSILQNTSGFCIIIAYTVQFMWEFHSAYDPLHVTVAIGVMRLLAILVSFVLFQHFGRKTIGAVSGFGAAIFLLGVYGYLIFAPRVQLLSENQWIPIVLFLAFIFTSSLGIYPLPWILPFELFPIKVRGMMCGACLCALYLNTFVAVMLYYVLIDNLRLGGTILLFAAGSALFGIFSMTLLVETHRRTLDDIECTFASGRVT |
| *BTST80* | METQLPPTGCCKPFVAVACLFPLQILVGAIFGQSAGMLPQLLEEDSWIRIDREEATWIASLPTIGTCVAATVSGSLSDTHGRIRVTQVAYFFIGMGFAVMATANNFTMLALGRFMGGLGIGCYFPALLYVSEIAPVAHRSILLALNGLMASAGLVYIYILGGYYPWPIAATASCLLAILGLLLTLFLYDSPVWLVRHDRLETARKSLHRIENPANVEATLKHLQETASNQPKCDFTLKVFVEPIVWKPFLIILALSVLQNLAGFYIIIAYTVQFMREFHSAFDPLQVTVAIGVFRLMAIALSAVLFRYFGRKTIGAVSGFGAAACLLATYAHWKFSSMVALLAENQWIPVMLFLAYVFFMSMGIFPLPWTIPYEVYPIKVRGMMCGVSFCSMYVIMFVAVKMYNILMDNLRLEGTILLFAAGSLLFGVFSVTILIETHRKT |
| *BTST81* | MDKKTAFSIEVLEPVVRKESKKTTQYVAALTATIGGFIAGNILAWSSPAGPKLMDGEYGFPVTEDDMSWVGGIMAIGAIIGCIITGLTVDVFGRKNLMLFLVAPTTIGWCAIIWAESVFILCCGRFLLGAACGSFSIVCPMYTGEIGENSIRGTLGTYFQLQIVIGILFVYLIGSILNTFWMSITCAVIPLVYAGLMGLMPESPTFHFKKGEVENAKMSLQWFRGPEYDINGEIKEMLDIIDRDEREKVPLAIAIRSKAAKKGFVIGLGIMFFQQFSGINAVIFYTTQIFQSAGSTIPPDLCTIMTGVVSVISCYIATVIVDKLGRRLLLLTSGTVMALCCGVLGGYFYMLKHNMDVSNIGWLPIACVCGFNIAFSLGFGPIPWMLVGEIFSSQIKGTASSIACLFNWACVFMVTKFFSVIAEMFGSYSTFWFFTAMLVTAIAFTFFVVPETKGKSFEQIQSELSGENESQSEASTVSAYPSKDLKY |
| *BTST82* | MSKNDRCCGVSLSVYRQFLSAIFCCIGSLSLGLSLGWAAPAFVKIKNGEAPFELTIYEQSIVVGALNIGLMIGTYPAGYLMDRIGRKTTLLYASSFSLINWILIAFASSELYLYVARLFAGLWAGAISTLVPIYVTECSETKIRGSTTTQHLVFMSAGILLGYIIGPQVGYMDFALICGAFTVFFAIVFSFPPESPYFLTMKGRTEEARAALVWLRATDDVDNELKSIEAFIADGTKGRCSYKDLVKNPLYRRPFFICLVLWFCQKFTGFYTLIAYQTVILPKKLGVLTSDNCTQIVGVIILTSVFIASRLMDATGRKVLLTVSHVGIAVFMGIVGALYALNDTGYIQIEDYSYLLVFSFVAYVFSFSIGIGPIASLYTGEVLPQAAKGTAGSVILSLSSIASAGNTFAFAATANWIGMHWNFFFYSALSVASLVFVHLCLTETRGRTFQDIQSNLSVKKKPEMTTIEKSDGSVAHFDGRTS |
| *BTST83* | MMSSIIEVRLHLFVMISFCYFXXGLGSMTVGLMVGWPAAAFPKILRHETPYHLSIFNEAFIISCMNIGSIVGTVPASLLMDRIGRRASFLLFSLFAVASWIFVAYAPTVEMLYCGRLLGGVFVGAYLTILPSYLSETLEPDMRGFLGTSSTLLNTLGTLMAYGLGPRVSFIDLSLISCCVAIVFIISLIFMPETPYLLVMRKDYAGARRTLAWLRGTSESDVTVELTTIQNFIETEKAKASLTTSDLFFDERYKWPFVSCVGLLLLQKSSGYFTVIGNQTIILPHHAWIFYSEDSTLIIGLILVIMSIVAALLMDALGRKVLLQISNVGQASAMLVVGAWYYLSAEQRTELEAYNYMPLLAVFAYVVAFSMGLGPVPHIYIGEVLPPLVKGRATGLLVTLAALFVVSVNEIFAAVTTFADMYVNFLFFGVCSLVGIYYVNGWVIETRGKTLPEIQEEFRHRRTMKDGYYILD |
| *BTST84* | MFFKISKGIRRQLLAAFCCCIGAMTLGAIAGWSAAAFPKIRNNELEFRLSLFQEAWVINAFYIGIMMGPLPAGIMMDAIGRKSTLLFFSTFAITNWTLVTLAYHEHMLYLARFCAGLWAGSVTTVVPAFLAEVLQPNVRGSLGTMYFIMYFAGNLYEYIIGPYVTYFTFGISSGLLCFVFATSFVFIPETPYYYIMKGKRKKAEASLRWLRGDEDVSAELESIQTYVKTFMKRRGRFKELILNENYRAAFINVQAVYFIQKLCGMFTVLAYLTLIIPSQVGPLSPEHCTLITGVVLWLSVFVATSLIDRVGRKPLFVISNIGIIITMTITGVWYFLNVHSDMDLSSTTWIPFSGLLLYGVSFCLGVGPIASLYQGEVLPSNIKARASTVTAIISAFASILNTTLFAICASYIGMYINFFLFALTSVFALYFAQYHFIETKGKTLQEIQEELRKKPGFSRETSVNSNYSTVSSLPAIYTVPIARTHSLEKSAIANSLAKNA |
| *BTST85* | MSRFINRGVARQLAVAFTAGGSCFIIGALMGWPAPTLKKLRAADTPIRLSVLEESWVVNALYLTTMVSPFMCGALMNSFGRKLTLLALTVFPTLSWILVFFSRSGAMLIAARFLAGFWVGGCSTVVPIYVAEIAEPAVRGVVGTFTAVSTMLGIISAYVIGPCVSVYTMAAIYVVTPVLFFALFSLCPESPYFFVMRDQHVAAAAALTWLRARDSVTAELAAIQGSVERDAQTRQGCLRKLFSLVSVSANRKAFVTVEFMMVLQRMSGFSCLMAYSSVILPSKVGPFTSDNCTLIMGIVWLGSALICSVLVDRVGRKPLLYFSSIGIFVSMLPTSLWYYLDRETSTDVSGANWVPLAGVLIFGLTFTMGLGAIPSIYQGEMFSSSLKGIGSALTVGVCAGSSALSVSVFAVLVKFVGLYAPFLLFAAVGPATFLFVYYFVMETRGKSLQAIQDELRGEELDR |
| *BTST86* | MGDDSSEKPPDRISKPLMAAVASYACQFQLGAILGQSSNMLPQLQAADSPIQIDYDSATWIASMDVLGTPISCLLCGPLTDKMGRKATIRLFLLLSAVGHAIVGVASDVTEILIGRFCLGIAAGFAFPSIVYISEISSVEHRTPLLAINTISSSFGLLYVFIVGAFISWDIISLMTSLISVISLVYAFFIPESPAWLFQNHRLNDAIDSIKWLKGDDCDMTQELKQLKDACTEEPKGTGTIFRHFTGVTVVKPFFILLVFAFLQNGTGFYILLHYSINFISEFKIDFDPRYISIGLAVVRLSVCIMASYFLSRVNRKTAGMVSGTGMVVVLGGTLVAMYFMMGDATMSTGYSVIVTVGLLAFIFVCGLGAHPLPWIMIYELYPLHVRGTMCGVSNAINYVFIFIFIKMYYVLILNLQIHGTVILFAAFSAAFAAYSFLILPETQGKSLVEIEQGFLPKKQRQNGSA |
| *BTST87* | MIDKVLNAGQFFEKEGNQHKNACRSLLSQVVATIVLGGLVFDIGMMTTMPTLVIGALHKNSAGELKMNDDQASWFGSIIFFAHPMGALISGYLQERFGRRGSMILVNVPVLAAWLTLHLADSVYQLHLVSVLMGLCVGFCEAPLHSYIGEVAEPHLRGTISTLVCIAGHTGGVLLHVLGYLAQWRTTALFCGAVPAITFFAMTQIPESPTWLILNSRLKEAQKALGWVRGWVEAEVVHEEFQRLLEHAAVAPKSRRVSTFEGQKYEMVPLTEDGTPQKSTQETESFLRIKCRELSDQKMFRPLRMVFIVFIFSFATQLMGMRPFLVNIFNEFGLQIDSQLVVALTRFSLLVGAILNVTLLRRFGKRKLTLLCQAAATVSIILLGVYCSIFDETNRNASLSWIPISLMTSVYFFIGFSLTILPWQLCAEVFPIRGRGAAQGLSAGWSYYVRFGMSVTYLYLEKWIRLSGVFYLYGVISVIGFWYYWRYLPETEGKSLEQIESYFTDNHDKTEKFSRVKSK |
| *BTST88* | MREIDESGGTQDGQINEQKSSKKHKTDLRSASAQILATLIQDWLLLGIGLTLGVPTLVVGALYRNPASTFTLDDDQASWIGSIPFICNIIGSLASGPFQEQFGRKGSMILVNIPFFCAWLLLFYARSVASLYAASAIMGLCAGFSEAPLHSYCGEIGEPHLRGMLSTMSTSAAIMGSLLIYVFAYFFEWRGAALISSAFPVITFISMTQIPESPTWLVMRGRLEDAQRSLCWLRGWVEPDQVRDEFEALVNYTKQKVASLETAHLNILERPAIEKCNVFTAHLKGLTAKNLLRPLRMECIVIANSYTTGFAGSKPYQIQIFRQLGYGDFAKRILILGHLIFFVGAMGNLILLPYFGKRKLALFAFGLSFSCLFGIGTFGIFRMELIQIPGLFWLPLILILVFKFTLGLSIMPLPWQLLCEVFPPIGRGTASGISSAFGNLISFGMTKSFLYLKAWLDLPGVIYLYVACAFLGWLYFYFYLPETEGKTLEQIESYFTENHDRKEKFSIGKSGRKN |
| *BTST89* | MEKLNESYGMQQVTIDTENTEIKHKQNLRSTSAQLLATLAQNWLLLDIGLTFGVPPLVLGALHLNTGSGLSLNSSETSWLGSLPSICHLIGSLASGLFQEQFGRKGSMVLVNIPFFCSWLLLYHAESVLALYIAFITMGLCAGFCEAPLHSYCGEIAEPRLRGTLSTLCVAATILGAVFMYLLGYFFEWRVAALISSGVPVITFLLTTQIPESPTFLLMRGKTEEARKALCWLRGWADPDEVKEEFQALINYTKQRVASLESSGGNKNASLKFKKPNDLKAQLSELTSDRILIPFRLEFFVTLNAGITSFVGFQPYQIIVYKELGYPNFGKEILVRYTRIFLGESDTVMKKRHDTKVRSSLTNVTDEQLLGETKENQKKLLSAIRGRGTASGISTAWAYLLTFSLTKTFLVMVAWLNLGNVFYLYGTCGVLGWIYFYFSLPETEGKSLEQIESYFTKNHDRKEKFSVGKSGHLK |
| *BTST90* | MCNKTEPSKSLDVPGNDAKPSFQYKNPGKSAFAQILAIIMQNWLLIEIGLEMAMPVIVLGALHNNPAAALNLNDDEASWFGSIPDFFHPIGSLTSGLLQEKFGRKGAVMMINIPIFIGWMTLYFAKSVYMLYAVSVIMGLCTGLAEAPLHAYIGEIGEPRMRGTLSTISTSCCIIGVSLMYLLGYLFEWKTVALISSSCSVITFLMMTQLPESPTWLIVRGRLDEARKSLCWLRGWVTADEAEPEFQALVNYTRNSAGLSRQDSTNSTVDDDGPLVRKDGFLTQQFKELMNKKTFRPLRMVFTLFIICFFGYVGGIRPYFINELKKLESPIDPKLFLTMGTGWLFLGAMINVVFLRRFGKRRIAIFSHALGGVAIAGVGIYATFLQGLTQYPLRVWVPIALWTVVNFLNGLSTVTLPWQIVCEVFPPLSRGTATGLSAAWAHLVLSVHVKLYLYVEAWIGFNGIMYLYGICTLLGSTYHYFCLPETEGKSLEQIESYFTKKHDKKEKFSMGKSVQKGDP |
| *BTST91* | MFSKFMPFKSKSMSMETGNDRAIKPLICVALLIVFLAGCILGRSEDPRDEDDPYNRLKNHHDPKTFGDILYEYIRDIVKVPVIATVFFCWVCGSFADEHGRVGAMQLFFMLSGIGFGFLVYAQEYDFSILGTFILGAALGCSIPAPIYIAELCPVAYRSFFLGLVPVALSLGMFTVDVIELRGAEDTAWKSLCCFSGIGFLLSLFLHEAPEWLVMRNRPDAAIESLKWLKETSVDVDVDLRKLQETSMAANHRSDTTLEMLTDKRVWKPFAMLLGLALFQHLCGFYILIFYAPYLVNQYRTNIYWFSSYTGTDFLLLVATSAALVFHANLPRRTVAGLSGIGSSAALLGLFLHAHLFVAPQDLLDPTPDKDMLVPVFFFTLYIFSAVMGIYTLPWILMFEVFPLRHRGILCGLSFSTLYLGLFAFESRLNNYLLTGMDLQSLLCFFGTCALGFALFARSCLVETHKKTFEEIERGFTKERIFLPIDEKM |
| *BTST92* | MLKNPTSRLKLMALLEGDSIIHSDRIGYSRVPSSDIDKDAAKSHIEAETDGSTGRKISRFRSALPQVLSVTAKNFILLDLGMTLAFSTIVVPVLLNKKDPRGLSFNESQATWFASLPMLCQPLGSALSGLISGPLGRKKSLMLVNIPQILGWLMLHSATSVEIMYLAAAIQGLGSGFMDAPVLTYVGEICEPSLRGILISYSLQFCSAGFFLQCLLGSVTTWRNVAFISLFFPATAFLCISQIPETPMWLLSKGRTRDAEKALCWLRGWVSPEDVAEEFSRLVEASNNAQYRHHAETKKPETNSHQGNSQSIVTCPSSRPPIPDPLVELHSPKLSFREKAKDLLRPEILRPMLVIMTMNFFYLGSGFPAFKTYMVLLFQRIRVPMNANWASVCVSSAIIIGHLVLMLAVKWLGKRRIALISIFCVAFFDLAIAVHVNFQTAIEDAFGASANWFLFSYFIILTLTVSFGLGPVPWMLMSEIFPFRGRSFASGICAALYYITSSLIAKTFLSILNLLGVPGSYCLFGTVGMLGFIYAYLYLPETEGKTLEDIEDIYKHSHRQRR |
| *BTST93* | MNKEEEDEKITWSCWLRTLFACSGAMLCFIFSGVTEAQSSTLLPQLKAKDSIIHVTPEEETWIASLGILMAPIASVVVGPTIDLIGRKKGLLFFYLDMGVGFTIIACATEVWHIYVGRCVCSFAIGMEVAAVVYFAETCTKKQRSVLLSIISASFTFGVSLTYFVGGYLPWNVASGVFALGCFLYFLIQLLAPESPAWLYKKGDIDASKRSLQRLGRSPSGILRELEMLRLSSKEQSEKFQFKVFLEPTVWKPFLIMCIFHILLNLSGVFHIMYYTLDFIERLGTSYDPLTVSIIISVTRVISCSTVGIYSTAYVGRKPATIVSSILMTLSFLSAGIYEYVFRDTPVGQKPYEWVPIVLLISNLVSGILAVAILPWLISGELFPLQIRGSMNGAVYVFGTSLMFVSIKLYAVCLEVFQMWGLLLVYSLGSFLAILFGIFVLPETQNKTLLEVERGFLPKNRRDIAPSPNAEATKNCGDTSQSEAGLERR |
| *BTST94* | MDEEKEDEKISWSCWLRTIFACSGAMMIFIFSGVTETMMIFIFSGVTETLSSTLLPQLKEKDSIIHATPEEETWIASLGILMAPIASLVVGPTIDLIGRKKGLLFFYLDMGVGFSIIACATEVWHICVGRCVCSFAIGMEVAAVVYIAETCTKKQRSVLLSILTVSFTFGVSLTYFVGGYLPWKVASGVFATGCYLYFFIQLFVPESPAWLYKKGDIDASKRSLQKLGRSPEGTLRELEILRLSAKELSEKFRFKVFLEPTVWKPFLIMSTFHLLQNLSGVFHIMNYTLDFIQRLGTSYDPLTVSIIIAVSRVITCCTAGMYATASVGRKPATIVSSILMTLSFLGAGIYEYVFQDTPVGQKPYEWVPIVLLIVNIISGSLAVSILPWLMSSELFPLQVRGSMNGAVYVVGTSLMFVSIKLYAVCLEIFEIWGLLLVYTLGSFLAVLFGIFVLPETQNKTLLEVERGFLPKNRRNIASSPNAEATRNSEDAGQIEAGSERSDESKE |
| *BTST95* | MERDDEDEKLSWSCWFRTMFACSGAMMLFVFTGVTEAQSAVLLPQLKERDSTIHTTLEEETWIASLGILLSPVSGVIVGPTIDAIGRKKGLLFFFINMGLGFGIIACATKIWHLYVGRCICSFAVGMEVAAIVYLAETCTKKQRSMLLSMVAASFTTGCSITYVVGGYLPWNIASAIFALCCFLYFLVQLLAPESPAWLYKQGRIDASTRSLQKLGRSPEGIARELEMLRLSSKEQSEKFQLKVLLEPTVWKPFIILCIYHFLQCATGVFQILYYTLDFIDRLGTSYNPLRVSIVISIVRVIANSTIGMYATAYVGRKPATVTSGILMTISFLGAGAYEYIYRTTPVGHRPCEWVPIVLLIVNIVGGILGVCILPWLMSGEVFPLRVRGSMSGAVFVVGSAIMFVSVKIYAAAMEVLAMWGMLFVYAAASFLAVLLGAFFLPETQNKTLYEIERGFLPRDRGDSRVQATEKSEIENETTVS |
| *BTST96* | MNEGTANLLLSQMKGATSLIHLSQDQETWVASLGILSAPIAAILIGPFIDAFGRKRGVLLFYLNMGLGWAVIASAREVTQIYIGRIICAFGEGFQACAVVYLTEICTKEQRSVVLACLIALFSGGVLFVSVVNTCLPWPMACSAFSLASFALAGAECFVPESPAWLFSQGEEAAAVRNLQKLGRSKAGVLLEIDALKERESCTEVLSWRTFLRPTVWKPFLILAVFHLLQFSTGFYDMIYYQVDYLERLGTKYDPIALSVAFSTVRFLSNATIGIYFRSLDRKFSTTVSGLCMTVPLLGAGIYELKYRDTPPLEKPFQWLLLFCIFAQLVAGNLAVTCLPWSMGAELYPLNVRGIMSGATLCVAYSIFFTYVKLYHVAMGALKVYGLLCLGLLF |
| *BTST97* | MTRINAHGVKKKDSFFGPFVASIPAFVTQVLAGVLEGHAAVLLPQLNDASSPVSISSDDAPWIVSLGFVLTPFIAVIRKPLMDTFGRKCNLYLFFVLSTFGFLSVSVASAPAHLYLGRVISSAAYGLSPDSFVYVSEICADGQRSSFLFFLSLMRPAGLLMVYSLSTILEWGICSMVFVVTSHIGLLFVFHVPQSPVWLVQQSRLEEAATSLKWLRSDHDVIDKEIAAMKISTPDEEFQQRSGLVAFFNPSIFKPLAALVAYSVLQHATGLLVVLSFALDFFGSLSSPFNPAYVIVCIAFYRIFLTAFWVYLSSKFDKVFLTTTAAFGAGCLVLSVCVMQVAYPDVVGRNESLQLLQLSLCASYTLIGGGIDFHNLPVDLRTVFPPDVLGTVRGFVKFLGGMFLFLSMLSYPFLVRVFGLSWVLFFFGISCFSFGLLVKSLLPKFKGKTRTDDTEGTHSTRLQLTTYL |
| *BTST98* | MSRGPYQMTGHALQPLNPTSNGLGTHTAGTMVNLPRGKSQYLSQVLAAVAISLGPLAAGLGKGYSSPAIASLQGKQSWEAGHGAGAYRGHGMGHRGNYTLLTVSPQEASWVASLSLLGALFGALVGGLAMKFGRKNVLLIASLPFSASWLVTVYAESVQTMFATSFVGGFCCAVVLMVSQVYISEISDPDIRGFLSAVLKIFSHIGTLLSLTLGAYLDWRELAMIISGAPLLLFVSMLYMPETPSFLVLSGREPDAVRALRFLRGNDTDITRELITIRNNILTASTHQYTYRGLAHAAARLAHPILITCGLMFFQRFSGANAFQFYSVTIFSQTFNGMNPHGGAIVVGFVQLLASLLSGLLIDTIGRLPLLIASSVFMSIALAGFGSFVYYEQLSRHNSYVHVQHLPPGVAPPGISATYDWIPLLCVLVFTVSFSMGISPISWLLIGELFPLEYRGLGSALATSFSYACAFIGVKTYVDFTQTLGLHGAFWLYAAFSLAGLCFIVCFVPETKGRDLDELDSRYI |
| *BTST99* | MISSGKTQSPNERPYQYEYTALGTQDVEKSGEHGTGVSGNRRKVNRFRSAAPQILAVTAKNLVLLDLGMTMAFSTIVVPVLLDPNNKDPNGLSFTEDQATWFASIPMVFQPLGSALSGLISAPLGRKRSLMLVNIPQIIGWLMLYSSSSVNIMYLAAAIQGLGAGFMDAPIFTYVGEICEPSLRGVLISYSLQFCSVGFFLQCLLGSLTTWRHVAFISMLFPTLAFLAISQIPETPMWLLSKNRMKEAEKALCWLRGWVSKEEVAEEFAQLVQYSKNSKYKSDDDKKKLQMDLISTAKQPCGGCTRPPIVPCDSNTGDDDYAKLKLHEKVKDLLRPEILKPMSIIIIVNFLYFTSGFPGFKTYMVLLFQRVHSPIDPNWASVFVSTSIILIHIAQMVAVKTIGKRWMTLISSFGAAVAGLAIGVHMSFQGFFDETFGDLSNWLLFTYFEILTLATVIGLGPVPWMLMSEIFPFRGRSFASGFCAAIYYAASFFAAKTYLSTLNLFGVAGTYYIFGTISALGLVYVYLYLPETEGLTLEEVEDIYRPKKRSEVKNL |
| *BTST100* | MDSTRGLRRQVTACIIANQGLFLIGINLGWSSAVNEHLLSGVLGYKYTQDQLSWAVSLLDLGTVFAPLPTGYLMNKIGRKFTFLLIASLFTLSWCLKVISVQPGFLYAAQILAGVARGVGLTVTPMYSGEIAETGLHGMLSTIFKLMFYSGMLLMIIVAPYMNYTTISYMGLTFSLLFFFSLFYIPDTPYYYCAVKKEREAFQSLKWLRNQDKTENASVLNKELAMIKVAIEKVMGEDSGFRGLIMKPSNRRALFIVLGLFILQRMIGLNTIIGYGSITLPKGHPFITPQTGMISFVVALFISSALIALFIDRIGTKPLLISSSIGCGFCTSVIAVYYWCDRTNGKAAVAGFFWVPYLFFVLEAFVFSIGVGVVPTVYLSQLFPINVVGQASAASVIVASFVTFVINKAYFYVGVQFGIFMMYVFFSMSAFGCAAFTHFFAIETRKKRDAEIVPVGSVIETASARK |
| *BTST101* | MPLRNVPVSILPFVYGLVQTWPNLAIEEILKGEAGFAVDPEDIAVIVSMVTLGEFLMPILFGFVLVRLGRKTNMLLNALIYAVAWALIIFARSPFWLIAANFAAGLGCGIILIIGPIYMAEIADSKIRGALISVYITVIAIGQIFMTSVGIFISYFDMNLIALILSIVAFFCILVIAVESPSWHLIDNDEVKAEESFNYYWNTGEEADRPQAGEALAELKETVKLEMESSSSYLELFRTPSNIRASIIVITLSFFQGASGIVAVLTYGSTTLPRYDGFWKPYPTMALVSALNLVFNLVSIALIDKLGRRPLSIVSNAGDALGTGIVAVYFFLEQNTEWDTTNIKWLPYIGMILYISSYGGAMAVIPHILVGELFPTNVR |
| *BTST102* | MTTMVIGALHNSASEELSMNDDQASWFGSLPDICHPVSSLLSGYVQEAVGRKTAMIMVTIPCFVAWITLYFAQSINTLYIVSIIMGLCTGLTEAPLHSYIGEIAEPHLRGTLSSISTSAALIGMFMMYVFTYFFYWRTVALICSACPVITFTVMTQIPESPTWLVVRNRLEEAKKALCWLRGWVKPDEVEEEFQALVKHAKKSVGLNQAGDDKPRTKIAFLKMQLTEMTRKKVLLPFRQICIVFFICSLAYFCAIRPYLIGELQKLDTPIDAKLILIYSQVLLFIGAMMNVMFLRRFGKRKIAIFCNTVIAISMFGLGIHYAYLKGSKQFPLLAWLPVVFWLSISFFGGFGPALLAWQLVSELFPIIGRGLATGISAAFSKLMGAAEVKSYLYIEAWVDLSGVMYLYGTATILGTLYLYFYLPETEGKSLEQIEVYFTENYDRKEKFSIGKRVKLEEKNPS |
| *BTST103* | MGPLTDAPESHSIKNGNAASSNRPRHSTRSTCAQVLATLIQNWLLIEIGLDIAMTTMVIGALHNSASEELSMNDDQASWFGSLPDICHPVSSLLSGYVQEAVGRKTAMIMVTIPCFVAWITLYFAQSINTLYIVSIIMGLCTGLTEAPLHSYIGEIAEPHLRGTLSSISTSAALIGMFMMYVFTYFFYWRTVALICSACPVITFTVMTQIPESPTWLVVRNRLEEAKKALCWLRGWVKPDEVEEEFQALVKHAKKSVGLNQAGDDKPRTKIAFLKMQLTEMTRKKVLLPFRQICIVFFICSLAYFCAIRPYLIGELQKLDTPIDAKLILIYSQVLLFIGAMMNVMFLRRFGKRKIAIFCNTVIAISMFGLGIHYAYLKGSKQFPLLAWLPVVFWLSISFFGGFGPALLAWQLVSELFPIIGRGLATGISAAFSKLMGAAEVKSYLYIEAWVDLSGVMYLYGTATILGTLYLYFYLPETEGKSLEQIEVYFTENYDRKEKFSIGKRVKLEEKNPS |
| *BTST104* | MQNDTAETHRHMHTRHKIFSRSAFAQILAILMQNCLLIEVGLDTAMPTMVIGALHKNPSETLNMNDDEASWFVSIPAIFYPLSSLTSGYVQELLGRKKAMLLVTIPTFAAWMILYFAQSIYTLYAASTLMALCNGLTEAALHSYVGEIGEPHLRGTLSTISISAMLFGGLMMFVLGYFFDWRTVILICGAYPIITFMVISQLPESPTWLIVKNRKEDAKRALCWLRGWVKPEEVEQEFQAHLSYAERSVSSSLEKLVSNGSLENLDYIKIQFTEMAKERVLRPLRMICIMFIICITAYCWAIRPYQIGELKKMGSPVDPKLVLIGAQVFIFVGLMMNVLFLRRFGKRRIAIFSCSIIAFCMFGIGFHHANLKGRETTFLAWLPVILWLTINCFAGFSAALLAWQLVSEVFPIVGRGLASGVSAAWSSIVVFVMIKSYLYIEVWIGLSGVMYMYGTITALGVLYLYFYVPETEGKTLEQIESYFTDNHDPEEKFSIGKSK |
| *BTST105* | MTNEMESSENQAESQTEDLIPKPKEIKYKNAGRSTFSQIVAMLVLACLLVDFGLELIIPTIVIGALHKNPDEALNLTDEQASWFGSILYFAHPIGALISGFLQELLGRKRSLLLVNIPMLVAWSTLYLASSVYQLYFVSAALGLCIGFCEAPLHSYIGEMSEPHVRGTLSAMGTASCLMGMLIMYLIGYLVHWRTAALISSFVPVITFLAIAQIPESPTWLVMNGREKEAQKALGWLRGWLKPEEVQEEFQRLLEYTDTKPKSKRFRGSQREKYEMVPTSENGVPQPLREHDESYWRKKFREITNKKLYLPLRMVFIVFFFGIATQLAAMRPFMVGVLIQFGLTVDNYLVLVLISFFYFVGAMMNVIFVRRLGKRRLTLYCQAIATLSILLLGVYLAYLTGPAKVRAIDWIPISLFVSLFFASGSSIALIAWQLCAEVFPVEGRGTAQGLVAAWAYLVNFVMSKSYLYLERLVQLKGVFYFYGALSALGFFYYWRYLPETEGKSLDQIETYFTENCDEKDKFTKRKNNRG |
| *BTST106* | MTENLPQSMETRQETSNPSTFRQLLPQVLACSAKSVLYLSLGMLVGLPTLLIPDVTDPSNLNELFLDNDQASWYGSLTYIFQPLGSLASGALLQSVGCKKLMIMVNIPQFVSWIMTYYASSALVLYISSALVGLVVGLMEAPTCRYISEITHPNYRGVLTSYSTSFVTVGFLLVYALGLVTNWRNVALISASTPVLAIIVLLMIPETPIWLMSKGRSEEALESLQWLRGWTTKEAVQEEYTKLQFYAKKQQGKTIHAYGKEVDIADEKPTVLNGASSGGHLEVEEVEERRGLKEKIRELTCKEMLVPLGKCIVLFFVSICSGLLSLRPYFVQVFEEFDLPTDGLTTSVLISVIAIVGNIVCMLIINRVKKRPLIIFSLISTALCLILLALFLMAPTNPHNNASLRWWSLILFLIVNFVNNLGIYPISWTYLSEILPYRGRGIATAIGSSFFYIVIAVGVKTYPSLEQQIGLDGIFLLYAVISLAGAYFTYFSLPETEGKFLSDIETHGKDKKIQVPSASLRP |
| *BTST107* | MRCFGSARGLTFFLSYTILASMLGMFQFGYNTGVINAPEKNIKDFMKDVYKSKYSEDISEETVQFLYSFAVSIFAIGGMIGGFSGGLIANKFGRKGGLLLNSFVGIAGASLMGFAKFFHSYEMIFIGRFIIGVTCGLNTSLVPMYISEIAPLNLRGGLGTVNQLAVTTGLLISQILGIEQILGTDDGWPLLLGLAICPAVLQLILLPVCPESPRYLLITKQWEEEARKALRRLRASNQIEEDIEEMRAEQRAQQAEATISMTQLLCSRTLRPPLIIGVVMQLSQQLSGINAVFYYSTGLFVSSGLSEETAKFMTPGIGVIMVTMTIITMPLMDRLGRRTLHLYGLGGMFIFSIFITISFLIKEMIDWMSYLSVISTLMFVVFFAVGPGSIPWMITAELFSQGPRPAAMSIAVLVNWIANFVVGIGFLPMKTALENYTFLPFSVFLAIFWIFTYKKVPETKNKTFEEILALFSKPGDTPPTRGQPQANPAFEETPLNTTKAFTGSTSTSLLNCVDQRLPPSERAALMVAEEKPLPDTSSSSSSSVLPASLYDADGTMSPPVNPGSRNQMQYGAASEHCVRDLSQPSRPPPPLPPPRSFLPNSAV |
| *BTST108* | MREIDESGGTQDGQINEQKSSKKHKTDLRSASAQILATLIQDWLLLGIGLTLGVPTLVVGALYRNPASTFTLDDDQASWIGSIPFICNIIGSLASGPFQEQFGRKGSMILVNIPFFCAWLLLFYARSVASLYAASAIMGLCAGFSEAPLHSYCGEIGEPHLRGMLSTMSTSAAIMGSLLIYVFAYFFEWRGAALISSAFPVITFISMTQIPESPTWLVMRGRLEDAQRSLCWLRGWVEPDQVRDEFEALVNYTKQKVASLETAHLNILERPAIEKCNVFTAHLKGLTAKNLLRPLRMECIVIANSYTTGFAGSKPYQIQIFRQLGYGDFAKRILILGHLIFFVGAMGNLILLPYFGKRKLALFAFGLSFSCLFGIGTFGIFRMELIQIPGLFWLPLILILVFKFTLGLSIMPLPWQLLCEVFPPIGRGTASGISSAFGNLISFGMTKSFLYLKAWLDLPGVIYLYVACAFLGWLYFYFYLPETEGKTLEQIESYFTENHDRKEKFSIGKSGRKN |
| *BTST109* | MGTAVADIYHDSVSNSGKSRRFDNQNNNINMFPRKENPAKPFRKALPQILAVTAKNLLVITYGMTLGLPSIAIPALQEKRNSTMNDGHPHDQLTLDEAQISIFSSLNLICVPIGCLLSGVLTQPFGRKRCMIFLNLPFIVAFLSFCYSSSVPMLYTALIISGLSGGLLEAPVLTYVAEITEPHLRGMLSATASMTTILGTVSQLLLGNFFTWRTVALIDLFFPVAAIVALCFVPESPHWLISKGRITAAEKALCWLRGWVEPDSVQSELSLLQKSHDQSLNRSSPTSSMYIMYMKRTFLIPFLIITMSYFIGHFGGMTVIQTYVVSIFEDLGAPIDKYFAAMLFGLVELAGALTCVGLIHYTGKRPLTMFSTVGCGICLFGVATCAYMGYGDASSKSQYSSFSTMLLLATAYLSHASIRLLPWIMIGEIYPAEIKGMASGASASVSYIFAFTANISYDTMIRYLSLHGTMYFYSAISLLGSLFLYYCLPETENRTLHEIENHFANKEHLFKKKISKTDILHRKDEKPEENEDESTEADCILETS |
| *BTST110* | MGKDEDEEKLSWGCWLRTAFACSGAMMAFVFNGVTEGQSAVLLPQLKEKESFIHITSEEETWIASLGILLSPVSALLIGPITDAFGRKLGLLFIHIFMGLGFAVIACATQVWHIYLGRCICSFALGLEVVSVVYMTETCAKRQRSFLLSTISPAFTLGVVVAYVIGGYLPWNIASAIFALSSFVYFVVQLLAPESPAWLFKRGRIDAAAWSLRKLGRSPSGIDHELQLLKLASSEESESFHLGIFLDPTVWKPFLILSLFHLVQCATGIYHIVYYTLDFVTRLGTTYDPLTVSIVISVVRVISNCTIGMYFTSYVSRRFSTILSALLMTVSSGAAGVSSSGIIGVTTLPWMMSGEVFPLRVRGAMSGAVFGVGAGSMFVFIKIYEDCLALLNIWGLLFGFAIASFLTALLGIFLLPETLNKTLYEIEQGFMPKEKRSNGEESTLPAEAVS |
| *BTST111* | MGGEDQKPRASKQSANGRLLFAISAAALGSAFQHGYNTGVVNAPQSLIESWISDVLRNRSGAGAEYKPEPSQVTMIWSIAVSIFCVGGMIGGSLTGLIAEKLGRKYGLLYNNILVLAGCLLQQHSKNFGSYEMFIAGRFFIGVNCGLNAGLVPMYLSEISPMNLRGAVGTVYQLVVTISILISQIFGLKSVFGTAENWPLLFEIALLPSIFQVITLPFCPESPRHTLLHHGLELQAQKDLSWFRGTIEVHDEMEEMKNEYEAMKLTPQVTIREMLSNAQLRIPLFIAAMVMVCQQLSGINAVMFFSTKIFKMAQLSDEAAQYSTLGMGSMNVLMTLISLVLVEKAGRKTLLLIGFSGMFVDTVLLTICLAFVEKSIVISYFCILLVIVFVVMFAVGPGSIPWFLVSELFNQSARPTAASIAVAVNWTANFMVGLGFLPLQEALGSNVFVIFAVLLGLFVLFVWKKVPETKNKTMEEISSMFRQISYQ |
| *BTST112* | MGPLTDAPESHSIKNGNAASSNRPRHSTRSTCAQILATLIQNWLLIEIGLDIAMTTMVIGALHNSASEELSMNDDQASWFGSLPDICHPVSSLLSGYVQEAVGRKTAMIMVTIPCFVAWITLHFAQSINTLYIVSIIMGLCTGLTEAPLHSYIGEIAEPHLRGTLSSISTSAALIGMFMMYVFTYFFYWRTVALICSACPVITFTVMTQIPESPTWLVVRNRLDEAKKALCWLRGWVKPAEVEEEFQALVKHAEKSVGLNQAGDDKPRTKIAFLKMQLTEMTRRKVLLPFRQICIVFFICSLAYFCAIRPYLIGELQKLDTPIDAKLILIYSQVLLFIGAMMNVMFLRRFGKRKIAIFCNTVIAISMFGLGIHYAYLKGSKQFPLLAWLPVVFWLSISFFGGFGPALLAWQLVSELFPIIGRGLATGISAAFSKLMGAAEVKSYLYIEAWVDLSGVMYLYGTATILGTLYLYFYLPETEGKSLEQIEVYFTENYDRKEKFSIGKRVKLEEKNPS |
| *BTST113* | MCSNPDMTSSSSMQNETAETHPHMHARHKMFSRSAFAQILAILMQNCLLIEVGLDTAMPTMVIGALHKNPSETLNMNDDEASWFVSIPAIFYPLSSLTSGYVQELLGRKKAMLLVTIPTFAAWMILYFAQSIYTLYAASTLMALCNGLTEAAIHSYVGEIGEPHLRGTLSTISISAMLFGGLLMFVLGYFFDWRTVILICGAYPIITFMVISQLPESPTWLIVKNRKEDAKRALCWLRGWVKPEEVEQEFQAHLSHAERSVSSSLEKLVSNGSLENLDYIKIQFTEMAKERVLRPLRMICIMFIICITAYCWAIRPYQIGGLKKMGSPVDPKLVLIGAQVFIFVGLMMNVLFLRRFGKRRIAIFSCSIIAFCMFGIGFHHANLKGRGTNFLAWLPVILWLTINCFAGFSAALLAWQLVSEVFPIVGRGLASGVSAAWSSIVVFVMIKSYLYIEVWIGLSGVMYMYGTITALGVLYLYFYVPETEGKTLEQIESYFTDNHDPEEKFSIGKSK |
| *BTST114* | MTNEMESSENQAESQTEDLIPKPKEIKYKNAGRSTFSQIVAMLVLACLLVDFGLELIIPTIVIGALHKNPDEALNLTDEQASWFGSILYFAHPIGALISGFLQELLGRKRSLLLVNIPMLVAWSTLYLASSVYQLYFVSAALGLCIGFCEAPLHSYIGEMSEPHVRGTLSAMGTASCLMGMLIMYLIGYLVHWRTAALISSFVPVITFLAIAQIPESPTWLVMNGREKEAQKALGWLRGWLKPEEVQEEFQRLLEYTDTKPKSKRFRGSQREKYEMVPTSENGVPQPLREHDESYWRKKFREITNKKLYLPLRMVFIVFFFGIATQLAAMRPFMVGVLIQFGLTVDNYLVLVLISFFYFVGAMMNVIFVRRLGKRRLTLYCQAIATLSILLLGVYLAYLTGPAKVRAIDWIPISLFVSLFFASGSSIALIAWQLCAEVFPVEGRGTAQGLVAAWAYLVNFVMSKSYLYLERLVQLKGVFYFYGALSALGFFYYWRYLPETEGKSLDQIETYFTENCDEKDKFTKRKNNRG |
| *BTST115* | MSLEAEKLEGLNTQNEAVTIIKSRYNYSRRSAFAQVLATLIQNWLLIEIGLDTAMTTMVIGALHLNSEEALSMNDEQASWFGSLPFICHPLASLLLSGYFQDKFGRRTTMILVTIPTFIAWVSLYFAQSMYVLYLVSAVTGMCTGLTEAQSPTWLIVKNRYEDARKSLCWLRGWVDPSEVEEEFQALVSHARNSVQKNKAAQSVGDGLIKKDSYLKTQFKEMTSKRVLLPLRLILIVFVFREIFIGAMMNVVCLRRLGKRKIAIFANGIVAICILGTGIYCSFLQDSTRFPQAAWLPVIFWLMLSLFCGFSATLLPWQLVCEIFPIVGRGLATGITAGTKYLIQSAMVKSYLFIETYIGLSGMMYLYGTGAVLGVIHLYFCLPETEGKTLQQIESY |
| *BTST116* | MAENPCETCRHSYKNGRRSMMSQVVVMLVLSGLFMNTGMQATMPTLVIGALHDNSAAHLELNDDEASWFGSILSFSHPIGALTSGLLQERLGRKGSMLFVNIPTLAAWTILFRATSVYQLYLVVVLMGVSIGLMEAPLHSYIGEVGEPHFRGIMSTMATAATILGVLAMHVLGYLFPWRKVALISAAVPLISILCLTQIPESPTWLILNGRAKDAQKALSWVRGWLRPEEVQKEFDELLHHTEVALKINRTSASDRETYRAVPAVENEALSRQVEPVLTEENGSCLKIKCEELTDKKVYRPLRMVCIVFFFSSVTELSELRPFMVGIFKDLGFPIKNHPISVLTAVFFFIGAMLHVVFLRRLGKRRLTLISQALATLSILLLGVYCTFFNKPNSSLVWIPISLMSCISFCGGFGIALIPWQLCAEVFPLKGRGTAQGIAASWAYYMRFVMSKTHFYLERWIKLNGVFFLYGVIAVAAFLYYLQYLPETEDKSLKHIETYFTENHDKTEKFCKPKHNRSHNVR |
| *BTST117* | MCHPSNPSKNVSIEDGPAEANVQYKYTRRSAFAQILATLIQNWLLIEIGLDWAMPTMVIGALHRNSEESFNMDDDEASWFGSIPSICHPLASLSSGYFQELLGRKSTMILVTIPTCAAWITLYFAQSVQTLYLVAGTMGICTGLTEAPLHSYIGEIGEPHLRGSLSTLSQSAGFIGVFLMYLLCYFYDWRTVALICSACPIITFTSMTQIPESPTWLIVKGRFEEAKKSLCWLRGWVKPCEVEEEFQTLVDHTKKSVKSIDSTQMVEDCASAKIDGYLRMQFERLTSKKVLLPLGMVCIVFFFSVVAGFVGIRPYLIGELKKLGVPIDPKLILIAFQVLVFVGAMMNVLLLRRFGKRRIALFSYSTHAFSILVLGIYCSSMESLEQFPQLAWLPVIFMLTLGFLTGFSSTLLPWQLVSELFPIVGRGLASGISAAWAYIVGFILVKSFLYTETWIGLSGVTYAYGAVSVIGVAYIYFYLPETEGKTLEQIEKYFTKNHDRKEKFSIGKSTNNSGLEHSS |
| *BTST118* | MKSSEEQTEVSFEEPPDTSKIRYENSRRSTCSQVVVMMVLVGLLLDIGMQASMPTIVIGALHRNPSERLSMNDEQASWFGSILSFSHPIGALISGFLQERFGRRGSMILVNIPTLAAWTTLHLADSIYQLYIVAATMGLSIGFLEAPLHSYIGEVGEPHFRGTMSTMGTAAALLGVLTIHMLGYLVRWRTAALISTAVPLITIVCLTRIPESPTWLIMNGRIKDAQKALGWIRGWLRPEAVQKEFQQLLNHIEAAPKIHRTRSIDNETYQMVPTADCEAESSLRPPEENKSYLRSKYEELTDKRLYRPLRMVFIVFFFTSATELSGMRPFMVGIFKDFGFAIDSQLLLVFSIAFFFAGAMLNVVLLRRLGKRRLTLMCQAVATLCILLLGTYTTLFNKSNRIPSLVWIPVTLMSCINFCGGFAITLIPWQLCAEVFPLKGRGTAQGLAAAWAYYVRFVMSKSHLYLERWIKLNGVFFLYGAVAIIAFLYHLRYLPETEDKSLEKIESYFTEDHDEAEKFLKPKSSNKSR |
| *BTST119* | MCHPSNPSKNVSIEDGPAEANVQYKYTRRSAFAQILATLIQNWLLIEIGLDWAMPTMVIGALHRNSEESFNMDDDEASWFGSIPSICHPLASLSSGYFQELLGRKSTMILVTIPTCAAWITLYFAQSVQTLYLVAGTMGICTGLTEAPLHSYIGEIGEPHLRGSLSTLSQSAGFIGVFLMYLLCYFYDWRTVALICSACPIITFTSMTQIPESPTWLIVKGRFEEAKKSLCWLRGWVKPCEVEEEFQTLVDHTKKSVKSIDSTQMVEDCASAKIDGYLRMQFERLTSKKVLLPLGMVCIVFFFSVVAGFVGIRPYLIGELKKLGVPIDPKLILIAFQVLVFVGAMMNVLLLRRFGKRRIALFSYSTHAFSILVLGIYCSSMESLEQFPQLAWLPVIFMLTLGFLTGFSSTLLPWQLVSELFPIVGRGLASGISAAWAYIVGFILVKSFLYTETWIGLSGVTYAYGAVSVIGVAYIYFYLPETEGKTLEQIEKYFTKNHDRKEKFSIGKSTNNSGLEHSS |
| *BTST120* | MWPWKKLESKDKLKSSKWLSGSIESLSEEELTAVYGAGARIHSSHHRPPPSALSSTCSSSTSVATNGSTVALLHSSQRKKSKNKYNVHKYNLYNKGIKMTSGAQKELNLLLTAPTVPEIIKSESEKDPKKDEANDGRLQNSHEATFETLNSSGIIIKEKQDSSQSASIIPQVLASLSVSLGSLAVGFSSAYTSPALPSMTDATSILYGKVSAEEMSWIGSIMPLAALFGGMAGGPLIESLGRRTTIISTAVPFIVSFLLIALAVNVAMVMTGRAIAGFCVGIASLALPVYLGETVLPEVRGMLGLLPTTLGNIGILLCYVAGAYLDWSMLAFAGALIPVPFLICMFFIPETPRWYIGRNKHKKARKALQWLRGQNADISAEFDEIEKTNAESNKNEKTAGCSELFAKMYRRPLLISIGLMFFQQMSGINAVIFYTVKIFKEAGSTIDGNICTIIVGIVNFGSTFVATMLIDRLGRKVLLYISSIAMIATLGVLGLFFWAKERNIDVTAYGWIPLASFVIYVIGFSIGFGPIPWLMMGEILPAKIRGPAASLATSFNWSCTFIVTKTFVDLLALIGSSGTFWLFTGICAVGLVFVVLFVPETQGKSLEDIERNLTGGPKVPVRQCRRMSSIANLKPLP |
| *BTST121* | MGKDEDEEKLSWGCWLRTAFACSGAMMAFVFNGVTEGQSAVLLPQLKEKESFIHITSEEETWIASLGILLSPVSALLIGPITDAFGRKLGLLFIHIFMGLGFAVIACATQVWHIYLGRCICSFALGLEVVSVVYMTETCAKRQRSFLLSTISPAFTLGVVVAYVIGGYLPWNIASAIFALSSFVYFVVQLLAPESPAWLFKRGRIDAAAWSLRKLGRSPSGIDHELQLLKLASSEESESFHLGIFLDPTVWKPFLILSLFHLVQCATGIYHIVYYTLDFVTRLGTTYDPLTVSIVISVVRVISNCTIGMYFTSYVSRRFSTILSALLMTVSSGAAGVXXXGIIGVTTLPWMMSGEVFPLRVRGAMSGAVFGVGAGSMFVFIKIYEDCLALLNIWGLLFGFAIASFLTALLGIFLLPETLNKTLYEIEQGFMPKEKRSNGEESTLPAEAVS |
| *BTST122* | MSRGPYQMTGHALQPLNPTSNGLGTHTAGTMVNLPRGKSQYLSQVLAAVAISLGPLAAGLGKGYSSPAIASLQGKQSWEAGHGAGAYRGHGMGHRGNYTLLTVSPQEASWVASLSLLGALFGALVGGLAMKFGRKNVLLIASLPFSASWLVTVYAESVQTMFATSFVGGFCCAVVLMVSQVYISEISDPDIRGFLSAVLKIFSHIGTLLSLTLGAYLDWRELAMIISGAPLLLFVSMLYMPETPSFLVLSGREPDAVRALRFLRGNDTDITRELITIRNNILTASTHQYTYRGLAHAAARLAHPILITCGLMFFQRFSGANAFQFYSVTIFSQTFNGMNPHGGAIVVGFVQLLASLLSGLLIDTIGRLPLLIASSVFMSIALAGFGSFVYYEQLSRHNSYVHVQHLPPGVAPPGISATYDWIPLLCVLVFTVSFSMGISPISWLLIGELFPLEYRGLGSALATSFSYACAFIGVKTYVDFTQTLGLHGAFWLYAAFSLAGLCFIVCFVPETKGRDLDELDSRYI |
| *BTST123* | MDSTRGLRRQVTACIIANQGLFLIGINLGWSSAVNEHLLSGVLGYKYTQDQLSWAVSLLDLGTVFAPLPTGYLMNKIGRKFTFLLIASLFTLSWCLKVISVQPGFLYAAQILAGVARGVGLTVTPMYSGEIAETGLHGMLSTIFKLMFYSGMLLMIIVAPYMNYTTISYMGLTFSLLFFFSLFYIPDTPYYYCAVKKEREAFQSLKWLRNQDKTENASVLNKELAMIKVAIEKVMGEDSGFRGLIMKPSNRRALFIVLGLFILQRMIGLNTIIGYGSITLPKGHPFITPQTGMISFVVALFISSALIALFIDRIGTKPLLISSSIGCGFCTSVIAVYYWCDRTNGKAAVAGFFWVPYLFFVLEAFVFSIGVGVVPTVYLSQLFPINVVGQASAASVIVASFVTFVINKAYFYVGVQFGIFMMYVFFSMSAFGCAAFTHFFAIETRKKRDAEIVPVGSVIETASARK |
| *BTST124* | MTSSVSDEETRGKISSNEETKDKTVSDTEIGAKPVNDEECGEEVSWRCWIRTLFAASGAMMVFVFTGVTEAQSAVMLPQLKKPDSYIRVGPDEETWIASLGILLAPPSGILVGPVIDAFGRKKGLLFFFLCMGLGFAVIACATEVYHIYIGRCICAFAVGLEVVAVVYLAEISTKRQRSGFFSMMSVVFSGGVTLTYLIGGYLPWYIASAIFSAGCFAYFAVVCFAPESPAWLFKTGQIDASTKSFLRLGRSHVGIVAELENLKLSSKEDDEKLEFKAFLEPTVWKPFVILSMYHIFQCGTGVYDILYYTVDFVESLGTSYDPLPVSILLSVARFVTTATLGIYFTASVSRRFATAFSAFWMAVTLAGTGVYTYVYRDTTQKPYDWFPIVCMLINIVASALGVTSLPLLMSGEVFPLRVRGAMTGASFLIGLGALFVVVKIYAFCLQILQIWGLLFVYAVFSVLCVLLGVFLLPETQGKTLWEIEQGFLPKKERRRNGERRTEDTLGSGVIRK |
| *BTST125* | MEFATEQEPKQTPSEKWGRMFWACGGAMMIFFFNGVAESHTAVLLPRLQEPDSPIHINPDQMTWIASLGIVGAPVSGVLCGPCVDYFGRKIVVQCYFIVCALGYALIGAASSVYEIYVGRLILSLGIGFEVAGIVYIAEVSTARMRSVLLSLTYSVLYGGGTLFAYVVGLSLPWNLGSAVFALACVLLFGYESFTPESPPYLVKNGHTDEAIAAFKRLGRSDDQIAQEIRILERKGEPRQQVEWRTFLEPTVWKPFLIISCFHFLQAVTGVWDTLYYTVDLVTNLGTQYDPYEVSLFLTVGRSLMASTAGVYFTTRVSRKMAAAVSTFSMAVSLFILAVYEKMYEFTSELERPYPLLPICALIGAVMASGAGFFFLPMLMSGEVFPLRVRGTMSGAVFFVGTGSMFLFLKLHVFLVTTLGVWGFYAMWTAASFITGFYSIFVLTETHGRELHEIENSYRSKKQKGADIERTSQF |
| *BTST126* | MATLPGKGEAPKGQELEKVAPEKWCRVLWACGGAMMIFFFSGVTEAHTAVLLPRLEEVDSPILIDADEKTWIASLGIVATPLSSVLCGPCVDYFGRKIMVQCYYLVCALGFALIASANSVYQIYAGRLICSLGIGFEVAAIVYIAEVSTVRMRSVLLSLTYSVLYGGGTLFAYAVGLSLPWNLGSAVFALVCLILFGYESFVPESPSYYYKKGDTKKAIVAFTQLGRTEDQIAQEIKILEERKTKTEQKVDWRTFIHPTVWKPFLIIAFFHCLQAFMGLWDELYYTVDLVTELDSAYDPFEVSFILTLSRFLVASTAGVYFTTRVSRKLAAAASSFSMAVALLVVAVYEKRYELTAKWERPYPLVPIVGLVGAVMASGAGMFFLPMLMSGEVFPLRVRGTMSGAVFFVGTGSMFLFLKLHVFLVTTLGVPGIYTMWTTACFVAGFFAVFVLTETHGKELHEIEDSYRSKKHRSTDIERTKF |
| *BTST127* | MISSGKTQSPNERPYQYEYTALGTQDVEKSGEHGTGVSGNRRKVNRFRSAAPQILAVTAKNLVLLDLGMTMAFSTIVVPVLLDPNNKDPNGLSFTEDQATWFASIPMVFQPLGSALSGLISAPLGRKRSLMLVNIPQIIGWLMLYSSSSVNIMYLAAAIQGLGAGFMDAPIFTYVGEICEPSLRGVLISYSLQFCSVGFFLQCLLGSLTTWRHVAFISMLFPTLAFLAISQIPETPMWLLSKNRMKEAEKALCWLRGWVSKEEVAEEFAQLVQYSKNSKYKSDDDKKKLQMDLISTAKQPCGGCTRPPIVPCDSNTGDDDYAKLKLHEKVKDLLRPEILKPMSIIIIVNFLYFTSGFPGFKTYMVLLFQRVHSPIDPNWASVFVSTSIILIHIAQMVAVKTIGKRWMTLISSFGAAVAGLAIGVHMSFQGFFDETFGDLSNWLLFTYFEILTLATVIGLGPVPWMLMSEIFPFRGRSFASGFCAAIYYAASFFAAKTYLSTLNLFGVAGTYYIFGTISALGLVYVYLYLPETEGLTLEEVEDIYRPKKRSEVKNL |
| *BTST128* | MTTDMNFGAKPESRKAILIQIISSVIASSTLLSSGMSLGFSGVALPHMEAPDSLVKVGPQEASWIASLANLATPVGCLLVGPLLDRLGRKNTMIFVGVPAVCGWLLIAVEPSLPRVYLGRLLTGLATGLSSIPSTVYTSEITSNAMRGILVTCSSISIAVGILTEYCLGWWFQRHWHCVALVSGVISILVSGLVLIGIPESPVWLVSRGQNQEASKALCTLRGTKSKNKIEKELNQIIENCRAYRGRSTSIARSISGLALPQAYKPLIIMNTYFLFQQVSGLFVIVFYAVDVIKIAGVTADAYLIAVLIAFLRLVTIIVSVWVNKAFGRRFASIISGVGITLSMFALVGYCYFVPGAAAPTPVLVNSTTTTTAIPQALVGSTDAPIPMANFSLVENVTVVMSESFQGVHGLSWIPIAALFVHIVFGTIGFLTVPWCMIGEVFPAQVRGVACSITSCFAYLSSFVVIKLYKSMLMSMGTVGIFTFYGIMSLLGTLFVMIYLPETKGKSFEAIEKHFANGSGVPASPEEVSLQTKNSKQPIIRPSRPN |
| *BTST129* | MAEEWTXTAPPKASFLRSFLVAASMFPLYICLGALIGQSAGMLPQLLEEDSTIHINKNQATWIASLPTIGTCMSSAASGYLSDLFGRIRVVQAAYSFFAIGFATMMAADSFMLLALGRFLAGIGMGCYFSGNVYLSEVTPPKYRGALLTLNSVLCSCGLVYVYIVGGYYPWYIAAAATCLISIIGLTLTFSLYDSPVWLVRQNRLKTAAKSLRLVEISSNVETKLRKLQETAENHPKTDFTLKILTEPSVWKPFVMILVLSILQNTSGFCIIIAYTVQFMWEFHSAYDPLHVTVAIGVMRLLAILVSFVLFQHFGRKTIGAVSGFGAAIFLLGVYGYLIFAPRVQLLSENQWIPIVLFLAFIFTSSLGIYPLPWILPFELFPIKVRGMMCGACLCALYLNTFVAVMLYYVLIDNLRLGGTILLFAAGSALFGIFSMTLLVETHRRTLDDIECTFASGRVT |
| *BTST130* | MNEGTANLLLSQMKGATSLIHLSQDQETWVASLGILSAPIAAILIGPFIDAFGRKRGVLLFYLNMGLGWAVIASAREVTQIYIGRIICAFGEGFQACAVVYLTEICTKEQRSVVLACLIALFSGGVLFVSVVNTCLPWPMACSAFSLASFALAGAECFVPESPAWLFSQGEEAAAVRNLQKLGRSKAGVLLEIDALKERESCTEVLSWRTFLRPTVWKPFLILAVFHLLQFSTGFYDMIYYQVDYLERLGTKYDPIALSVAFSTVRFLSNATIGIYFRSLDRKFSTTVSGLCMTVPLLGAGIYELKYRDTPPLEKPFQWLLLFCIFAQLVAGNLAVTCLPWSMGAELYPLNVRGIMSGATLCVAYSIFFTYVKLYHVAMGALKVYGLLCLGLLF |
| *BTST131* | MFSKFMPFKSKSMSMETGNDRAIKPLICVALLIVFLAGCILGRSEDPRDEDDPYNRLKNHHDPKTFGDILYEYIRDIVKVPVIATVFFCWVCGSFADEHGRVGAMQLFFMLSGIGFGFLVYAQEYDFSILGTFILGAALGCSIPAPIYIAELCPVAYRSFFLGLVPVALSLGMFTVDVIELRGAEDTAWKSLCCFSGIGFLLSLFLHEAPEWLVMRNRPDAAIESLKWLKETSVDVDVDLRKLQETSMAANHRSDTTLEMLTDKRVWKPFAMLLGLALFQHLCGFYILIFYAPYLVNQYRTNIYWFSSYTGTDFLLLVATSAALVFHANLPRRTVAGLSGIGSSAALLGLFLHAHLFVAPQDLLDPTPDKDMLVPVFFFTLYIFSAVMGIYTLPWILMFEVFPLRHRGILCGLSFSTLYLGLFAFESRLNNYLLTGMDLQSLLCFFGTCALGFALFARSCLVETHKKTFEEIERGFTKERIFLPIDEKM |
| *BTST132* | MTDKKPQKSAHHNYKIVSTTDVELNQALGKNDSERDGSDESLPKAVSRFRSALPQVLATTAKNLILLDLGMTIAFPTIVIPTLLDGKDPSGLTFNTAQASWFGSIAFICQPLGSVLSGIVLEPLGRKRSMLLVNVPHLIGWILFYNAESLSILYITCALMGLGVGFMEAPIITYVGEISEPALRGILTSYSGIFVSVGFLFEYTLGNFVDWRTAALISAMVPVITLIAISQVPETPTWLLSKGRNEEAKKSLQWLRGWVPVGLIMDEFDQLKRYNEATRYHAQVTSHTVSSPDVNEKPRPVSYANDVSIDDELTKPDMKTNGNAYSVNGGVSPVGAVKGRKLSFEEKFYDLIRPEMVRPLGLVVVFFFIFYSCGPPGMRPYMIKLFDKMNLPVTGKRVTVIMGLIGILGNIFCMICVKWCGKRPLSLVSTAGSAASIIILGFCALDAVNGPAAVPGAQSVKWTPFVLFCSLWFFSNFGLSQIPWMLTSEVFPNRGRGLASGIAAACSYLMAFVASKTYPDLERYLGIHGVCFLYGTLTLLGYIFIYFCLPETEGRSLAEIEGIYSTQQKAEKKAPSS |
| *BTST133* | MLAFVFTGMVEAQSAVLLPQLKDKDSRIPVTPEEETWIASLGILTSPISAIISGPIVDMIGRKKGLQFFYVNIGVGFGIIACATEVWHLYVGRIICAFAVGLEVVAVVYLAEICTKKQRSAIFSVMFTLAATGVLLTYVVGGYLPWNIASGIFSLACFAYLIIQSLAPESPAWLFKTGRIEASIRSLQRLGRSNSGVLREVDLLKLSTQEKTEKFEFRIFLQPTVWKPFLILSIYHFLATASGAYDIMAYTVEFIAALGTSYDPLAASILLSVIRVIVNATAGIYFVGSVSRRLATALSALLMTISLLGTGLYSYVYREAAPGSKPHEWVPIALMILNIGAGAIGVTSLPWLMSGEMFPLEVRGAMTGAAFVIGSGFMFLFIKIYYIMLEGLQMWGLLLAWAVPSAMAVAFGVWILPETQGKTLYEIERGFLPQGERMREPPQVAPEQIEIDVTKNGT |
| *BTST134* | MDSFPIAGDLENLIPTRRPSILTFEPGVAKVLPQYLATIIVTIGGFICGTIIAWSSPAAVKLENGEDGFPVDENDMSWIGGIMPIGAILGCILTALVVDILGRKNTMIVVVLPCTIGWSLIVWADSVLMICLGRFILGTTCGSFTIICPMYTAEICQKEVRGTLGTIFQLQVVSGILFLYILGSFLSLFHLSLICMVLPTVYLVFICMIPESPVYHLKLGRIDEAKQSLQSLRGPNYNILTELVDLSALVDSSSETEVIPFSIAIRSPAAIKGLIIGLGVMFFQQFSGVNAVIFYAASIFKDAGSSFSYNVSSIIVGSVCVAFTYLSTLIIDKLGRRVLLLFSSVVMTGCTCGLGVYFYALSHNYDMSHVQIFPILSVCGFIVAFSLGFGPIPYMLISEIFSPQIKGTASSIVCLFNWVCCFIVTKYFCILSTRFGSDVTFGAFSFLSFLGIFFVYFVIPETKGKSMEEIQSHLAGSDDS |
| *BTST135* | MSEESDVLGEPTSPRRSNVESGMLSKKGMSTLRVVILGLIVIVPGVAPGMSFGFPAVALPQLNLNIDEASTFASLGAIAMPIGCLLSGPVIDRYGRRTALMLINLPSFMGWLLIASKPHLTRLYVARLLTGLAVGLATTPAAVYSAECLTVHKMSLRGSLTTWSTVALTSGILLVYFTGALLAYTTVAIIASAISLLSLVLISLFIPESPTWLIDQGRFDDAENADKILKIHRKRTASECSQLIPKDSPKEKKEEFFSMETIRKTVQDFREPEAYKPLVIMITFLFFQQFSGLYVMITYMVDIISSAGVEVVNPYLVTVISGVVILIAAISVTFLLPIFGVRKLSMFSCLGVSVSMLTYGMYLSVRSKVPWVSNYPIFGLIPVFAIVLNVWMSGIGFIPIPYSMLGEVFPPHVKGTAGGIASSLSSIFCFIAIKTYPYLFLNLEAGIFYLYGTLALFASLFVIYYLPETRGRTLEEINSSFSSKKKSEYEQH |
| *BTST136* | MGTQCVMPTIVLGALRNNPDEELSLNDYDAAWLGSILFLCQPVGSIVSGFLCERFGRRGSMALINIPFIVGWILLHYAASVTGLFAAALAMGMGIGFCEAPIAAYLGEIGEPHLRGSLLSIMISATSFGYLSTYFLGSIMPWRTFALVNLIYPVTTMILFTQIPESPVWLVHKGRLKEAQKALGWLRGFLQPNQVQEEFDRMVKHIESSKSASPTEKKLNDFGEVTSSERSGIIGKLLLLRDPMLFQPVRLIFLTFVFTQCMCLQAFKPYLVGILKTFQFPVNPKWVLIIIGLMSFVGSTMPLFIFRFTGKRNLILYNQFICCVTVFGLGIYCSFFNDTLSSDSPWRWLPIVFFAIVFFSSSMGIMNVPWMLMGEVFPIRWRSFATGICGAWAYCVTFVTARLYLPMESVLSLSGMFYLYGAIGILGFFYFYFFLPETEGKTLETIESYFTPYHDKKEKFSRPKR |
| *BTST137* | MDAPLACLMDDTKEKLVVGPPHAQIKVAELTKSTPEIRRKKGSSLRQIGAAVFANLGTINTGLVFGFSAVALPQLTRPDSEIPIDENQASWLASMSSVSTPCGCILSGYLMDLIGRRRTLIVTEIPLIIGWILIGMAPNIWWMYVGRLLVGLGVGMVGAPSRVYTAEATQPHLRGILAALASVHVSLGVMIEYILGYYFSWSSMAFLNTLVPIGSLGACLLLPDSPAWLLSRGRFEDSKRSLQRLRGATCDVEHEMGMLVAFAQTNATGSPGSAKQTLRAILHPSARKPFLILMVYFAIYQFCGINPLTFYAVEVFQHSGSDWDKNVATIILGVVRLVFTIVGCLLMRRVGRRPLTFLSSIGCGVPMLGLGYYMWLKDDWISNGVTPKFQWFPVLNIFAFMAFSSIGYLVVPWVMIGEVFPAKVRGIIGGLTTCGSHFMVFVAVKSYPLMQKVLTEAGSYVFYGVISLLGTVYFYACLPETKGRSLQEIEDFFSGRRESLAPDAKRRIVNNNNNRPTILKPQKGKILP |
