# Supplementary material for: Genome-Wide Characterization and Expression Profiling of Sugar Transporter Family in the Whitefly, Bemisia tabaci (Gennadius) (Hemiptera: Aleyrodidae)
Source: Front Physiol. 2017 May 23;8:322. doi: 10.3389/fphys.2017.00322 (PMC5440588; doi:10.3389/fphys.2017.00322)
Supplement: Supplementary file 9 [file Table9.DOCX]

**Table S9. Amino acid sequences of the *B. tabaci* non-sugar transporters involved in this study**

| Genes | Amino acids sequences |
| --- | --- |
| *NON_BTST1* | MPRCIPRHIFNQIVSSITAFLTLLLSGIWLGWMSAVLPKFRGGEIPIPMTTDDLTWTVALMDFGNLLSPIPTGYLMDRYGRLLTLRLAMLAFVAASALVLAASAPYHLFLARLLAGVGKGVGFTAATLYVAEIAGAKIRGALSGVFIVMLMGGTVVSMTVGPYVSFTTLNVITAVCPVVGLFLTLFIVESPYYHLIRDDPAAAGEAFARVRDQSKGAANEAEFALVKRKVAEDMSGQKSILNLFTEKGNRRGLIIILVQGFLQRSGGISCILAYASTTLPSDSFWQGKISVMVFSWIMVVFGLVALSLVDRFGRKPLHLISCVGLTLVTGVSAVYYYFYQKTEVDVSQFMFVPHVGVVLFGVFYPVGVGQIPHTLQSELFPTSVKGQASALMTMALAISSFIVNKVYFAVDRGLGVYFMYLIFALSNFLSMVFTAFYVFETKGKTLEEIQHFLKK |
| *NON_BTST2* | MVIGFPAILIPAVTNDDNADNLHLTMAQASWCASLSFIFQPVGGIMTGLCLQSLGCKAVMILLNIPHIVCWLMTYYASSIYTLCFAQAFFGCVLGLIEVPGLRYVSEISEPSVRGIIISSTSFFVSVGYLIMIFIGSLTDWRNAAAISASLPLLCIILLILIPESPMWLMSKGRSEDALRSLQWLRGWTSAQMVHEEFQRIQFYSKNKTQKFLKTDHSGDSRSGSGGLSYFTTSTFLKPLIKCCIIFAIFDFGGMISFRSYLVKILQDLHSPVSSKWSSLWVALFGILGNVGCMLFIKKVKKKPMLRISLLCCILCLIFLTMFLFGYMQVIADSAFHHWCPLIMIVALFFFYNLGIHPIAWAFLGEILPYRGRGPATSFVVCSHNLFTFVSVKTFPNLTQWLGLEGALLVYAGVCLCGILFTYFLPETEGKHLSDIEIEVACGGKTNEDGLKATPCGS |
| *NON_BTST3* | MELHLDTCPDGRSQQCQSSSLRQTLAQVLAATGKSLIMLSIGMLIGFPTVLIPVLTSKDYKGDLHFNRDQASWYGALTYIFQPVGSIASGALLQSFGCKKLMILINVPQIGCWLMIYFATSNFVLYVSSALVGLVIGLMEAPTIRYISEISHPSLRGILTTYSVLFTSLGFLAVYSLGSLTDWQHVALISAAVPVICIIILFQIPETPMWLMSKGRAAEALEALQWLRGWTTADMVEDEFAKLEYYAKKKSCKTLHGYGKSVDTKDEVKETPAENGIDGVSGGGKVSVKPSVSFQVEVGFEKRGFADKLGDLTCKEMTVPLGKCIVLITLSCFSGLPLIRPYLVAIFAQLNLPIQPNWTSVLVTFLGIAGNIGCMALVNRVGKKPLVICSSAASAVCLILLSIFLMQSTPEAMQDRANPNWWPLILFFVLFFSFNMGLQPLPWVYLSEILPYKGRGIATGIAASIFYIVIFFGVKMFSTMERELGLQGTFLLYAAVCIAGIFFTYFILPETEGRFLSDIETDAEREITVISNKGNTVNA |
| *NON_BTST4* | MSCSSGNAVYAALPKHGDPVEIENFDVERPSTKTEFIHSKRDILYQGFTSLVAFSHVIHAGINLSFSTVLHPQLDITKEQASWIASLGAVGTPIGSISIGFIMDRIGRQKTLLATAAINILAWSTLCLSPAQVDIKMIYLARLLEGIASGMTSIAIVYVAETTDKHWRPLFLSMNSIALSGGILLTTTVGVVLAWRAFAVFCLGVSTLSALLSLTLPESPSWLAQHHPERAKQALARLVTDPRAFEIEWTSLEPKAPKLLKTRKRPSLDKKIIRPLAVVLILITLQQLVGIYPTIFYSLELFQKINKRAQQSTEFNSSEETTMIIPTTPHIVTTPSSAQLFNSSSFEKSTGYPLNLDVFNTSALIDHQHNVSAFIRGTGNPHDTRAFIQRTSLPQDAKVVINGTKHELGDELGEIKSKFDLRSGAVQALMGLGIIRFITSVSMTALSKHLGVRTLLMSSSLGVGLTAILFSAYQAGWCSRSSDAVSFAIVLAFITFGSLGLLVIPWTLVGELLPFEFRGVGQGIVVAYAYVIMSVVVKSYPSVEDELGTSAVFGFFGAVAFLTIAFIYYFVPETKGRTMDEIQVYFQKR |
| *NON_BTST5* | MENKRSDEYTALKLKNFDNNVQIVKHEFPDSESSDTDHRFRDGLYQSFVSLGVLSLLVHPGICVTLSTILYPQLNITKYQNAWIASLLSIGMPIGSLFVGPMLDKFGRKRTCIFTAILTSITWAMIISLPEDFDLNLLYIIRFISGIALGLATSVIVYVSEVTNKHWRPVYLATVSVFLASGVLVATTVGIMMPWRSFALFSFGVSLVNITLILLVPESPHWLVRFSPEQAKRALVKLNKNKQDFNEEWEALEARRSARASSTFRKARLLSREVRRPMLRLAMVFTFQQLGGGYLVVVYALQMFQTVVQSATTTSNHAPTPAPTNVTLPLVQKHEFRLGDLDTLCFICVGVIRVGMSGLAVLLTKHVNSRPILISSALSSAFSAFAFAAVMSGVFGPVRDLLPLFFVLLFLLLQSYGMLVIPWAQIGELIPLSYRAKGGGFMTAYAYILMFVAVKIYPYAVDWVGLASVFVFFGLVAALASVYVYLYIPETHGKTFKEIEDFFK |
| *NON_BTST6* | MEYDNECTSLRHEENSARIVKGAAEKNGAVTSDHTWRDGFYQGFVSLVALSMLIHPGVGMGFSTILHPQIKHLVSDEQNSWIASLVAFGTPVGALSSGPLMDKFGRRSTCILTCGVAIASWSALVFMPPEFSLLLLYIARILSGVAGGLTSAGVVYVSEVTNKYWRSVFLGLASVLLSTGVLLVTSVGYWMHWKSFSVFCLAIAVLNLLLLLTIPESPHWLIRTRPEKAKRALMRLNKNIESFEEEWQSLDEQFRKKQKAQMEGTPRPSLSSRQVYVPFLIMGVTFTMQQLCGVYPIIFYALEVFQAITGDPGEISPSANSTNPSTETTTLASLIGNSTLMATDGSRQDLKVKSLIGVGLIRFVMSILAVSLSRTIGRRPLLLSSCAGSAISGFAFSLYFFGCFGQEVNDLVSMSLVLVFLLFSSYGLLVIPWAQIGELIPSSHRAKGGSYLISYAYFLMFLVVKVFPFTMETFGIGGLFMFFSIILTLEGFFVYFYMPETLGKTFLEIEKYFATGVDSNGKGARSKWPIDKV |
| *NON_BTST7* | MFPGLESVALVYLVELSVKEYRSLLLGATAAIYTVGILISNVVGGYLPWHLASGIFSLTAFGFGVVHFFAPESPAWLYKSGRPDAAVRSLQALGRSPASVRAELQLLELSARTVSENVSVAVLLEPTVWKPLVIVSLLLVFQAFTGGYQINSYSEDIVQRLGTKYDPLHVSNIMSVATAVTNCTLGVYCISYMRRRPATITLSILVTLASLGAGIYELLLRGAPGPFDWLPIALLAANLSLGSVVTNISWILSGEVFPLRVRGSTTGAIFFVGWGSQSLAIKLYYASLAALQVSGLCFAYAAGSLCTVPLAVFALPETHNKTLYEVEQSFKRRESKDAETPVQDESNL |
| *NON_BTST8* | MKSALKELHRIWESGIGRTVAATVAAHLNSISVGMCQGYSAVLLPQLTSHASPLQVSNDEASWIASLGVISNPVGALLSGVCMEIFGRRTAVQLTSLPFLIGWTIIALSQTLTTLCIGRFISGMAIGMASACYVYVAEISQPEHRGILSSTGPVFVSLGVLIVYSLGSLCSWQFVSAVCAAAAMLSFSAMQLVPESPYWLASKGMTKESHAALSWLRSSAHVEKDISELVNNSRDISPRVSTLKLISDRFNDPCVWKPFFILVGFFLFQEGSGIYIILYYAVDFFRRAGSTVDHNVASIIVASLRFAMSIFGSLCIQNFGRRTLAVTSGILMALSIGAAGVYEHFFEDFAPADRPYPWVPLACILTNVCASMLGLLQLPWLMIGELFPLKVRGIMGGVVSSLAYLFIFATVKIYPNLMANLQMSGSMFGFAIASLMVVVYALMFLPETRGKTLLEIEQRFCDIPKTNSTENLEKGFYINPAISVSTVCAIVENIKK |
| *NON_BTST9* | MYRRFKDITMSAKKELDLAVAADGDQRPKAAPNKEEKPLISSKAKFSPFFRQFLAASGPIIATLSSGMTAGFSAVLLPQLKSPNSTLKIDHDQASWIASMAALPMALGCIFSGVLMERYGRRMTQLLLCVPFLLGWVLLSLATTVWHLYVGRFLTGFSVGLLGPPSIVYIAETAEPRHRGALLATVTLAISVGILLSHVLGTFLYWKVAAAVSVFFPCLSFGLFWICPETPSWLAIKGYTSEAEEAFHWLRGYSDQAQGELKVILSKKPASRSDSEEGSFKRLAHSLRSIFSLSFLKPFFIMNVFFFVQQFSGVNAVAFYSVDIMKTVSGNVDEYLATIVIDVIRVVMSLATCILLRQFGRRPLGLISLVGTTVSLLSLAAVLKTPFYKEYPSLSWLPTGLLASYICFISIGLVPLPWVMTGEVFPAAHRELGSGATSFFGFFVFFVVVKSSPFFFSTLGMVGTFQLFGGITLLGTVFIFCFLPETKNKTLEEIEDLFSKSKPKPSPESAEVV |
| *NON_BTST10* | MHNQAEPSTSLDVPEQNVKDHFQYANATKSAWAQILASIMQNWLFIEIGLELVMPTVILGSLHNNPAEPLNMNDDQASWFGSIPAFCHPIGSLMSGLLQDKFGRKGAMMLVNIPIFMGWMILYFAESIHALYIVSVIMGLCTGLAEAPLHAYIGEIGEPRMRGTISTISTSCCSIGVWLMFLFGYLFDWRTVALVSSSCSIITFTFMTQLPESPTWLTLRGRLDEAKKSLCWLRGWVSSAEVEPEFLSLVKYTVKSARLSQENSAYSSLPIKEGEPVAKRGGFLKEQLKELTNKRTFRPLRLMFIVFIITDIAWVHGIKPYFVKELRMLESPIDPNLALMIFSGLFIMGAMVNVAFLRRFGKRRIALFSHILAGICILSIGMYASFLQSLTQYPLRVWLPLMLWFIIKFLHGFSIITLPWQLVCEVFPLSGRGTATGLAAAWTHIVMSVLTKTYLYMEAWLGFSGVMYLYGVCTMAGVVHHYFYLPETEGKTLEQIETYFTKNHDKREKFSVGKLQRQRAQSGSPF |
| *NON_BTST11* | MGDSPKSSEREKLIKRYGYSSRSDFAQISATLIQGFILINHGLFMAAPTLILGALYEHPEDELYMDDDEASWFGSIPYICTPLANLASGLLQEMLGRRGSGLLSTMPMFATWILLYSANSVTTLYAVAAMMGLSVGLSEAPLNSYLGEISEPHIRGTLVTIASSAISVGIIVLYALGSFYDWRTTASIISVVPVITFILMTQIPESPAWLIGRNRLEDAKKSLSWLRGWTSPNEVEEEFADLVSYTRHHVEDDEGALAKVAKMNGLLMTQVNAVFSKKVLRPLRLVLISFAITFLAAISGMRPYSINELNAMNCPIDPKLILMVAQVLFIGGAVVNVSFLQRLGKRKIALFSYGVAAVSIIGIGAYCSFYRDLSSYSSAVWLPPSLLLVINFLGGLSILVLPWQLMSEVFPPAGRGLATGISAAWTHLVVSALIKSYLSIKSRVGMDGVMYLYGGATALGCVYFYFCLPETEGKTLEQIEACFVDQPDTEEMLSVGASPVRHPENTRLRGNRKERGYGSTARSEL |
| *NON_BTST12* | MSDPSPASGGPSDEKSSLTTKRQYGYSSRSTYAQALATIIQGWILIDHGLMMGASTLILGALHGNPNEELNMDDAQASWFGSLPYFCTPLANFASGFVQEALGRRGSGMLVNIPIFAAWILLSFADSIATLYVVAIIMGVCIGLSEAPLNSYLGEIGEPHIRGTLITMTSTAISTGMTIMYGLGVFFDWRTTALISSIFPVLTFALMSQIPESPAWLISHNRLDEAKKALCWLRGWVGPDEVEEEFQNLLMYAKKSAKEAKASEKTDDIDSKTDGLLMTQLKALTNKNVLRPLRLVLISFTITFLAVVSGMRPYSINELNALNSPVDPKIILILFQILFILGAVAYGSFLRDFGKRKIALFSNGIAAASIIGIGVFCSFFLDASEYPYLVWFPSILLLVINFLSGFGLYALPWQLMSEVFPQAGRGLATGISAAWTHLVVSALIKSYLYIKAWVGLSGVMYLYGATTFLGFLYYYLYLPETEGKSLEQIESYFTDDPDLEEFFSVRRSPATDAEKIGLRSNKEDHSYGSTA |
| *NON_BTST13* | MRDPPGPSVSLIDDAVDVDEDVPVPTNTRYEYSNRSTFAQVLATIVQSWLLIDNGLMKAVPTLILGSLHDNPNEPLDMNDDQASWFGAIPYICTPITSFASGIFQEKFGRKGSMILVNIPIFVAWILLYVAESIAAFYTVAVIMGLSIGLSEAPLSCYIGETSEPHLRGTLATIMSTAMIIGYFIMYTLGYFFDWRTAALISSAFPIVTFVLMTPIPESSTWLIGRSRFKDAKKSLRWLRGWVTAEAVEEEYQSLLSNNRDPIRKKPSESTLQERGPEQEDYGFFRTQYQTLMNDNIMLPLRLVLITSFIGYVAVLRGMTPYLIGELNALSTPIDAKLVLIITQILFLLGAAADMMFLQGLGKRKIALFSHAIAAVCLLGIGFYAFHLQASAVYYPHLAWLPVIFLMVINFLGGFSLQVLPWQLMCEVFPRVGRGLATGISAAWTHLVISLLIKSYLYIKAWIGLGGVMYLYGTITAFGVVYFYLHLPETEGKSLKQIETYFTSNHDRKEKFEVEK |
| *NON_BTST14* | MSDPSEHSKSLCTGKNPEKTSSRYGYSSRSAFAQVIATLIQSWLILENGLLFGAPTLILGALHGNSSAEGLRMNDDEASWFGAIPSICTPLASFASGYLQDRFGRKGVALLANIPILATWLLLYTANSIPALYIGAGMMGLSQGLAEAPIISYTGEISEPHLRGILSTITSTAVMIGMIIMFVLGYYFDWRTATLISAAFPMITIAVMTQIPESPTWLLGKNRLDDAKRSLCWLRGWISNEEIEEEFQNLVNYTRNSAKEDCSATSNGHVQDSCNVKSDGFLKTHYNILTRKNVLRPLRLVMLTAFFTFVAVLVGMRPYYINELKALNSPLDPKLLLIIGQFLFIGGAVTNMAVLWMTGKRKLVLFSYLTAAISILGLAAYSSFLKDSATWVTWIPIVLLCVISFLSGLSILILPWQLSGEVYPPVGRGLATGISAAWTHLVISALIKSYLYMKAWVGFSGVMYLHCGCVVIGFVYLYCNLPETEGKSLEQIETYFTKNVSRREKFSISKATNKSKSLRGDQRL |
| *NON_BTST15* | MSDSQETISAHDEVPLVPRNTRYGYSSKSTYAQVSAVLIQSWLFIDLGLQMTMPTLVLGALHRNPDAAPLDLDDDQASWFGSIPDLSLPLASLSSGLFQDTFGRKGSMMLVTIPLFSGWLLLYSARSITTLYAVAVIWGLVGGLCEAPLMCYMGEIGEPHLRGTLSSISTLATLTGSFMMYTLGYFFDWRTAALVCSAFPAITFVIVTQMPESPSWLIARNRLDDAKKALCWLRGWVEPHEVEEEFQDIVNYTRMSTGVETILDDSDQSIEGNSLGKKDGYLKTQYKQMTDKKILRPLRLIFALFSICGVASVAGNRPYLIGELTELGVPINPKLVLIGALVCFTLGAMGNVIFLRRFGKRNIALVSHFLGALCIFGIGAYCSYAKVFTPEPQLRWLPVILWFTLQFLAGLSIILLPWQLVSEVFPLTGRGLASGIAGAWAHLSSSILVKSYLYTESLISLSGIMYLYGCGTVVGLIYLYLYLPETEGKSLEQIETYFTDQHDRKEKFSIGRPSSFSHASP |
| *NON_BTST16* | MTDTVVPSRGSDVQHGKSTSNHQTKSSYRSGFAQILVALIQSCLIFDHGLEMGIPILVIGALHRNSSEALNMNDDQASWFGSILNIVHPVASLTSGFFQEKLGRKGSMISTTIPLFGAWMTLYFAQSVYALYAVVLIFGLCRGLTEAPLHAYTGEIGEPLLRGTMSTISVSAAIIGASVVFALNYFFNWRSVALICSAFPIVTFALLTQIPESPTWLISKNRLDDAMKSLCWLRGWVEPNKVETEFKNLVNYVRNSTEQNESSSNGSDQTKKDGFFAKGTRYLATNYKIMTSRKVLRPLRLVFIVVVVSLTAFLAGIRPYFIREIQELKSPIDARLILVSSTGSLFIGAIMNVAFLRRFGKRKIALFSHAVAACCIFGMGAYSSFLRDAGSYVQLRWIPIIFWLLLNVVCGLSISVLPWQLICEVFPVAGRGLAVGISAAWAHVVMGLMVKFYIYTEAWLGFSWMMYLHGAGTLIGLTYYFFYFPETEGKSLEQIEQYFAGNYNREENNSIDRRMKY |
| *NON_BTST17* | MSSPEKPVKIQPIYQGCETNKFRYKNASRSTLSQVVATLIQNWLLIDLGMQLVMPTIVLGAIHNNPAEDLSMNDEQASWFGSILFFAHPIGSMVSGFLQEQFGRKGSLILVNIPIFAAWSTLYLAGSIYMLYFVSLAMGLSVGFCEAPLHSYIGEVGEPHLRGTLSTVTSAACILGMLIMYIIGYLVHWRTAALISSAVPVITIIFMTQIPESPTWLIMKDRLKDAQKSLSWLRGWVEPEEVQEEFQELLSYTKISPPPYQANDIEIEKYELVRTDENGREVAKEKESYLQAKFRELTDKKLLRPLRMVFIVFVFCYASSLIAMRPYMVGVFNEFGFPMDSKLILILTSAFFFVGSILNVVLLRRLGKRRLTLLCQGMASVSIVLLGVYCSFFDRTNRIPSLVWVPISLLVSISFFSGLSVALLPWQLLSEVFPLKGRGAAGGISAAWAYYVGAVMSKTYLYLERWIKLNGVLFFYGAISLIGFWYFLRYLPETEGKSLEKIESYFTKNHDKKEKFSKPKRSKKPVSPL |
| *NON_BTST18* | MMPAADGSAPRTSSKWFRTFLAVTGAIGIEFIAGTIEAQSAVLLPQLEGSKELPITKDQASWIASMGTLLCPLTSILCGPLMDILGRRLVFKIYYSVSTIGYLIIAFAKEVWHLYIGRLCLAFSLGFTVANVIYLPEITTTSQRSLVLATINPLFSLGLLFSYVVGGYLRWDVASLIHTLICGLGLFSVLFLPESPAWLVKEQRPEEARNVFRWLGRNAAKIDGDISRLQTTGNGPVKRSIPLKQLRHATVWKPFLILITFHFLQTMTGIYNIMFYTVEFFRDLGTAFDPVLVTIGFAFSRFVVCVTVGYYFTTKCPRRVATAVSGFGSGAAYLVAVAYEVWWKGDRRFQWVPVAAVLVSPGSPVDYDRRGVPALRARVHGRRDILRRERVPVPVHQVPLRAGRGPGDARRDDLLRGGVPRVGRLRAPRPPGDPGQDHAPDRARLHGQEAEAATGEEVPIRQGGGALVRAAQRGSSTARGSARDGGGVRCQTTINGMEMLHV |
| *NON_BTST19* | MGVGTYRQLVAGITASISVMCLGIAMGWSSPILQKFATERPSPILPVPTEDQLSWMIAFMEFGNLFTPVITGILVDVIGRQKTLVLIGPLFALSWLIIYLSQTIYFLYVARVIQGLGCGVVYTAVPIYLGEISDPKVRGALSNLFQGFMYIGLLYAYVLGPFYSYSNFTLFCMAIPLVYSISVLFVPETPYFLLMQNKDTLARKVLRELRDSTDDINEEMRVMKESVEKEMEGAKNPDKNRKITNIKLFLISQFFGSCQIMTNMYAILTYSSMVFDKGGSHWLTPDQYTIFLGVVTLVSTVPSSFLVDKLGRKPLLVTSAVTCGVLELLAGVYFLLRERNAIQGDDYGWCVFVFVSGLSFCYSFGLGPIIPTIQCELFPTNARGLAFGLTILITSVTAFLNILQFQWFASSPKLGMSANFFFGCVMCLLVAEFTVAVMPETKGRTFAEIQLLFHKDEGVERVSVLEAGEGDGPGSRLNPEEREQDEGAGVGS |
| *NON_BTST20* | MLIPRCHLRQYYAAAIASLSAMMTGAAMGWPSPVLEHFCEGAHCEVRMTAGEASWVLSLIEIGNLFSPIPCGYLVDMYGRQPCLFATGPLFLGSWTLIICSRSVGYLYLARLVQGLGMGIVYTVTPMYISEIAGADVRGRLSILFVGLLNLGILLEYIVGPFVSYRTLGYISISVPILFIATSIWLPESPYFLLMNDKSKEAINALMWLRSDYSKDRIFEELTLIKDEVEQEKGTKTAAKNTFGDIFSSAANRKAFLIVQIAACADVLSGMTAILAYASVIFAAPHNTTMEAEDYPIMLGLIMLLAIFPAAYLVDMAGRRPLLIFSCLFSGLFELVAAFYFYAAMKLDCDVARLKWIPLLAICAFSVAYSMGLGSLVPTLMGECFPSQIRGPASSMTSITLCAISFLVIKFFQVVNEEIGLYFNFFIYGVSSIACSAVLWLVLPETKGKTLSEIQQDLKASCKPSKPSLPLYVNASVPKGVEAS |
| *NON_BTST21* | MFVKLQRGVVNQIAAAFTAELAGYNFGVWRVWPSLSITELRAGSAGFAVSDDQLAWITSLLYLGFLLTPFFCSYLVVRLGRRTILYLTSALHTVSWLLVVLAQNPYHLYIANFFGGLAGGVGMTMVPVYVSEISSVNIRGALIGSFLVFINLGQVMMVNMGIWLSYQEVNLFGLGGASVAFVLQFVVLTESPFYLLASKREEKATEAYKRFHASSGEDKIETEVSALKAAVEKDMEHKSSYMELLACKRAFIILVVESFFQNLGGANSMLAFGVISLPKTELFLTPHQTILICVISGTFFNCISSSIVDSVGRKPLLVISVGMCSVFTGGLCVYFFLVEELQVDSLEYEYIPHLLLIGFIASYTSGFAISRSLIIGEFFPTNTRTHAGLTSTFCFATAGFVVTLSFLHVVRLIGLYFMFFLFFLVNFTNFWFSLFFFVETKGKNLFEIQEYLKSL |
| *NON_BTST22* | MTPRKPPVPSPNQFFISCENVQLMQTEWSDETTNNTSTNTDDIDRPPASIFKPVLATLASTACQFFLGAMLGQSSTMLPQLKAEGSSIRITDEQATWIASMGVIGTPMSSILCGPLTDKLGRKRIILVFLFLSAVGHALLGYSSNLTEILIGRFLLGTAAGFGFPSLVYISEISTPKHRSLLLSSATISASLGLTYVYSVGGSMPWDRASLITASLSLLALVYACSIPESPAWLFQHQRRQETIESLKWLKGQHCNIEVELKLLEASCNHQKKRALALSQLTKPTVFKPFLVLSCLAFMQNGTGFYILLYYSVDFLREFKTTMDPQKVSIALALTRLISCTVASLFIKRLKRRTVGIFSGLAMAGILGVIYLSLTAFREAVGSGPVPVLSLLAYIFACSLGVHPLPWLMIFELYPLSVRGLMCGLSNSVCYLFTFLFLKLYYVMITNLQIHGTILLFLCSSAAFGLFSAFLLPETQGKTLLEIEQGFMSKKDRAARRSG |
| *NON_BTST23* | MEADKLPVSEGFIRPFLLSACIYPLHIVGGAIMGQSAGMLPQLLNKDSSIPIDMEQATWIASSTAIGTCISSAISGPLSDMFGRMRVVQMSYFLMALGHALMMAASSFTGLVVGRLTVGLGLGCDFASFIYVSETVPAALRGVLMALYTVMCSLGFIYIYVVGGYYHWTIATGINAITATSGLALSFFLYETPVWLVRQGRLKAARKSLRQSGIAASNLEAKLKELQDAAENKSTETFSLGDLLGPTVWKPFSMVCIMAVLQNMAGFYIVISYSIQFMAEFHSGYSPVQVTVGIAVVRLIAMTLTSFWMRHARRRVIGSVSGFGSSACLLAVFAFLHFGHLAPVLVENQWILIALFFAYIFTMTLGIYALPWTMPFEIFPMKVRGLMSGMTYVSQFIAMFVSVKLYNVLMDNLHLQGMILMFAVGSALFGSFCVTWLVETHRRTLDEIEAEFAGKSKVYT |
| *NON_BTST24* | MEAEPPASAPTGCLRPFFVAACLFPLHICVGSIYGQSAGMLPQLLEKGSSIPIDRDQATWIASAPTLGACLASAISGTLSDVFGRMRVVRLSFFCMALGYAVMVAAESFMLIVLGRFLAGAGIGCNFPAFVYVSETAPPAYRGLFLSLNALMSSLGLVYIYSLGGYFPWVYAAGATCLMATAGLVLTFFLHDSPAWLVRNGKLEAAQKSLRRIEENAANVEIKLKELQETAKNEPTSSFNLRIFIEPTVWKPFMQVLGMSVLQNIAGFYIVIYYTVQFMSEFHSTIGPLEVTVLIAVVRLVGISVASAWMRHAGRKFIGAFSGFSTAVVLLAIYAILKLGDRVKFLAENNWILIALFLLYVLTMTLGIFPMPWTMPYEMFPIKIRGMMCGVCFCAMHVVMFVSVKLYNVLLDNLELDGMILLFAAGAALFGVFSASMLVETHRRTLDEIEAVFAGRPIVTPPQKQKT |
| *NON_BTST25* | MAESGDRKESLSWRCWSRTLIACSGAMMAFVFSGVTEGQSAVLLPQLKDEASPIHLTPEEETWIASLGIVLSPVSASLTGPITDAFGRKLGLVVYHIIMGIGFAVIAVAKEVWHFYVGRCICSFAIGLEVAAIVYLTETCSKEQRGLLLSTISPAFTIGVVVAYVIGGFLPWNVASAIFAAGSFLCSLGQLFGVESPAWLYKRGHTEASTRALRRLGRTQAGIRQELELFKLTVKEQSQKFHLRELLHPTVWKPFIIMTIFHLIHCATGVHHIVYYTIDFINRLGTTYDPLTVSIAISVARTIATCTIGVYFTSYVKRRFATILSGTLMTILSVGAGVYVYVWRDTAVDRRPFQWLPVACVIAYIVIGRVGVTPLPWLMSSEVFPLRVRGSMSGATFVIGTGSIFISIKMYEDLIAAFHIWGLLFIFGTACFSAVLLAVFVLPETLNKTLYEIEQYFMPKKGKKSGEQVDSTSRGEVIDSSPFKAVVKNRFLRHSLRTPCLSIIFQRFIWQINRY |
| *NON_BTST26* | MFSATPGVRRQFAAAITCSIGCLIAGLMVGWPAPTLKKLRQPDSPVHLTPHEEAWVVNAMYYGTILSPFPSGFLINLIGRKTTLLVLAVFPTLSWILVYFSTSATMLMVARLFSGFWLGGIQTVVPLYTGEISEPHVRGIFGSFFQVSSFVGNNFSFIVAPYVTIQTMATICGFFPVLFIILFVFCPESPYYYAMKKNSKAAGKSLSWLRGDEPIMKELQTIQTSVDKELKDDETFTQKLTSIATDPANRKGFIIVETLDVMQRLCGISCMKAFSSIILPPKLGPLTTDHCTIIIGFVWMFSSLICTGLIDKAGRKPLLYASSLGIFVSMLWTGIWYYLNDNTDIDVSSFKWLPLAGFLVFGVTFSFGLGPIVKIYQGEMFPNNLKGMASALTAIIAAFASSVSTGMFPILTETIGMYANFLIFSTVGLVNFLFTYFYVIETKGKSLQEIQAAELNGERPVVTQSNGDSEKAV |
| *NON_BTST27* | MATLPGKGEAPKGQELEKVAPEKWCRVLWACGGAMMIFFFSGVTEAHTAVLLPRLEEVDSPILIDADEKTWIASLGIVATPLSSVLCGPCVDYFGRKIMVQCYYLVCALGFALIASANSVYQIYAGRLICSLGIGFEVAAIVYIAEVSTVRMRSVLLSLTYSVLYGGGTLFAYAVGLSLPWNLGSAVFALVCLILFGYESFVPESPSYYYKKGDTKKAIVAFTQLGRTEDQIAQEIKILEERKTKTEQKVDWRTFIHPTVWKPFLIIAFFHCLQAFMGLWDELYYTVDLVTELDSAYDPFEVSFILTLSRFLVASTAGVYFTTRVSRKLAAAASSFSMAVALLVVAVYEKRYELTAKWERPYPLVPIVGLVGAVMASGAGMFFLPMLMSGEVFPLRVRGTMSGAVFFVGTGSMFLFLKLHVFLVTTLGVPGIYTMWTTACFVAGFFAVFVLTETHGKELHEIEDSYRSKKHRSTDIERTKF |
| *NON_BTST28* | MEFATEQEPKQTPSEKWGRMFWACGGAMMIFFFNGVAESHTAVLLPRLQEPDSPIHINPDQMTWIASLGIVGAPVSGVLCGPCVDYFGRKIVVQCYFIVCALGYALIGAASSVYEIYVGRLILSLGIGFEVAGIVYIAEVSTARMRSVLLSLTYSVLYGGGTLFAYVVGLSLPWNLGSAVFALACVLLFGYESFTPESPPYLVKNGHTDEAIAAFKRLGRSDDQIAQEIRILERKGEPRQQVEWRTFLEPTVWKPFLIISCFHFLQAVTGVWDTLYYTVDLVTNLGTQYDPYEVSLFLTVGRSLMASTAGVYFTTRVSRKMAAAVSTFSMAVSLFILAVYEKMYEFTSELERPYPLLPICALIGAVMASGAGFFFLPMLMSGEVFPLRVRGTMSGAVFFVGTGSMFLFLKLHVFLVTTLGVWGFYAMWTAASFITGFYSIFVLTETHGRELHEIENSYRSKKQKGADIERTSQF |
| *NON_BTST29* | MLTLTLGIRRQLAAAFACSLASLIAGCVLGWPSPTLKKLREPDSPLHLSTYQEAWVVNALYYGTVLSPFPSGYLMNKLGRKMSLLVLCVFPTLSWILIYFSSSAYMLMLARLFAGFWTGGTQTVMPIYIAEISEPQVRGVFGTFIQLNIYLGTNFAFLVGPYVSIQLMAILCGILPVIFFVLFGLCPESPYFYTMEGRHAAAADALTWLRGDAPVDAELRTVRHSVEKESANQSGVFRRIADLVTVPANRKAFIIVETMNALQRFSGISCMMAFSSVVLPETGALNSDHCTIIMGIVWMVSCLGTSGLIDKAGRKPLLYVSSIGIGVSMLWTGVWYYLDENTTYDVTGWNWLPLAGFLAYGCTFSLGLGPLSSTYQGEMFPSNLKGQASAITTITTALASAISTGLFAVLSKNVGVYMNFYIFSAVGFINFFFTYFYVIETKGKSLQMIQAELNGEEIIKPEMKRLGKSVKK |
| *NON_BTST30* | MYTFACSGIQRQLAVAFTAAFSQFLIGFLMGWPAPTLKILRHPSSEVHLTPSEEAWVVNAMYVTSFLSPLPSGVLMDTIGRKTTMVVLCLFPIISWILIFYQQTGLMLLIARAFAGVFVGGVQMLSPVYAGEIAEPRVRGIAGALIMVHGFAGAISVYIIGPYVSIRTMAVIGGAFPIIFLLLFTLCPESPYYYIMRGRQKSAEEALTWLRGGAPVKQELDIIQTAIEKETQSGKGYFTKMLSLVTVPGNRKAFFIVEVMNFMQRFSGLSCLTVFSTIVLPERVGPVTSDHGTLLMGVCCLLASLGCIALIDKAGRKPLLYFSSIGIFFSMLPTAFWYYLDRETSTNVKEVNWIPYAGFLSFAATLSLGLGTIAPAYKGEMFPSDLKGQACALTSIIVGIASALGTALFPVLTSHVGLYANFLLFAAMGLVNLIFTYFCVIETKGKTLQMIQAELNGETLEKI |
| *NON_BTST31* | MYLFKLNLGVERQLAAAFVASISIFTVGMLLGWPAPTLKLLRQPDSPLHLTPSEEALIVNALFFGTFLAPFPCGALMDHVGRKTSMLTLALFPILSWAVNGFAGMIFVYIVGPFVSLPTMAMIGGIFPVTFLVLFLHCPESPYYYIKRGLHADAGKALSWLRGGASIETELASIRTSIEKEAKADRGLVKMLRLITNPANRRAFIIVQGIACLHRSTGIPCIVAFSTVLLPSHIGALTNDNCTIIMGVALLSASLCCSAIVDTVGRKPLLYLSSIGMFTSMLPTAVWYYLDRETSTDVSGVNWIPLAGLLCFAVTFSVGLGPIMQIYAGEMFPTDLKGHACALATMNQAVSAVIVTQIFVALTVYVGLYANFVLFAAMALVNLGFTYFCVIETKGKTLQMIQAELNDFVQPLLLTRSTVRRSQ |
| *NON_BTST32* | MTSFIYGTLVGWSAPTLKKLREPDSPIHLTPGEEVQMINAIYAGTLLGTFPCGALMNRVGRKGSLLLLSAFPITSWSAIYFARTASTLLIARFFAGVWGAAAMTIRPIYVAEIAEPRVRGAAGAFTMVAMFAGTIFVFVVGPCVSIQTMAVINGVAPPVFFLLFSLCPESPYYYIMRGRHADAAKTLAWLRGGAPIESELTSIQTSIEREAKAGQGYFKKMLSLVTVPANRKAFFIVEVMNFLQRVCGLSCMAAYSTVVLPQRVGPFTADHCTLIIGIVWFLSSLGCSTLVDKLGRKPLLYVSSIGILASMLPTSAWYYLDKETATDVTWINWVPLAGFLLFGVTVNVGLGSIAPTYMGEMFPSNLKAEASALTIMAVSVSSGVSIAVFALLTVHVGLYANFLVFAAVGVVNWVFTYFCVIETKGKSLQLIQDELHGGTRWKPPKNNHSDGKLTV |
| *NON_BTST33* | MLNFIVVSGIERQLAAALISGFSCFIAGALTGWPAPTLKKLREPDSAIRLTPSEEAWVVNALHITTILSTLPLGSLMNTLGRKTTMLVLCVSPIVSWVLVYFARTSFVFIVARSIGGLWLGGCQTLLPIYIAEIAEPRVRGIAGSFIMVNAFAGIIFVFTIGPYVSVPLMAVINAACPCVFFLLFLFCPESPYFYVMRGRYEAAGRALTWLRGGAPIDGELNIIQTSIENEAKEGQGFYKRMLLLITNPANRKAFIIVEVMTFLQRFSGLSVLNSFSTVILPERTGLLTADHCTLIMGIVWLLASMCCSALIDKLGRKPLLHISSIGIFASMIPTAVWYYLDRETSTDVTRVNWVPFAGFLVFGFTVSIGIGPIAAMYPGEMFPSHLKAQASALSNMVSSISATLSTVMFVTINAHIGLYANFLVFAAVGIVNLVFTCFCVIETKGKSLQMIQEKLKHGTWKESGVNRETPETH |
| *NON_BTST34* | MLNFITGGIQRQLAASLIAGFSCFIGGSLMGWPAPTLKKFREPNSAVRMSPSEEAWMVNALYIMSMLCTLPIGAVMNRIGRRTTMLVLCASPTISWIMIYFARTSFVLIAARAIAGFWLGGCLTVLPIYIAEIAEPRVRGIAGCFMMLNAMVGMLSAFAIGPLLSVLTVAVINLVYPILFFALFLFCPESPYFYAMRGRHAAAGRALAWLRGGAPIEGELTIIQTSIEDEAKAGQGYVTRMLLFITNAANRKAFFIVEVMNFLQRFSGLSALAAFSTVILPNRIGPLTADHCTFFMGVNWLVASVCCTCLIDKVGRKPLLYLSGIGIFASMFPTAVWYYLDRETSTDVTRVNWVPFAGFLLFGFTMDIGLGCIAPIYTGEMFPSNLKAQAAALSNMVASISSTLSTALFVVISEKVGLYANFLVFASVGIVVFLFTYFCVIETKGKSLQMIQEELHCKSRKKSETDREKSVTS |
| *NON_BTST35* | MLTLTFSGTQRQLLAAFISTISLFMLGSMMGWPAPTLKLLREPDSSLHLTPSEEAWVVNALYFTTILSPLPSGALMNAIGRKATMLALCVFPTASWALIYFGRTASVLLAARVLAGFWVGGCQTIMPIYIGEIAEPRVRGIAGTSIMVNAFLGTIFVFIVGPYVSVPTMAVMNGVIPPVFFLLFSFCPESPYYYVMRGRHADAARTLAWLRGGAPIESELTSIQTSIQTCIEREARAGQGYFNKMLSIVTVPANRKAFFIVEFMNFLQSAFSTVILPAHAGPLTADQCTLLLGAAWLISSLCCSALIDRLGRKPLLYFSSLGILVSMLPTAVWYYLDRETSTDVREVEWVPFAGFLLFGLTFSAGLGSIGPAIAGEMFPSHLKGQASALTTITAAASSTLSIALFSALDARVGMYANFLIFAAVGPVSGVFTATSGDYREAVEFQRASVLRACLNGDFSGKFEVMRRSRNNRKLPYSASGAAGSSPPGKSPAEKRSRNMTEILHYLSEHR |
| *NON_BTST36* | MSFRRVHGVRKLQPAISRDDAAESEHYGKMVFACGGAIMIFFFNGVVEAHSAVLLPCLQEPDSPIQITKDQETWIASLGIFAAPLSAILCGPFVDYFGRKVVIQCYFLTSALGYGIIAAATSVIHLYIGRILCSLGVGFEVAGIVYIAEVCTKRQRSLCMSLSYSTFTAGILFTYVVGAALPWNLGSALYALLCLLLFLYEWFTPESPPWLVKKGRSDRAVAELQRLGRTETAIAEEIKVLRLTCQEESNQRVEWHTFLQPTVWKPFLIIALFHFLQAATGMYDLLYYTVDFIDQLRTDYDSFKVSMGLAIGRFLMTSTVGSFFTTKVPRKLATAISGFSMGGTLLVAAYYEYLFDGVAPGQRPYTWVPILAVFASVMVSCAGVLHLPWMMSGEVFPLNVRGAMGGAVFFVGSWAMFVFLKYYIFFMETFKVTGTLLLCAAASIVTGLFGVFVLTETQNKTLQEVEDSYRRKPRKEIDVEKTGL |
| *NON_BTST37* | MFLTPGVRRQLAAALTCSLCCLITELMVGWAAPSLKKLREPDSPVHLTRHQEAWVVNAMYYGNIVSPLPSGFLINLIGRKTTLLIVAVLPTVGWILIYFSTSATMLMVARFLYGLWTGVIMTTLPIYTGEISEPHVRGVFGSFFQICNSVGSNLSFLVAPYVSIQTMAVLCGSVPIMFIVLFSQCPESPYYYVMKKRPDAAARSLFWLRGGKPVSEELQVIQTSVANEEKHGSRCINLSVPATRKAFVIVVTMNILQRLSGISFMKAFSSVVMPRVGILSPDHCTIIMGVVWTVSSFICTALIDRAGRKPLLYASSLGIFVSMLWTSVWFYLNDKTDVDVSHLSWLPLAGVLVYGCTFSFGLGPITKLYPGEMFPSDVKGQAAALTVMIAAFSSSVSTGLAPILNEHFGVYSNFLIFSLIGLINLLFTYFCVVETKGKSLQLIQAQLRGERLDEIRDSDASEKHA |
| *NON_BTST38* | MCLALGIKRQLIAAVACALATLVGGMINGWPAPTLKKLRQPGSPIHLTTEQETWLVTALHVGTLLSPFPAGFMMNKLGRKASLLALGVLPVISFGLIYLSTTPEMLILARLFAGLWIGGSHTVVPIYIAEISEPEVRGILSALNQVLSFLGNILIYAVGPYVSIRSTAVFCGAVAVIFLVLFASCPESPYFHIMRGHPERAVTSLIWLRGGPPTPAELGAIRGYLAARGGHGWRRITDLLTTPENRKAFVIVEVLSGLDRLSGISCMKAFSSVVLPAHLGPLTSDHCTLIVSLVWMASSMVCTALIDKTGRKPLLYISSLGVAVSMFWTGMWYFLSSQTHIDVSSFSWLPLAGFLVYGCAFSVGWAPITHTFQGELFPSHLKEQASAMTTIVTALTASFSTFVFGMVTKRVGVYANFLWFSVVGVVNLIFVYVYVIETKGRTLEEIQAELKGKKREEPVVRASTDSIFYFN |
| *NON_BTST39* | MIATLKLIKEKIFNKTLLAVIIVNSINIATGMGQGFSAILLPQLENSKEFFISQEEKSWLASLGVILNPVGAMAAGVIMQFAGRKYTLIGACIPFFFGWLIIAMSTSLAMLYVGRLLSGLGMGMASAAYVYISEISTTHERGLYSSFGPTGTSFGVLTVYFLGYVADWKTVAWICAATCALNALSICFMPETPSWLVSRQRLPDALRSLVWLRRNELVAKKELNDIVSHAVLESQVTRKQRTILSILRKATVWKPTVILVAFFILQQGSGIYIMLFYSVTVFQEIQSVLNPFVDSIIVSVVRLLTCIIGSAFIQVISRKRLVILSSFGMFFSMLSLFAYGNIASDDATARLVPWFPEMCLLINISFSMFGTLQLPWIMIGELYPLAYRGIMGGLISSVGYALIFLHVKIFPAISTVMNIYSIFLVYGLFSLVAIVFGKVYLPETKDKELHEIEEIFKKKKGEVQKVDEKPNQTPIFICNPKLPFVPMQEQVVLSAPQV |
| *NON_BTST40* | MSQGDKMERGQESITMIPRGDVREEGKKLPQYIAAITATLGAVAIGTVLGWSSSASPFLKGEITNVTSTIDPPLSVDESARVESFVAIGAIMGALPAGYFADLLGRKTLIAALTLPFLLSWIMILLAKVAWLLYVARILAGIATGATCTVVPMYISEIAELSIRGTLGAYFQLMITLGIFYAYVYGYLVRFAVLNILCALIPIAGFFMFMFVPESPKYLLMRQKKQSAEKSLRWLRGNKYNIKQEIETLQNEIAKSSRTKVSFKDLVATKVAFKSVNIALGLMVFQQLSGVNAVIFNMNAIFMASGSTIEPAICSIIIGAIQVIVTFFSSILIDKAGRKILLLISLGVSTLSLGVLGYYFHLKNSGEDVSGIGFIPLICLILFIVVFSLGLGPIPWMMSGEILAAEIKGLASSLATALNWTLTFVVTRSYAPMEKTLGTDVTFWLFACICAIGFVFVVLIVPETKGKTVDQVQQLLAGKKPARKNGLV |
| *NON_BTST41* | MSTPEKQPPPSYQSSEKGRFHYSKKSTYAQVLVALLQNWLLLDFGMMLAMPAIVLGSLHNHPSEELCLNDAQASWFGSILFFSHPLGSLCSGFFQEQFGRKGSMMLVNIPIFIAWITLYFAESVYALYFVALTMGLGIGFDEAPLHSYIGEMGEPHLRGTLCALLCAASFFGTLVMYFIGYLVPWRTAALISSAVPIITVVCMSQIPESPTWLVMQGRLKDAQKSLCWLRGWTEPEVIRAEFEQLCSYAKISPNPDQEKNPEKEKYELVPGNDNEKVSPEEEEESYLKTKFKELTDKKLLLPLRMVFIVFIFCHATQLTAMRPYMVGILDEFGFPVDSKLVLIVTGASVFAGTTMNMLFLRKFGKRKMTLTSQGMAALCILLLGVYCSFYDRSNRILSLAWMPVALMLLASFFGGLSLALLPWQLLSEVFPLKGRGAAGGISAAWAYYVSFAMSKTYLYLEHWIKLNGVFFFYGGITLCGFLYFLRNLPETEGKSLEQIESYFTKNYDRREMFSKPKRSKKVPFSA |
| *NON_BTST42* | MCIPEEPSRQSREMGPFNYDRRSTYAQVLVALLQTPMIFNFGMMLSMPTIVLGALHNRKSEALWLDDDQASWFGSILFATHPLGSVCSGFFQTYLGRKGSMLLVNILFFIAWITLYSAESPLVLCIAALTMGLGIGFDEAPLHSYIGEMSEPHLRATLCALLVSCSWAGALLMYLIAYLAPWRTVALISSAVPIFTIICISQIPESPTWYVMKGRLNDAQKSLCWLRGWTEPDVTRTEFEQLCGHIKKSSNSDQEKPQAEEKYQAVPGNDNEKESVGKAESLFKTKFRELTDKKLLLPLRMVFIVFIFSRATQLTPMGPYMIGILDDFGFPADSKLILFVAGLSGFAGTLMNILLVRKLGKRKISLTSQGTVAFCMLLLGVYCSFFDRSNRILSLTWIPISLLVIAGFFAGLSMALLPWQLLSEVFPLKGRGVGGGVSAAYAYYVSFAMTKTYLYLEHWIQLNGVFFFYGGITLYGVWYLLRYLPETEGKSLEQIETYFTKNYDKKEMFSKPRGSAA |
| *NON_BTST43* | MAIDKGKLRQYLAGFIASIGSACFGVAMGWPAPVMWALRDPRGRIRMTAEESSWMVSIMELGNLLSPIPGGVLADRYGRKFVLHLAAPLFAASWIIVLLSKAKLMLYAMRVLQGLATGLVFTLTPMYLGEISKKEHRGTIGSMFSVMMYVGSLYAYVFGPPFSYDVFAMICLAMPTVMFFGFMFIPETPYFYLMVGDLKAARKSLAFFRSKDDPIEEELLLMQESVETDMANKSTFMDLMTEVGNRKALIILQVLSMFKIMTGICALLTYATMTFEETGTRCDANLVSISFAVVIVVSTIASAGFVELTYATMTFEETGTRCDANLISISFAVIIVVSTIASAGFVDRCGRRPMLLISSAGLTLTNVLIALYFYELRLLPPLSDYAFIVFAAVGALACFHTIGYGAVHSTIQCEYFPSNTRGLANGITAVTLTVFSFFTLKIFQSIDTYFGMYVNFIVFAAFCFSASVFVNEVVFETKGKSFSEIHAEFRNRVSEPYVDEHHVVTV |
| *NON_BTST44* | MDTPTIEAQPVSVFTKLHFQKSESSVSTSKADGMKEIEAAKPEDSPREVFTLKDEEVDESKAKTGTQYLAAAIASLTGVMMGQMLSWSSPVTPLLIKEGKIDKIEESWLVSILNFGAVLGCTAAGSVNSYVGRKSVLLTACLPQMASWLLLALCSDIRLLCLGRFLGGLCVGFFCVTSPLYISEIAQVSVRGALGALFQLSVTIGILTTYTLGLLPTATSITLASSSTVVLFFALFFWMPETPVFLLRTSQSNRAATSLRWFRGPAYNLIPEMRLLERMVQKADSAAAYSDFVTDPASRRALVVALGLFFFQQFSGINAVVFYMNTVFRTAGGDVSPTVATIVIAALQVVGTALSVFLMERAGRRFLFLASFSACTLCVFALGLFFFLKERGHPVAGPWQWVPLGSVGLFLVVYALGAGPVPWAVVGELFSKRMAALAMSLVTGVGHWASAFVVTKAFAMLEAWLGIGGTFWVFVGFCLVGIVFAWALLPETKGRPLQEILDELGGKKKGVKSET |
| *NON_BTST45* | MMAETNDNMDLPATNKVQANTTEDSTTNSRPPPCPQEVCDTEKGDGSRTGPQFLATGIVSLTGFLMGEMVSWSSPVTPLLIKSHRITKDEESWIVSTVNFGAIIGCLLAGYVNKYVGRKTVLLSLCVPEMISWLMLAFCEGAILLCLARFLSGLCLGFICVTTPLYIAEIAQPCVRGALATFFQLFIVIGILFTFILGILQDALWITLGCSIVVLVFFALFLWMPESPVYLTMVSRPKAAAASLQWLRGRDYDIYAEIRVIEAVVQEGRDVEVTYGDFISDAASFRGIIIAMGLFFFQQMCGINVVIFYMNTVFETAGSTISPTLATVIIGIVQVLATALSVYMMDKAGRRFLFIFSQAACSLCLISLGTYFFLKSRGDDVTPIGWLPVASVAVFLVMFAFGSGPVPWAITSEIFSKNIASLALSLVTAVHWLLAFFVTKVYTSLEAFMGTGPTFWMFAAWCWVGVTFCCLLMPETKGRPQEDIVDELRGCKKKQTSGNCP |
| *NON_BTST46* | MAPQDTLFGSGEKKKEYDDSVDEVKSLDLETSDSEQSVNMDNMRTALPQVLATLAQSLLLLSLGMIIAVPTIVIGAIYKAKEGLSLDDDQSSWFCSILLIVQPIGSLLSGYVQEVVGRKISLVVVNIPQLVGWYLMYAATTVDMLYWSCVTLGFSIGFMEAPTLAYVGEISQPRLRGMLSCITNSHVPLGHLVEFFIGGYVAKDWRMAMAISAVFPIISILAISQVPESPVWLLTKGRKADAMKALCWLRGWTTPECVRDEFEGLVRYVEASRLQNENQQKAAGKNYVQVPTAGYVNADGKGTTPAKVPAENSYKFKVTIGEKIKDLLRPAMLRPLVLVVSYFFFYNCASLNAIRPYMVPVFQKLRLPKDPHFVAILSAALQVLGGLVCIATVHKLGKRCLSLISMTLCAVACILIGIYAVLIERTDFDCPWFPFIVLLALYFCCNVGISPIPWMLISEVFPSRGRGAGGGVSAALFYIILSIISKTYLDLESIVTFPGVFFVYGLVACAGVIFIFLCLPETEGKTLQEIEDYFTRSRKKGINNLSV |
| *NON_BTST47* | MTEPDSTPPSSGCCRPFLVISSLFPLYVCVGALFGESAGMLPQLMEEDSLIPTSREEATWIASVPTIGTCIAATTSGSLSDVFGRIRMVQMAYFLLGMGYGIIGAANDFTLLVVGRFLAGVGVGCSFPANVYVSEMAPPAYRGLFLVLNPLLASTGLVYMYVVGVYLPWNIAALFSCLIAFLGLLLTFFCRDSPVWLLRKNRPDAARRSLAQIEGPANVDARLKQLQEIADAQRDAETQSGHKTFSLRVLASPTVWKPYLTVLILSALQNISGFYIVISYTVNFMREFHSTFDPLQATVAIGVVRLAAICVTSALLRHVGRRTIGAVSGFGAAASLLLVYWCLVKPELAPNAWTPMALFLAYIFTMTLGIFPLPWTMPYEMFPIKVRGTMCGVSFCSMYGLMFVSVKLYNTLLDNLRLEGMILLFAAGSLVFGLYSATLLVETHRKTLDEIEAVFAGKRTKKVDG |
| *NON_BTST48* | MEDPYVPPRSDVKKISRFRQILPQIIATSATIVLYLTMGMIIGFPTILIPALTAKNSQDVLHLTMEQASWCGSVGCIFQPLGSIIAGLALQPLGCKKSMMLLNIPLIACWLIVHFATSNYALYFANGLFGCVMGLTTAPGLRYVIEISEPSLRGILVASTSLFISLGFSFIIFLNSLTDWRQTAAISASIPLLCIIILFQVPETPMWLVSKGRSDAALKSLRWLRGWTDAETVREEYEKIVSFTKNQNQKLLKRQWSGKVAEYKNCPTVEEAELAEPASASQGLSERVRKMFKDMTRKEMLIPLTKCCIIFAINCFSGVPILRTYMVKIFDDLNLPVDPKKASVWVALMGMLGNIGCMFVIKKLKKKPLFLASLAGSALCLFSMAADLMGYLEGTVIASFHRWCHLTFAMALYFFWNLGIQPIAWSYLGEILPYKGRGPATSVASSFYFILTFVGIRTFPAMTEFLRLEGVLLFYAVVCVAGLFFTHFLPETEGRHLSDIQAQDKEGAETEKL |
| *NON_BTST49* | MSGNGKRQPVEKSFLGYPGDEVDRCKSTLSQFYATCVQCIFLISLGMQFVMPTIVLGALHNKAVINDAMYLDDADASWIGSTLYICHPIGSLISGFLSERFGRKGGMMLVNIPFIGGWVLLYCATSVRGLYVATLTMGLGMGFCEAPIAAYLGETSEPRLRSIFTTMTTAACNLGVLIELAIGSSLDWRTSTLVSSFVPVFSFVLFFTIPESPVWLITKGRMKDAQKSLAWLRGFAKPHQVQNEFDELVRYTKMSYALGNSQGNPEEKAPLDNKNNTSKHVEEDSDGWLKTRYKEITNPKLYMPLKFVMFSFFWAQCACLIPFRSYMIGILENFWFPVDRKWILIMTGVVAFIGSVVPMFIIQYTGKRKLGLSCMFVATASILCLGVFASFYTHTENLVVAWLLIVDLAVVHFVGFLGIINVSWMLVCEIFPVRARGIATGISTGWSCFIAFLLTKGFLWMESMVGLSGLFYMYGVLSFLGCIYYYFNLPETEGKTLERIETYFTSNHDKKEKYSMPSRSASKA |
| *NON_BTST50* | MIQDSTQLVSAPNSPFFTPTRIKQGWTSLLMFCAFSTIMGLAIPMGYGIGVINTPADVIRAWCNETLQANYDVVLTDKKDLDLIWSVIVSVFLLSGVVGSFIGGWLANLIGRKGAMLVSCLLSTVAGFCFLSPLIVNRIELLFAGRVIVGLSAGLGTAVVPMYLLEIAPTKLQGSIATFFSLGITIGVLLGQILGLNWLLGGETRWPYLLSAYILCVLFCLLTFPCLPESPKYLFSVKNERQSALQALSRLRGLPADLLQSELDSKDTAENNFNNEEIQTWSVAQVLRTRSLLLPLALVIALQAGQQFAGINAVFFYSSDIFKSAGLDETSREYAVIGTGCVNLGVNVIAVFTLKYFTRRFLVLLSCYGTVLSLLLLTLCSHYMQTVSWLPNASIAVVMLYVFMYGVGLGPIPYFIGSELFAVGPRPIAMAFGSFANWGGNFLVSLTFTTFFNYLAGYSFLIFAGSTMLLSIFIHAYLPETKNSVASL |
| *NON_BTST51* | MVVATGDFVSTSRDSFIRPLLASVPVFWLQLLTGSIEGHSAVLLPQLEESEKFYISLEEESWIASLGIMATPLVAVLSGPMVERYGRKFIFYIFYILCTLGFSFIGLAQRVEHIYIGRILGAGAHGLTLCSILYIYEICVAHQRNKLLPLLCPMCSAGILYAYVIGGYLPWNVASLVLAGSGVVGLICIFFIPESPAWLVMQGDINAAIQSLEWLKRDKETIAQEVDELRKSSTSKDLPQSISLQHFLHPTVWKPFLILLIFSALQNGSGFYMLLYYTVNFFQNLGTGGEIDPLTITVGLALMRLVSGSFGALFIARFSRKKLTATTAFGMFIVASAAVAYLVTFGEDPTNRPHQWFLVLCSLSYVLLCTLAIQPLPWLMTNELYPLQLRGLMSGITFFCLFTMVFVGIKAYPFFMFYIHITGILCIFAGACLLAVIFAVFFLPETYNKTPYEIEEYFMRRKKKSVHCVNFYEKTCVQIPLKFKEFASYYGEHSLFARKSA |
| *NON_BTST52* | MMAIKKELNLDNPRESYVRPIAAGAILGMQLINGIMEAQSAVMLPQLAAESSAISITQSQSSWIASLGIVASPISSVLCGPLMDFFGRKMILEGYYVIAMLGFLIIACAKKVVHLYIGRFALSIANGFGVGTIVYLPEMCSRDQRSRLAALLMPLFSSGILTAFLVGGYLPWNVASASYTGVCTIGLICSVVWTPESPCWLVNEGRYEEAKRSLRYLRGSDSTNIEAHVEVLKMSRKSVLVERNAVFSDLLEPTVWKPFVILVLFHFLQTGTGFYGLMYYTVDFFDDLRTSFDPLTVTIFLSVARLVMSCVFGTYCATRLNRKVVTALSSGLSGVSLLGAAAYEHVFTSIDADERLHTWIPIGCILTNVLVCTITVQPLPWLMTRELFPLPVRGIMCGLTYFIGTVLVFLSVKYFMSVMGLFGIPGALSFFSASSFLVCLFGIFVLPDTNNKNRSEIERNFTKAKPRVEEQPLIGKGVGNVMPTLLV |
| *NON_BTST53* | MVFACGGAIMIFFFNGVVEAHSAVLLPCLQEPDSPIQITKDQETWIASLGIFAAPLSAILCGPFVDYFGRKVVIQCYFLTSALGYGIIAAATSVIHLYIGRILCSLGVGFEVAGIVYIAEVCTKRQRSLCMSLSYSTFTAGILFTYVVGAALPWNLGSALYALLCLLLFLYEWFTPESPPWLVKKGRSDRAVAELQRLGRTETAIAEEIKVLRLTCQEESNQRVEWHTFLQPTVWKPFLIIALFHFLQAATGMYDLLYYTVDFIDQLRTDYDSFKVSMGLAIGRFLMTSTVGSFFTTKVPRKLATAISGFSMGGTLLVAAYYEYLFDGVAPGQRPYTWVPILAGRAPPPLDDERRGLPAQRPRGHGRRRLLRRLLGHVRLPQVLHLLHGDVQGHGNAPLVRRRVHRHRSLRGLRPHGDAEQDAPGSGG |
| *NON_BTST54* | METFKEQKSPPPENWIKTFWACGGAITILVFNGVVEAHSAVLLPQLQEAYSPIHVDKDEETWIASLGISASPLSAVLCGPCIDRYGRKIVIQGYFLISAIGYGIIAAASSVVHLYIGRIICSLGVGFEVAGVIYIAEVCTKYQRSLFLSLTLPMFTGGILFTYVVGATLPWTMGSSLYALLCLLLFVYESFTPESPPWLVKQGKISRAKAEFKRLGRSDEWIEEELKLLQASCDTLERNHHLDCKTWLEPTVWKPFLIIALFHFLQAATGVYDLLYYTVDFVGELGTQYDPFQVSLYLAIARFLMTSTVGLYFTSKVRRKTATALSGFAMGASLLVAAIYEQRFDGVAPAERAHTWIPILAVSVSVLVSCAGVLHLPWLMSGEVFPLRVRGLMSGYVFFVGSCSMFVFLKSYVFFVEVFKVTGVLLLCAAASVLIALFGLFVLTETQDKSLYEIERGYEKKSRGDADKTNLREVE |
| *NON_BTST55* | MKLMIGDEEVCVKIGNDGKEELSWRCWLRTMFAASGAMAVFVFTGVTEAQSAVMLPQLKQKDSPIQISADEETWIASLGILLTPVSAILAGPLVDAFGRKKGLQGFYIIIGLGFGVIASAKEVYQIYIGRCICAFAVGMEPIAVIYLAEISTKRQRSLFFSLMAAMYSGGVTITYVIGGFLPWNVASAIFSLGCFAIFVVQCVTPETPAWLYKTNQVEASTESYLRLGRSHTNILQELESLGLSSQQRTEKFHIRAFLEPTVWKPFLILSLFHIIHCGAGIYDVLFYTVEFVETLGTSYDPLAVSIFTSVARFITNMTVGLYFTASLSRRFATIFSSFFMCLSLFVMGVYEYLYRDVSVKPFDWVPVLFTVVSVVSCSTGLLSLPWLMPGEMFPLHVRGVMNGAAFLVGSACMFVTLKLYAFCMETLQIWGMLLMFAGFAFTGIFFGMFVLPETQGKTLYEIEQGFLPTQKKRENEISPSGKVETIT |
| *NON_BTST56* | MAFVVDTEVACAKIQSSDEDESEKLSWRCWFRTLFAASGPLMVFLYTGVAEAHSAVLLHQLKKEDSQIPVTTDEATWIASLGILLAPISALLAGPVLDAFGRKKGLLSFFLSMGLGFCVVAFAEEVYHIYIGRCICAIAIGLEVTSVVYLAEICTKRQRSCFLSLTAPFFSLGVALVYLVGGYLPWQMAATIFSLSSFGFFVIQCFAPESPAWLFKTGQIEASTKSLRRLGRSHENILHELDLLTLSTRTRSGKFHLRAFLEPTVWKPFLILSIFHFITNAAGVFDVLYYTVDFVKAFGLTVDPLIFTVLLAVARFTTNCTLGAYFLVSVPRKFTTAFSGFIMAASLLGSTVYEYAYRGLAEKPLQWIPVTLTVIAIVASAMGMNFLPWIMPGEMFPLQVRGAMTGASFLVGTFCTFVSLKIYGFYVETFCIWGLLLRFALFAFAGALFGILVLPETQNKTLYEIEQGFVAGNKKIPEAPEQ |
| *NON_BTST57* | MTSSVSDEETRGKISSNEETKDKTVSDTEIGAKPVNDEECGEEVSWRCWIRTLFAASGAMMVFVFTGVTEAQSAVMLPQLKKPDSYIRVGPDEETWIASLGILLAPPSGILVGPVIDAFGRKKGLLFFFLCMGLGFAVIACATEVYHIYIGRCICAFAVGLEVVAVVYLAEISTKRQRSGFFSMMSVVFSGGVTLTYLIGGYLPWYIASAIFSAGCFAYFAVVCFAPESPAWLFKTGQIDASTKSFLRLGRSHVGIVAELENLKLSSKEDDEKLEFKAFLEPTVWKPFVILSMYHIFQCGTGVYDILYYTVDFVESLGTSYDPLPVSILLSVARFVTTATLGIYFTASVSRRFATAFSAFWMAVTLAGTGVYTYVYRDTTQKPYDWFPIVCMLINIVASALGVTSLPLLMSGEVFPLRVRGAMTGASFLIGLGALFVVVKIYAFCLQILQIWGLLFVYAVFSVLCVLLGVFLLPETQGKTLWEIEQGFLPKKERRRNGERRTEDTLGSGVIRK |
| *NON_BTST58* | MGKVEMHSSEKVYRPCEMTTSNVNNVKSTVAQFYATFVECCFLILVGMIYMMPTIVVGALHKTESSNSTLTSPEDEAMRMDDHTASWIGSIVLMSHPVGALTSGFVSERFGRRGAMMLGNVPFLGCWVLYYLATSVKGLFIASMLMGFTIGLCEAPMGAYLSESCEPRFRGISNSMVVAFCTMGNSLELFLGSAFHWRTSALVGVSVPVICFLGFLTVPESPVWLITKGRLEEAHKALAWFRGFAEPQHVREEFDDMVRYSMASSRLSRHDLSAISEEKAPLDGKHNIQDPEKSQSIKRGNWLVERWRELSNPRLYLPLRMVLITFFFTQSAGLVPFKAFIIEILNEFWFPFDNKWAVVATGVASFLGSVGATILVKLAGKRLMCIICMIISTISIFVLGFSASFFRHQEDLLLSWLILSVFAVVHFVGNVGVINIPWMLSYEVYPVRARGMANGISAASGCFMAFLQTKTYLDTERAIGLDGVFYAYGVVALAGCIYVILYIPETEGKSMEQIETYFTPHHDRKEKYRMPSKKNRSKA |
| *NON_BTST59* | MSEKEKLHSTEKVYKPCEYSTENTGNFKSNLAQFYVTCVECIFLISLGMQYVMPTIVVGALHNKVGDSMALDDTTASWIGSILYFCQPLGSVTSGFLSERFGRKGAMMLVNVPFVAGWILLYYATSVQGLCIATLTMGLGIGFCEAPIAAYIGEVSQPHLRGIFTAMTTAACQLGNLIELFVGSVFDWRTSALISTVIPLISLISFTTIPESPVWLITKGKMEEAQKALGWLRGFLEPHHVQKEFDEMVRYARMSNTLSSEPGENFSEKIPLDSEKIPIKEVSDGFFKQRYRELTNPKLFLPLRMIFITFFFTQTASLAPFRAYFVRILDQFWFPIQSRWVLVMTGATAFIGSVTAIFILQKTGKRRVMLFSMAVNLLATFILAIYATFFTHTVDMTVSWILIVTFGISYFVGSLGINNIPWMMLCEVFPVRARGIASGLSAAWSYFVQFVMTKTFLQTESLIGLSGMFYMYALISVGACIYTYLCVPETEGKSLELIETYFTKNCDRKQKFRMLKRGRNPSKA |
| *NON_BTST60* | MISSGKTQSPNERPYQYEYTALGTQDVEKSGEHGTGVSGNRRKVNRFRSAAPQILAVTAKNLVLLDLGMTMAFSTIVVPVLLDPNNKDPNGLSFTEDQATWFASIPMVFQPLGSALSGLISAPLGRKRSLMLVNIPQIIGWLMLYSSSSVNIMYLAAAIQGLGAGFMDAPIFTYVGEICEPSLRGVLISYSLQFCSVGFFLQCLLGSLTTWRHVAFISMLFPTLAFLAISQIPETPMWLLSKNRMKEAEKALCWLRGWVSKEEVAEEFAQLVQYSKNSKYKSDDDKKKLQMDLISTAKQPCGGCTRPPIVPCDSNTGDDDYAKLKLHEKVKDLLRPEILKPMSIIIIVNFLYFTSGFPGFKTYMVLLFQRVHSPIDPNWASVFVSTSIILIHIAQMVAVKTIGKRWMTLISSFGAAVAGLAIGVHMSFQGFFDETFGDLSNWLLFTYFEILTLATVIGLGPVPWMLMSEIFPFRGRSFASGFCAAIYYAASFFAAKTYLSTLNLFGVAGTYYIFGTISALGLVYVYLYLPETEGLTLEEVEDIYRPKKRSEVKNL |
| *NON_BTST61* | MSDTTPSREAPAPTMTTESPANSLDVYYIPNINNEEKNYKSNCKSTLSQVIATLVESLLMVVLGTQSVMPTIVLGALRNNPHETLSLNDYDAAWLGSILFLCQPFGSVASGFLSEKFGRRGSMTLINVPFIVGWILLYYASSVTGLFAAVLVMGIGIGFCEAPIAAYLGEIGEPHLRGSLLCIMCSAVSLGYLSTFFLGSIMPWRTFALVNVIYPVTTMILFTQIPESPIWLIHKGRLKEAQKALGWLRGFVEPRRVQQEFDRMVKHIEASKSSDRPKGNVESQDDGSCESKFTVIKRICELRNKKLYLPLRLVFITFIFTQCMCLQAFKPYLVNILDTFKFPVDSKWVLVMIGLMNFVGSAMPLFIFRFTGKRQLILCNQFICVVGVFALGLYCSFLNDTLDTNSDWRWLPIVLFAIVFFSASTGIMNIPWMLMGEVFPIQYRSFANGLCGAWAYCVTFVTARLYLPMEHVLSLSGMFYLYGIVGILGFFYFLFFLPETEGKTLEKIESYFTPHHDKKEKFTRPKR |
| *NON_BTST62* | MAKNEDEPPTGGFLRPFLIMTALAPIQLVVGSVLGQSAGMIPQLMQEDSVIKIDIDVATWIASMSTVGTFVAASSSGFFADKFGRIRMVQVAYFFLAIGYGIIGAANTFFLLIFGRRLIGFGVGCSFPASVYISEIAPPAYRGLLLTTNPAIASLGLVYMYVLGGYYPWNIASLATGLMSILGLIIAFFFYDSPVWLLRKNRIEDARKSLSRIEGPTNVDVKLKQLQEIVDSHPVSKFSPHVFIEPTVWKPYVITIILSILQNTAGFYIVVSYTVNFMREFHSTYDPLQIMVAIGLVRLVAICLSSAILRHVGRKTIGAFSGFAAAACLLPIYGCLVAPHAAVLRTYPWIPIALFLGYIFTMTLGIFALPWTMPYEMFPIKVRGFMCGVSFCSMYALIFVAVKLYNFLLENLQLPGMILMFAVGSLLFGVFSATVLVETHKKTLDEIEEVFLGRRSKKIQKTTSDS |
| *NON_BTST63* | MFSVSILPFGFGQFATWPSLAIEQLLEGDAGFSVDQSEISIIASTWSLGLCLLPILFGFMLVRQGRRRNLLITAVVYIVAWALILFARSPLWLMAGNFIGGLGSSIQLIIGPIFIAEIADKYIRGALISFYIAAIPLGQAFMCSVGIYVTYFQLNLIALVISTVAFFCILATAVESPSWKLMKQKESEAESCFNYYWNTRNADRTESTVALAELRETVELEMRSKCSYSELVRTPSNVRGTIIVAAISLFQSASGILVILDYGSTTLPKYEGFWAPHPTMAAVSILYFFLSLVSAGLVDRLGRKPLTILSNAGDALGTAIVAVFFALERRTEWDTTNLQWLPYVGMLLFIASYGSAMSAMPHVLVGELFPANVRYHASVLSVIAIAGSLAFFNYTYLGGCRLLGMDVMFFIYTLCSIAATIFSWLFMFETKNLSLAEIQAIMTGRKMASNDPPQELNDLR |
| *NON_BTST64* | MVQLAWLSRNRGRVRQISVCCSASILPFGHGLLVAWPSLAIERLRRGDAGFEVSSGEISIIVSMMSLGMCLMPIPFGYVLVRLGRKTNLLINAVVYAVAWGLIAFAPSPLWIMVGNFFAGLGSSIQLIIGPLYIAEVADKDIRGALISIYIMAIAIGQVFMTSIGIFVSYFELNLMSLIIAIVAFFCILTTAVESTSWYLMVDNEYEAERSFHYYWNTGAGTDRTQSLATLKETVALEMKSACSYVELFRTPSNIRASIIVIAQSVFQSAGGIVAILTYGSTTLPAYDGFWKPNPTMALVSVLNLVFNIVSAGFVDRIGRKPLTIISNAGNALGTAMVAAYFAVERRTNWNVTDLEWLPYVGFLTYVAFYGSGMFTVPHILVGELFPVNVRYHAAVLSTISIAGSCAFFNYIYLGVSQVAGVDVMFLIFTLCSISATVFSWIFMFETKNLSLADIQAKMTRNSTRASDPTPELENRR |
| *NON_BTST65* | MVKDEIEIELSKEYDDSVRCPVNRLGKFRQCLATFIANIITICLGTVNGWAAPVQPQLQSETPPVGRRLSDDEISWLGAITFMGGVAGVLVWARAADLLGRKGAGYLIAAPFLLSWTLLLFCDHYYLLLAARFIAGFGGTGVLVNTPLYVGEIACAQLRGPLGSSLILFINFGYLLAYFFGSVLTYARFNLFCLLLPVVYLALFAYLPETPNYLYMNRREDEAKRSLLYFCGDNARAMNHEFNLIASVTNGGPRVELSDFLRKKSTRRALVIGMVLITGQQVVGINILLTYTVAIFSAAGSAISPNLCSVIVGVAMLIASIPSCYLINRLGRKYLLIFTSTGMSASLLLLAVCFLFDKSNAFVQSTYLPLVSFSTAIVCYALGVGPVPFVLSSEIFPSSVRNMATSLIIAWGIFGSFATVKLYPSMLSLLGYFGTFSLFSVSALCLSLFIHFCVPETKNLSLNAVIELLENHSTLKVGRFS |
| *NON_BTST66* | MASLGIVTAPIGAILIGPFVDAFGRKVGILIFYLTIGSGFGVIALSMDVTQIYIGRIICAFCEGFKACAVVYIAEICTPTQRSLFLSAISTMFSGGVLICTVMSAFVSWNSACLAYSLAAFAFAGVQWFVPESPGWLYRHGKEDEALRSLERLGRSKADILREMDDLKERKSNQEKLELKSFFEPIVWKPFVILSTFHVLQFSTGIYDIIYYQVDFIQSLGTTYDPMTVSVAMSTIRFLSNATIGVYAKSVSRKGSTALCGLGMALTLLATGAYELAYRDTEIPARPYQWLPISLILSCIVASNLSVTCLPWAMSGEMYPLRVRGIMSGATLVVAYFAFFFYIKMYYVFLEALKIYGVLFVFAACSVVVLLFGIFVLPETQGKSLLEVELGFEKKAKRSENVENRNGRVEKGEKSDFVTRF |
| *NON_BTST67* | MLIEDLHVECIIAADVTFSRVKLIVRLSSNYNIECLVEKEYLAGATRQFLILSSISPQNRKLRVQAAPLKTIDDMAGEPEIITWSCWLRTVVAGITALFLLAFAGMNNGASNLLLSQLTKKDSLIPISQDQESWVASLGLLAAPIAPILIGPFIDFFGRKKGVLVFYLIMGIGWAVIGSAKNVTQLYIGRMICSFGEGFEACAVVYLAEICATEQRSIVLAWLRALFSAGVLFVDVINTCVSWPVACLGFSLAAFAFAIAELFVPESPAWLFRQGEEEAAVKNLQRLGRSQAGVHLEIETLRQRESSTESLSWRTFLKPTVWKPFVILAVFHVLQLSTGCDVIIFYQVDFLASLGTTYDPVSVSVALSTVRFLSNITVGVYTNSISRKISTAISGFCMAVPLAGAVIYEYHYRSVPVLDRPVQWLLLTFIFAYLVAAELAVNCLPGTMVGELFPLSVRGTMSGATHFAAHCSYFAYVKFYFACLRVLKIHGILFVFAASSFLAGLFGIYILPETHGKSLVEVEQGFEGKTQEIDQNIVVPLSSINS |
| *NON_BTST68* | MITVASAVMENDTNSCRKASVENGLKTDISNNRRGKRRFRSASAQILACVIQAWLLVDLGMEMATPTLIIGALHKISAEAEPLHMNDEEASWFGSISNMVFLFASLSSGFLQELIGRKGSMIVVNVPRFAGWMTLYFASSLSTMYLAAVVMGICEGLCEASVHSYIGEIGDPRLRGTLASISSHGYFFGTLTTLILGCYFEWRTVVLISSAVPVLAFICLTQIPESPTWLIVRNRLDEAKKSLCWVRGWVSPDEVEEEFQEMVNYVKNSSEESLKAFNSNECAESNAKDLTVFKGVLSTMKAVASKKVLRPLCMVCTAFLTSLAGNVIGITPYMIRELRELGAIVEPKLILVMFQIIFVVGSLTNVAFVRRFGKRRLALLSQGLAVLCILGIGTFCSLAFSSADRSPQLSWIPVALFFFLNFINGVGVRLLPWQLLSEVFPPVGRGFASAISVAFAKLILFTLIKTFLMTEDWLHLSGVMYLYAGVSFFGLCYYYLYLPETEGKTLEQIESYFTKNHDRTEKFRIGNQDRNTLY |
| *NON_BTST69* | MGASMNACTRTPTEDASKTTAKRHPCRSTCAQLLATLIQGWLFLDLGLEAAVPTLIIGALHRNPSATESLRMNNDQASWYGSLQSFCFPIASLSSAFLQELIGRRGCMMAVNVPSFAAWMTLYFAESVPALYVASAIMGLSSGISEASLHSYIGEIGEPRLRGTLSSLSSSGFCAGSLGGFILGYYFDWSDWRTVVLISSACPVIAFICMTQIPESPTWLIVRNRMDEAKKSLCWLRGWVSPNEIEEEFQTLVQYVKNSSAETRRGLRQETSIASTEENSSILKEFILIIKVLASKKVFRPLRMVFITFVISSVACVGGIRPFLIGELKDLGTTIDPKLVLIMFEVIFFVGSMFNVTFVHRFGKRRLAIYSHSFAAIMITGMGVYCSYSSFYEDNSNSQLPWIPVALFAILNLVEGVGISLLPWQLTCEVFPPVGRGLAAGMSAAWSKLVFSALIKSFLYLEVWLNLSGVMYLYAGLTVFVSLLLSTRNGGEKSGADRIILHESSHPEGEN |
| *NON_BTST70* | MTETEKNETSGQEEAPSEPVISNTTYRQIVLALILAIPSIAPGMTFGYSAVSLDSIPANLSQESWFASLAWIATPVGCLASGPIMDNWGRRPALLLINIVGFCGWILLAYASTTLSLYTGRILTGASIGFASAPSSVYVAECIASNSLQLRGILLTWPTVALSTGILLVYIMGSLLRFTVVAGLGAIISVASFFCILFFIPESPAWLLLKGRREDAEVAQRRLGLGKPLSESRVESGEASTSTKLLPSQSELTWSTAWEELKKPEAYKPLTIVIFFFLFQQFSGVLVVINYLVEIVRISGFVLLNPYFVTVVAGFIILICACSVSFLLPKFGVKGLSTISGVGIAISWLIIGLYIFIRRTWLVELQYSLFNLIPLCGIILNVVSSSIGFYPLPFAILGEIFPPKIKGVASGIATCVAYLFSFIAVKTFIYLQLHFYSAVIFFYAVMAAFGVIHVNLFLPETTGKSLQEIVKHFSATKSGYEKI |
| *NON_BTST71* | MGAEDAASAESFLKPFLAALASFMCQFQLGAILGQSSTMLPQLQAEDSPIRITKEYASWIASAGVIGTPIASVLAGPLTDKMGRKSVIRMHFLLSAIGHTIVGVSSDGTEILIGRVILSCATGFGVPSLVYIPEICNPRHRSPLLFTATVSSSLGLVYVYTLGGILSWDITAMLTSSLAIIGLVYTFIVPESPAWLFRSHRLNEAIDSIKWLKGQNVNMELELRSLKDACHEQPKERVSLLKQFASPTVIKPFLVLTIISFLQNASGFYILLYYSIDFFLEFKSSYDPRFVSVGLAVTRLVSCTVASVIINRFCRKTMGTFSGLSMGVILLGILGYLHAFGDDVEVLSRYSWVPAAGLTLYVFACSLGVHPLPWLMIFELYPLEVRGRMCGISNGMCYVFTFVFTKLYYTFIANFKIQGTILLFMVASVLFGLFSAFVLPETQGKTLVEIEDRFRPKKKPDKESTLP |
| *NON_BTST72* | MSLEAEKLEGLNTQNEAVTIIKSRYNYSRRSAFAQVLATLIQNWLLIEIGLDTAMTTMVIGALHLNSAEALSMNDEQASWFGSLPFICHPLASLLLSGYFQDRFGRRTTMILVTIPTFIAWVSLYFAQSMYVLYMVSAVTGMCTGLTEAXLHSYIGXIGEPHLXGTLSSISTSAVXVGIFMMYVFCYXFTWRTVALICSACPVITFTCMTQIXESPTWLIVKNRYEDARKSLXWLRGWVDPSEVEEEFQALVXHARNSXQKNKXAQSXGXGLIKKDSYLKTXFKEMTSKRVLLPXRLILIVFVFREITTFSAIRPYLIGELNKLHTPINAKLILILSEVLVFVGAMMNVVFLRRLGKRKIAIFANGIXXICILGTGIYCSFLQDSTRXPQAAWLPXXXXLMLSLFCGFSATLLPWQLVCEIFPIVGRGLATGITAGTKYLIQSAMVKSYLFIETYIGLSGMMYLYGTGAVLGVIHLYFCLPETEGKTLQQIESYFTKNHDRKEKYSIGKAA |
| *NON_BTST73* | MTQASDETEKLVESPSTTAVYRQSLAALTCCLSCFTIGLSIGWSSPAFHKIQASETSFTLDGFQQSLVVSALNIGIMFGAIPTSFLMDQLGRKKTLLYTATLSLLHWVLIAGAMNAKFLYFGRFLGGIYSGIATAIAPVYLAENLEPQIRGSIGTLFSILLYGGILCTYIIGPIASYMNLSLFCGAFTVLFMVTFAPMPETPYFCIIKNRREDARKSLEWLRGHSNVDAELKQIEAYVTSEQEHVTGWSDIFTDPNLRRPFLVCVALCFIQKSTGFFTIISYQSVILPGMVGPLTSEGATLVIGVVLLMAGTASAFLIDKVGRIILLNLSYVGVVLSMIPTALWFYFNKTDEPADVEYVNHYNWVPFFGFIAFIVCHAMGLGPVGNIYPGEVLPLSIKADAMALVVSLAALFTAINTEIFAFFNAYIGMYANYFSYAVVAVVGALFTRLYIVETKGKSLQAIQEEFIEQAKMPRMKGDYFVL |
| *NON_BTST74* | MTVGAIAGWSASAFPKIRNDELKFRLTLFQEAWVINTYYVGIMMGPLLAGIAMDAIGRKTTLLLFSIFSVANWTLVILASNEYMLYIARVFSGLWAGSVFTVCPAFLAEVLQPHVRGSLGSFLMSMYFLGNLYEYIIGPYVSYSTFGIASCIPCLIFAVAFLFIPESPYYYIMKNQRGKAEASLSWLRGDVDVNQELDAIETYAVAFMRNRGSFKDVFLNENYRAALINVQAIYFLQKLCGMFTVLAYLTVIIPPYVGPFTSENCTLIVGVVLWISTTLAASLMDRIGRKRLLVISNAGIIVTMTITGAWYYLDSTDLDLSETTYVPFLGLVIYGIFFCLGLGPIPTLYQGEILPSNIKARACTVTTMCSAWASILNTTLFAICIRYIGLYINFFLFAATSVFGLYFAKYHFIETSGKTLQEIQEELMKRRHRGKFTDDKKSPVNAKPTIYTVPMPNATEKVETKKHFEKDAKWTDE |
| *NON_BTST75* | MFKISRSIFRQTLAAFCCSIGPMTVGAIAGWSASAFPKIRNDELNFRLTLFQEAWVINTYYVGIMMGPLLAGIAMDAIGRKTTLLLFSIFSVANWTLVILASNEYMLYIARVFSGLWAGSVFTVCPAFLAEVLQPHVRGSLGSFLMSMYFLGNLYEYIIGPYVSYSTFGIASCIPCLIFAVAFLFIPESPYYYIMKNQRGKAEASLSWLRGDVDVNQELDAIETYAVAFMRNRGSFKDVFLNENYRAALINVQAIYFLQKLCGMFTVLAYLTVIIPPYVGPFTSENCTLIVGVVLWISTTLAASLMDRIGRKRLLVISNVGIIVTMTITGAWYYLDSTDLDLSETTYVPFLGLVIYGIFFCLGLGPIPTLYQGEILPSNIKARACTVTTMCSAWASILNTTLFAICIRYIGLYINFFLFAATSVFGLYFAKYHFTETSGKTLQEIQEELMKRRHRGKFTDDKKSPVNAKPTIYTVPMPNATEKVETKKHFEKDAKWTDE |
| *NON_BTST76* | MFIIPKRVRRQIFAALSCCIGPLMVGSIAEWSASAFPKIRSNELGFRLSVFQEAWVINLIYAGIMVGPLLAGIAMDAIGRKSTLLLFTVFAIINWTLVTFAPTKHITLLLFTVFAVTNWTLVTFAPTKHILYLGRFCGGIWNGCVITIVPAFLAEILEPDVRGSLGSLFVMMYFAGNLYENLIGPYVTYRSFCLISSAPVFVFAATFVFIPETPYYYMMKGQRKKAEASLMWLRGDGDVTVELDKIEKYAETFMKQRGSFKDLIFNEKYRKAFLNVQGVYFIQKLCGTFTVLAYLTVIIPKRVGPLAPSNCTQITGIVLLLSTFSSTFLLDAVGRKPLFIISNIGIIVTTSITGAWYFLDGHTDFNMAGTTYVPFLGILLYGGFFCVGVGPIASIYQGEVLPSNIKARASTVTTMISAFASIVNTTLFAVCNRYIGIYVNFFLFALTSVFGLYFAKYHFIETKGKTLQEIQEELMMSYQKRKASALSGKLGVCQVPIPHSVKTATKR |
| *NON_BTST77* | MTEEETKEKLSWSCWLRTMFACSGAMMLFVFTGVVQAQSAVLLPQLKGNDSIIHVTPEEETWIASLGIFMSPVSALFVGPFIDVLGRKKGLLFFYINMGLGFSIIACASKVWHIYLGRCICSFAVGLEVAAVVYMSETCPKELRSIILSISSATLTIGISITYVIGGYLHWALASAIFAVGCFVYVIIQALAPETPPWLFKQGFKDDATRSLQQLGRSPSGILREIKLLEISAPEHTERLSIGTFLDPTIYKPFLIIFAFMFLQVLTGVYHIMYYTLNFVERLGTTYDSLQVSIIIALARMLANLTLGGYSTAFVSRKWATALSAGLGAIVLALAGAYEFLYRSVPVGQKPYEWVPIALVVVNIAASMIAVTPLPWLMGGEVFPLRVRGSMSGAVFVVGSAMMFVFIKIYEELMELLQIWGMLFFYAVASVVMVLFAVYLLPETQGKSLFEIEQGFLPKNKRLSREPEPAGGATS |
| *NON_BTST78* | MTTDMNFGAKPESRKAILIQIISSVIASSTLLSSGMSLGFSGVALPHMEAPDSLVKVGPQEASWIASLANLATPVGCLLVGPLLDRLGRKNTMIFVGVPAVCGWLLIAVEPSLPRVYLGRLLTGLATGLSSIPSTVYTSEITSNAMRGILVTCSSISIAVGILTEYCLGWWFQRHWHCVALVSGVISILVSGLVLIGIPESPVWLVSRGQNQEASKALCTLRGTKSKNKIEKELNQIIENCRAYRGRSTSIARSISGLALPQAYKPLIIMNTYFLFQQVSGLFVIVFYAVDVIKIAGVTADAYLIAVLIAFLRLVTIIVSVWVNKAFGRRFASIISGVGITLSMFALVGYCYFVPGAAAPTPVLVNSTTTTTAIPQALVGSTDAPIPMANFSLVENVTVVMSESFQGVHGLSWIPIAALFVHIVFGTIGFLTVPWCMIGEVFPAQVRGVACSITSCFAYLSSFVVIKLYKSMLMSMGTVGIFTFYGIMSLLGTLFVMIYLPETKGKSFEAIEKHFANGSGVPASPEEVSLQTKNSKQPIIRPSRPN |
| *NON_BTST79* | MAEEWTXTAPPKASFLRSFLVAASMFPLYICLGALIGQSAGMLPQLLEEDSTIHINKNQATWIASLPTIGTCMSSAASGYLSDLFGRIRVVQAAYSFFAIGFATMMAADSFMLLALGRFLAGIGMGCYFSGNVYLSEVTPPKYRGALLTLNSVLCSCGLVYVYIVGGYYPWYIAAAATCLISIIGLTLTFSLYDSPVWLVRQNRLKTAAKSLRLVEISSNVETKLRKLQETAENHPKTDFTLKILTEPSVWKPFVMILVLSILQNTSGFCIIIAYTVQFMWEFHSAYDPLHVTVAIGVMRLLAILVSFVLFQHFGRKTIGAVSGFGAAIFLLGVYGYLIFAPRVQLLSENQWIPIVLFLAFIFTSSLGIYPLPWILPFELFPIKVRGMMCGACLCALYLNTFVAVMLYYVLIDNLRLGGTILLFAAGSALFGIFSMTLLVETHRRTLDDIECTFASGRVT |
| *NON_BTST80* | METQLPPTGCCKPFVAVACLFPLQILVGAIFGQSAGMLPQLLEEDSWIRIDREEATWIASLPTIGTCVAATVSGSLSDTHGRIRVTQVAYFFIGMGFAVMATANNFTMLALGRFMGGLGIGCYFPALLYVSEIAPVAHRSILLALNGLMASAGLVYIYILGGYYPWPIAATASCLLAILGLLLTLFLYDSPVWLVRHDRLETARKSLHRIENPANVEATLKHLQETASNQPKCDFTLKVFVEPIVWKPFLIILALSVLQNLAGFYIIIAYTVQFMREFHSAFDPLQVTVAIGVFRLMAIALSAVLFRYFGRKTIGAVSGFGAAACLLATYAHWKFSSMVALLAENQWIPVMLFLAYVFFMSMGIFPLPWTIPYEVYPIKVRGMMCGVSFCSMYVIMFVAVKMYNILMDNLRLEGTILLFAAGSLLFGVFSVTILIETHRKT |
| *NON_BTST81* | MDKKTAFSIEVLEPVVRKESKKTTQYVAALTATIGGFIAGNILAWSSPAGPKLMDGEYGFPVTEDDMSWVGGIMAIGAIIGCIITGLTVDVFGRKNLMLFLVAPTTIGWCAIIWAESVFILCCGRFLLGAACGSFSIVCPMYTGEIGENSIRGTLGTYFQLQIVIGILFVYLIGSILNTFWMSITCAVIPLVYAGLMGLMPESPTFHFKKGEVENAKMSLQWFRGPEYDINGEIKEMLDIIDRDEREKVPLAIAIRSKAAKKGFVIGLGIMFFQQFSGINAVIFYTTQIFQSAGSTIPPDLCTIMTGVVSVISCYIATVIVDKLGRRLLLLTSGTVMALCCGVLGGYFYMLKHNMDVSNIGWLPIACVCGFNIAFSLGFGPIPWMLVGEIFSSQIKGTASSIACLFNWACVFMVTKFFSVIAEMFGSYSTFWFFTAMLVTAIAFTFFVVPETKGKSFEQIQSELSGENESQSEASTVSAYPSKDLKY |
| *NON_BTST82* | MSKNDRCCGVSLSVYRQFLSAIFCCIGSLSLGLSLGWAAPAFVKIKNGEAPFELTIYEQSIVVGALNIGLMIGTYPAGYLMDRIGRKTTLLYASSFSLINWILIAFASSELYLYVARLFAGLWAGAISTLVPIYVTECSETKIRGSTTTQHLVFMSAGILLGYIIGPQVGYMDFALICGAFTVFFAIVFSFPPESPYFLTMKGRTEEARAALVWLRATDDVDNELKSIEAFIADGTKGRCSYKDLVKNPLYRRPFFICLVLWFCQKFTGFYTLIAYQTVILPKKLGVLTSDNCTQIVGVIILTSVFIASRLMDATGRKVLLTVSHVGIAVFMGIVGALYALNDTGYIQIEDYSYLLVFSFVAYVFSFSIGIGPIASLYTGEVLPQAAKGTAGSVILSLSSIASAGNTFAFAATANWIGMHWNFFFYSALSVASLVFVHLCLTETRGRTFQDIQSNLSVKKKPEMTTIEKSDGSVAHFDGRTS |
| *NON_BTST83* | MMSSIIEVRLHLFVMISFCYFXXGLGSMTVGLMVGWPAAAFPKILRHETPYHLSIFNEAFIISCMNIGSIVGTVPASLLMDRIGRRASFLLFSLFAVASWIFVAYAPTVEMLYCGRLLGGVFVGAYLTILPSYLSETLEPDMRGFLGTSSTLLNTLGTLMAYGLGPRVSFIDLSLISCCVAIVFIISLIFMPETPYLLVMRKDYAGARRTLAWLRGTSESDVTVELTTIQNFIETEKAKASLTTSDLFFDERYKWPFVSCVGLLLLQKSSGYFTVIGNQTIILPHHAWIFYSEDSTLIIGLILVIMSIVAALLMDALGRKVLLQISNVGQASAMLVVGAWYYLSAEQRTELEAYNYMPLLAVFAYVVAFSMGLGPVPHIYIGEVLPPLVKGRATGLLVTLAALFVVSVNEIFAAVTTFADMYVNFLFFGVCSLVGIYYVNGWVIETRGKTLPEIQEEFRHRRTMKDGYYILD |
| *NON_BTST84* | MFFKISKGIRRQLLAAFCCCIGAMTLGAIAGWSAAAFPKIRNNELEFRLSLFQEAWVINAFYIGIMMGPLPAGIMMDAIGRKSTLLFFSTFAITNWTLVTLAYHEHMLYLARFCAGLWAGSVTTVVPAFLAEVLQPNVRGSLGTMYFIMYFAGNLYEYIIGPYVTYFTFGISSGLLCFVFATSFVFIPETPYYYIMKGKRKKAEASLRWLRGDEDVSAELESIQTYVKTFMKRRGRFKELILNENYRAAFINVQAVYFIQKLCGMFTVLAYLTLIIPSQVGPLSPEHCTLITGVVLWLSVFVATSLIDRVGRKPLFVISNIGIIITMTITGVWYFLNVHSDMDLSSTTWIPFSGLLLYGVSFCLGVGPIASLYQGEVLPSNIKARASTVTAIISAFASILNTTLFAICASYIGMYINFFLFALTSVFALYFAQYHFIETKGKTLQEIQEELRKKPGFSRETSVNSNYSTVSSLPAIYTVPIARTHSLEKSAIANSLAKNA |
| *NON_BTST85* | MSRFINRGVARQLAVAFTAGGSCFIIGALMGWPAPTLKKLRAADTPIRLSVLEESWVVNALYLTTMVSPFMCGALMNSFGRKLTLLALTVFPTLSWILVFFSRSGAMLIAARFLAGFWVGGCSTVVPIYVAEIAEPAVRGVVGTFTAVSTMLGIISAYVIGPCVSVYTMAAIYVVTPVLFFALFSLCPESPYFFVMRDQHVAAAAALTWLRARDSVTAELAAIQGSVERDAQTRQGCLRKLFSLVSVSANRKAFVTVEFMMVLQRMSGFSCLMAYSSVILPSKVGPFTSDNCTLIMGIVWLGSALICSVLVDRVGRKPLLYFSSIGIFVSMLPTSLWYYLDRETSTDVSGANWVPLAGVLIFGLTFTMGLGAIPSIYQGEMFSSSLKGIGSALTVGVCAGSSALSVSVFAVLVKFVGLYAPFLLFAAVGPATFLFVYYFVMETRGKSLQAIQDELRGEELDR |
| *NON_BTST86* | MGDDSSEKPPDRISKPLMAAVASYACQFQLGAILGQSSNMLPQLQAADSPIQIDYDSATWIASMDVLGTPISCLLCGPLTDKMGRKATIRLFLLLSAVGHAIVGVASDVTEILIGRFCLGIAAGFAFPSIVYISEISSVEHRTPLLAINTISSSFGLLYVFIVGAFISWDIISLMTSLISVISLVYAFFIPESPAWLFQNHRLNDAIDSIKWLKGDDCDMTQELKQLKDACTEEPKGTGTIFRHFTGVTVVKPFFILLVFAFLQNGTGFYILLHYSINFISEFKIDFDPRYISIGLAVVRLSVCIMASYFLSRVNRKTAGMVSGTGMVVVLGGTLVAMYFMMGDATMSTGYSVIVTVGLLAFIFVCGLGAHPLPWIMIYELYPLHVRGTMCGVSNAINYVFIFIFIKMYYVLILNLQIHGTVILFAAFSAAFAAYSFLILPETQGKSLVEIEQGFLPKKQRQNGSA |
| *NON_BTST87* | MIDKVLNAGQFFEKEGNQHKNACRSLLSQVVATIVLGGLVFDIGMMTTMPTLVIGALHKNSAGELKMNDDQASWFGSIIFFAHPMGALISGYLQERFGRRGSMILVNVPVLAAWLTLHLADSVYQLHLVSVLMGLCVGFCEAPLHSYIGEVAEPHLRGTISTLVCIAGHTGGVLLHVLGYLAQWRTTALFCGAVPAITFFAMTQIPESPTWLILNSRLKEAQKALGWVRGWVEAEVVHEEFQRLLEHAAVAPKSRRVSTFEGQKYEMVPLTEDGTPQKSTQETESFLRIKCRELSDQKMFRPLRMVFIVFIFSFATQLMGMRPFLVNIFNEFGLQIDSQLVVALTRFSLLVGAILNVTLLRRFGKRKLTLLCQAAATVSIILLGVYCSIFDETNRNASLSWIPISLMTSVYFFIGFSLTILPWQLCAEVFPIRGRGAAQGLSAGWSYYVRFGMSVTYLYLEKWIRLSGVFYLYGVISVIGFWYYWRYLPETEGKSLEQIESYFTDNHDKTEKFSRVKSK |
| *NON_BTST88* | MREIDESGGTQDGQINEQKSSKKHKTDLRSASAQILATLIQDWLLLGIGLTLGVPTLVVGALYRNPASTFTLDDDQASWIGSIPFICNIIGSLASGPFQEQFGRKGSMILVNIPFFCAWLLLFYARSVASLYAASAIMGLCAGFSEAPLHSYCGEIGEPHLRGMLSTMSTSAAIMGSLLIYVFAYFFEWRGAALISSAFPVITFISMTQIPESPTWLVMRGRLEDAQRSLCWLRGWVEPDQVRDEFEALVNYTKQKVASLETAHLNILERPAIEKCNVFTAHLKGLTAKNLLRPLRMECIVIANSYTTGFAGSKPYQIQIFRQLGYGDFAKRILILGHLIFFVGAMGNLILLPYFGKRKLALFAFGLSFSCLFGIGTFGIFRMELIQIPGLFWLPLILILVFKFTLGLSIMPLPWQLLCEVFPPIGRGTASGISSAFGNLISFGMTKSFLYLKAWLDLPGVIYLYVACAFLGWLYFYFYLPETEGKTLEQIESYFTENHDRKEKFSIGKSGRKN |
| *NON_BTST89* | MEKLNESYGMQQVTIDTENTEIKHKQNLRSTSAQLLATLAQNWLLLDIGLTFGVPPLVLGALHLNTGSGLSLNSSETSWLGSLPSICHLIGSLASGLFQEQFGRKGSMVLVNIPFFCSWLLLYHAESVLALYIAFITMGLCAGFCEAPLHSYCGEIAEPRLRGTLSTLCVAATILGAVFMYLLGYFFEWRVAALISSGVPVITFLLTTQIPESPTFLLMRGKTEEARKALCWLRGWADPDEVKEEFQALINYTKQRVASLESSGGNKNASLKFKKPNDLKAQLSELTSDRILIPFRLEFFVTLNAGITSFVGFQPYQIIVYKELGYPNFGKEILVRYTRIFLGESDTVMKKRHDTKVRSSLTNVTDEQLLGETKENQKKLLSAIRGRGTASGISTAWAYLLTFSLTKTFLVMVAWLNLGNVFYLYGTCGVLGWIYFYFSLPETEGKSLEQIESYFTKNHDRKEKFSVGKSGHLK |
| *NON_BTST90* | MCNKTEPSKSLDVPGNDAKPSFQYKNPGKSAFAQILAIIMQNWLLIEIGLEMAMPVIVLGALHNNPAAALNLNDDEASWFGSIPDFFHPIGSLTSGLLQEKFGRKGAVMMINIPIFIGWMTLYFAKSVYMLYAVSVIMGLCTGLAEAPLHAYIGEIGEPRMRGTLSTISTSCCIIGVSLMYLLGYLFEWKTVALISSSCSVITFLMMTQLPESPTWLIVRGRLDEARKSLCWLRGWVTADEAEPEFQALVNYTRNSAGLSRQDSTNSTVDDDGPLVRKDGFLTQQFKELMNKKTFRPLRMVFTLFIICFFGYVGGIRPYFINELKKLESPIDPKLFLTMGTGWLFLGAMINVVFLRRFGKRRIAIFSHALGGVAIAGVGIYATFLQGLTQYPLRVWVPIALWTVVNFLNGLSTVTLPWQIVCEVFPPLSRGTATGLSAAWAHLVLSVHVKLYLYVEAWIGFNGIMYLYGICTLLGSTYHYFCLPETEGKSLEQIESYFTKKHDKKEKFSMGKSVQKGDP |
| *NON_BTST91* | MFSKFMPFKSKSMSMETGNDRAIKPLICVALLIVFLAGCILGRSEDPRDEDDPYNRLKNHHDPKTFGDILYEYIRDIVKVPVIATVFFCWVCGSFADEHGRVGAMQLFFMLSGIGFGFLVYAQEYDFSILGTFILGAALGCSIPAPIYIAELCPVAYRSFFLGLVPVALSLGMFTVDVIELRGAEDTAWKSLCCFSGIGFLLSLFLHEAPEWLVMRNRPDAAIESLKWLKETSVDVDVDLRKLQETSMAANHRSDTTLEMLTDKRVWKPFAMLLGLALFQHLCGFYILIFYAPYLVNQYRTNIYWFSSYTGTDFLLLVATSAALVFHANLPRRTVAGLSGIGSSAALLGLFLHAHLFVAPQDLLDPTPDKDMLVPVFFFTLYIFSAVMGIYTLPWILMFEVFPLRHRGILCGLSFSTLYLGLFAFESRLNNYLLTGMDLQSLLCFFGTCALGFALFARSCLVETHKKTFEEIERGFTKERIFLPIDEKM |
