# Supplementary material for: Genome-Wide Characterization and Expression Profiling of Sugar Transporter Family in the Whitefly, Bemisia tabaci (Gennadius) (Hemiptera: Aleyrodidae)
Source: Front Physiol. 2017 May 23;8:322. doi: 10.3389/fphys.2017.00322 (PMC5440588; doi:10.3389/fphys.2017.00322)
Supplement: Supplementary file 10 [file Table10.DOCX]

**Table S10 Expression value of *BTSTs* across different developmental stages.** The expression values were presented as FPKM. E, Eggs; N1-2, 1^st^ and 2^nd^ nymphs; N3, 3^rd^ nymphs; N4, 4^th^ nymphs; M, Males; F, Females.

| **Gene** | **E** | **N12** | **N3** | **N4** | **F** | **M** |
| --- | --- | --- | --- | --- | --- | --- |
| *BtST1* | 39.64237 | 5.485277 | 5.52082 | 0 | 0 | 0 |
| *BtST2* | 0 | 0 | 0 | 0 | 0 | 0 |
| *BtST3* | 1.582753 | 16.63043 | 23.10483 | 15.57267 | 3.747067 | 6.77833 |
| *BTST4* | 0.59998 | 1.201499 | 2.412863 | 1.6459 | 0.878879 | 1.661744 |
| *BTST5* | 0.208197 | 3.91998 | 4.484293 | 4.380047 | 17.7772 | 34.01537 |
| *BTST6* | 0.132874 | 0.950288 | 2.05723 | 1.663817 | 2.73078 | 4.61792 |
| *BTST7* | 0.22027 | 0.194324 | 0.385751 | 0.380886 | 0.49944 | 0 |
| *BTST8* | 0.403738 | 4.396473 | 4.02002 | 2.77193 | 2.58799 | 4.08716 |
| *BTST9* | 1.049517 | 7.26004 | 7.44413 | 7.379717 | 8.86626 | 1.450042 |
| *BtST10* | 6.511093 | 5.958983 | 4.032283 | 4.047547 | 1.066231 | 2.152233 |
| *BtST11* | 2.108387 | 22.00173 | 28.26063 | 10.54562 | 17.10413 | 5.415323 |
| *BtST12* | 0.613815 | 3.040657 | 7.404157 | 10.54997 | 14.75833 | 6.570813 |
| *BtST13* | 10.76971 | 3.266967 | 11.20064 | 21.65403 | 28.36233 | 10.10921 |
| *BtST14* | 0.536118 | 1.353432 | 4.028227 | 3.76034 | 7.87693 | 4.014907 |
| *BtST15* | 9.581643 | 12.0407 | 12.39803 | 11.90027 | 10.34831 | 9.776293 |
| *BtST16* | 0 | 0.964037 | 1.341477 | 1.37535 | 0.303734 | 0.223857 |
| *BtST17* | 0.301663 | 17.9261 | 10.43473 | 17.38083 | 2.376592 | 1.987403 |
| *BtST18* | 3.35548 | 5.5925 | 2.46363 | 1.374727 | 3.05634 | 2.547247 |
| *BtST19* | 12.92479 | 22.60367 | 25.52353 | 16.13847 | 10.70015 | 8.390183 |
| *BtST20* | 1.299268 | 1.91969 | 3.122247 | 1.973707 | 8.279277 | 0.906971 |
| *BtST21* | 1.702937 | 12.18327 | 6.815313 | 4.533457 | 9.036067 | 5.834347 |
| *BtST22* | 0.156644 | 0.187051 | 1.202718 | 4.335567 | 2.456817 | 5.506097 |
| *BtST23* | 0.110768 | 2.41027 | 2.125333 | 0.278349 | 0.47871 | 0.336974 |
| *BtST24* | 0.615675 | 8.892707 | 8.24588 | 9.283717 | 7.125893 | 11.33577 |
| *BtST25* | 0 | 1.019718 | 0.887618 | 1.278531 | 0.87067 | 1.075658 |
| *BtST26* | 0.097369 | 0.825509 | 1.177846 | 0.089049 | 0.095032 | 1.090152 |
| *BtST27* | 0.210196 | 1.244538 | 1.781754 | 0.260261 | 0.575564 | 0.246368 |
| *BtST28* | 0.121797 | 0.616915 | 0.745619 | 0.332156 | 1.004907 | 0.058684 |
| *BtST29* | 0.370561 | 12.68577 | 9.314317 | 7.154927 | 13.58113 | 16.57557 |
| *BtST30* | 0 | 0.396819 | 0 | 0 | 0.122162 | 0.135028 |
| *BtST31* | 0.107749 | 0.673827 | 0.799171 | 0.307218 | 0.136131 | 0.171934 |
| *BtST32* | 0.2499 | 0.648805 | 0.501936 | 0.146317 | 0 | 0 |
| *BtST33* | 0.271365 | 0.447692 | 0.381925 | 0.408783 | 0 | 1.969933 |
| *BtST34* | 0.281946 | 0.516285 | 0.554377 | 0.35785 | 0.40857 | 0.420465 |
| *BtST35* | 0.406441 | 0.368053 | 0.435529 | 0.253067 | 0.362915 | 0.11558 |
| *BtST36* | 0.107682 | 3.696317 | 4.192197 | 2.226813 | 1.63055 | 2.545397 |
| *BtST37* | 0.132687 | 4.3014 | 5.731967 | 0.278325 | 0.268194 | 0.320524 |
| *BtST38* | 0.101796 | 7.218723 | 17.31 | 6.60809 | 8.56415 | 5.320273 |
| *BtST39* | 18.44323 | 34.34803 | 42.03353 | 38.42913 | 35.8281 | 44.51687 |
| *BtST40* | 0.28054 | 4.19068 | 3.6244 | 8.083977 | 5.807133 | 6.9545 |
| *BtST41* | 0.10806 | 18.00243 | 28.38217 | 17.6058 | 20.03377 | 21.43453 |
| *BtST42* | 1.340213 | 0.56724 | 0.314458 | 0.406389 | 1.208865 | 1.28232 |
| *BTST43* | 1.54751 | 17.38927 | 7.16023 | 2.930033 | 5.980137 | 1.18629 |
| *BTST44* | 0.190104 | 2.47726 | 0.804905 | 1.610563 | 3.37902 | 3.210377 |
| *BTST45* | 2.16522 | 4.99988 | 13.06973 | 8.87494 | 8.508367 | 7.88802 |
| *BTST46* | 7.14149 | 15.8674 | 13.87847 | 11.47353 | 6.070957 | 7.778587 |
| *BTST47* | 0.894211 | 6.06033 | 2.44674 | 0.88011 | 3.05322 | 1.71407 |
| *BTST48* | 1.261886 | 1.811733 | 1.46661 | 0.662733 | 0.247438 | 0.302116 |
| *BTST49* | 5.46958 | 10.44775 | 4.105847 | 2.454213 | 4.733017 | 4.164563 |
| *BTST50* | 2.61777 | 1.84621 | 2.475413 | 1.552351 | 0.656739 | 0.423523 |
| *BTST51* | 0.191463 | 2.80711 | 1.51171 | 0.68897 | 3.251367 | 3.650743 |
| *BTST52* | 0.912252 | 0.320753 | 0.284512 | 0.502762 | 0.311819 | 0.119409 |
| *BtST53* | 0 | 3.744047 | 4.7148 | 2.654123 | 1.396019 | 2.92538 |
| *BtST54* | 0.408694 | 1.55421 | 2.052393 | 2.097553 | 4.826313 | 0.859932 |
| *BtST55* | 0 | 1.853333 | 2.070597 | 0.612822 | 2.00022 | 2.254707 |
| *BtST56* | 0 | 2.372503 | 0.913919 | 1.40841 | 3.23183 | 3.45974 |
| *BtST57* | 0.04902 | 1.176567 | 0.8063 | 0.349787 | 1.961323 | 1.5443 |
| *BtST58* | 7.08978 | 23.7947 | 26.31753 | 13.88633 | 27.08217 | 21.06483 |
| *BtST59* | 8.90463 | 39.8156 | 31.12467 | 35.85233 | 20.0657 | 17.4667 |
| *BTST60* | 2.468413 | 5.580687 | 6.09529 | 8.207997 | 8.67296 | 4.28439 |
| *BtST61* | 2.438026 | 12.1045 | 18.66297 | 12.50813 | 37.81137 | 5.605063 |
| *BtST62* | 0.355085 | 2.514037 | 0.807534 | 0.550627 | 2.161173 | 0.957912 |
| *BtST63* | 0.190656 | 0.746726 | 1.74553 | 0.208076 | 0 | 0.198475 |
| *BtST64* | 1.51529 | 4.077793 | 2.64557 | 2.001847 | 2.57729 | 0.863693 |
| *BtST65* | 8.204707 | 3.33438 | 4.47149 | 9.501117 | 2.744663 | 2.189897 |
| *BtST66* | 7.21E-05 | 1.71E-05 | 4.99E-06 | 2.25E-05 | 7.03E-06 | 8.62E-06 |
| *BtST67* | 2.37E-05 | 3.11E-05 | 3.27E-05 | 0.200405 | 1.2E-05 | 1.793537 |
| *BtST68* | 0.17821 | 2.168753 | 0.799957 | 0.468879 | 0.824976 | 0.371535 |
| *BtST69* | 3.24147 | 4.059353 | 1.21083 | 0.789061 | 2.67117 | 0.207002 |
| *BtST70* | 110.2647 | 49.46767 | 68.75953 | 107.1723 | 65.08487 | 21.94363 |
| *BtST71* | 0.269937 | 27.32373 | 23.33703 | 16.89773 | 10.91191 | 16.783 |
| *BtST72* | 0.491696 | 3.404127 | 1.621317 | 0.425116 | 0 | 0 |
| *BtST73* | 25.5133 | 18.89013 | 32.47793 | 32.73667 | 26.91603 | 10.63671 |
| *BtST74* | 0.306368 | 0.239316 | 0.518406 | 0.231751 | 0.330366 | 0.17572 |
| *BtST75* | 0.223431 | 11.762 | 10.72298 | 2.111687 | 3.763873 | 2.595427 |
| *BtST76* | 0 | 0.198122 | 0.290272 | 0.310335 | 1.8199 | 0 |
| *BtST77* | 0.150089 | 1.582823 | 2.6253 | 0.941449 | 1.506343 | 0.891749 |
| *BtST78* | 12.47033 | 23.00987 | 17.27387 | 39.414 | 28.46297 | 18.67207 |
| *BtST79* | 0.097331 | 0.773123 | 1.295387 | 0.280894 | 0.65743 | 0.627528 |
| *BtST80* | 0.273392 | 3.400293 | 14.37793 | 6.154117 | 2.039877 | 2.082147 |
| *BtST81* | 1.358337 | 30.5853 | 49.61817 | 33.70247 | 15.22897 | 30.6989 |
| *BtST82* | 1.400769 | 6.333863 | 7.315543 | 4.376433 | 10.55678 | 6.375343 |
| *BtST83* | 5.39503 | 7.674057 | 7.783267 | 4.244143 | 20.43037 | 7.54123 |
| *BtST84* | 1.172092 | 96.32657 | 52.76983 | 21.72147 | 50.22363 | 15.761 |
| *BTST85* | 0.193232 | 1.684427 | 0.818372 | 0.1338 | 0.174671 | 0.257847 |
| *BtST86* | 0.971491 | 11.65384 | 12.04087 | 10.0362 | 13.45824 | 13.38814 |
| *BtST87* | 0.420882 | 0.773867 | 0.603949 | 0.14906 | 0.141046 | 0.166706 |
| *BtST88* | 0.292592 | 1.594897 | 0.693632 | 0.64132 | 0.17799 | 0.258005 |
| *BtST89* | 0.421932 | 2.57668 | 1.44154 | 0 | 0 | 0.130951 |
| *BtST90* | 0.636014 | 20.3598 | 6.780863 | 6.92197 | 28.70087 | 27.70713 |
| *BtST91* | 2.84542 | 16.35173 | 7.370343 | 5.318367 | 6.16094 | 2.9346 |
| *BtST92* | 2.218693 | 1.450047 | 0.620225 | 0.802207 | 0.808099 | 0.525508 |
| *BtST93* | 0.011582 | 0.987975 | 0.777361 | 0.291922 | 0.921854 | 1.08646 |
| *BtST94* | 0.125915 | 1.39426 | 2.708258 | 0.268415 | 2.288843 | 7.886487 |
| *BtST95* | 0.121396 | 0.176171 | 0.488379 | 0.865798 | 0.833506 | 3.682023 |
| *BtST96* | 0 | 0 | 0.205814 | 0.293802 | 0.350009 | 0.253913 |
| *BtST97* | 0.165808 | 11.57031 | 10.58454 | 4.168577 | 4.69321 | 3.223313 |
| *BtST98* | 1.822877 | 0.590805 | 1.22774 | 1.60881 | 0.504901 | 0.22891 |
| *BtST99* | 4.7101 | 8.618103 | 9.869723 | 17.41637 | 15.64967 | 6.955283 |
| *BtST100* | 0.086272 | 0 | 0.424008 | 0.207752 | 0 | 0.490033 |
| *BtST101* | 0 | 9.648573 | 12.1719 | 3.26565 | 1.719895 | 1.456883 |
| *BtST102* | 0 | 1.906043 | 0.604295 | 0.281779 | 0.135204 | 0 |
| *BtST103* | 0 | 0 | 0 | 0 | 0 | 0 |
| *BtST104* | 0.095042 | 2.60348 | 0.870297 | 0.084002 | 0.147546 | 0 |
| *BtST105* | 0 | 0.755865 | 0.322225 | 0.8527 | 1.216517 | 0.17486 |
| *BtST106* | 0.236512 | 1.854537 | 5.142837 | 2.928033 | 3.63578 | 0.966243 |
| *BtST107* | 3.74616 | 5.092127 | 6.275547 | 8.502627 | 6.360677 | 3.26404 |
| *BTST108* | 0.097314 | 1.72121 | 0.693782 | 1.001436 | 0.234748 | 0.52031 |
| *BTST109* | 4.112197 | 10.7239 | 9.727197 | 5.46706 | 8.91425 | 4.28581 |
| *BTST110* | 0.192139 | 0.685331 | 1.072551 | 0.326747 | 0.15209 | 0.189373 |
| *BTST111* | 13.58317 | 23.1976 | 34.69273 | 21.54097 | 15.90833 | 19.90117 |
| *BtST112* | 0.443747 | 0.317771 | 0.698499 | 0.279454 | 0 | 0 |
| *BtST113* | 0.178306 | 1.772113 | 0.462538 | 0.192249 | 0.400593 | 0.11121 |
| *BTST114* | 0 | 0.557492 | 0.335896 | 0.509772 | 0.72649 | 0.043715 |
| *BTST115* | 0.137911 | 0.184037 | 0.550851 | 0.16877 | 0.16559 | 0.367653 |
| *BTST116* | 0 | 0.363612 | 1.08432 | 0.319843 | 0.700135 | 0.480724 |
| *BTST117* | 0.288734 | 0.917749 | 1.044561 | 0.280735 | 0.201031 | 0 |
| *BTST118* | 0.215753 | 1.456367 | 1.507409 | 0.290729 | 0.630965 | 0.384775 |
| *BTST119* | 0 | 1.214829 | 0.650744 | 0.123501 | 0.125215 | 0.336246 |
| *BtST120* | 4.7894 | 18.24927 | 23.51867 | 40.4623 | 15.43087 | 11.23423 |
| *BtST121* | 0.201088 | 0.353558 | 0.690085 | 0.23487 | 0.17516 | 0.199229 |
| *BtST122* | 2.035893 | 1.209587 | 1.41726 | 1.93312 | 0.66923 | 0.289863 |
| *BtST123* | 0.230198 | 0.138355 | 0.952409 | 0.667767 | 0 | 1.184401 |
| *BtST124* | 0.04902 | 0.885877 | 0.334515 | 0.059412 | 2.042953 | 1.771717 |
| *BtST125* | 0 | 0.264143 | 0.48302 | 0.163346 | 0.216945 | 0.058684 |
| *BtST126* | 0 | 0.485776 | 0.457362 | 0.066951 | 0.189752 | 0.100911 |
| *BtST127* | 7.023657 | 10.4324 | 11.7387 | 17.74607 | 18.7522 | 11.13902 |
| *BtST128* | 8.75977 | 11.76333 | 11.31176 | 20.4778 | 15.76863 | 10.58796 |
| *BtST129* | 0.100006 | 0.498573 | 1.029762 | 0.241562 | 0.59769 | 0.464705 |
| *BtST130* | 0.394411 | 1.101584 | 0.32875 | 0.343664 | 0.284089 | 0 |
| *BtST131* | 3.208347 | 18.30497 | 8.021857 | 6.322293 | 5.942563 | 4.472537 |
| *BtST132* | 6.7149 | 15.82983 | 13.05793 | 23.08473 | 9.15458 | 9.641613 |
| *BtST133* | 0 | 8.849323 | 0 | 0 | 0 | 0 |
| *BtST134* | 4.7398 | 7.379347 | 5.181353 | 7.41286 | 4.081013 | 4.843557 |
| *BtST135* | 5.495343 | 4.72103 | 5.718903 | 5.25283 | 7.470487 | 2.09298 |
| *BtST136* | 4.92288 | 4.76197 | 0 | 0 | 8.455363 | 7.545193 |
| *BtST137* | 0 | 6.722933 | 8.523707 | 9.55076 | 9.28156 | 21.93693 |
