# Supplementary material for: Genome-Wide Characterization and Expression Profiling of Sugar Transporter Family in the Whitefly, Bemisia tabaci (Gennadius) (Hemiptera: Aleyrodidae)
Source: Front Physiol. 2017 May 23;8:322. doi: 10.3389/fphys.2017.00322 (PMC5440588; doi:10.3389/fphys.2017.00322)
Supplement: Supplementary file 11 [file Table11.DOCX]

**Table S11 Expression value of *BTSTs* in male and female adults that fed on different host plants.** The expression value was presented as FPKM. CaF, Females reared on cabbage; CaM, Males reared on cabbage; CuF, Females reared on cucumber; CuM, Males reared on cucumber; CoF, Females reared on cotton; CoM, Males reared on cotton. ToF, Females reared on tomato; ToM, Males reared on tomato.

| **Gene** | **CuM** | **CuF** | **ToM** | **ToF** | **CoM** | **CoF** | **CaM** | **CaF** |
| --- | --- | --- | --- | --- | --- | --- | --- | --- |
| BTST1 | 0 | 6.3364 | 9.5328 | 29.276 | 9.2089 | 5.3364 | 18.2089 | 7.15336 |
| BTST2 | 0 | 0 | 0 | 0 | 0 | 0 | 0 | 0 |
| BTST3 | 4.25901 | 9.75817 | 5.40625 | 15.4073 | 6.15211 | 24.7957 | 5.80713 | 14.3705 |
| BTST4 | 3.16677 | 2.04222 | 7.06869 | #VALUE! | 3.60505 | 8.41971 | 4.53063 | 3.10791 |
| BTST5 | 7.89674 | 22.247 | 27.2729 | 39.965 | 11.4143 | 61.9955 | 10.9692 | 27.646 |
| BTST6 | 1.05422 | 2.88937 | 2.96499 | 4.40296 | 1.64211 | 10.9161 | 1.72679 | 4.86827 |
| BTST7 | 0 | 0 | 0.932167 | 0 | 0.328533 | 0 | 0.172737 | 0 |
| BTST8 | 3.67649 | 8.91976 | 21.1096 | 51.013 | 4.29839 | 36.4421 | 5.50265 | 14.6944 |
| BTST9 | 10.0146 | 6.01279 | 40.14 | 24.6361 | 14.695 | 9.48238 | 14.129 | 6.48616 |
| BTST10 | 0.345209 | 1.79524 | 0.924668 | 2.76015 | 0.597466 | 4.16084 | 0.628274 | 2.98259 |
| BTST11 | 14.749 | 4.0559 | 30.1546 | 9.41003 | 20.1792 | 11.0668 | 20.9014 | 6.62309 |
| BTST12 | 20.9979 | 3.37397 | 22.0168 | 8.79607 | 15.5521 | 4.46982 | 20.2998 | 4.48741 |
| BTST13 | 18.2616 | 5.58771 | 42.0371 | 16.661 | 21.6131 | 14.3433 | 31.6849 | 6.64434 |
| BTST14 | 10.9981 | 1.29003 | 11.7743 | 3.09619 | 5.00457 | 2.94773 | 9.47796 | 1.57154 |
| BTST15 | 17.7325 | 15.5361 | 40.3163 | 31.6424 | 19.9478 | 25.6547 | 15.0735 | 16.3236 |
| BTST16 | 0.322034 | 1.67472 | 0.479219 | 1.27228 | 0.658694 | 1.23503 | 0.479533 | 3.07019 |
| BTST17 | 9.6435 | 29.1268 | 7.67439 | 18.6355 | 9.82125 | 26.6178 | 4.89069 | 22.7348 |
| BTST18 | 4.02785 | 0.626352 | 13.8751 | 7.41643 | 6.47224 | 9.20253 | 4.36717 | 3.26813 |
| BTST19 | 15.1274 | 22.9999 | 46.6504 | 57.1247 | 23.4227 | 70.6788 | 18.7104 | 23.9607 |
| BTST20 | 8.61943 | 3.70863 | 31.6737 | 15.1569 | 14.8903 | 12.9141 | 13.679 | 6.56845 |
| BTST21 | 8.54389 | 7.75744 | 28.1753 | 30.6223 | 13.3056 | 29.5206 | 10.7104 | 10.5164 |
| BTST22 | 0.502484 | 3.86087 | 3.58918 | 6.61733 | 1.37039 | 11.7459 | 1.88445 | 4.65749 |
| BTST23 | 1.41518 | 2.54256 | 2.38977 | 1.38908 | 2.55579 | 3.0339 | 1.52703 | 2.34643 |
| BTST24 | 5.71886 | 15.1234 | 14.0486 | 36.1024 | 9.28369 | 45.2571 | 8.98141 | 26.3235 |
| BTST25 | 1.19297 | 3.37729 | 2.34321 | 7.06221 | 1.48399 | 8.29137 | 3.83363 | 3.24308 |
| BTST26 | 0.271435 | 2.81052 | 1.278 | 3.54013 | 6.77267 | 19.29 | 2.18318 | 3.46101 |
| BTST27 | 0.759236 | 0.36939 | 0.834327 | 0.230736 | 0.827017 | 1.24788 | 0.579774 | 0.243599 |
| BTST28 | 1.18882 | 0.050128 | 2.22905 | 1.54322 | 1.29064 | 0 | 1.34244 | 0 |
| BTST29 | 7.54368 | 61.7816 | 18.3428 | 49.6105 | 8.30376 | 78.8037 | 6.18636 | 29.0663 |
| BTST30 | 0 | 0.414072 | 0 | 0.55424 | 0 | 0 | 0.060928 | 0.073142 |
| BTST31 | 0.204995 | 0.806753 | 0.06101 | 0 | 0.064508 | 0.449241 | 0.067834 | 0.325728 |
| BTST32 | 0.063059 | 0.478611 | 0.056303 | 0.996529 | 0.119061 | 0.621867 | 0 | 0.300595 |
| BTST33 | 0.059877 | 2.92871 | 0.481152 | 4.02149 | 0.339155 | 7.67625 | 0.237762 | 3.49645 |
| BTST34 | 0.556952 | 0.781003 | 0.756246 | 1.38591 | 0.530263 | 2.60877 | 0.36742 | 1.24355 |
| BTST35 | 1.44069 | 0.420561 | 2.07792 | 1.03203 | 0.784652 | 0.364295 | 0.990134 | 0.594309 |
| BTST36 | 2.27346 | 6.40764 | 6.26325 | 14.0638 | 2.8993 | 19.7158 | 3.17748 | 11.9246 |
| BTST37 | 0.061376 | 0.207036 | 0.712395 | 0.96992 | 0.231763 | 0.201754 | 0.060928 | 0.365711 |
| BTST38 | 3.86038 | 15.1331 | 11.6329 | 28.2901 | 9.45254 | 45.1086 | 13.7722 | 15.5626 |
| BTST39 | 45.8613 | 143.281 | 143.343 | 325.369 | 62.4455 | 329.026 | 60.7608 | 154.887 |
| BTST40 | 5.87787 | 27.6443 | 27.9311 | 46.7951 | 7.36301 | 44.5888 | 9.03313 | 22.092 |
| BTST41 | 39.7901 | 139.332 | 37.0246 | 127.946 | 40.2112 | 172.831 | 14.3588 | 122.037 |
| BTST42 | 0.598254 | 1.16956 | 1.14115 | 0.184174 | 0.770147 | 3.93317 | 0.755869 | 0.777764 |
| BTST43 | 4.81506 | 0.510771 | 22.5233 | 6.83673 | 9.03403 | 3.98192 | 5.68189 | 0.866144 |
| BTST44 | 5.72754 | 12.3973 | 18.41 | 40.5768 | 5.79324 | 39.6277 | 8.09554 | 15.7314 |
| BTST45 | 19.5411 | 22.9405 | 51.7895 | 68.532 | 16.8768 | 93.2371 | 14.1361 | 37.5827 |
| BTST46 | 3.14849 | 4.60373 | 8.41079 | 11.0059 | 5.03372 | 16.0254 | 4.48669 | 7.65551 |
| BTST47 | 9.43447 | 6.62166 | 27.635 | 32.3952 | 13.5321 | 42.9181 | 10.7555 | 17.4814 |
| BTST48 | 0.118786 | 0.100174 | 0.636356 | 0.670424 | 0.953179 | 1.17143 | 0.471685 | 1.34482 |
| BTST49 | 15.4299 | 15.5026 | 22.3753 | 26.3659 | 20.4683 | 42.7632 | 17.2454 | 23.3021 |
| BTST50 | 2.76817 | 3.20986 | 1.02983 | 2.34351 | 1.79661 | 2.27489 | 1.145 | 3.16142 |
| BTST51 | 9.9354 | 6.30324 | 14.6221 | 13.6157 | 12.7927 | 18.1776 | 3.43849 | 10.4281 |
| BTST52 | 1.45121 | 0.356952 | 3.72522 | 2.25241 | 1.37 | 0.795074 | 1.32059 | 0.28824 |
| BTST53 | 2.30401 | 6.45766 | 5.52105 | 14.8204 | 2.58024 | 25.6728 | 3.53175 | 13.022 |
| BTST54 | 6.55871 | 2.38465 | 29.5255 | 8.77769 | 10.5666 | 5.93864 | 7.44665 | 2.62098 |
| BTST55 | 4.8923 | 4.46754 | 4.96262 | 5.81648 | 6.77748 | 12.9418 | 4.45435 | 9.90107 |
| BTST56 | 2.93646 | 5.74517 | 3.56571 | 5.90007 | 2.82757 | 8.10832 | 2.56527 | 6.43896 |
| BTST57 | 8.06713 | 6.83762 | 6.27812 | 7.87554 | 11.475 | 17.6042 | 4.89701 | 12.8292 |
| BTST58 | 23.6982 | 43.3375 | 26.53 | 56.0762 | 28.785 | 96.6518 | 28.5448 | 46.781 |
| BTST59 | 14.6205 | 91.5925 | 20.5614 | 63.5356 | 15.8752 | 88.3755 | 15.471 | 50.8028 |
| BTST60 | 9.44524 | 6.11322 | 16.0789 | 7.75294 | 18.6981 | 9.76556 | 12.8234 | 14.9502 |
| BTST61 | 37.8298 | 6.90997 | 68.836 | 25.4049 | 32.9 | 21.163 | 50.7062 | 11.6035 |
| BTST62 | 5.48716 | 3.80477 | 14.0173 | 11.321 | 11.8279 | 15.8329 | 5.4472 | 8.17394 |
| BTST63 | 0.062259 | 0.262519 | 0.333529 | 0 | 0.058774 | 0.409314 | 0.247221 | 0.445168 |
| BTST64 | 2.0967 | 0.353636 | 4.43053 | 3.55009 | 2.1773 | 1.37846 | 4.92604 | 0.666312 |
| BTST65 | 3.30383 | 2.1306 | 7.69275 | 2.85184 | 2.32392 | 3.40718 | 3.34408 | 1.46681 |
| BTST66 | 0 | 0 | 0.476437 | 0 | 0.846318 | 0 | 0 | 2.72768 |
| BTST67 | 0.371936 | 1.25487 | 0.355068 | 0 | 1.37601 | 0 | 0 | 0.364126 |
| BTST68 | 3.43947 | 1.14265 | 1.53548 | 0.2353 | 4.7475 | 1.88438 | 1.96588 | 1.86313 |
| BTST69 | 3.40673 | 1.01128 | 3.40673 | 3.38402 | 4.86271 | 1.97096 | 3.97713 | 1.2342 |
| BTST70 | 107.276 | 23.4199 | 102.217 | 47.5507 | 100.028 | 47.7633 | 94.8043 | 40.8804 |
| BTST71 | 32.922 | 55.6917 | 65.3473 | 160.681 | 31.4447 | 178.246 | 24.2053 | 90.2346 |
| BTST72 | 0.581893 | 0.163573 | 0 | 0 | 0.549329 | 0.637601 | 0.385103 | 0.346726 |
| BTST73 | 37.1765 | 10.4009 | 89.6196 | 44.4565 | 43.5436 | 35.6074 | 49.9382 | 21.1522 |
| BTST74 | 0.872794 | 1.0599 | 1.70403 | 1.53691 | 1.18649 | 2.18048 | 1.1437 | 1.04012 |
| BTST75 | 8.77596 | 8.06715 | 22.5513 | 25.5119 | 18.7909 | 24.5579 | 13.213 | 16.2915 |
| BTST76 | 1.23331 | 0.148582 | 3.42588 | 0.596636 | 1.44151 | 0.193055 | 1.63244 | 0.139977 |
| BTST77 | 4.69964 | 3.91312 | 6.32074 | 6.71508 | 7.86241 | 11.1465 | 1.88979 | 7.72748 |
| BTST78 | 32.9548 | 27.4896 | 81.2635 | 60.3728 | 43.6638 | 99.0667 | 62.0862 | 37.402 |
| BTST79 | 2.48658 | 2.8402 | 3.96257 | 5.18729 | 3.98171 | 9.62244 | 1.81229 | 6.11415 |
| BTST80 | 5.00565 | 8.21185 | 5.30735 | 6.93049 | 5.61157 | 18.7686 | 3.10575 | 12.2103 |
| BTST81 | 54.3316 | 196.436 | 125.646 | 358.622 | 67.5879 | 415.212 | 60.7066 | 190.616 |
| BTST82 | 17.0247 | 9.13944 | 29.8208 | 17.2155 | 22.9547 | 22.4114 | 15.2185 | 12.8918 |
| BTST83 | 15.0575 | 8.19731 | 35.594 | 18.2421 | 26.7409 | 20.9751 | 18.8551 | 12.0814 |
| BTST84 | 56.8827 | 40.1179 | 172.368 | 125.105 | 82.4138 | 80.3221 | 67.3924 | 49.9669 |
| BTST85 | 0.245297 | 0.258579 | 0.054754 | 0.761445 | 0.057893 | 0.604757 | 0.182633 | 0.803894 |
| BTST86 | 21.3999 | 34.8085 | 51.4151 | 89.6733 | 27.2243 | 114.838 | 38.1002 | 54.2386 |
| BTST87 | 0.320165 | 0.562502 | 0.547903 | 0.963731 | 0.151124 | 0.263112 | 0.158917 | 0.826685 |
| BTST88 | 1.95215 | 0.800275 | 1.54933 | 1.46904 | 1.12622 | 1.24777 | 0.888218 | 1.45401 |
| BTST89 | 0 | 0.150627 | 0 | 0 | 0.112411 | 0 | 0.118207 | 0.141903 |
| BTST90 | 103.688 | 148.372 | 126.061 | 250.734 | 136.709 | 652.72 | 98.962 | 421.498 |
| BTST91 | 5.89326 | 7.25531 | 20.8554 | 23.5661 | 10.4772 | 17.6755 | 7.92857 | 10.7824 |
| BTST92 | 2.34563 | 1.82253 | 2.51789 | 1.36849 | 2.33877 | 1.90599 | 2.45936 | 2.26139 |
| BTST93 | 2.77276 | 2.93939 | 3.89655 | 5.17435 | 4.46335 | 9.46957 | 2.70597 | 6.57328 |
| BTST94 | 1.15622 | 3.29504 | 3.48861 | 14.9415 | 1.84429 | 8.12574 | 3.24551 | 4.65633 |
| BTST95 | 1.23048 | 3.46976 | 2.16296 | 12.1099 | 1.66983 | 14.9154 | 1.5269 | 6.32378 |
| BTST96 | 6.5718 | 2.40158 | 1.72119 | 1.58254 | 6.07995 | 2.01627 | 1.78321 | 11.6954 |
| BTST97 | 12.8095 | 14.4483 | 9.72153 | 15.0738 | 10.8834 | 13.0534 | 8.7742 | 13.1282 |
| BTST98 | 1.29197 | 1.3264 | 6.41975 | 5.54813 | 2.1742 | 3.04675 | 2.39784 | 1.60661 |
| BTST99 | 12.4215 | 6.95318 | 18.6525 | 11.1533 | 14.27 | 11.9276 | 9.97366 | 9.44975 |
| BTST100 | 0.030409 | 1.17963 | 0 | 4.42792 | 0.057414 | 4.09834 | 0 | 1.84816 |
| BTST101 | 1.99289 | 7.36637 | 5.6568 | 5.43921 | 1.79712 | 11.5376 | 1.44687 | 6.62862 |
| BTST102 | 0.798547 | 0.077703 | 0.767836 | 0 | 0.724865 | 0 | 0.701263 | 0.256212 |
| BTST103 | 0 | 0 | 0 | 0 | 0 | 0 | 0 | 0 |
| BTST104 | 0.140862 | 0.213824 | 0 | 0 | 0.425533 | 0.277826 | 0.251705 | 0.369309 |
| BTST105 | 0.565763 | 0.220208 | 1.36778 | 0.707403 | 1.19028 | 0.228896 | 1.36522 | 0.228201 |
| BTST106 | 3.29082 | 1.68332 | 5.32255 | 2.923 | 4.27803 | 2.83741 | 5.19484 | 1.92872 |
| BTST107 | 18.236 | 7.59623 | 16.8958 | 11.3312 | 16.7521 | 11.7793 | 18.6636 | 9.27201 |
| BTST108 | 2.29953 | 0.935396 | 1.88408 | 2.38193 | 1.37913 | 1.42288 | 0.832546 | 2.03112 |
| BTST109 | 19.2428 | 12.6453 | 25.9636 | 18.8954 | 15.3492 | 23.1908 | 12.6013 | 15.5191 |
| BTST110 | 0.620565 | 1.00641 | 2.21631 | 3.55632 | 1.17167 | 4.70753 | 0.284329 | 2.04795 |
| BTST111 | 37.7847 | 28.7441 | 47.4734 | 45.5641 | 27.3279 | 56.7298 | 27.7044 | 34.4799 |
| BTST112 | 0 | 0.154705 | 0 | 0 | 0.288637 | 0 | 0.182112 | 0.072873 |
| BTST113 | 0.309896 | 0.071275 | 0 | 0 | 0.691492 | 0.277826 | 0.587312 | 0.302162 |
| BTST114 | 0.356866 | 0.088083 | 1.50767 | 0.1179 | 1.09168 | 0.400568 | 1.31338 | 0.165964 |
| BTST115 | 0.557469 | 0.188049 | 0.497742 | 1.19561 | 0.999918 | 1.09951 | 1.32818 | 0.730781 |
| BTST116 | 0.585695 | 0.74089 | 0.998346 | 0.540922 | 0.703714 | 0.70011 | 1.48 | 0.824889 |
| BTST117 | 0.802742 | 0.225655 | 0.621171 | 0 | 0.656777 | 0.703675 | 0.318759 | 0.446433 |
| BTST118 | 0.315026 | 0.0797 | 0.675058 | 1.49351 | 0.713752 | 0 | 1.06329 | 0.901014 |
| BTST119 | 0.878633 | 0.231552 | 0.392248 | 0.61987 | 0.829463 | 0.180516 | 0.70869 | 0.588984 |
| BTST120 | 33.1351 | 29.166 | 114.06 | 110.942 | 44.0726 | 62.5063 | 26.6429 | 32.9725 |
| BTST121 | 0.758911 | 0.877041 | 2.10809 | 2.91896 | 1.27367 | 3.88064 | 0.725481 | 2.67973 |
| BTST122 | 1.03667 | 1.23851 | 7.27623 | 6.97233 | 2.28353 | 4.68565 | 2.144 | 0.926562 |
| BTST123 | 0.030409 | 1.53865 | 0 | 4.22197 | 0 | 6.49737 | 0 | 2.3555 |
| BTST124 | 7.68557 | 5.84933 | 7.03247 | 7.5679 | 7.53849 | 16.1708 | 4.19358 | 10.6855 |
| BTST125 | 0.356647 | 0.050128 | 1.22067 | 1.00645 | 0.785606 | 0 | 0.693347 | 0 |
| BTST126 | 0.408819 | 0.12313 | 0.5736 | 0.230736 | 0.275672 | 0.287973 | 0.521797 | 0.173999 |
| BTST127 | 19.1747 | 11.1287 | 19.9189 | 13.07 | 19.8515 | 13.0761 | 17.7045 | 12.8741 |
| BTST128 | 18.8877 | 16.8513 | 102.675 | 76.6958 | 23.4343 | 47.3269 | 34.8953 | 18.7127 |
| BTST129 | 1.97234 | 2.64494 | 4.53247 | 6.4966 | 2.86926 | 7.54638 | 1.34812 | 5.20188 |
| BTST130 | 0.274651 | 0 | 0.735673 | 0 | 0.691415 | 0 | 0.27265 | 0 |
| BTST131 | 6.63032 | 7.45529 | 21.9786 | 32.1736 | 10.5616 | 28.2086 | 10.0319 | 15.7848 |
| BTST132 | 20.9086 | 6.55133 | 10.7368 | 4.7368 | 0.999918 | 1.09951 | 10.5189 | 10.7467 |
| BTST133 | 5.05948 | 6.14797 | 15.3545 | 20.3545 | 0.703714 | 0.70011 | 4.58288 | 4.56894 |
| BTST134 | 4.97414 | 5.79751 | 8.93973 | 3.614 | 0.656777 | 0.703675 | 18.0121 | 8.42933 |
| BTST135 | 8.37836 | 3.21772 | 18.3344 | 33.2779 | 0.713752 | 0.703675 | 8.04134 | 5.39849 |
| BTST136 | 8.54389 | 3.75744 | 16.9906 | 8.27228 | 0.829463 | 0.180516 | 7.50532 | 5.34449 |
| BTST137 | 34.5939 | 17.2531 | 46.7961 | 20.594 | 23.7961 | 15.5939 | 22.0684 | 34.5939 |
