# Supplementary material for: Genome-Wide Characterization and Expression Profiling of Sugar Transporter Family in the Whitefly, Bemisia tabaci (Gennadius) (Hemiptera: Aleyrodidae)
Source: Front Physiol. 2017 May 23;8:322. doi: 10.3389/fphys.2017.00322 (PMC5440588; doi:10.3389/fphys.2017.00322)

**Figure S1. Model outline for branch-based molecular evolution of *B. tabaci* Sternorrhyncha expanded *STs*.** Bayesian phylogeny for *B. tabaci* sternorrhynchan expanded clades is shown, including insect orthologs and outgroups genes (see Figure 5). Each branch was assigned a ω category as indicated. Expressional levels of each *ST* were quantified by transcriptomes as described in Figure 2. E, Eggs; N1-2, 1^st^ and 2^nd^ nymphs; N3, 3^rd^ nymphs; N4, 4^th^ nymphs; M, Males; F, Females. A: Sternorrhynchan expanded clade 1; B: Sternorrhynchan expanded clade 2.


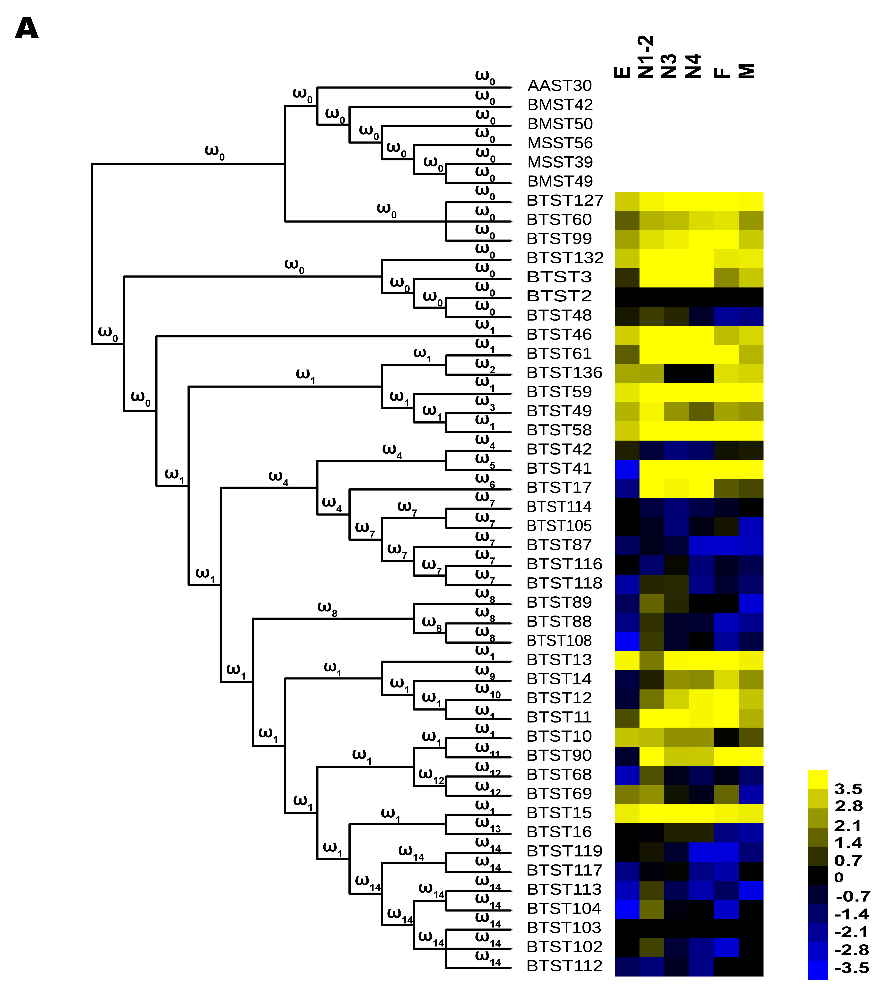


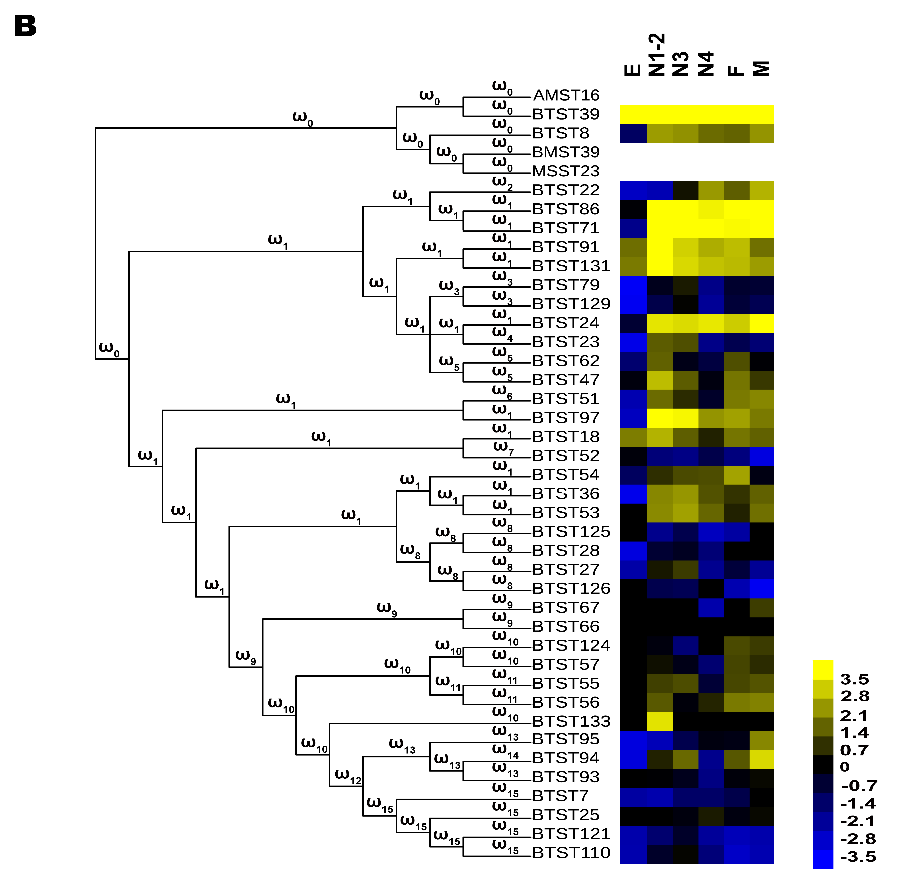

Supplement: Supplementary file 12 [file DataSheet1.DOCX]
