# Supplementary material for: Genome-Wide Characterization and Expression Profiling of Sugar Transporter Family in the Whitefly, Bemisia tabaci (Gennadius) (Hemiptera: Aleyrodidae)
Source: Front Physiol. 2017 May 23;8:322. doi: 10.3389/fphys.2017.00322 (PMC5440588; doi:10.3389/fphys.2017.00322)
Supplement: Supplementary file 13 [file DataSheet2.DOCX]

**Figure S2. Cloning of full-length coding sequences of nine *BTSTs*.** A PCR amplication of eight *BTSTs* full-length coding sequences. A, *BTST40*; B, *BTST44*; C, *BTST45*; D. *BTST50*; E, *BTST81*; F, *BTST107*; G, *BTST111*; H, *BTST120*. B. PCR amplication of full-length coding sequences of *BTST134*. I, *BTST134.*
