# Supplementary material for: Genome-Wide Characterization and Expression Profiling of Sugar Transporter Family in the Whitefly, Bemisia tabaci (Gennadius) (Hemiptera: Aleyrodidae)
Source: Front Physiol. 2017 May 23;8:322. doi: 10.3389/fphys.2017.00322 (PMC5440588; doi:10.3389/fphys.2017.00322)
Supplement: Supplementary file 14 [file DataSheet3.DOCX]

**Figure S3. Cloning of partial-length coding sequences of 24 *BTSTs*.** Those *BTSTs* were randomly selected from the three groups which were divided based on the expressional levels across the whole development stages (Figure 3). The cDNA was prepared from total RNA obtained from from eggs, nymphs (first, second, third, and fourth instar), and newly emerged adults of *B. tabaci*, Primers were listed in Table S6. A: Group 1; B: Group 2; C: Group 3.
